# Digital and hybrid interventions for subacute and chronic low back pain: component network meta-analysis

**DOI:** 10.1038/s41746-026-03416-5

**Published:** 2026-11-04

**Authors:** Svenja Kaczorowski, Lars Donath, Patrick J. Owen, Nitin Kumar Arora, Tobias Saueressig, Hugo Pedder, Niamh L. Mundell, Tim Schleimer, Rebekka Döding, Daniel L. Belavy

**Affiliations:** 1 Department of Nursing, Midwifery and Therapeutic Sciences, Division of Physiotherapy, Bochum University of Applied Sciences, Bochum, 44801 Germany; 2 Department of Intervention Research in Exercise Training, German Sport University, Cologne, 50933 Germany; 3 Eastern Health Emergency Medicine Program, Monash University, Melbourne, VIC Australia; 4 Eastern Health Clinical School, Monash University, Clayton, 3800 VIC Australia; 5 Physio Meets Science GmbH, 69181 Leimen, Germany; 6 Bristol Medical School, Population Health Sciences, University of Bristol, Bristol, BS8 1TH UK; 7 Institute for Physical Activity and Nutrition (IPAN), School of Exercise and Nutrition Sciences, Deakin University, Geelong, 3220 VIC Australia; 8 Graduate School for Applied Research in North Rhine-Westphalia (Graduate School NRW), 44801 Bochum, Germany

**Keywords:** Diseases, Health care, Medical research

## Abstract

Digital and hybrid interventions combining exercise, education, and psychological components are increasingly used for low back pain (LBP), but it remains unclear which components and combinations drive efficacy. We conducted a prospectively registered component network meta-analysis (CNMA) to evaluate their efficacy and safety for non-specific LBP. Six databases and two trial registries were searched to May 2025. Data on disability, pain intensity, and adverse events were analysed using a Bayesian random-effects CNMA. Of 2809 records, 56 reports of 46 studies (6717 participants; 80% chronic low back pain) were included. Hybrid interventions combining digital and in-person exercise with in-person psychological components (MD: –22.21; 95%-CrI: –34.11 to –10.67), digital and in-person exercise (MD: –15.75; 95%-CrI: –22.79 to –8.80), or digital exercise with physiotherapy (MD: –13.17; 95%-CrI: –21.18 to –5.16) reduced disability versus no intervention at short-term (minimal clinically important difference (MCID): 4.92/100 Oswestry Disability Index). Digital and in-person exercise (MD: -1.76; 95%-CrI: -2.62; -0.87) and combined digital exercise training with in-person education (MD: -1.73; 95%-CrI: -2.71; -0.83) improved pain intensity beyond the MCID (0.82/10 numeric pain rating scale). No serious adverse events were noted. Multicomponent hybrid interventions appear safe and may provide clinically meaningful improvements for chronic LBP.

## Introduction

Digital interventions for low back pain (LBP) often integrate multiple treatment components, such as exercise training, education and psychological therapies, which are guideline-recommended treatments for LBP, each of which represent a distinct therapeutic approach. However, prior meta-analyses have overlooked this complexity by focusing on either individual components (e.g., psychological treatments alone)^1^ or grouping multiple components together (e.g., combining exercise training, education, and psychological therapies in one analysis)^2^. This distinction is crucial because treatment components span different therapeutic disciplines and may have distinct or synergistic effects when combined^3^. Furthermore, guideline-recommended management of acute LBP differs substantially from subacute and chronic LBP: acute LBP is usually self-limiting and is primarily managed with reassurance, advice to remain active, and avoidance of low-value care, whereas exercise and psychosocial interventions are mainly recommended for persistent/chronic LBP^4^. Given that LBP is highly prevalent, with 619 million people affected worldwide in 2020^5^, represents the leading cause of disability^5^, and imposes a substantial societal and economic burden^6^, optimizing treatment strategies and resource usage is considerable. It remains unclear which treatment components or combinations of components are most effective.

Although evidence supports the efficacy of digital interventions among patients with LBP for improving pain intensity and disability, our prior umbrella review^7^ of 19 systematic reviews identified limited knowledge on the comparative efficacy of different types of digitally delivered interventions among patients with LBP. However, these reviews generally evaluated interventions as whole entities and did not disentangle the effects of the individual treatment components. To date, no network meta-analysis has examined the relative contributions of these individual components and their combinations. Moreover, the quality of all included reviews for LBP^1,2,8–11^ was rated critically low or low, partly due to heterogeneous pooling of combinations of components. Such approaches limit the ability to disentangle the contribution of individual treatment components, a methodological challenge that component network meta-analysis is specifically designed to address. Thus, a lack of evidence-based guidance on which digital intervention components are most efficacious poses a limitation for clinicians to tailor treatments to patient needs.

Component network meta-analysis (CNMA) extends traditional meta-analysis by comparing not only complex interventions as a whole, but also the individual components they consist of within a single analysis^12^. In CNMA, complex interventions are broken down into their components, and the overall effect of a combined treatment can be understood as the contribution of its individual parts^12^. This makes it possible to identify which components of an intervention are effective and which are not^13^. Importantly, CNMA more closely reflects real-world clinical practice, where clinicians often combine different treatment components based on individual patient needs. By explicitly accounting for such combinations and their contributions, CNMA accommodates this variability and can provide more targeted and clinically relevant recommendations for digital interventions.

CNMA has been used in the field of early psychosis to disentangle the effects of different psychological intervention components when delivered alone or in combination^14^. Similarly, in the context of LBP, CNMA can help determine whether exercise training, education, or psychological therapies provide the greatest benefit when delivered via digital or hybrid delivery modes. CNMA also allows assessing whether combining those components enhances efficacy beyond monotherapy. Further, by incorporating both indirect and direct evidence, the comparative efficacy can be estimated when specific interventions have not been directly compared in primary studies^15^. The aim of this CNMA was to evaluate the comparative efficacy of digitally and hybrid delivered interventions for adults with subacute and chronic non-specific LBP in reducing pain intensity and disability. Further, we examined the safety of digitally and hybrid delivered interventions by summarizing adverse events reported in RCTs.

## Results

### Study selection

**Fig. 1:**
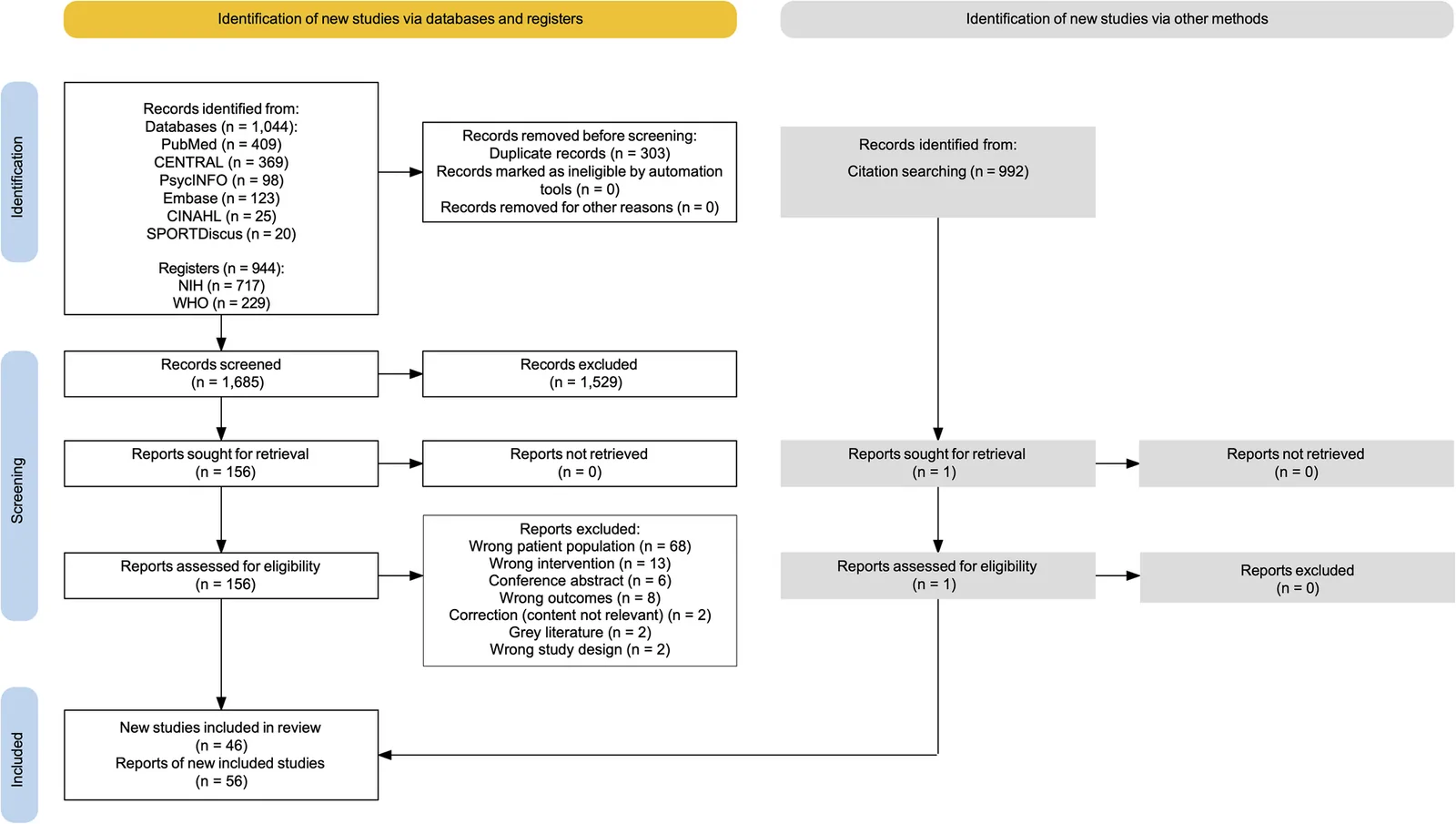
Prisma flow chart diagram. The diagram shows the number of reports found in search, screened in each stage and included in the review.

The search yielded 2980 articles, from which 303 were automatically identified as duplicates. From 1686 screened articles, 157 articles were included in the full text screening. We finally included 56 records of 46 unique studies, representing 6717 participants (Fig. 1). The reasons for exclusion at the full text stage are displayed in Supplemental Data 12. We contacted authors of 20 studies for further information, of which 12 authors replied, and nine could provide information and/or data, while three were not able to provide the requested data (Supplemental Data 13).

### Presentation of network structure

**Fig. 2:**
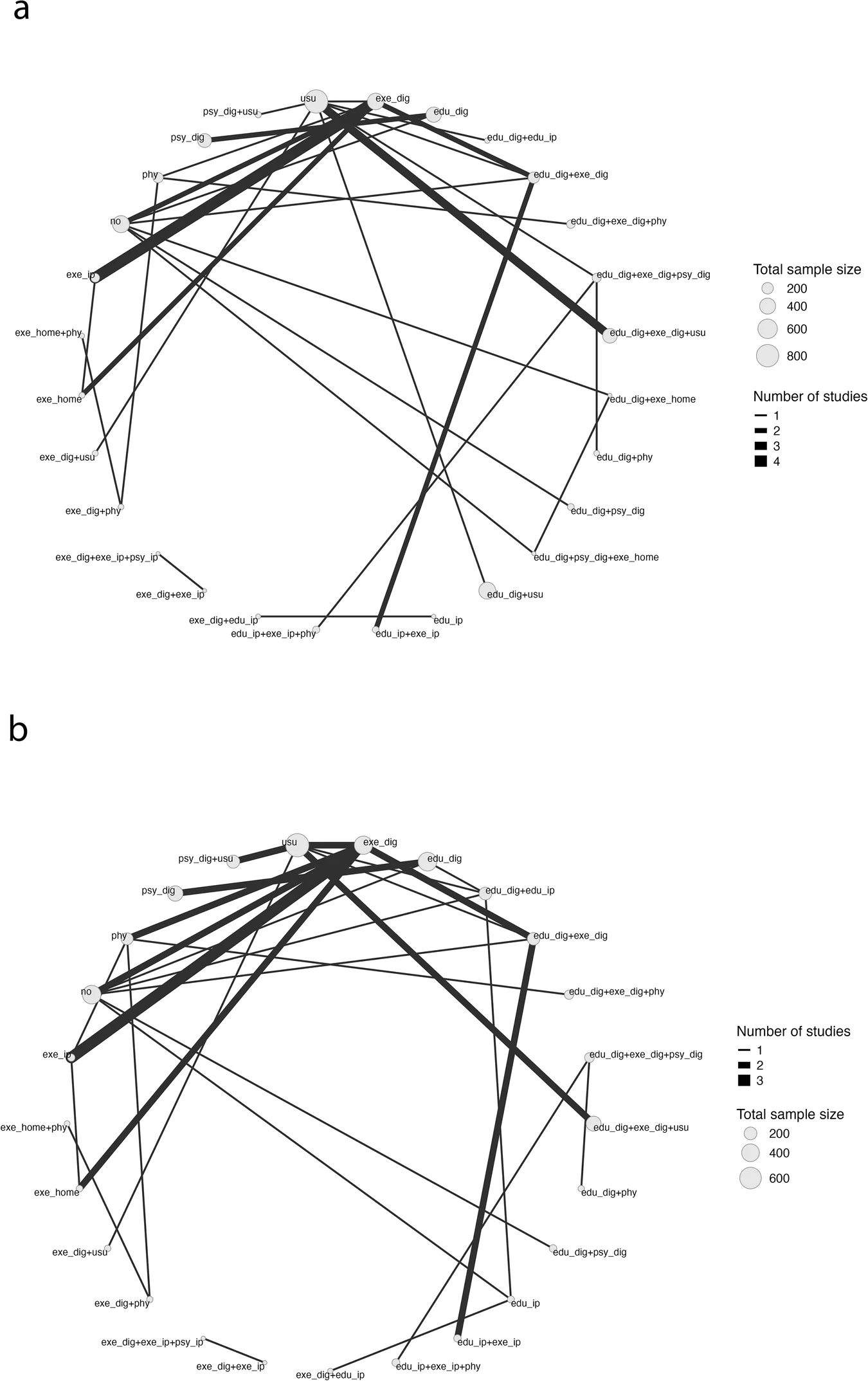
Network plots at the immediate post-intervention timepoint. **a** Network plot for disability at the immediate time-point; circles represent the total sample size at the node; thickness of the lines represents the number of studies per contrast; edu = education, exe = exercise, psy = psychological, phy = physiotherapy, usu = usual care, no = no intervention, dig = digital, ip = in-person, home = at home. **b** Network plot for pain intensity at the immediate time-point; circles represent the total sample size at the node; thickness of the lines represents the number of studies per contrast; edu = education, exe = exercise, psy = psychological, phy = physiotherapy, usu = usual care, no = no intervention, dig = digital, ip = in-person, home = at home.

**Fig. 3:**
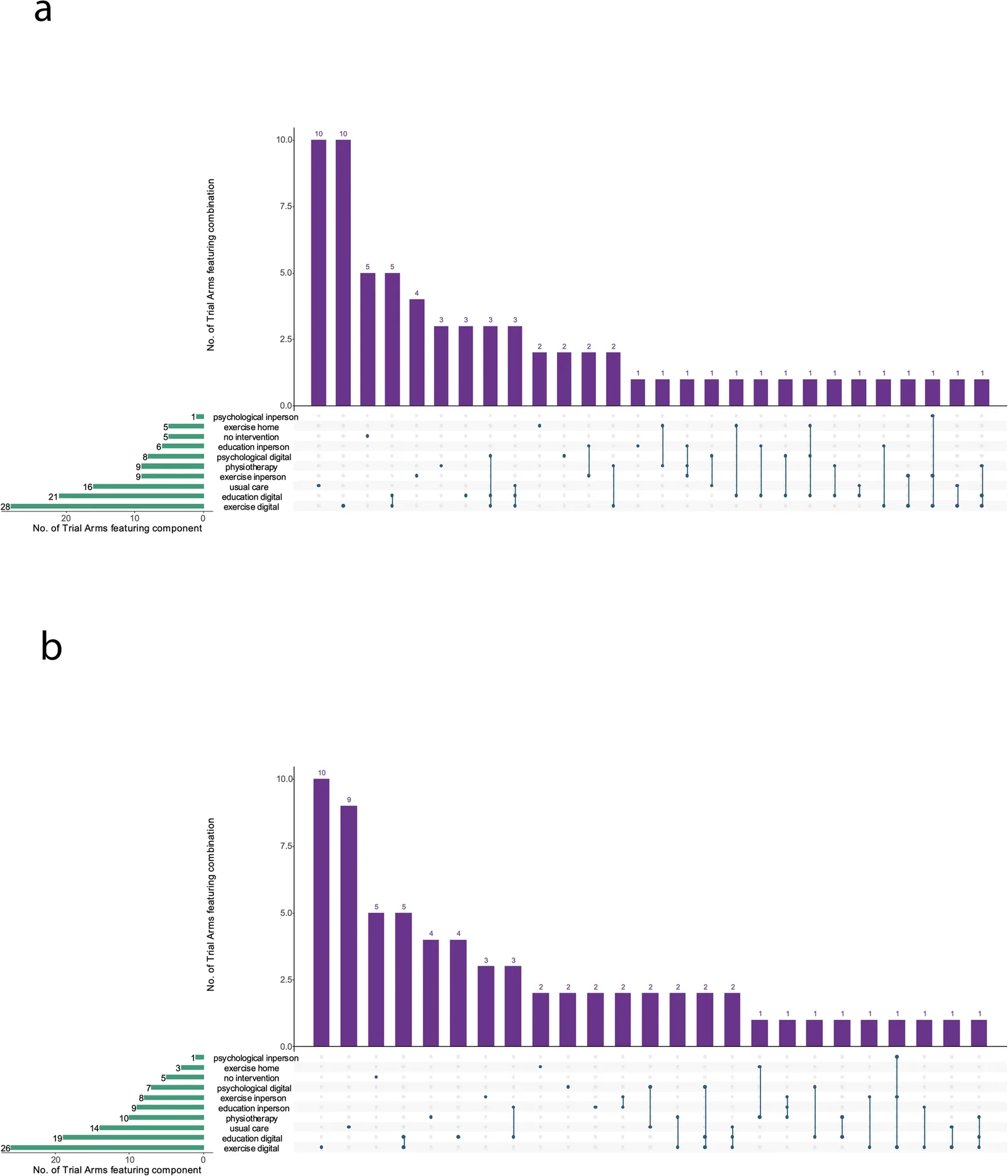
UpSet plots. **a** UpSet plot for disability at the immediate time-point showing the distribution of components and their combination; each column in the dot-matrix represents an intervention (combination of components) with vertical lines indicating common components; the horizontal bar chart shows the number of arms including each component, the vertical bar chart shows the number of arms including each intervention. **b** UpSet plot for pain intensity at the immediate time-point showing the distribution of components and their combination; each column in the dot-matrix represents an intervention (combination of components) with vertical lines indicating common components; the horizontal bar chart shows the number of arms including each component, the vertical bar chart shows the number of arms including each intervention.

We present the network structure for the standard NMA model for both outcomes (Fig. 2) as well as the structure of the components within the network for both outcomes (Fig. 3) for the immediate post-intervention time-point. Although follow-up time points were prespecified, the available data were insufficient to support network meta-analyses at these time points due to poor network connectivity. The corresponding network graphs are therefore provided in Supplemental Data 14 without further quantitative synthesis.

### Summary of network geometry

The network for disability at the immediate post-intervention timepoint included a total of 33 studies, while the same network for pain intensity included a total of 32 studies. One study^16^ was excluded from the quantitative analysis. This study was a small pilot RCT that produced very large effect estimates (pain intensity; mean difference [95%-confidence interval] compared to placebo: −4.0 [−5.44; −2.55]) compared to all other studies in the network, suggesting a likely violation of the transitivity assumption. Overall, a total of 45 studies were included in the analysis, comprising 38 two-arm and seven three-arm studies.

Most studies (k = 36, 80%) included patients with chronic pain, one study included sub-acute patients, six studies included a combination of sub-acute and acute pain, and two studies did not pre-specify the pain duration, but the final sample consisted of chronic pain patients. As only a small number of study authors provided data on the actual pain duration of the included population, it was not possible to present a meaningful average pain duration (range: 20.7–964.7 weeks). The median sample size was 98 (range 8–825), the mean age of the participants included in the analysis was 48 years (standard deviation (SD) = 8.9), and 56.9% (*n* = 3819) of the participants were female.

The duration of the intervention ranged from one day to one year, with a mean duration of 10.2 weeks. On the intervention level, digital exercise training was used the most (k = 10), followed by combined digital education digital and exercise training digital (k = 6) and digital education (k = 6). The number of studies conducted per intervention is provided in Supplemental Data 15. On the component level, digital exercise training was the most frequently used component in 33 arms, followed by digital education in 31 arms, while digital psychological intervention was used in 11 arms. The various delivery pathways and modes employed in each study are presented in Table 1. In-person components were typically delivered in clinical or physiotherapy practice settings or at home, while digital components were delivered via mobile applications, websites, videoconferencing platforms, or telephone. Of the 97 intervention arms, 58 included a single-component intervention, 28 included a two-component intervention, and 11 included a three-component intervention.

**Table 1:** Characteristics of included studies

Author, year	Study design	Pain type	*N* arms	Interventions	Pathway	Mode	Outcomes	Timepoints	N randomized	Mean age	N (%) female	Country	Funding
Akodu 2024^128^	Parallel trial	chronic	2	Arm 1: Exercise in-person Arm 2: Exercise digital	Arm 1: Practice Arm 2: Combination	Arm 1: Synchronous Arm 2: Combination	Disability	immediate	54 Arm 1: 27 Arm 2: 27	41.1 Arm 1: 48.9 Arm 2: 33.3	25 (46.3%) Arm 1: 13 Arm 2: 12	Nigeria	no funding
Almhdawi 2020^16^	Parallel trial	chronic	2	Arm 1: Education digital + Exercise digital Arm 2: placebo	Arm 1: App / App Arm 2: App	Arm 1: Asynchronous / Asynchronous Arm 2: Asynchronous	Pain intensity Disability	immediate > 1 week, less than 3 months > 3 months, less than 1 year	41 Arm 1: 21 Arm 2: 20	41.1 Arm 1: 40.5 Arm 2: 41.7	22 (53.7%) Arm 1: 14 Arm 2: 8	Jordan	public funding
Amorim 2019^17^	Parallel trial	chronic	2	Arm 1: Exercise digital + Education in-person Arm 2: Education in-person	Arm 1: Home / Combination Arm 2: Home	Arm 1: Combination / Combination Arm 2: Combination	Pain intensity Disability	immediate > 1 week, less than 3 months > 3 months, less than 1 year	68 Arm 1: 34 Arm 2: 34	58.3 Arm 1: 59.5 Arm 2: 57.1	34 (50%) Arm 1: 15 Arm 2: 19	Australia	public funding
Bayattork 2022^129^	Parallel trial	chronic	3	Arm 1: Exercise digital Arm 2: Exercise in-person Arm 3: Physiotherapy	Arm 1: Videoconference Arm 2: Practice Arm 3: Practice	Arm 1: Synchronous Arm 2: Synchronous Arm 3: Synchronous	Pain intensity	immediate > 1 week, less than 3 months > 3 months, less than 1 year	60 Arm 1: 20 Arm 2: 20 Arm 3: 20	37 Arm 1: 38.5 Arm 2: 35.1 Arm 3: 37.3	NA	Iran	no funding
Bernardelli 2020^130^	Parallel trial	chronic	2	Arm 1: Exercise in-person Arm 2: Exercise digital	Arm 1: Practice Arm 2: Videos only	Arm 1: Synchronous Arm 2: Asynchronous	Disability	immediate	101 Arm 1: 51 Arm 2: 50	51.2 Arm 1: 50.5 Arm 2: 51.9	63 (62.4%) Arm 1: 34 Arm 2: 29	Italy	no funding
Carpenter 2012^131^	Parallel trial	chronic	2	Arm 1: Education digital + Psychological digital Arm 2: No intervention	Arm 1: Website / Website	Arm 1: Asynchronous / Asynchronous	Pain intensity Disability	immediate > 1 week, less than 3 months > 3 months, less than 1 year	141 Arm 1: 70 Arm 2: 71	42.5 Arm 1: 42.5 Arm 2: 42.5	NA	United States	public funding
Chhabra 2018^132^	Parallel trial	chronic	2	Arm 1: Exercise digital + Usual Care Arm 2: Usual Care	Arm 1: App	Arm 1: Asynchronous	Pain intensity Disability	immediate > 1 week, less than 3 months > 3 months, less than 1 year	93 Arm 1: 45 Arm 2: 48	41.2 Arm 1: 41.4 Arm 2: 41	NA	India	industry or private funding
Chiauzzi 2010^133^	Parallel trial	chronic	2	Arm 1: Education digital Arm 2: Psychological digital	Arm 1: Mail Arm 2: Website	Arm 1: Asynchronous Arm 2: Asynchronous	Pain intensity Disability	immediate > 1 week, less than 3 months > 3 months, less than 1 year	209 Arm 1: 105 Arm 2: 104	46.2 Arm 1: 45.1 Arm 2: 47.3	134 (64.1%) Arm 1: 70 Arm 2: 64	United States	public funding
Cui 2023^20^	Parallel trial	chronic	2	Arm 1: Education digital + Exercise digital + Psychological digital Arm 2: Education in-person + Exercise in-person + Physiotherapy	Arm 1: App / App / Mail Arm 2: Practice / Practice / Practice	Arm 1: Asynchronous / Asynchronous / Asynchronous Arm 2: Synchronous / Synchronous / Synchronous	Pain intensity Disability	immediate > 1 week, less than 3 months > 3 months, less than 1 year	140 Arm 1: 70 Arm 2: 70	52.5 Arm 1: 50.5 Arm 2: 54.5	95 (67.9%) Arm 1: 46 Arm 2: 49	United States	no funding
Day 2024^134^	Parallel trial	chronic	2	Arm 1: Psychological digital Arm 2: Education digital	Arm 1: Videoconference Arm 2: Videoconference	Arm 1: Synchronous Arm 2: Synchronous	Pain intensity Disability	immediate > 1 week, less than 3 months > 3 months, less than 1 year	302 Arm 1: 201 Arm 2: 101	49.6 Arm 1: 48.1 Arm 2: 51	232 (76.8%) Arm 1: 154 Arm 2: 78	United States	public funding
DelPozo-Cruz 2012^135^	Parallel trial	sub-acute	2	Arm 1: Usual Care Arm 2: Education digital + Exercise digital + Usual Care	Arm 2: Videos only / Videos only	Arm 2: Asynchronous / Asynchronous	Pain intensity Disability	immediate > 1 week, less than 3 months > 3 months, less than 1 year	100 Arm 1: 50 Arm 2: 50	46.2 Arm 1: 45.9 Arm 2: 46.6	85 (85%) Arm 1: 42 Arm 2: 43	Spain	public funding
Fanuscu 2024^136^	Parallel trial	chronic	2	Arm 1: Exercise in-person Arm 2: Exercise digital	Arm 1: Practice Arm 2: Videos only	Arm 1: Synchronous Arm 2: Asynchronous	Pain intensity Disability	immediate > 1 week, less than 3 months > 3 months, less than 1 year	42 Arm 1: 21 Arm 2: 21	39.7 Arm 1: 40.7 Arm 2: 38.6	27 (64.3%) Arm 1: 14 Arm 2: 13	Turkey	not reported
Garreta-Catala 2023^137^	Parallel trial	chronic	2	Arm 1: Education digital + Exercise digital + Psychological digital Arm 2: Usual Care	Arm 1: Videoconference / Videoconference / Videoconference	Arm 1: Synchronous / Synchronous / Synchronous	Disability	immediate > 3 months, less than 1 year	18 Arm 1: 9 Arm 2: 9	53.2 Arm 1: 51.1 Arm 2: 55.2	13 (72.2%) Arm 1: 8 Arm 2: 5	Spain	no funding
Geraghty 2018^138^	Parallel trial	not specified	2	Arm 1: Education digital + Usual Care Arm 2: Usual Care	Arm 1: Website	Arm 1: Asynchronous	Pain intensity Disability	immediate > 1 week, less than 3 months > 3 months, less than 1 year	87 Arm 1: 59 Arm 2: 28	58.6 Arm 1: 56.9 Arm 2: 60.3	51 (58.6%) Arm 1: 36 Arm 2: 15	United Kingdom	public funding
Geraghty 2024^139^	Parallel trial	combination	2	Arm 1: Education digital + Usual Care Arm 2: Usual Care	Arm 1: Website	Arm 1: Asynchronous	Pain intensity Disability	immediate > 1 week, less than 3 months > 3 months, less than 1 year	825 Arm 1: 551 Arm 2: 274	54.3 Arm 1: 54 Arm 2: 54.5	479 (58.1%) Arm 1: 321 Arm 2: 158	United Kingdom	public funding
Goode 2018^140^	Parallel trial	chronic	3	Arm 1: Education digital + Exercise home Arm 2: Education digital + Psychological digital + Exercise home Arm 3: No intervention	Arm 1: Home / Telephone Arm 2: Telephone / Home / Telephone	Arm 1: Asynchronous / Synchronous Arm 2: Synchronous / Asynchronous / Synchronous	Disability	immediate	60 Arm 1: 20 Arm 2: 20 Arm 3: 20	70.3 Arm 1: 69.6 Arm 2: 69.5 Arm 3: 71.9	4 (6.7%) Arm 1: 1 Arm 2: 2 Arm 3: 1	United States	public funding
Hodges 2021^141^	Parallel trial	not specified	2	Arm 1: No intervention Arm 2: Education digital	Arm 2: Website	Arm 2: Asynchronous	Pain intensity Disability	immediate > 1 week, less than 3 months > 3 months, less than 1 year	440 Arm 1: 226 Arm 2: 214	48 Arm 1: 47.8 Arm 2: 48.1	335 (76.1%) Arm 1: 174 Arm 2: 161	Australia	public funding
Itoh 2022^142^	Parallel trial	chronic	2	Arm 1: Education digital + Exercise digital + Usual Care Arm 2: Usual Care	Arm 1: App / App	Arm 1: Asynchronous / Asynchronous	Pain intensity Disability	immediate > 1 week, less than 3 months > 3 months, less than 1 year	101 Arm 1: 50 Arm 2: 51	47.4 Arm 1: 47.9 Arm 2: 46.9	44 (43.6%) Arm 1: 21 Arm 2: 23	Japan	industry or private funding
Karaduman 2023^143^	Parallel trial	chronic	3	Arm 1: Exercise in-person Arm 2: Exercise digital Arm 3: Exercise home	Arm 1: Practice Arm 2: Videoconference Arm 3: Home	Arm 1: Synchronous Arm 2: Synchronous Arm 3: Asynchronous	Pain intensity Disability	immediate > 1 week, less than 3 months > 3 months, less than 1 year	66 Arm 1: 22 Arm 2: 22 Arm 3: 22	45.6 Arm 1: 46.5 Arm 2: 46.3 Arm 3: 44	30 (45.5%) Arm 1: 2 Arm 2: 13 Arm 3: 15	Turkey	no funding
Kazemi 2021^144^	Cluster trial	combination	3	Arm 1: Education in-person + Exercise in-person Arm 2: Education digital + Exercise digital Arm 3: No intervention	Arm 1: Practice / Practice Arm 2: Website / Website	Arm 1: Synchronous / Synchronous Arm 2: Asynchronous / Asynchronous	Pain intensity Disability	immediate > 1 week, less than 3 months > 3 months, less than 1 year	180 Arm 1: 60 Arm 2: 60 Arm 3: 60	36.7 Arm 1: 36 Arm 2: 37 Arm 3: 37	180 (100%) Arm 1: 60 Arm 2: 60 Arm 3: 60	Iran	not reported
Kohns 2020^145^	Parallel trial	chronic	2	Arm 1: Psychological digital Arm 2: Education digital	Arm 1: Combination Arm 2: Combination	Arm 1: Asynchronous Arm 2: Asynchronous	Pain intensity	immediate > 1 week, less than 3 months > 3 months, less than 1 year	54 Arm 1: 28 Arm 2: 26	45.5 Arm 1: 45.4 Arm 2: 45.7	34 (63%) Arm 1: 14 Arm 2: 20	United States	public funding
Koppenaal 2022^146^	Cluster trial	combination	2	Arm 1: Education digital + Exercise digital + Physiotherapy Arm 2: Physiotherapy	Arm 1: App / App / Practice Arm 2: Practice	Arm 1: Asynchronous / Asynchronous / Synchronous Arm 2: Synchronous	Pain intensity Disability	immediate > 1 week, less than 3 months > 3 months, less than 1 year	208 Arm 1: 104 Arm 2: 104	47.7 Arm 1: 48.1 Arm 2: 47.3	102 (49%) Arm 1: 45 Arm 2: 57	Netherlands	public funding
Krein 2013^147^	Parallel trial	chronic	2	Arm 1: Education digital + Exercise digital Arm 2: Usual Care	Arm 1: Website / Website	Arm 1: Asynchronous / Asynchronous	Pain intensity Disability	immediate > 1 week, less than 3 months > 3 months, less than 1 year	229 Arm 1: 111 Arm 2: 118	51.5 Arm 1: 51.2 Arm 2: 51.9	28 (12.2%) Arm 1: 12 Arm 2: 16	United States	public funding
Lara-Palomo 2022^148^	Parallel trial	chronic	2	Arm 1: Exercise digital + Physiotherapy Arm 2: Exercise home+ Physiotherapy	Arm 1: App / Home Arm 2: Home / Home	Arm 1: Asynchronous / Asynchronous Arm 2: Asynchronous / Asynchronous	Pain intensity Disability	immediate > 1 week, less than 3 months > 3 months, less than 1 year	80 Arm 1: 40 Arm 2: 40	48.2 Arm 1: 41.9 Arm 2: 54.6	43 (53.8%) Arm 1: 22 Arm 2: 21	Spain	public funding
Licciardone 2020^149^	Parallel trial	chronic	2	Arm 1: Education digital + Usual Care Arm 2: Usual Care	Arm 1: Mail	Arm 1: Asynchronous	Pain intensity Disability	immediate > 1 week, less than 3 months > 3 months, less than 1 year	102 Arm 1: 52 Arm 2: 50	51 Arm 1: 51.3 Arm 2: 50.7	86 (84.3%) Arm 1: 42 Arm 2: 44	United States	public funding
Licciardone 2022^150^	Parallel trial	chronic	2	Arm 1: Education digital + Usual Care Arm 2: Usual Care	Arm 1: Mail	Arm 1: Asynchronous	Pain intensity Disability	immediate > 1 week, less than 3 months > 3 months, less than 1 year	331 Arm 1: 166 Arm 2: 165	51 Arm 1: 51.9 Arm 2: 50.2	249 (75.2%) Arm 1: 122 Arm 2: 127	United States	public funding
López-Marcos 2024^151^	Parallel trial	chronic	2	Arm 1: Exercise digital + Education in-person + Exercise in-person Arm 2: Exercise digital	Arm 1: App / Practice / Practice Arm 2: App	Arm 1: Asynchronous / Synchronous / Synchronous Arm 2: Asynchronous	Pain intensity Disability	immediate > 1 week, less than 3 months > 3 months, less than 1 year	98 Arm 1: 49 Arm 2: 49	51 Arm 1: 52.2 Arm 2: 49.9	74 (75.5%) Arm 1: 34 Arm 2: 40	Spain	no funding
Mbada 2019^152^	Parallel trial	chronic	2	Arm 1: Education digital + Exercise digital Arm 2: Education in-person + Exercise in-person	Arm 1: App / App Arm 2: Practice / Home	Arm 1: Asynchronous / Asynchronous Arm 2: Synchronous / Asynchronous	Pain intensity Disability	immediate > 1 week, less than 3 months > 3 months, less than 1 year	56 Arm 1: 24 Arm 2: 32	48.6 Arm 1: 47.3 Arm 2: 50	34 (60.7%) Arm 1: 14 Arm 2: 20	Nigeria	public funding
Mesa-Castrillon 2023^153^	Parallel trial	chronic	2	Arm 1: Exercise digital Arm 2: Usual Care	Arm 1: App	Arm 1: Combination	Pain intensity Disability	immediate > 1 week, less than 3 months > 3 months, less than 1 year	97 Arm 1: 47 Arm 2: 50	61 Arm 1: 61 Arm 2: 61	53 (54.6%) Arm 1: 31 Arm 2: 22	Australia	public funding
Okudan 2024^154^	Parallel trial	chronic	3	Arm 1: Exercise digital Arm 2: Education digital + Exercise digital Arm 3: No intervention	Arm 1: Videoconference Arm 2: Videoconference / Videoconference	Arm 1: Synchronous Arm 2: Synchronous / Synchronous	Pain intensity Disability	immediate > 1 week, less than 3 months > 3 months, less than 1 year	51 Arm 1: 17 Arm 2: 17 Arm 3: 17	52 Arm 1: 52.3 Arm 2: 52.9 Arm 3: 50.8	21 (41.2%) Arm 1: 11 Arm 2: 6 Arm 3: 4	Turkey	no funding
Park 2023^155^	Parallel trial	chronic	2	Arm 1: Exercise digital Arm 2: Physiotherapy	Arm 1: App Arm 2: Practice	Arm 1: Asynchronous Arm 2: Synchronous	Pain intensity Disability	immediate > 1 week, less than 3 months > 3 months, less than 1 year	100 Arm 1: 50 Arm 2: 50	35.2 Arm 1: 37.1 Arm 2: 33.2	40 (40%) Arm 1: 20 Arm 2: 20	South Korea	public funding
Petrozzi 2019^156^	Parallel trial	chronic	2	Arm 1: Usual Care Arm 2: Psychological digital + Usual Care	Arm 1: Practice Arm 2: Website	Arm 1: Synchronous Arm 2: Asynchronous	Pain intensity Disability	immediate > 1 week, less than 3 months > 3 months, less than 1 year	108 Arm 1: 54 Arm 2: 54	50.4 Arm 1: 50.6 Arm 2: 50.1	54 (50%) Arm 1: 25 Arm 2: 29	Australia	no funding
Pongsatornsaiklang 2025^157^	Parallel trial	chronic	2	Arm 1: Exercise digital Arm 2: Usual Care	Arm 1: Videoconference	Arm 1: Synchronous	Pain intensity	immediate > 1 week, less than 3 months > 3 months, less than 1 year	54 Arm 1: 36 Arm 2: 18	20.6 Arm 1: 20.2 Arm 2: 21	29 (53.7%) Arm 1: 20 Arm 2: 9	Thailand	public funding
Sanabria-Mazo 2023^158^	Parallel trial	chronic	2	Arm 1: Psychological digital + Usual Care Arm 2: Usual Care	Arm 1: Videoconference	Arm 1: Synchronous	Pain intensity	immediate > 1 week, less than 3 months > 3 months, less than 1 year	234 Arm 1: 156 Arm 2: 78	54.3 Arm 1: 54.9 Arm 2: 53.8	158 (67.5%) Arm 1: 107 Arm 2: 51	Spain	public funding
Sandal 2021^159^	Parallel trial	combination	2	Arm 1: Usual Care Arm 2: Education digital + Exercise digital + Usual Care	Arm 2: App / App	Arm 2: Asynchronous / Asynchronous	Pain intensity Disability	immediate > 1 week, less than 3 months > 3 months, less than 1 year	461 Arm 1: 229 Arm 2: 232	47.5 Arm 1: 46.7 Arm 2: 48.3	255 (55.3%) Arm 1: 134 Arm 2: 121	Denmark + Norway	public funding
Schaller 2016^160^	Parallel trial	chronic	2	Arm 1: Education digital + Education in-person Arm 2: Education digital	Arm 1: Practice / Combination Arm 2: Website	Arm 1: Synchronous / Combination Arm 2: Asynchronous	Pain intensity	immediate > 1 week, less than 3 months > 3 months, less than 1 year	412 Arm 1: 201 Arm 2: 211	50.4 Arm 1: 49.7 Arm 2: 51.1	126 (30.6%) Arm 1: 58 Arm 2: 68	Germany	industry or private funding
Shaygan 2022^161^	Parallel trial	chronic	3	Arm 1: Education in-person Arm 2: Education digital + Education in-person Arm 3: No intervention	Arm 1: Practice Arm 2: Videos only / Practice	Arm 1: Synchronous Arm 2: Asynchronous / Synchronous	Pain intensity	immediate > 1 week, less than 3 months > 3 months, less than 1 year	90 Arm 1: 30 Arm 2: 30 Arm 3: 30	50.5 Arm 1: 47.4 Arm 2: 51 Arm 3: 53.2	21 (23.3%) Arm 1: 6 Arm 2: 5 Arm 3: 10	Iran	public funding
Skolasky 2024^162^	Parallel trial	chronic	3	Arm 1: No intervention Arm 2: Education digital Arm 3: Education digital + Psychological digital	Arm 2: Website Arm 3: Website / Telephone	Arm 2: Synchronous Arm 3: Synchronous / Synchronous	Disability	> 1 week, less than 3 months > 3 months, less than 1 year	34 Arm 1: 12 Arm 2: 4 Arm 3: 18	44 Arm 1: 44 Arm 2: 41 Arm 3: 47	19 (55.9%) Arm 1: 4 Arm 2: 3 Arm 3: 12	United States	public funding
Toelle 2019^19^	Parallel trial	combination	2	Arm 1: Education digital + Exercise digital + Psychological digital Arm 2: Education digital + Physiotherapy	Arm 1: App / App / App Arm 2: Practice / Mail	Arm 1: Asynchronous / Asynchronous / Asynchronous Arm 2: Synchronous / Asynchronous	Pain intensity Disability	immediate > 1 week, less than 3 months > 3 months, less than 1 year	101 Arm 1: 53 Arm 2: 48	42 Arm 1: 41 Arm 2: 43	66 (65.3%) Arm 1: 35 Arm 2: 31	Germany	industry or private funding
Villatoro-Luque 2023^163^	Parallel trial	chronic	2	Arm 1: Education digital + Exercise digital Arm 2: Education in-person + Exercise in-person	Arm 1: Videos only / Website Arm 2: Practice / Practice	Arm 1: Asynchronous / Asynchronous Arm 2: Synchronous / Synchronous	Pain intensity Disability	immediate > 1 week, less than 3 months > 3 months, less than 1 year	78 Arm 1: 39 Arm 2: 39	43.4 Arm 1: 42.4 Arm 2: 44.4	36 (46.2%) Arm 1: 16 Arm 2: 20	Spain	no funding
Williams 2018^164^	Parallel trial	chronic	2	Arm 1: Education digital + Education in-person Arm 2: Usual Care	Arm 1: Practice / Telephone	Arm 1: Synchronous / Synchronous	Pain intensity Disability	immediate > 1 week, less than 3 months > 3 months, less than 1 year	160 Arm 1: 80 Arm 2: 80	56.7 Arm 1: 56 Arm 2: 57.4	94 (58.8%) Arm 1: 48 Arm 2: 46	Australia	public funding
Yang 2019^165^	Parallel trial	chronic	2	Arm 1: Exercise digital + Physiotherapy Arm 2: Physiotherapy	Arm 1: App / Practice Arm 2: Practice	Arm 1: Asynchronous / Synchronous Arm 2: Synchronous	Pain intensity Disability	immediate > 1 week, less than 3 months > 3 months, less than 1 year	8 Arm 1: 5 Arm 2: 3	42.6 Arm 1: 35 Arm 2: 50.3	4 (50%) Arm 1: 1 Arm 2: 3	Hongkong	not reported
Yang 2024^166^	Parallel trial	combination	2	Arm 1: Exercise digital Arm 2: No intervention	Arm 1: Videoconference	Arm 1: Synchronous	Pain intensity Disability	immediate > 1 week, less than 3 months > 3 months, less than 1 year	350 Arm 1: 175 Arm 2: 175	58.8 Arm 1: 57.9 Arm 2: 59.7	278 (79.4%) Arm 1: 138 Arm 2: 140	United States	industry or private funding
Zheng 2022 a^167^	Parallel trial	chronic	2	Arm 1: Education digital + Exercise digital Arm 2: Exercise digital	Arm 1: App / Videoconference Arm 2: App	Arm 1: Asynchronous / Synchronous Arm 2: Asynchronous	Pain intensity Disability	immediate > 1 week, less than 3 months > 3 months, less than 1 year	40 Arm 1: 20 Arm 2: 20	34.5 Arm 1: 34 Arm 2: 34.9	26 (65%) Arm 1: 14 Arm 2: 12	China	public funding
Zheng 2022 b^18^	Parallel trial	chronic	2	Arm 1: Exercise digital + Exercise in-person + Psychological in-person Arm 2: Exercise digital + Exercise in-person	Arm 1: Practice / App / Practice Arm 2: Practice / App	Arm 1: Synchronous / Asynchronous / Synchronous Arm 2: Synchronous / Asynchronous	Pain intensity Disability	immediate > 1 week, less than 3 months > 3 months, less than 1 year	40 Arm 1: 20 Arm 2: 20	35.4 Arm 1: 31.5 Arm 2: 39.2	28 (70%) Arm 1: 14 Arm 2: 14	China	public funding
Özden 2022^168^	Parallel trial	chronic	2	Arm 1: Exercise digital Arm 2: Exercise home	Arm 1: Website Arm 2: Home	Arm 1: Combination Arm 2: Asynchronous	Pain intensity Disability	immediate > 1 week, less than 3 months > 3 months, less than 1 year	54 Arm 1: 27 Arm 2: 27	41.2 Arm 1: 40.1 Arm 2: 42.3	30 (55.6%) Arm 1: 14 Arm 2: 16	Turkey	no funding

Disability was assessed in 39 studies, and pain intensity was assessed in 40 studies. Adverse events were documented in 21 (47%) of the 45 studies.

Of the 45 distinct studies, 43 (96%) were parallel RCTs and two (4%) were cluster RCTs. Most studies (k = 17, 38%) were conducted in Europe, with 10 (22%) studies conducted in Asia, 11 (24%) in America, five (11%) in Australia, and two (4%) in Africa. The majority of studies (k = 26, 58%) were publicly funded, five (11%) were industry- or privately funded, 11 (24%) received no funding, and three (7%) did not report on funding. In 36 (80%) studies, the researchers declared that they had no conflict of interest. In six (13%) trials, a conflict of interest was declared, mostly regarding an involvement with the developing company of the app used in the trial. In three (7%) trials, a statement regarding a conflict of interest was missing.

### Study characteristics

A summary of the key characteristics of the trials is provided in Table 1.

### Risk of bias within studies

**Fig. 4:**
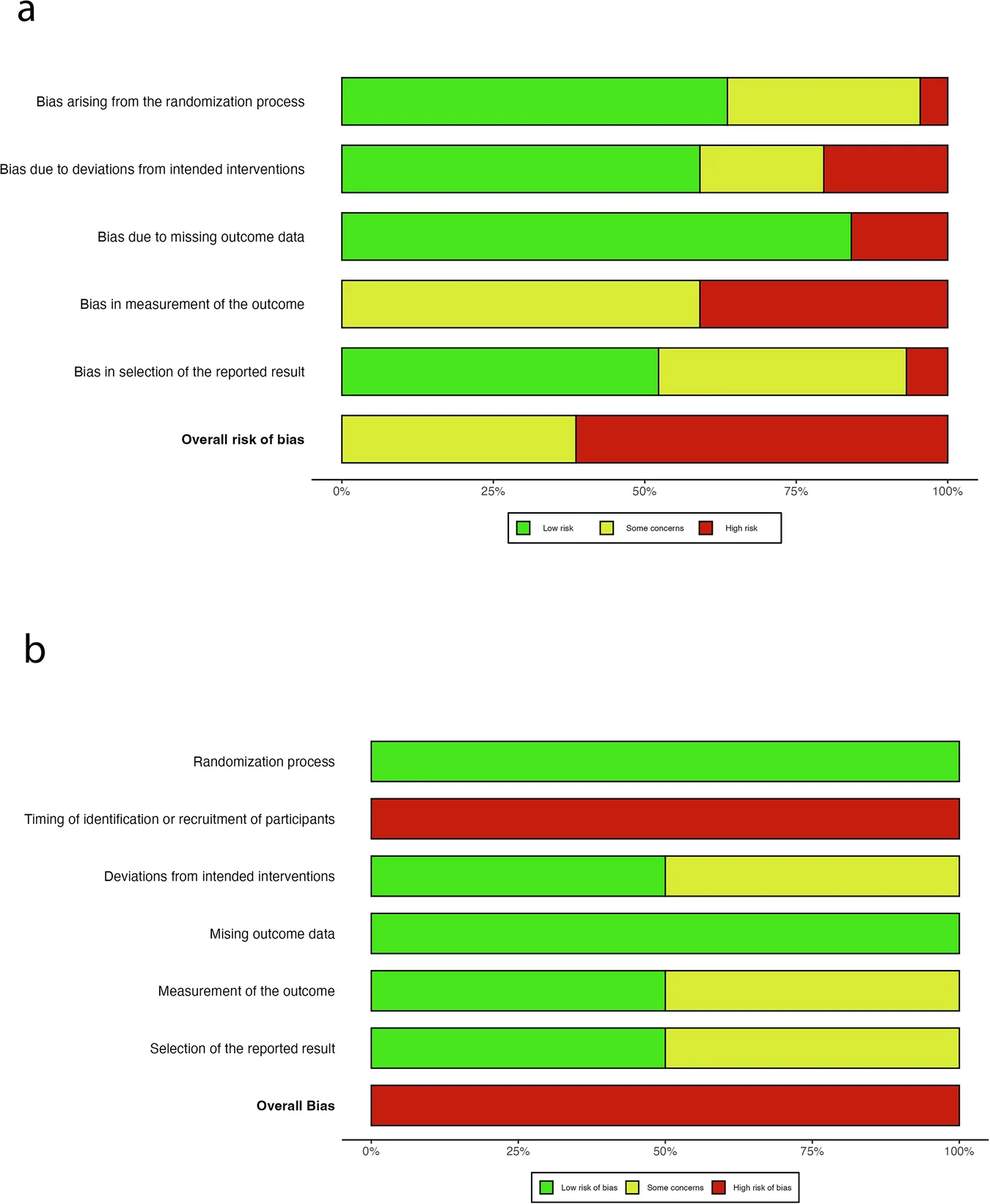
Risk of bias summary plots. **a** Risk of bias ratings (parallel trials) for the pain intensity outcome at the immediate time-point. For ratings on the individual study level, please refer to Supplemental Data 16. **b** Risk of bias ratings (cluster trials) for the pain intensity outcome at the immediate time-point. For ratings on the individual study level, please refer to Supplemental Data 16.

The results of the risk of bias assessment of the included trials are displayed in Fig. 4a for parallel trials and Fig. 4b for cluster trials. A rating of high risk of bias was concluded in 29 of 45 (64%) studies. The most critical domain was measurement of the outcome due to the lack of blinding of participants in combination with self-reported outcomes. Traffic plots and ratings for each item in each domain per study are available in Supplemental Data 16.

### Results of individual studies

Pairwise meta-analyses of all included comparisons for pain intensity and disability for all time points can be found in Supplemental Data 17.

### Synthesis of results

For disability immediate, the MCID of 0.5 SD was back transformed using the internal reference SD of the Oswestry Disability Index (ranging from 0 to 100), which resulted in an MCID of 4.92.

The single-effect NMA model on the intervention level was disconnected and split into three sub-networks (Fig. 2). The additive model connected all nodes and was used as the primary model because it showed better model fit (lower DIC) compared to the single-effect model, and lower model complexity compared to the two-way interaction model (Supplemental Data 18). A comparison of results between the single-effect NMA, the additive and the interaction model is displayed in Supplemental Data 19.

On the component level, no unique component exceeded the MCID compared to no intervention (Supplemental Data 20). The SUCRA ranking with the underlying ranking distribution is displayed in Supplemental Data 20.

**Fig. 5:**
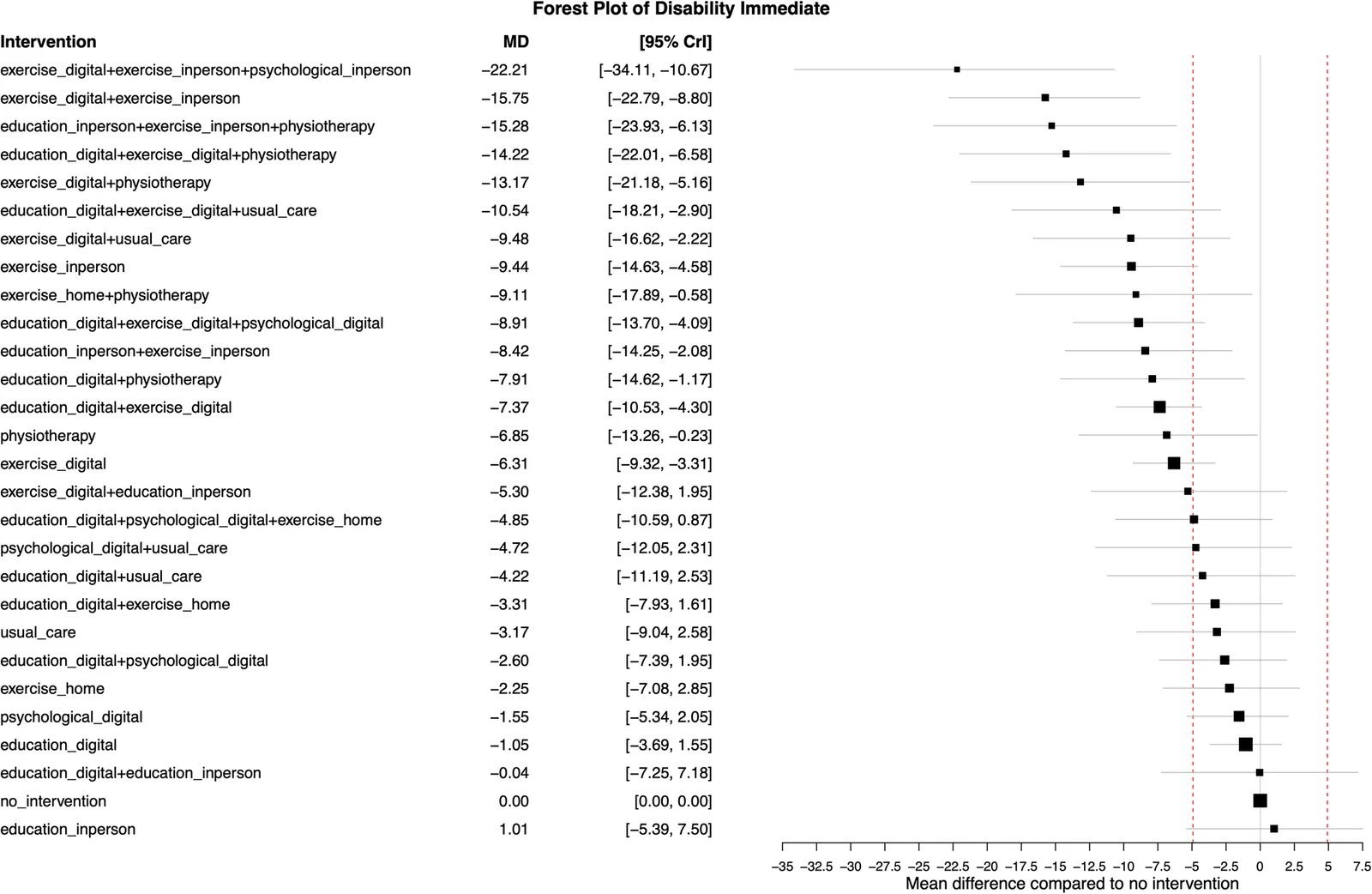
Forest plot of disability immediate. Forest plot of relative effects for the additive disability immediate network on the intervention level with 95%-credible intervals; red dotted lines represent the MCID of 4.92 for this outcome. MD = mean difference, CrI = credible interval.

On the intervention level, four combinations of components with at least one digital component showed clinically important differences compared to no intervention: combined digital and in-person exercise training with in-person psychological intervention (mean difference (MD): −22.21; 95%-credible interval (CrI): [−34.11; −10.67]), combined digital and in-person exercise training (MD: −15.75; 95%-CrI: [−22.79; −8.8]), combined digital education with digital exercise training and physiotherapy (MD: −14.22; 95%-CrI: [−22.01; −6.58]), and combined digital exercise training with physiotherapy (MD: −13.17; 95%-CrI: [−21.18; −5.16]) (Fig. 5, Supplemental Data 20). A table of all pairwise comparisons on the intervention level is available in Supplemental Data 20.

The results for all clinically plausible combinations of components are shown in Supplemental Data 20.

**Fig. 6:**
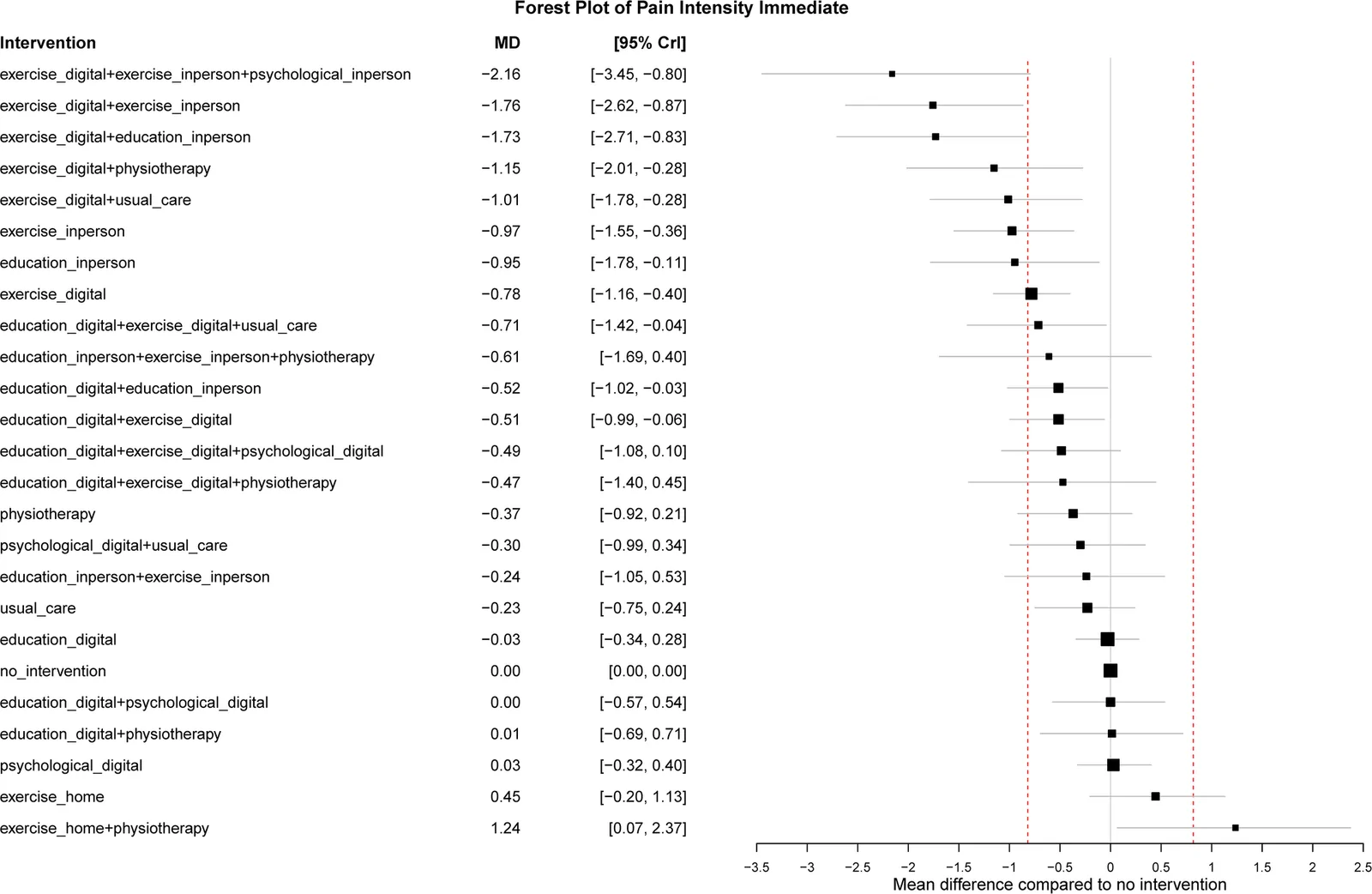
Forest plot of pain intensity immediate. Forest plot of relative effects for the interaction pain intensity immediate network on the intervention level with 95%-credible intervals; red dotted lines represent the MCID of 0.82 for this outcome. MD = mean difference, CrI = credible interval.

For pain intensity immediate, the MCID of 0.5 SD was back transformed using the internal reference SD of the numeric pain rating scale (ranging from 0 to 10), which resulted in an MCID of 0.82 (Fig. 6).

The single-effect NMA model was disconnected and split into three sub-networks (Fig. 2). The additive and the two-way interaction model connected all nodes. The two-way interaction model was used as the primary model because it showed better model fit (lower DIC) compared to the single-effect and additive model (Supplemental Data 18). A comparison of results between the single-effect NMA, the additive and the interaction model is displayed in Supplemental Data 19.

On the component level, no unique component exceeded the MCID compared to no intervention (Supplemental Data 20). The SUCRA ranking with the underlying ranking distribution is displayed in Supplemental Data 20.

On the intervention level, two combinations of components exceeded the MCID compared to no intervention: combined digital and in-person exercise training (MD: −1.76; 95%-CrI: [−2.62; −0.87]) and combined digital exercise training with in-person education (MD: −1.73; 95%-CrI: [−2.71; −0.83]) (Fig. 6, Supplemental Data 20). A table of all pairwise comparisons on the intervention level is available in Supplemental Data 20.

Results for all clinically plausible combinations of components are shown in Supplemental Data 20.

Due to heterogeneous methods of monitoring and reporting adverse events within the included studies, it was not possible to statistically summarize them. No serious adverse events related to the intervention were reported by any of the included studies. Of the 21 studies that reported adverse events, 14 did not define adverse events in the methods of which 10 did report zero adverse events. Contrarily, six of the seven studies that defined adverse events reported at least one adverse event during the study period. The level of reporting was dependent from the unique definition, however most of the reported adverse events were non-serious like temporarily increased pain or psychological discomfort. In the case of non-serious events, it was frequently not reported whether the adverse event was related to the intervention or not. A narrative summary of adverse events in those studies that reported it is available in Supplemental Data 21.

### Exploration for transitivity

For disability immediate, the inspection of boxplots of the effect modifiers baseline pain intensity, baseline disability and age showed some variability between studies for the outcome disability at the immediate time-point (Supplemental Data 22). However, NMR of baseline disability and age with a common interaction term for all active interventions versus no intervention did not show evidence for effect modification (Baseline disability = 0.00; 95%-CrI: [−0.02, 0.02]; Age = 0.0; 95%CrI: [−0.08, 0.11]), which was consistent across delivery pathways (Supplemental Data 22). Due to data paucity, a network meta-regression of baseline pain intensity was not feasible.

Tau-values of frequentist pairwise random-effect meta-analysis show high heterogeneity within contrasts in the following contrast: combined digital education with digital exercise training and usual care vs usual care (tau: 9.18, k = 3) (Supplemental Data 17 and 22). The evaluation of prediction intervals was not feasible due to data paucity.

For pain intensity immediate, the inspection of boxplots of the effect modifiers baseline pain intensity, baseline disability, and age showed some variability between studies for the outcome pain intensity at the immediate time-point (Supplemental Data 22). However, NMR of baseline pain intensity and age with a common interaction term for all active interventions versus no intervention did not show evidence for effect modification (Baseline pain intensity = −0.00; 95%-CrI: [−0.03, 0.02]; Age = 0.01; 95%-CrI: [−0.03, 0.05]), which was consistent across delivery pathways (Supplemental Data 22). Due to data paucity, a network meta-regression of baseline disability was not feasible.

Tau-values of frequentist pairwise random-effect meta-analysis showed low to moderate heterogeneity within contrasts (Supplemental Data 17 and 22). The evaluation of prediction intervals was not feasible due to data paucity.

### Exploration for inconsistency

Since formal methods for evaluating inconsistency in CNMA models have not been developed yet, we used the single-effect NMA model without studies that disconnect the network^17,18^ to evaluate global and local inconsistency.

For disability immediate, the between-study standard deviation, residual deviance, and DIC were similar between the standard NMA and unrelated mean effects model, indicating that the assumption of global consistency holds (Supplemental Data 23). Due to heterogeneity detected in the transitivity assumption, we decided to evaluate local inconsistency using node-splitting. This did not reveal local inconsistency (Supplemental Data 23).

Since model fit parameters indicate a similar heterogeneity between the standard NMA and additive CNMA model, we concluded that assuming additive effects of components in the CNMA model is not likely to be introducing any additional inconsistency (Supplemental Data 18).

As for the outcome disability, we used the standard NMA model without studies that disconnect the network^18–20^ to evaluate global and local inconsistency for pain intensity immediate.

The between-study standard deviation, residual deviance, and DIC were similar between the standard NMA and unrelated mean effects model, indicating that the assumption of global consistency holds (Supplemental Data 23). Due to variability detected in the transitivity assumption, we decided to evaluate local inconsistency using node-splitting. This did not reveal local inconsistency (Supplemental Data 23).

Since model fit parameters indicate a similar heterogeneity between the standard NMA and additive CNMA model, we concluded that assuming additive effects of components in the CNMA model is not likely to be introducing any additional inconsistency (Supplemental Data 18).

### Risk of bias across studies

We were not able to statistically evaluate small study effects with funnel plots and the Egger’s test due to less than 10 studies per contrast in both networks^21^. However, NMR revealed no evidence of influence of the sample size on effect estimates (Disability: 0.00; 95%-CrI: [−0.01, 0.03], pain intensity: 0.00; 95%-CrI: [0, 0.06] (Supplemental Data 24).

The trial registry search identified 32 currently ongoing trials^22–53^, 10 trials without published results^54–63^, one trial with results published in another language^64^ than eligible for this review, five terminated trials^65–69^ and two trials that published results in a different format than eligible for this review^70,71^.

Four of the 43 included studies received private or industry funding which could potentially bias results, and three out of 43 studies did not report a potential conflict of interest.

### Results of additional analyses

For disability immediate, the subgroup analysis that included only chronic pain showed no substantial differences compared to the main analysis on the component level. On the intervention level, combined digital education with digital exercise training and digital psychological intervention (MD: −9.85; 95-CrI: [−14.49; −5.44]) additionally exceeds the MCID. (Supplemental Data 24)

The subgroup analysis with at least one fully digital intervention arm showed no substantial differences compared to the main analysis on the component level. On the intervention level, none of the truly digital interventions exceeds the MCID. (Supplemental Data 24)

Excluding all studies with 10 participants or less in at least one arm as sensitivity analysis showed no substantial differences on the component and intervention level. (Supplemental Data 24) Treating no intervention and waiting list as separate nodes, as well as using 0.40 and 0.81 as correlation coefficients to impute missing change SDs did not substantially change treatment rankings and effect estimates. (Supplemental Data 24) Results were robust to the choice of priors for the between-trial SD (Supplemental Data 24).

All other pre-planned sensitivity and subgroup analyses were not feasible due to disconnected networks in the subgroups.

None of the subgroup and sensitivity analyses conducted for pain intensity (chronic pain, truly digital interventions, excluding small studies) showed substantial differences compared to the main analysis on the component and intervention level (Supplemental Data 24).

Treating no intervention and waiting list as separate nodes, as well as using 0.40 and 0.81 as correlation coefficients to impute missing change SDs did not substantially change treatment rankings and effect estimates. (Supplemental Data 24) Results were robust to the choice of priors for the between-trial SD (Supplemental Data 24).

All other pre-planned sensitivity and subgroup analyses were not feasible due to disconnected networks in the subgroups.

### Certainty assessment

One study in the disability immediate network showed to be sensitive to the level of imprecision, which could lead to a new first-ranked intervention of “in-person exercise training”. All other treatment rankings were robust to individual study-level changes in the evidence (Supplemental Data 25).

Two studies in the pain intensity immediate network showed to be sensitive to the level of imprecision, leading to different possible new first ranked treatment recommendations. For those two studies, the invariant interval falls within the range of effects of the other studies, making it likely that the evidence might change inside this range. One of the studies were rated as high risk of bias due to deviations from the intended interventions and measurement of the outcome, which might have an influence on the study’s estimate. The other study was rated as “some concerns” due to bias in measurement of the outcome and selection in the reported results, which might also have an influence on the study’s estimate. All other treatment rankings were robust to individual study-level changes in the evidence (Supplemental Data 25).

## Discussion

This component network meta-analysis evaluated the comparative efficacy of digital and hybrid interventions for LBP. The findings indicate that although individual components do not demonstrate clinically meaningful effects, certain hybrid combinations of digital and in-person interventions, encompassing education, exercise training, and psychological interventions, showed clinically meaningful improvements on disability at the immediate post-intervention timepoint. For pain intensity, combined digital and in-person exercise, as well as digital exercise with in-person education demonstrated clinically meaningful benefits. No conclusions on long-term outcomes could be drawn due to data paucity. No serious adverse events related to the intervention were reported in any of the studies. However, only few studies reported and pre-defined adverse events, and most studies did not reported a relation of the adverse event with the intervention. Although most studies had a high risk of bias, threshold analysis supported the robustness of our findings.

Our study is the first in the field of digital interventions for low back pain that evaluates the comparative efficacy of treatment components and their combinations. Our findings align with findings in the field of digital interventions for patients with depression. Duarte-Díaz et al.^72^ showed hybrid interventions had a greater effect on reducing depressive symptoms compared to fully digital interventions. It can be hypothesized that the addition of a personal component to a digital treatment could add a small contextual effect like a patient-therapist-relationship, which can account for a small amount of the total effect^73^. Further, qualitative research in the field of digital interventions has shown that patients often express a preference for a personal component in their treatment, which is seen as a facilitator for the usage of these interventions^74^.

Our analysis showed that in-person interventions included in the networks yield similar effects as digital or hybrid interventions. This is supported by findings of meta-analyses in other medical fields like depression^75^, metabolic bariatric surgery^76^, and insomnia^77^. These findings imply that digital components may not necessarily offer additional clinical benefits beyond those of traditional in-person therapy. However, digital components may enhance accessibility and self-management^74^, due to flexible availability compared to in-person delivery and the opportunity for patients to engage with content at their own pace and take a more active role in managing their condition. Nevertheless, it is not clear yet whether digital interventions may be superior in improving important outcomes like self-efficacy, adherence and coping with pain compared to in-person therapy.

The high number of ongoing studies (k = 32) in this field underscores the continued interest and importance of digital interventions. Our threshold analysis suggests that current treatment rankings for the immediate post-intervention timepoint are robust to changes in the evidence in both outcomes. However, it cannot be ruled out completely that the high number of ongoing studies might change the rankings to some extent. It was not possible to conduct a NMA on longer term follow-up timepoints, which underlines the importance of primary trials with a follow-up timespan extending the immediate follow-up timepoint.

Regarding safety, there is currently limited data on adverse events associated with digital and hybrid interventions. It was noticeable that those studies that did not define adverse events a priori were more likely to report zero events compared to those that did provide a definition. Further, most studies did not report a relation between the adverse event and the intervention, which is crucial to rate the safety of adverse events^78^. While no serious adverse events linked to the intervention were reported in any of the included trials, future studies should systematically assess and report adverse events.

This study has several strengths. One of the main advantages is the use of a CNMA approach, which improved precision compared to a single-effect model by connecting several interventions via the underlying components with assumptions on how the combinations interact. For disability, an additive CNMA model was applied, in which the effect of a multicomponent intervention is estimated as the sum of the effects of its individual components. This approach allows estimation of the relative contribution of each component and of combinations of components, thereby supporting interpretation of hybrid treatment strategies. For pain intensity outcomes, an interaction model could additionally be applied, representing a further methodological strength, as it allows for the possibility that treatment components may interact synergistically or antagonistically rather than contributing purely additively. Further, CNMA allowed us to estimate the effects of individual treatment components and their various combinations, even in cases where certain combinations had not yet been directly tested in clinical trials. This can support the suggestion of interventions to be tested in clinical trials. This unique approach fills a gap in the current evidence base for digital interventions for LBP and has notable potential to guide clinical practice. By providing insights into how specific components and their combinations may work, this study can provide valuable information to healthcare professionals and decision makers. In addition, the use of threshold analysis further strengthens the robustness of our effect estimates, adding confidence in the reliability of our conclusions and confirming that findings were unlikely to be due to high risk of bias observed in any single included trial. It has to be noted though, that a contrast-level threshold analysis for CNMA has not been developed yet, but could give more insights on the robustness besides possible bias on the study-level. In addition, the comprehensive and systematic search strategy we used including a search validation increased the rigour of our study and ensured that our analysis was based on the full body of evidence available.

Although this network meta-analysis was thoroughly planned and implemented, it is important to consider its limitations. The inclusion of both sub-acute and chronic patient populations may introduce heterogeneity in treatment responses since chronic pain patients with a long pain duration might show lower treatment responses compared to subacute patients^79^. However, subgroup analyses revealed that no evidence for a difference between treatment effects for sub-acute and chronic patients was present. Given the limited number of studies including sub-acute patients, this finding should be interpreted cautiously and does not confirm equivalence between sub-acute and chronic populations, but rather suggests no clear evidence that their combination substantially influenced the estimated treatment effects. In addition, acute low back pain populations were not included, which may limit the generalizability of our findings to this group. Regarding the interpretation of clinical relevance, we applied a distribution-based MCID corresponding to one-half of the pooled baseline standard deviation, as proposed by Watt et al.^80^. This approach has been recommended for systematic reviews synthesizing continuous outcomes measured with different instruments, particularly when a single instrument-specific MCID cannot be applied consistently. The resulting thresholds (0.82/10 for pain intensity and 4.92/100 for disability) are consistent with commonly reported MCIDs for these outcomes^81,82^ and are only slightly lower than frequently cited instrument-specific thresholds, indicating that our interpretation of clinical relevance was not overly conservative. Although the search was restricted to English and German publications, empirical evidence suggests that excluding non-English studies rarely changes the conclusions of systematic reviews^83–85^. Nevertheless, the potential for language bias cannot be entirely excluded.

On the level of the evidence included in the review, we found heterogeneity in baseline values across studies identified in the transitivity assessment. However, NMR showed no evidence of effect modification. Additionally, long-term efficacy remains unclear since follow-up analyses were not possible, highlighting a gap in the literature. Some treatment effects were mainly derived from indirect comparisons, which may increase uncertainty. Nonetheless, CNMA can partially account for this by integrating information across common intervention components. Further, the primary analysis for disability relied on an additive model, which assumes that the effects of individual treatment components combine additively when delivered together. In clinical practice, however, treatment components such as exercise, education, and psychological interventions may interact or produce synergistic effects. Although interaction models were explored, reliable estimation of interaction terms requires substantially larger and more densely connected networks. Given the limited number of studies evaluating specific component combinations, interaction effects could not be estimated reliably and should be explored in future research.

It may be valuable for further research to investigate whether the combination of digital and in-person interventions influences other important outcomes, such as self-efficacy, coping strategies, and treatment adherence. Understanding these aspects is crucial to determine the broader role of digitally delivered interventions compared to in-person delivered interventions. Furthermore, combinations of components that appear clinically meaningful—especially those that are plausible, but have not yet been studied in trials (e.g., combined digital and in-person exercise training with physiotherapy, combined digital exercise training with in-person psychological intervention and physiotherapy, combined digital and in-person exercise training with usual care, or combined digital psychological intervention with in-person exercise training and physiotherapy) could be evaluated in high-quality randomized controlled trials. Future research might also investigate potential interaction effects between treatment components, e.g., in a factorial design, as multicomponent interventions may produce synergistic effects that cannot be fully captured under the additive CNMA assumption. In addition, future clinical trials could include a common systematic approach to defining, monitoring and reporting adverse events and the relation to the intervention under investigation to assess the safety of digital interventions.

Our results indicate that digital and hybrid interventions can be used in clinical practice for patients with LBP, as their effects compared with no intervention were no different to those of in-person interventions. A hybrid approach may offer additional benefit over fully digital interventions by enabling some degree of clinician interaction, which could enhance engagement and lead to greater improvements in disability. Furthermore, our results support the use of multicomponent interventions, particularly those combining exercise training and psychological components, which were associated with the largest effects. These insights highlight the potential for flexible, scalable treatment models, but also underscore the importance of aligning intervention strategies with patient needs, preferences, and resource availability in clinical settings. While costs were not evaluated in this study, existing evidence suggests that digital interventions may offer efficiency gains, although cost-effectiveness likely varies across healthcare systems and implementation contexts^86,87^.

In conclusion, whilst interventions comprised of just a single component may not be efficacious, multicomponent hybrid interventions have the potential to yield clinically significant improvements in disability and pain intensity for individuals with low back pain. In contrast, fully digital interventions do not appear to demonstrate comparable efficacy. The findings were robust to risk of bias in the individual studies. Digital and hybrid interventions appear to be safe, however, heterogeneous reporting of adverse events does not allow for definitive conclusions. Future research could benefit from systematically assessing adverse events and investigating how hybrid approaches may influence key factors such as treatment adherence, patient engagement, and self-efficacy. Additionally, future studies need to consider the long-term sustainability of hybrid interventions and their feasibility in routine clinical practice.

## Methods

This review was conducted and reported in accordance with the Cochrane Handbook for Systematic Reviews of Interventions^88^ and the PRISMA Extension Statement for Reporting of Systematic Reviews Incorporating Network Meta-analyses (Supplemental Data 1)^89^. Further, we included all information required as per AMSTAR 2 (Supplemental Data 2) to ensure a high methodological quality^90^.

### Protocol and registration

The protocol was prospectively registered on the Open Science Framework (https://doi.org/10.17605/OSF.IO/4UVKR)^91^. Amendments to the protocol since registration are listed in Supplemental Data 3.

### Patient and public involvement

Patients and members of the public were not involved in this study.

### Intervention definition

Interventions were defined as combinations of treatment components and delivery modes. Treatment components included education, psychological, and exercise interventions, as well as comparator components (usual care, physiotherapy, placebo, no intervention, and waiting list). Delivery was categorised as digital or in-person. Interventions could consist of a single component or a combination of components and could be delivered digitally, in-person, or a combination of these. Comparator components were not linked to a delivery mode. No restrictions were placed on intervention duration, frequency, or setting.

### Eligibility criteria

All inclusion criteria followed the Participants, Interventions, Comparators, Outcomes and Study design (PICOS) framework^92^. A list of exclusion criteria can be found in Supplemental Data 4.

We included studies on adults (≥ 18 years) with non-specific sub-acute or chronic low back pain, defined as pain between the costal margin and gluteal folds, with or without leg pain^93^. Following Bardin et al.^94^, non-specific low back pain excludes specific spinal pathologies (e.g., fractures, malignancies, infections, spondyloarthritis) and radicular syndromes. Subacute pain was defined as lasting >6 but <12 weeks^95^, while chronic pain persisted for ≥12 weeks^96^. Studies were included if they explicitly used these terms or required a minimum pain duration of 6 weeks. If some participants had pain <6 weeks, inclusion was allowed if the mean duration across all patients was ≥6 weeks and the lower bound of the 95% CI did not exceed four weeks. For studies reporting pain duration on an ordinal scale, inclusion required ≥50% of participants experiencing pain for at least 6 weeks.

Eligible studies assessed digital synchronous (real-time contact with therapist) and asynchronous delivery of interventions (no real-time contact or self-guided) for LBP delivered via app, website, online platform, or videoconferencing. Telephone-based interventions were included if they provided interactive treatment (e.g., psychological therapy), but not if used solely for reminders or follow-up prompts. Hybrid interventions combining digital and in-person therapy were included, while no threshold regarding the amount of each component was applied (e.g., the intervention was included even if the digital part was only 20%). The content of the digital intervention could be one or a combination of the following: education, exercise training, or psychological interventions. In the analysis, we distinguished between components and interventions. A component was defined as a distinct part of therapy that is considered to impact the therapeutic efficacy of that therapy (e.g., digital exercise training). An intervention was defined as a therapy, that consists of ≥1 component(s) that, in combination, impact its therapeutic efficacy (e.g., digital exercise training + in-person psychological intervention), and that could feasibly be delivered as a therapy to a patient. A definition of each component is available in Supplemental Data 5.

Any of the components listed above, either digitally or in-person as well as physiotherapy (labelled as such by the trial authors and not standardized, delivered in-person), usual care (labelled as such by the trial authors and not standardized), no intervention, and waiting list. No intervention and waiting list have been considered as the same node in the network.

The main outcomes were disability and pain intensity. Adverse events and adherence rates were extracted from included studies but were not an inclusion criterion.

We included parallel-group, cluster, and cross-over RCTs as they are the gold standard for assessing intervention efficacy. Only peer-reviewed studies in English or German were included, excluding grey literature. While non-English studies may narrow confidence intervals, these likely do not affect effect estimates^97^. Therefore, we pragmatically chose to include articles in languages in which the author-team is fluent. Multiple publications from the same RCT (e.g., follow-ups) were included and their data were combined for analysis.

### Information sources

Searches were conducted in PubMed, SPORTDiscus, CENTRAL, EMBASE, PsycINFO, and CINAHL from inception till 11 April 2024 and updated on 20 May 2025. Additionally, we manually screened reference lists of included studies and relevant systematic reviews (Supplemental Data 6) and performed forward citation tracking with all included records of studies. Duplicates were removed automatically using Covidence (http://www.covidence.org). The MEDLINE search strategy was validated for sensitivity and precision using 17 reference studies from a previous umbrella review^7^, using a search strategy validation approach^98^. We also searched the WHO and NIH trial registries.

### Search

We used a combination of MeSH and free-text terms to identify relevant RCTs. The full search strategy is available in Supplemental Data 7.

### Study selection

Two independent reviewers (SK, NKA) screened titles/abstracts and full texts using Covidence (http://www.covidence.org), resolving conflicts through discussion or adjudication with a third reviewer (NLM) if needed. Before screening, the method was piloted by the two reviewers (SK, NKA) on 70 title/abstract and 10 full-text studies. To ensure methodological quality, a third reviewer (RD) randomly cross-checked 10% of the included studies at each screening stage, which revealed no issues with screening.

### Data collection process

Two independent reviewers (SK, TiS) extracted data using a custom-developed Google Sheet, resolving conflicts through discussion or adjudication with a third reviewer (NLM) if needed. Before extraction, the method was piloted by the two reviewers (SK, TiS) on five randomly selected papers. To ensure methodological quality, a third reviewer (TS) randomly cross-checked 10% of the extracted data, which revealed no issues with extraction.

### Data items

We extracted the following data from the included studies: publication information (author, title, year), study design, the number of participants randomized per group, and participant characteristics such as age, sex, and pain duration. Outcome measures, including disability and pain intensity, were also extracted, with the hierarchy of outcome assessment instruments detailed in Supplemental Data 8. Means and standard deviations were collected for pre- and post-intervention assessments and synthesized as mean change from baseline. If change from baseline SDs were missing, they were imputed with a correlation of ρ = 0.59, based on an empirical analysis of pre- and post-treatment continuous outcome trials^99^. Intervention characteristics were documented, including the delivery pathway (i.e., app, website, telephone, online platform, video or videoconferencing) and the delivery mode (i.e., synchronous, asynchronous, or combination). Adverse events, both serious and non-serious, dropout rates (i.e., the number of participants assigned to the intervention vs. the number who completed the intervention regardless of the follow-up assessment), funding sources (public funding, private/industry funding, no funding), and conflicts of interest were also extracted. Adverse events were classified as serious or non-serious by reviewers. Serious events included those causing death, life-threatening conditions, hospitalization, or significant disability^78^. Non-serious events encompassed all other unpredicted conditions during the trial^78^. If available, causality and intervention discontinuation due to adverse events were noted. Reports of adverse events that were not described further by trial authors, were classified as “unclear adverse event”.

Where available, data were extracted for the following time points: immediate post-intervention, short-term ( > 1 week to <3 months), intermediate-term ( ≥ 3 to <12 months), and long-term follow-up ( ≥ 12 months). If a study reported multiple time points within one timespan, the longest follow-up was chosen for extraction. Data collected during the intervention (e.g., at week 3 of a 6-weeks intervention) were not extracted. In cases where pain intensity was assessed across different conditions (e.g., pain at night, at rest, or during the day), pain at rest was chosen as the primary outcome measure.

When data were presented as medians or other measures of spread, these were converted to mean and standard deviation using the methods outlined by Higgins et al.^100^. If only graphical representations were provided, data were extracted using ImageJ (https://imagej.nih.gov/ij/)^101^. If the required data could not be extracted, the authors were contacted at least three times over a 4-week period. If no response was received, the standard deviation was imputed using data from similar studies following established methods^102^. The complete data handling is described in Supplemental Data 9.

### Geometry of the network

We explored network connectivity with network plots. To visualize the distribution of components across studies within the network, we used UpSet plots showing which combinations of components were present in how many studies and how often each component was used^103^.

### Risk of bias within individual studies

We used the Version 2 of the Cochrane Risk of Bias Tool to examine the risk of bias in the included studies^104^. It was applied to the outcome pain intensity at the earliest available post-intervention follow-up timepoint for the intention-to-treat analysis. When pain intensity was not reported, it was applied to the outcome disability. Risk of bias ratings were conducted by two independent reviewers (SK, TiS) and conflicts resolved by an adjudicator (NLM). The guidance sheet is available in Supplemental Data 10.

### Summary measures

Since outcomes were continuous or ordinal, but measured on different scales, mean differences were standardized using an internal reference SD (i.e., the mean of the pooled SD of each scale)^105^ and back-transformed to the most frequently used scale (Supplemental Data 9). If multiple groups in a study were classified as the same intervention, their data were pooled (Supplemental Data 9)^106^. Reverse-scaled outcomes (higher values indicating better outcomes) were multiplied by -1, following Cochrane Handbook recommendations^106^.

Multiple reports from the same study (e.g., follow-ups, secondary analyses) were cross-checked for originating from the same study by two extractors (SK, TiS). Data from the main report were included, along with additional timepoints if available.

Adverse events per treatment (or combination) were summarized narratively. Where possible, the number of serious and non-serious adverse events were reported separately.

### Planned methods of analysis

All analyses have been conducted in the R environment (v 4.4.2)^107^ using RStudio. Bayesian random-effects component network meta-analysis (CNMA) was performed per outcome and time-point in accordance with current PRISMA network meta-analysis guidelines^89^ via R2jags (v 0.8-9)^108^. JAGS code for the implemented models is provided on the OSF (https://osf.io/ha834/?view_only=a9b65c1102774be8a7e5d9cea8a30765).

**Fig. 7:**
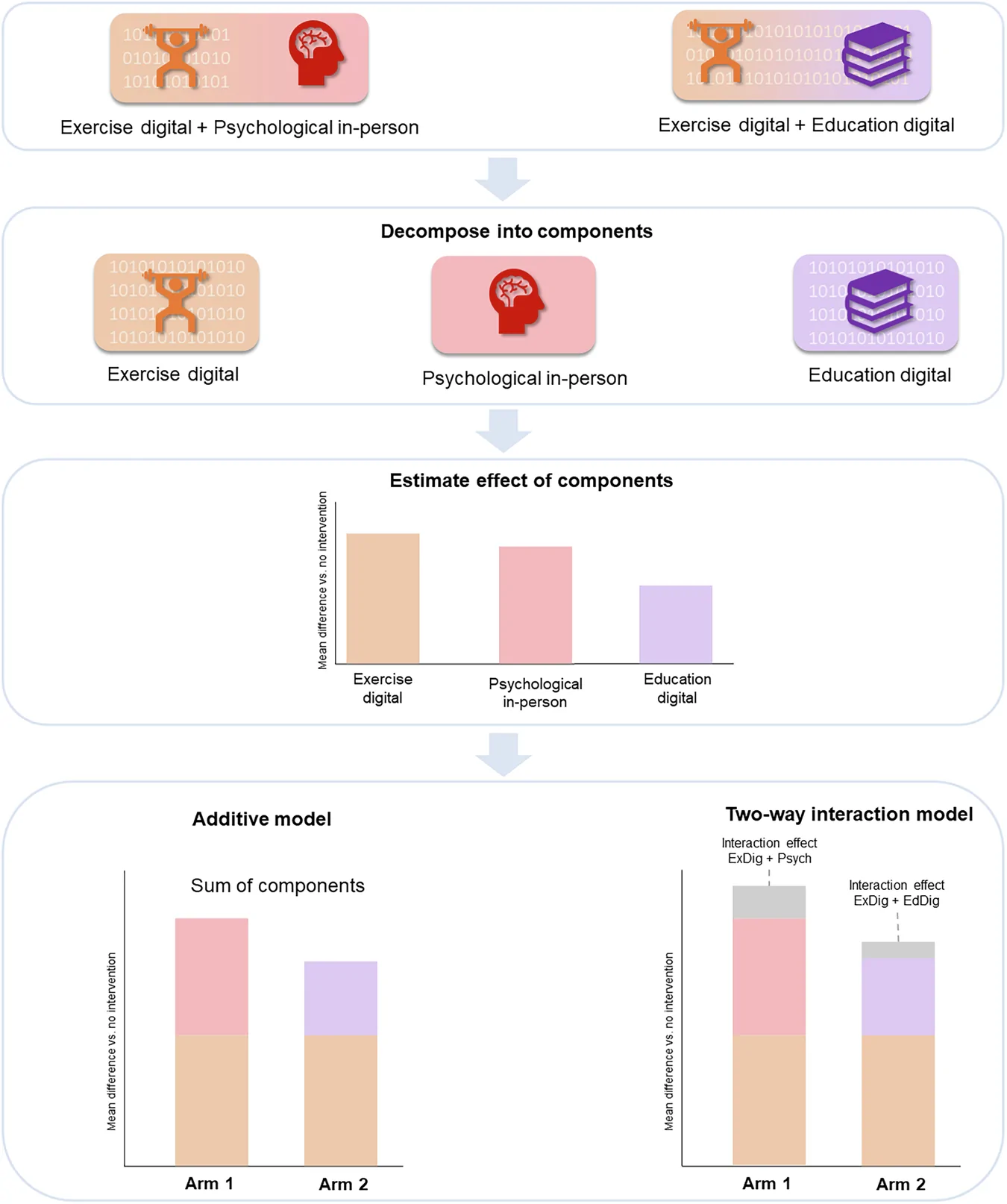
Conceptual overview of component network meta-analysis (CNMA). Illustrative example of how interventions in a two-arm study can be represented as combinations of treatment components (e.g., exercise, education, psychological) and delivery modes (digital or in-person). CNMA decomposes these interventions into their individual components and estimates the contribution of each component to treatment effects. In an additive model, the effect of a combined intervention is assumed to reflect the sum of its components, whereas interaction models additionally allow for effects arising from specific component combinations. The example shown is for illustration only; in practice, component effects are synthesised across multiple studies and treatment comparisons within the network.

Interventions were defined and decomposed into their constituent treatment components (e.g., an app-based exercise training intervention with in-person education was classified as digital exercise training + in-person education), which subsequently formed the basis for network construction and the component network meta-analysis (Fig. 7). A complete list of components is provided in Supplemental Data 11.

A *single-effect network meta-analysis* (NMA) on the intervention level was first conducted, treating each intervention as a distinct node. This approach is mathematically equivalent to the maximally estimable interaction model^109^. Subsequently, an *additive component NMA* (CNMA) was implemented to estimate treatment effects based on individual components^12^. In this model, the effect of a combined intervention is modelled as the sum of the effects of its components (additive assumption). This means that each component is assumed to have a similar contribution whether it is delivered alone or together with other components. As a third step, a *two-way interaction model* was used to assess potential interactions between components^12^. Model fit was evaluated using the Deviance Information Criterion (DIC)^110^, with differences of 3 points considered meaningful.

The treatment effects and study baselines were assigned vague normal priors with a mean of 0 and a variance of 10,000. The between-trial standard deviation was given a uniform prior between 0 and 5^39^. A sensitivity analysis explored the influence of this prior by implementing an informed prior based on Rhodes et al.^111^. Specifically, a weakly informative Student-t prior (ν = 5, μ = −3.02, σ = 2.27) was placed on log τ^2^.

The minimal clinically important difference (MCID) for each outcome was estimated using the distribution-based approach proposed by Watt et al.^80^, defined as one-half of the pooled baseline standard deviation across all included studies. The resulting MCID was subsequently back-transformed to the most commonly used measurement scale for each outcome to facilitate clinical interpretation. Effect estimates were considered clinically meaningful when the entire credible interval exceeded the MCID.

Comparative efficacy was assessed using the surface under the cumulative ranking curve (SUCRA), which ranks interventions based on relative effects. The primary analysis estimated relative effects and SUCRA rankings for components and component combinations (interventions) observed in the dataset. Additionally, to explore the efficacy of untested combinations, we identified the top ten clinically plausible combinations. Clinically plausible combinations were defined as combination that included at least one digital component combined with any other component except for no intervention. We thus estimated effects at three levels: firstly, on the component level; secondly, on the level of intervention; and finally, on the level of clinically plausible interventions.

### Assessment of inconsistency

To assess clinical similarity among the included studies, transitivity was evaluated by qualitatively comparing the distribution of potential effect modifiers across direct comparisons^112,113^. Suspected effect modifying patient characteristics in LBP include age, baseline pain intensity, disability, and pain duration^114,115^.

Transitivity and homogeneity were additionally assessed through forest plots, statistical heterogeneity (I^2^, Tau), and 95% prediction intervals from frequentist pairwise random-effects meta-analyses per control intervention. Between-study variance (Tau^2^) was estimated using a restricted maximum likelihood estimator for continuous outcomes, a Paule-Mandel estimator for dichotomous outcomes^116^, and a Knapp-Hartung adjustment^117^ for confidence intervals of summary effects.

Consistency between direct and indirect treatment comparisons was assessed in single-effect NMA models using an unrelated mean effects (UME) model^118^. Consistency was assumed if the DIC and the between-study SD of the NMA and UME models were similar. To evaluate local inconsistency, we compared the residual deviance contributions from each of the models using a dev-dev plot. Additionally, the node-splitting approach was applied^119^. If inconsistency remained, potential sources were explored, and analyses were adapted through subgroup analyses or by excluding studies contributing to heterogeneity. The consistency assessment was conducted using the package *multinma* (v. 0.7.2)^120^.

While methods for assessing inconsistency in CNMA models are not established, inconsistency in NMA models implies potential inconsistency in CNMA models, given that CNMA models make additional assumptions and include fewer parameters^121^. However, it should be noted that the absence of inconsistency in single-effect NMA models does not rule out inconsistency in CNMA models. However, similar model fit and between-study SD between CNMA, single-effect NMA, and UME models are supportive of overall consistency^121^.

### Additional analyses

Subgroup analyses or network meta-regression (NMR) for pain duration, baseline pain intensity and disability and age were conducted if data allowed. NMR was explored assuming a common interaction term for all interventions versus the reference intervention (no treatment)^122^. If the common NMR model was suggestive of the presence of effect modification (evaluated by comparing DIC and between-study SD), a more complex NMR model was explored that assumed common interaction terms for digital, hybrid and in-person interventions separately versus no treatment^122^. Additional subgroup analyses examined fully digital treatments, combined digital and in-person delivery, and varying intervention durations. Sensitivity analyses were performed by excluding high RoB studies, studies with imputed missing mean and/or SD, studies with fewer than ten participants in at least one arm, control groups “waiting list” and “no intervention” as separate nodes, and different correlation coefficients for imputing SDs of change.

### Risk of bias across studies

A comprehensive search was conducted to minimize the risk of bias due to missing results, including searches in trial registries to identify unpublished studies. Funding sources and potential conflicts of interest were also assessed.

Contour-enhanced funnel plots were visually inspected for each pairwise comparison that had 10 studies and more, and Egger’s test was used to statistically evaluate publication and small study bias^123^. Additionally, NMR of the CNMA model was performed to explore potential small-study effects by assessing associations between sample size and effect size with sample size as continuous covariate^124^.

### Certainty assessment

To assess the certainty of treatment recommendations, we used an approach recommended for complex networks by the National Institute for Health and Care Excellence (NICE)^125^. A threshold analysis based on invariant intervals^126^ was conducted using the R package *nmathresh* (v0.1.6)^127^. This approach evaluates the sensitivity of intervention rankings to changes in the evidence and is recommended for NMA instead of GRADE, as it accounts for the influence of bias on final intervention rankings, which is not feasible in GRADE^126^. Threshold analysis can estimate how much the evidence per contrast or study would need to change before the overall intervention ranking changes^126^. Since contrast-based threshold analysis is only established for single-effect NMA models, we applied a study-based threshold analysis. If the confidence intervals of studies extended beyond the invariant interval limits, the recommendation was considered sensitive to the level of uncertainty in the data^125,126^. Clinical conclusions were drawn based on the magnitude of the invariant interval compared to clinically plausible effects, as well as on potential sources of sensitivity such as risk of bias within the specific study^125^.

## Supplementary information

Supplementary Information

## Data Availability

Data and analysis code are openly available on the Open Science Framework (https:/osf.io/ha834/?view_only=a9b65c1102774be8a7e5d9cea8a30765))

## Code availability

Code is openly available on the Open Science Framework (https://osf.io/ha834/?view_only=a9b65c1102774be8a7e5d9cea8a30765).
