# Supplementary material for: Digital and hybrid interventions for subacute and chronic low back pain: component network meta-analysis
Source: NPJ Digit Med. 2026 Nov 4;9:851. doi: 10.1038/s41746-026-03416-5 (PMC13736906; doi:10.1038/s41746-026-03416-5)
Supplement: Supplementary file 1 [file 41746_2026_3416_MOESM1_ESM.pdf]

Content

Supplemental Data 1 – PRISMA NMA checklist....5
Supplemental Data 2 – AMSTAR 2 checklist....9
Supplemental Data 3 – Amendments to the protocol since registration....14
Supplemental Data 4 – Exclusion criteria....15
Supplemental Data 5 – Definition of intervention components....17
Supplemental Data 6 – Manually searched systematic reviews....18
Supplemental Data 7 – Search strategy....20
Supplemental Data 8 – Hierarchy of outcome assessments....23
Supplemental Data 9 – Data handling....24
Supplemental Data 10 – RoB guidance sheet....26
Domain 1: Risk of bias arising from the randomization process....26
Domain 3: Risk of bias due to missing outcome data....35
Domain 4: Risk of bias in measurement of the outcome....38
Domain 5: Risk of bias in selection of the reported result....42
Supplemental Data 11 – List of components....47
Supplemental Data 12 – Excluded studies at full text screening....48
Supplemental Data 13 – Authors contacted for further information....59
Supplemental Data 14 – Network plots of follow-up timepoints....63
Disability....63
>1 week < 3 months....63
> 3 months < 1 year....64
Pain intensity....65
>1 week < 3 months....65
> 3 months < 1 year....66
Supplemental Data 15 – Studies per node....67
Disability immediate....67
Pain intensity immediate....68
Supplemental Data 16 – Risk of bias within studies....69
Traffic plot parallel trials....69
Traffic plot cluster trials....70
Risk of bias individual ratings....71
Supplemental Data 17 – Pairwise meta-analyses....72

Disability immediate....73
Network 1....73
Network 2....74
Network 3....74
Disability >1 week <3 months....75
Network 1....75
Network 2....75
Network 3....76
Disability >3months <1 year....77
Network 1....77
Network 2....77
Network 3....78
Pain intensity immediate....79
Network 1....79
Network 2....80
Network 3....80
Pain intensity >1 week <3months....81
Network 1....81
Network 2....81
Network 3....81
Network 4....82
Pain intensity >3 months <1 year....83
Network 1....83
Network 2....83
Network 3....84
Supplemental Data 18 – Model fit....85
Disability immediate....85
Pain intensity immediate....85
Supplemental Data 19 – Forest plot of single-effect, additive and interaction model....86
Disability immediate....86
Pain intensity immediate....87
Supplemental Data 20 – Network meta-analysis results....88
Disability immediate....88
Component level....88

Intervention level....89
All clinically plausible combinations of components....91
Pain intensity immediate....97
Component level....97
Intervention level....99
All clinically plausible combinations of components....101
Supplemental Data 21 – Narrative summary of adverse events....107
Supplemental Data 22 – Transitivity assessment....110
Disability immediate....110
Boxplot of baseline pain intensity by treatment....110
Boxplot of baseline pain intensity by treatment comparison....111
Boxplot of baseline disability by treatment....112
Boxplot of baseline disability by treatment comparison....113
Boxplot of mean age by treatment....114
Boxplot of mean age by treatment comparison....115
Network meta-regression....116
Tau-plot of random-effect pairwise meta-analyses....117
Pain intensity immediate....118
Boxplot of baseline pain intensity by treatment....118
Boxplot of baseline pain intensity by treatment comparison....119
Boxplot of baseline disability by treatment....120
Boxplot of baseline disability by treatment comparison....121
Boxplot of mean age by treatment....122
Boxplot of mean age by treatment comparison....123
Network meta-regression....124
Tau plot of random effect pairwise meta-analyses....125
Supplemental Data 23 – Inconsistency assessment....126
Disability immediate....126
Comparison between UME and standard NMA model....126
Local inconsistency - nodesplitting....128
Pain intensity immediate....131
Comparison between UME and standard NMA model....131
Local inconsistency - nodesplitting....133
Supplemental Data 24 – Results of additional analyses....138

Disability immediate....138
Subgroup analysis: chronic pain patients....138
Subgroup analysis: Fully digital interventions....142
Sensitivity analysis: Excluding studies with less than 10 participants in one arm....146
Network meta-regression sample size....150
Sensitivity analysis: No intervention and waiting list as separate nodes....151
Sensitivity Analysis: Correlation coefficient for imputing missing SDs – 0.40....154
Sensitivity Analysis: Correlation coefficient for imputing missing SDs – 0.81....155
Sensitivity Analysis: Informative prior for between-trial standard deviation....156
Pain intensity immediate....157
Subgroup analysis: chronic pain patients....157
Subgroup analysis: Fully digital interventions....161
Sensitivity analysis: Excluding studies with less than 10 participants in one arm....165
Network meta-regression sample size....169
Sensitivity analysis: No intervention and waiting list as separate nodes....170
Sensitivity Analysis: Correlation coefficient for imputing missing SDs – 0.40....171
Sensitivity Analysis: Correlation coefficient for imputing missing SDs – 0.81....172
Sensitivity Analysis: Informative prior for between-trial standard deviation....173
Supplemental Data 25 – Results of threshold analyses....174
Disability immediate....174
Pain intensity immediate....175

Supplemental Data 1 – PRISMA NMA checklist

Supplemental Table 1 – PRISMA NMA checklist

Section/Topic	Item #	Checklist Item	Reported on Page #
**TITLE**			
Title	1	Identify the report as a systematic review *incorporating a network meta-analysis (or related form of meta-analysis).*	***1***
**ABSTRACT**			
Structured summary	2	Provide a structured summary including, as applicable:<br>**Background:** main objectives<br>**Methods:** data sources; study eligibility criteria, participants, and interventions; study appraisal; and *synthesis methods, such as network meta-analysis.*<br>**Results:** number of studies and participants identified; summary estimates with corresponding confidence/credible intervals; *treatment rankings may also be discussed. Authors may choose to summarize pairwise comparisons against a chosen treatment included in their analyses for brevity.*<br>**Discussion/Conclusions:** limitations; conclusions and implications of findings.<br>**Other:** primary source of funding; systematic review registration number with registry name.	5
**INTRODUCTION**			
Rationale	3	Describe the rationale for the review in the context of what is already known*, including mention of why a network meta-analysis has been conducted.*	***6***
Objectives	4	Provide an explicit statement of questions being addressed, with reference to participants, interventions, comparisons, outcomes, and study design (PICOS).	6-7
**METHODS**			
Protocol and registration	5	Indicate whether a review protocol exists and if and where it can be accessed (e.g., Web address); and, if available, provide registration information, including registration number.	8
Eligibility criteria	6	Specify study characteristics (e.g., PICOS, length of follow-up) and report characteristics (e.g., years considered, language, publication status) used as criteria for eligibility, giving rationale. *Clearly describe eligible treatments included in the treatment network, and note whether any have been clustered or merged into the same node (with justification).*	*8-9*

Information sources	7	Describe all information sources (e.g., databases with dates of coverage, contact with study authors to identify additional studies) in the search and date last searched.	10
Search	8	Present full electronic search strategy for at least one database, including any limits used, such that it could be repeated.	10
Study selection	9	State the process for selecting studies (i.e., screening, eligibility, included in systematic review, and, if applicable, included in the meta-analysis).	10
Data collection process	10	Describe method of data extraction from reports (e.g., piloted forms, independently, in duplicate) and any processes for obtaining and confirming data from investigators.	10
Data items	11	List and define all variables for which data were sought (e.g., PICOS, funding sources) and any assumptions and simplifications made.	10-11
**Geometry of the network**	**S1**	Describe methods used to explore the geometry of the treatment network under study and potential biases related to it. This should include how the evidence base has been graphically summarized for presentation, and what characteristics were compiled and used to describe the evidence base to readers.	*12*
Risk of bias within individual studies	12	Describe methods used for assessing risk of bias of individual studies (including specification of whether this was done at the study or outcome level), and how this information is to be used in any data synthesis.	12
Summary measures	13	State the principal summary measures (e.g., risk ratio, difference in means). *Also describe the use of additional summary measures assessed, such as treatment rankings and surface under the cumulative ranking curve (SUCRA) values, as well as modified approaches used to present summary findings from meta-analyses.*	12
Planned methods of analysis	14	Describe the methods of handling data and combining results of studies for each network meta-analysis. This should include, but not be limited to:<br>• *Handling of multi-arm trials;*<br>• *Selection of variance structure;*<br>• *Selection of prior distributions in Bayesian analyses; and*<br>• *Assessment of model fit.*	12-13
**Assessment of Inconsistency**	**S2**	Describe the statistical methods used to evaluate the agreement of direct and indirect evidence in the treatment network(s) studied. Describe efforts taken to address its presence when found.	13-14
Risk of bias across studies	15	Specify any assessment of risk of bias that may affect the cumulative evidence (e.g., publication bias, selective reporting within studies).	15
Additional analyses	16	Describe methods of additional analyses if done, indicating which were pre-specified. This may include, but not be limited to, the following:<br>• Sensitivity or subgroup analyses;<br>• Meta-regression analyses;<br>• *Alternative formulations of the treatment network;*	*14-15*

		and • *Use of alternative prior distributions for Bayesian analyses (if applicable).*	

RESULTS†

Study selection	17	Give numbers of studies screened, assessed for eligibility, and included in the review, with reasons for exclusions at each stage, ideally with a flow diagram.	16
**Presentation of network structure**	**S3**	Provide a network graph of the included studies to enable visualization of the geometry of the treatment network.	***16***
**Summary of network geometry**	**S4**	Provide a brief overview of characteristics of the treatment network. This may include commentary on the abundance of trials and randomized patients for the different interventions and pairwise comparisons in the network, gaps of evidence in the treatment network, and potential biases reflected by the network structure.	***16-17***
Study characteristics	18	For each study, present characteristics for which data were extracted (e.g., study size, PICOS, follow-up period) and provide the citations.	17, Table 1
Risk of bias within studies	19	Present data on risk of bias of each study and, if available, any outcome level assessment.	17
Results of individual studies	20	For all outcomes considered (benefits or harms), present, for each study: 1) simple summary data for each intervention group, and 2) effect estimates and confidence intervals. *Modified approaches may be needed to deal with information from larger networks.*	***17***
Synthesis of results	21	Present results of each meta-analysis done, including confidence/credible intervals. *In larger networks, authors may focus on comparisons versus a particular comparator (e.g. placebo or standard care), with full findings presented in an appendix. League tables and forest plots may be considered to summarize pairwise comparisons.* If additional summary measures were explored (such as treatment rankings), these should also be presented.	***18-19***
**Exploration for inconsistency**	**S5**	Describe results from investigations of inconsistency. This may include such information as measures of model fit to compare consistency and inconsistency models, *P* values from statistical tests, or summary of inconsistency estimates from different parts of the treatment network.	***19-21***
Risk of bias across studies	22	Present results of any assessment of risk of bias across studies for the evidence base being studied.	21
Results of additional analyses	23	Give results of additional analyses, if done (e.g., sensitivity or subgroup analyses, meta-regression analyses*, alternative network geometries studied, alternative choice of prior distributions for Bayesian analyses,* and so forth).	***21-22***

Section/Topic	Item #	Checklist Item	Reported on Page #
**DISCUSSION**			
Summary of evidence	24	Summarize the main findings, including the strength of evidence for each main outcome; consider their relevance to key groups (e.g., healthcare providers, users, and policy-makers).	22-23
Limitations	25	Discuss limitations at study and outcome level (e.g., risk of bias), and at review level (e.g., incomplete retrieval of identified research, reporting bias). *Comment on the validity of the assumptions, such as transitivity and consistency. Comment on any concerns regarding network geometry (e.g., avoidance of certain comparisons).*	24
Conclusions	26	Provide a general interpretation of the results in the context of other evidence, and implications for future research.	26
**FUNDING**			
Funding	27	Describe sources of funding for the systematic review and other support (e.g., supply of data); role of funders for the systematic review. This should also include information regarding whether funding has been received from manufacturers of treatments in the network and/or whether some of the authors are content experts with professional conflicts of interest that could affect use of treatments in the network.	***27***

PICOS = population, intervention, comparators, outcomes, study design.
* Text in italics indicateS wording specific to reporting of network meta-analyses that has been added to guidance from the PRISMA statement.
† Authors may wish to plan for use of appendices to present all relevant information in full detail for items in this section.

Supplemental Data 2 – AMSTAR 2 checklist

Supplemental Table 2 – AMSTAR 2 checklist

1. Did the research questions and inclusion criteria for the review include the components of PICO?

For Yes:<br>x Population<br>x Intervention<br>x Comparator group<br>x Outcome	Optional (recommended)<br>x Timeframe for follow-up	x Yes<br>☐ No

2. Did the report of the review contain an explicit statement that the review methods were established prior to the conduct of the review and did the report justify any significant deviations from the protocol?

For Partial Yes:<br>The authors state that they had a written protocol or guide that included ALL the following:<br>x review question(s)<br>x a search strategy<br>x inclusion/exclusion criteria<br>x a risk of bias assessment	For Yes:<br>As for partial yes, plus the protocol should be registered and should also have specified:<br>x a meta-analysis/synthesis plan, if appropriate, *and*<br>x a plan for investigating causes of heterogeneity<br>x justification for any deviations from the protocol	x Yes<br>☐ Partial Yes<br>☐ No

3. Did the review authors explain their selection of the study designs for inclusion in the review?

For Yes, the review should satisfy ONE of the following:<br>x *Explanation for* including only RCTs<br>☐ OR *Explanation for* including only NRSI<br>☐ OR *Explanation for* including both RCTs and NRSI	x Yes<br>☐ No

4. Did the review authors use a comprehensive literature search strategy?

For Partial Yes (all the following):<br>x searched at least 2 databases (relevant to research question)<br>x provided key word and/or search strategy<br>x justified publication restrictions (e.g. language)	For Yes, should also have (all the following):<br>x searched the reference lists / bibliographies of included studies<br>x searched trial/study registries<br>x included/consulted content experts in the field<br>x where relevant, searched for grey literature<br>x conducted search within 24 months of completion of the review	x Yes<br>☐ Partial Yes<br>☐ No

5. Did the review authors perform study selection in duplicate?

For Yes, either ONE of the following:

x at least two reviewers independently agreed on selection of eligible studies and achieved consensus on which studies to include<br>🕊 OR two reviewers selected a sample of eligible studies and achieved good agreement (at least 80 percent), with the remainder selected by one reviewer.	x Yes<br>🕊 No

6. Did the review authors perform data extraction in duplicate?

For Yes, either ONE of the following:

- x at least two reviewers achieved consensus on which data to extract from included studies
- ☐ OR two reviewers extracted data from a sample of eligible studies <u>and</u> achieved good agreement (at least 80 percent), with the remainder extracted by one reviewer.

- x Yes
- ☐ No

7. Did the review authors provide a list of excluded studies and justify the exclusions?

For Partial Yes:

- x provided a list of all potentially relevant studies that were read in full-text form but excluded from the review

For Yes, must also have:

- x Justified the exclusion from the review of each potentially relevant study

- x Yes
- ☐ Partial Yes
- ☐ No

8. Did the review authors describe the included studies in adequate detail?

For Partial Yes (ALL the following):

- x described populations
- x described interventions
- x described comparators
- x described outcomes
- x described research designs

For Yes, should also have ALL the following:

- ☐ described population in detail
- ☐ described intervention in detail (including doses where relevant)
- ☐ described comparator in detail (including doses where relevant)
- x described study's setting
- x timeframe for follow-up

- ☐ Yes
- x Partial Yes
- ☐ No

9. Did the review authors use a satisfactory technique for assessing the risk of bias (RoB) in individual studies that were included in the review?

RCTs

For Partial Yes, must have assessed RoB from

- x unconcealed allocation, *and*
- x lack of blinding of patients and assessors when assessing outcomes (unnecessary for objective outcomes such as all-cause mortality)

For Yes, must also have assessed RoB from:

- x allocation sequence that was not truly random, *and*
- x selection of the reported result from among multiple measurements or analyses of a specified outcome

- x Yes
- ☐ Partial Yes
- ☐ No
- ☐ Includes only NRSI

NRSI

For Partial Yes, must have assessed RoB:

- ☐ from confounding, *and*
- ☐ from selection bias

For Yes, must also have assessed RoB:

- ☐ methods used to ascertain exposures and outcomes, *and*
- ☐ selection of the reported result from among multiple measurements or analyses of a specified outcome

- ☐ Yes
- ☐ Partial Yes
- ☐ No
- x Includes only RCTs

10. Did the review authors report on the sources of funding for the studies included in the review?

For Yes

- x Must have reported on the sources of funding for individual studies included in the review. Note: Reporting that the reviewers looked for this information but it was not reported by study authors also qualifies

- x Yes
- ☐ No

11. If meta-analysis was performed did the review authors use appropriate methods for statistical combination of results?

RCTs

For Yes:

x The authors justified combining the data in a meta-analysis<br>x AND they used an appropriate weighted technique to combine study results and adjusted for heterogeneity if present.<br>x AND investigated the causes of any heterogeneity	x Yes<br>☐ No<br>☐ No meta-analysis conducted

For NRSI

For Yes:

x The authors justified combining the data in a meta-analysis<br>x AND they used an appropriate weighted technique to combine study results, adjusting for heterogeneity if present<br>x AND they statistically combined effect estimates from NRSI that were adjusted for confounding, rather than combining raw data, or justified combining raw data when adjusted effect estimates were not available<br>x AND they reported separate summary estimates for RCTs and NRSI separately when both were included in the review	x Yes<br>☐ No<br>☐ No meta-analysis conducted

12. If meta-analysis was performed, did the review authors assess the potential impact of RoB in individual studies on the results of the meta-analysis or other evidence synthesis?

For Yes:

☐ included only low risk of bias RCTs<br>x OR, if the pooled estimate was based on RCTs and/or NRSI at variable RoB, the authors performed analyses to investigate possible impact of RoB on summary estimates of effect.	x Yes<br>☐ No<br>☐ No meta-analysis conducted

13. Did the review authors account for RoB in individual studies when interpreting/ discussing the results of the review?

For Yes:

☐ included only low risk of bias RCTs<br>x OR, if RCTs with moderate or high RoB, or NRSI were included the review provided a discussion of the likely impact of RoB on the results	x Yes<br>☐ No

14. Did the review authors provide a satisfactory explanation for, and discussion of, any heterogeneity observed in the results of the review?

For Yes:

☐ There was no significant heterogeneity in the results<br>x OR if heterogeneity was present the authors performed an investigation of sources of any heterogeneity in the results and discussed the impact of this on the results of the review	x Yes<br>☐ No

15. If they performed quantitative synthesis did the review authors carry out an adequate investigation of publication bias (small study bias) and discuss its likely impact on the results of the review?

For Yes:

x performed graphical or statistical tests for publication bias and discussed the likelihood and magnitude of impact of publication bias	x Yes<br>☐ No<br>☐ No meta-

	analysis conducted

16. Did the review authors report any potential sources of conflict of interest, including any funding they received for conducting the review?

For Yes:	
x The authors reported no competing interests OR	x Yes
🕊 The authors described their funding sources and how they managed potential conflicts of interest	🕊 No

Supplemental Data 3 – Amendments to the protocol since registration

Firstly, we decided to use “physiotherapy” as an additional control intervention, since it did not fit into the pre-defined control interventions but served as an important comparator. Secondly, those exercise interventions that were not delivered digitally but conducted at home were classified as “exercise home” to account for potentially different effect sizes compared to “exercise in-person”.

Supplemental Data 4 – Exclusion criteria

POPULATION

- NOT adult (≥18 years)
- NOT sub-acute or chronic (>6 weeks)
    - if a study calls their collective "sub-acute" or "chronic" then it is included
    - if for chronic conditions the inclusion criteria required a minimum of 12wks duration of pain OR the reported mean/median duration of pain in the results section is 12 weeks or greater then it should be included.

      > Handling of "**Duration" at title/abstract stage**:
        - if abstract says 'acute and sub-acute', then go to full-text (check if mean/median pain duration is min. 6 weeks)
        - if abstract says 'acute', then exclude

    - recurrent back pain = EXCLUDED where clearly fits **criterion of 6month break between episodes** for recurrent back pain
- NOT low back disorder
    - <u>EXCLUDED</u> = pregnancy, labour or post-partum related back pain
    - <u>EXCLUDED</u> = specific spinal pathology (i.e. vertebral fracture, malignancy, spinal infection, axial spondyloarthritis, cauda equina syndrome)
    - <u>EXCLUDED</u> = post-operative pain if the study is clearly about "immediate post-operative pain", which is iatrogenic pain
    - <u>EXCLUDED</u> = secondary back pain
    - <u>EXCLUDED</u> = generalised MSK pain
    - <u>EXCLUDED</u> = radicular pain

INTERVENTION/COMPARATOR

- NOT one of the following treatments (digitally at least for intervention group):
    - Education
    - Exercise
    - Psychological therapies
    - Usual care
    - No treatment (true control)
    - Waiting list
    - Included combinations of the above categories
- Studies evaluating the effectiveness of wearable technology, movement trackers, or virtual reality will be excluded. Studies using telephone calls or text messaging only as reminder of an in-person intervention without a bilateral exchange with the participants targeted to specific goals will be excluded.
- If a study includes interventions that are classified as the same treatment, are both delivered digitally and do not have an additional group with a different treatment (e.g. group 1: Exercise digital, group 2: Exercise digital) or the additional group has a treatment that doesn't fall under the inclusion criteria (e.g. group 1: Exercise digital, group 2: Exercise digital, group 3: Surgery), this study will be excluded.

OUTCOME

- NOT including at least one of the following outcomes:
  - Pain intensity
  - Disability

STUDY DESIGN

- NOT randomised controlled/clinical trial/cross-over trial (cluster-design included)

LANGUAGE

- NOT: English or German

ARTICLE

- NOT **fully** peer-reviewed journal publication (EXCLUDED: conference papers, theses, grey literature)
- **Missing abstract**: If a title does not have an abstract and the title is 'potentially included', then it is included at the stage of full text screening

Supplemental Data 5 – Definition of intervention components

- Education: Education has previously been defined as "*as any advice or information (verbal, written or audiovisual) given by a health care professional in order to improve patients' understanding of their […] problems and what they should do about them*".[1] Therefore, any information about the patients' condition, advice on staying physically active as well as educative interventions aiming to change the patients' behaviour were included and classified as 'education'.

- Physical activity and/or exercise training: We used the definitions of exercise training as "*a series of specific movements with the aim of training or developing the body by a routine practice or as physical training to promote good physical health*"[2] as well as "*physical activity that is planned, structured, repetitive, and purposive in the sense that improvement or maintenance of one or more components of physical fitness is an objective*"[3] for the inclusion criteria. Every intervention covered by these definitions was included.

- Psychological interventions: Interventions that address psychological aspects and aim to improve the mental health as part of the overall pain management. Interventions including cognitive-behavioural strategies that "*include, but are not limited to, cognitive restructuring, relaxation techniques, […] and sleep hygiene*"[4] were classified as cognitive behavioural therapy and included.

Supplemental Data 6 – Manually searched systematic reviews

Supplemental Table 3 – Manually searched systematic reviews

Author, year	Title	doi
Molina-Garcia, 2024	Effectiveness and cost-effectiveness of telerehabilitation for musculoskeletal disorders: A systematic review and meta-analysis	https://doi.org/10.1016/j.rehab.2023.101791
de Melo Santana, 2023	Efficacy of mHealth Interventions for Improving the Pain and Disability of Individuals With Chronic Low Back Pain: Systematic Review and Meta-Analysis	https://doi.org/10.2196/48204
Hong, 2023	Software-based interventions for low back pain management: A systematic review and meta-analysis	https://doi.org/10.1111/jnu.12937
Mitchai, 2023	Mobile technologies for rehabilitation in non-specific spinal disorders: a systematic review of the efficacy and potential for implementation in low- and middle-income countries	https://doi.org/10.1007/s00586-023-07964-2
Moreno-Ligero, 2023	mHealth Intervention for Improving Pain, Quality of Life, and Functional Disability in Patients With Chronic Pain: Systematic Review	https://doi.org/10.2196%2F40844
Toonders, 2022	Effectiveness of remote physiotherapeutic e-Health interventions on pain in patients with musculoskeletal disorders: a systematic review	https://doi.org/10.1080/09638288.2022.2135775
Stark, 2022	App-Based Rehabilitation in Back Pain, a Systematic Review	https://doi.org/10.3390%2Fjpm12101558
Valentijn, 2022	Digital Health Interventions for Musculoskeletal Pain Conditions: Systematic Review and Meta-analysis of Randomized Controlled Trials	https://doi.org/10.2196%2F37869
Lara-Palomo, 2022	Efficacy of e-Health Interventions in Patients with Chronic Low-Back Pain: A Systematic Review with Meta-Analysis	https://doi.org/10.1089/tmj.2021.0599
Didyk, 2022	Effectiveness of smartphone apps for the self-management of low back pain in adults: a systematic review	https://doi.org/10.1080/09638288.2021.2005161
Lewkowicz, 2021	Digital Therapeutic Care and Decision Support Interventions for People With Low Back Pain: Systematic Review	https://doi.org/10.2196%2F26612
Adams	The effectiveness of exercise-based telemedicine on pain, physical	https://doi.org/

e, 2018	activity and quality of life in the treatment of chronic pain: A systematic review	10.1177/1357633X17716576
Chen, 2021	Efficacy of Mobile Health in Patients With Low Back Pain: Systematic Review and Meta-analysis of Randomized Controlled Trials	https://doi.org/10.2196/26095
Cottrell, 2016	Real-time telerehabilitation for the treatment of musculoskeletal conditions is effective and comparable to standard practice: a systematic review and meta-analysis	https://doi.org/10.1177/0269215516645148
Cuenca-Martinez, 2022	Implementation of Online Behavior Modification Techniques in the Management of Chronic Musculoskeletal Pain: A Systematic Review and Meta-Analysis	https://doi.org/10.3390/jcm11071806
de Olivieira Lima, 2021	Self-Guided Web-Based Pain Education for People With Musculoskeletal Pain: A Systematic Review and Meta-Analysis	https://doi.org/10.1093/ptj/pzab167
Dias, 2021	Effectiveness of exercises by telerehabilitation on pain, physical function and quality of life in people with physical disabilities: a systematic review of randomised controlled trials with GRADE recommendations	https://doi.org/10.1136/bjsports-2019-101375
Du, 2020	The efficacy of e-health in the self-management of chronic low back pain: A meta analysis	https://doi.org/10.1016/j.ijnurstu.2019.103507
Du, 2017	Self-management program for chronic low back pain: A systematic review and meta-analysis	https://doi.org/10.1016/j.pec.2016.07.029
Eccleston, 2014	Psychological therapies (Internet-delivered) for the management of chronic pain in adults	https://doi.org/10.1002/14651858.CD010152.pub2
Mehta, 2018	Internet-delivered cognitive behaviour therapy for chronic health conditions: a systematic review and meta-analysis	https://doi.org/10.1007/s10865-018-9984-x
Moman, 2019	A Systematic Review and Meta-analysis of Unguided Electronic and Mobile Health Technologies for Chronic Pain-Is It Time to Start Prescribing Electronic Health Applications?	https://doi.org/10.1093/pm/pnz164
O'Brien, 2018	Effectiveness of telephone-based interventions for managing osteoarthritis and spinal pain: a systematic review and meta-analysis	https://doi.org/10.7717/peerj.5846

Supplemental Data 7 – Search strategy

Date of database search: 11/04/2024

MEDLINE

Search	Query	Hits
#1	back pain[MeSH Terms] OR low back pain[MeSH Terms] OR sciatica[MeSH Terms] OR back pain*[Title/Abstract] OR lumb* pain[Title/Abstract] OR lumbago[Title/Abstract] OR backache*[Title/Abstract] OR back ache*[Title/Abstract]	103,287
#2	"internet"[MeSH Terms] OR "mobile applications"[MeSH Terms] OR "telemedicine"[MeSH Terms] OR "telerehab*"[Title/Abstract] OR "tele rehab*"[Title/Abstract] OR "mhealth"[Title/Abstract] OR "app"[Title/Abstract] OR "ehealth"[Title/Abstract] OR "online"[Title/Abstract] OR "telemed*"[Title/Abstract] OR "telehealth"[Title/Abstract] OR "e-health"[Title/Abstract] OR "m-health"[Title/Abstract] OR "digital health"[Title/Abstract] OR "videoconferenc*"[Title/Abstract] OR "telehealthcare"[Title/Abstract] OR "tele-healthcare"[Title/Abstract]	449,750
#3	"random*"[Title/Abstract]	1,509,935
#4	#1 AND #2	1,278
#5	#3 AND #4	338

Results after search update on 20/05/2025: 409

SPORTDiscus

Search	Query	Hits
#1	(DE "lumbar pain") OR (DE backache) OR (DE sciatica) OR (TI ("back pain*" OR "lumb* pain" OR lumbago OR backache* OR "back ache*") OR AB ("back pain*" OR "lumb* pain" OR lumbago OR backache* OR "back ache*"))	12,053
#2	(TI (telerehab* or "tele rehab*" or telemed* or telehealth or tele-health or telehealthcare or tele-healthcare or internet or e-health or ehealth or m-health or mhealth or app or "digital health" or videoconferenc*)) OR (AB (telerehab* or "tele rehab*" or telemed* or telehealth or tele-health or telehealthcare or tele-healthcare or internet or e-health or ehealth or m-health or mhealth or app or "digital health" or videoconferenc*))	11,694
#3	TI (random*) OR AB (random*)	71,049
#4	#1 AND #2	49
#5	#3 AND #4	19

Results after search update on 20/05/2025: 20

CINAHL (via EBSCOhost)

Search	Query	Hits
#1	(MH low back pain) OR (MH back pain) OR (TI ("back pain*" OR "lumb* pain" OR lumbago OR backache* OR "back ache*") OR AB ("back pain*" OR "lumb* pain" OR lumbago OR backache* OR "back ache*"))	44,617
#2	(TI (telerehab* or "tele rehab*" or telemed* or telehealth or tele-health or telehealthcare or tele-healthcare or internet or e-health or ehealth or m-health or mhealth or app or "digital health" or videoconferenc*)) OR (AB (telerehab* or "tele rehab*" or telemed* or telehealth or tele-health or telehealthcare or tele-healthcare or internet or e-health or ehealth or m-health or mhealth or app or "digital health" or videoconferenc*))	72,757
#3	TI (random*) OR AB (random*)	455,865
#4	#1 AND #2	268

#5	#3 AND #4	89

Results after search update on 20/05/2025: 25

EMBASE

Search	Query	Hits
#1	low back pain/ or backache/	139,995
#2	("back pain*" or "lumb* pain" or "lumbago" or "backache*" or "back ache*").ab. or ("back pain*" or "lumb* pain" or "lumbago" or "backache*" or "back ache*").ti.	93,599
#3	#1 OR #2	156,925
#4	telemedicine/ or telerehabilitation/ or telehealth/ or Internet/ or mobile application/	208,760
#5	(telerehab* or “tele rehab*” or telemed* or telehealth or tele-health or telehealthcare or tele-healthcare or internet or e-health or ehealth or m-health or mhealth or app or “digital health” or videoconferenc*).ab. or (telerehab* or “tele rehab*” or telemed* or telehealth or tele-health or telehealthcare or tele-healthcare or internet or e-health or ehealth or m-health or mhealth or app or “digital health” or videoconferenc*).ti.	212,323
#6	#4 OR #5	309,288
#7	#3 AND #6	1,136
#8	random*.ab. or random*.ti.	2,054,468
#9	#7 AND #8	284
#10	Limit 9 to “remove medline records”	76

Results after search update on 20/05/2025: 123

CENTRAL

Search	Query	Hits
#1	(MeSH descriptor: [back pain] explode all trees) OR (MeSH descriptor: [low back pain] explode all trees) OR ("low back pain" or "back pain" or "lbp" or "lumbago" or "backache"):ti,ab,kw (Word variations have been searched)	21,578
#2	(MeSH descriptor: [Telemedicine] explode all trees) OR (MeSH descriptor: [Telerehabilitation] explode all trees) OR (MeSH descriptor: [Mobile Applications] explode all trees) OR (telerehab* or tele*rehab or telemed* or telehealth or tele*health or telehealthcare or tele*healthcare or telehealth*care or internet or e-health or ehealth or m-health or mhealth or app or videoconference* or “digital health”):ti,ab,kw (Word variations have been searched)	37,398
#3	#1 AND #2	434
#4	(random*):ti,ab,kw	1,328,430
#5	#4 AND #5 Filters: Trials	326

Results after search update on 20/05/2025: 369

PsycINFO (via EBSCOhost)

Search	Query	Hits
#1	DE "Back Pain"	4,720
#2	TI (“back pain*” OR “lumb* pain” OR lumbago OR backache* OR “back ache*”) OR AB (“back pain*” OR “lumb* pain” OR lumbago OR backache* OR “back ache*”)	6,167

#3	#1 OR #2	6,746
#4	DE "Telemedicine" OR DE "Online Therapy" OR DE "Teleconferencing" OR DE "Teleconsultation" OR DE "Telerehabilitation" OR DE "Mobile Applications" OR DE "Mobile Devices" OR DE "Mobile Health" OR DE "Internet"	57,240
#5	(TI (telerehab* or "tele rehab*" or telemed* or telehealth or tele-health or telehealthcare or tele-healthcare or internet or e-health or ehealth or m-health or mhealth or app or "digital health" or videoconferenc*)) OR (AB (telerehab* or "tele rehab*" or telemed* or telehealth or tele-health or telehealthcare or tele-healthcare or internet or e-health or ehealth or m-health or mhealth or app or "digital health" or videoconferenc*))	64,453
#6	#4 OR #5	90,390
#7	#3 AND #6	123
#8	TI random* or AB random*	220,116
#9	#7 AND #8	50

Results after search update on 20/05/2025: 98

WHO + NIH trial registry

Search	Query	Hits
#1	(back pain OR low back pain OR sciatica OR back pain* OR lumb* pain OR lumbago OR backache* OR back ache*) AND ("internet" OR "mobile applications" OR "telemedicine" OR "telerehab*" OR "tele rehab*" OR "mhealth" OR "app" OR "ehealth" OR "online" OR "telemed*" OR "telehealth" OR "e-health" OR "m-health" OR "digital health" OR "videoconferenc*" OR "telehealthcare" OR "tele-healthcare")	944

Results from forward and backward citation tracking: 992

Date of citation tracking: 06/09/2024

Supplemental Data 8 – Hierarchy of outcome assessments

If multiple versions of an individual outcome were presented, the most commonly used measure (as per the priority list) were extracted and used in further analysis:

- Pain intensity:
  - VAS (0-10cm)
  - VAS (0-100mm)
  - NRS or NPRS (0-10)
  - NRS or NPRS (0-100)
  - Borg back pain intensity scale (10-point scale)
  - Brief pain inventory (BPI) short form (0-10 points)
  - Brief pain inventory (BPI) long form (0-10 points)
  - Other validated pain intensity measures
- Disability:
  - Oswestry Disability Index (0-100 points)
  - Modified Oswestry Index (0-100 points)
  - Roland Morris Disability Questionnaire (0-24 points)
  - Modified Roland Morris Disability Questionnaire (23 questions)
  - Quebec Back Pain Disability Scale (0-100 points)
  - SF-36 (physical function subscale; 100 to 0 points)
  - SF-12 (physical function subscale; 100 to 0 points)
  - Funktionsfragebogen Hannover (FFbH-R) 0-100 points
  - Other validated disability measures

Supplemental Data 9 – Data handling

We used the recommendations of the Cochrane handbook to transform data to the desired measures.[5]

Transformation of different measures of spread

95%-confidence interval to standard deviation (assumes outcome is approximately normally distributed):

$$SD = \sqrt{n} \times \frac{Upper\ Limit - Lower\ Limit}{2 \times t_{\left(1-\frac{\alpha}{2}, df=n-1\right)}}$$

Standard error to standard deviation:

$$SD = SE \times \sqrt{n}$$

Mean from median and quartiles (assumes outcome is symmetrically distributed):

$$Mean \approx \frac{Median + Q_1 + Q_3}{3}$$

Standard deviation from quartiles (assumes outcome is approximately normally distributed):

$$SD \approx \frac{Q_3 - Q_1}{2 \times qnorm\left(\frac{0.75 \times n - 0.125}{n + 0.25}\right)}$$

Mean from 95%-confidence interval where only median is reported (assumes outcome is symmetrically distributed):

$$Mean \approx \frac{Upper\ Limit + Lower\ Limit}{2}$$

Standard deviation of change from baseline:

$$SD_{change} = \sqrt{SD_{baseline}^2 + SD_{follow-up}^2 - 2 \times Corr \times SD_{baseline} \times SD_{follow-up}}$$

Handling of cluster trials

To adjust the effect of cluster trials for cluster-based randomization, we calculated the effective sample sizes of the trials as per the recommendations of the Cochrane Handbook.[6] This is done by dividing the original sample size by the design effect, which is defined as follows:

$$1 + (M - 1) \times ICC$$

M is the average cluster size, and ICC is the intracluster correlation coefficient. We used an ICC of 0.0325 based on Adams et al. 2004.[7]

Calculation of internal reference standard deviation

We calculated a pooled SD for each study with the following formula:

$$SD_{pooled}=\sqrt{\frac{\sum_{i=1}^{k}(n_i-1)\times SD_i^2}{\sum_{i=1}^{k}(n_i-1)}}$$

We then calculated the mean of pooled SDs for each outcome scale to be further used in data transformation.

Pooling of intervention arms with the same intervention

Pooled sample size

$$n_{combined}=n_1+n_2$$

Pooled mean

$$Mean_{combined}=\frac{n_1\times\bar{x}_1+n_2\times\bar{x}_2}{n_1+n_2}$$

Pooled standard deviation

$$SD_{pooled}=\sqrt{\frac{(n_1-1)\times SD_1^2+(n_2-1)\times SD_2^2+(\frac{n_1\times n_2}{n_1+n_2})\times(\bar{x}_1-\bar{x}_2)^2}{n_1+n_2-1}}$$

Imputation of missing SD

Accordingly to Furukawa et al. (2006)[8] we used the pooled SD from studies that used the same outcome scale at the same time-point to calculate the SD to be imputed.

$$SD_{pooled}=\sqrt{\frac{\sum_{i=1}^{k}(n_i-1)\times SD_i^2}{\sum_{i=1}^{k}(n_i-1)}}$$

Supplemental Data 10 – RoB guidance sheet

Changes specifically made for this review compared to the original guidance are written in red color.

Domain 1: Risk of bias arising from the randomization process

Signalling questions	Elaboration	Response options
**1.1 Was the allocation sequence random?**	Answer 'Yes' if a random component was used in the sequence generation process. Examples include computer-generated random numbers; reference to a random number table; coin tossing; shuffling cards or envelopes; throwing dice; or drawing lots. Minimization is generally implemented with a random element (at least when the scores are equal), so an allocation sequence that is generated using minimization should generally be considered to be random.<br>Answer 'No' if no random element was used in generating the allocation sequence or the sequence is predictable. Examples include alternation; methods based on dates (of birth or admission); patient record numbers; allocation decisions made by clinicians or participants; allocation based on the availability of the intervention; or any other systematic or haphazard method.<br>Answer 'No information' if the only information about randomization methods is a statement that the study is randomized.<br>In some situations a judgement may be made to answer 'Probably no' or 'Probably yes'. For example, in the context of a large trial run by an experienced clinical trials unit, absence of specific information about generation of the randomization sequence, in a paper published in a journal with rigorously enforced word count limits, is likely to result in a response of 'Probably yes' rather than 'No information'. Alternatively, if other (contemporary) trials by the same investigator team have clearly used non-random sequences, it might be reasonable to assume that the current study was done using similar methods.	Y/PY/PN/N/NI
**1.2 Was the allocation**	Answer 'Yes' if the trial used any form of remote or centrally administered method to allocate	Y/PY/PN/N/NI

**sequence concealed until participants were enrolled and assigned to interventions?**	interventions to participants, where the process of allocation is controlled by an external unit or organization, independent of the enrolment personnel (e.g. independent central pharmacy, telephone or internet-based randomization service providers).<br><br>Answer 'Yes' if envelopes or drug containers were used appropriately. Envelopes should be opaque, sequentially numbered, sealed with a tamper-proof seal and opened only after the envelope has been irreversibly assigned to the participant.<br><br>Answer 'No' if there is reason to suspect that the enrolling investigator or the participant had knowledge of the forthcoming allocation. This is also the case in block randomization with fixed numbers.	

**1.3 Did baseline differences between intervention groups suggest a problem with the randomization process?**	Always answer 'No'. Rationale: CONSORT recommends not to compare baseline data.<br><br>Exception: When the baseline table doesn't contain information on the whole sample but only on those who completed the study, choose 'No information'.	N/NI

**Risk-of-bias judgement**	See algorithm.	Low / High / Some concerns

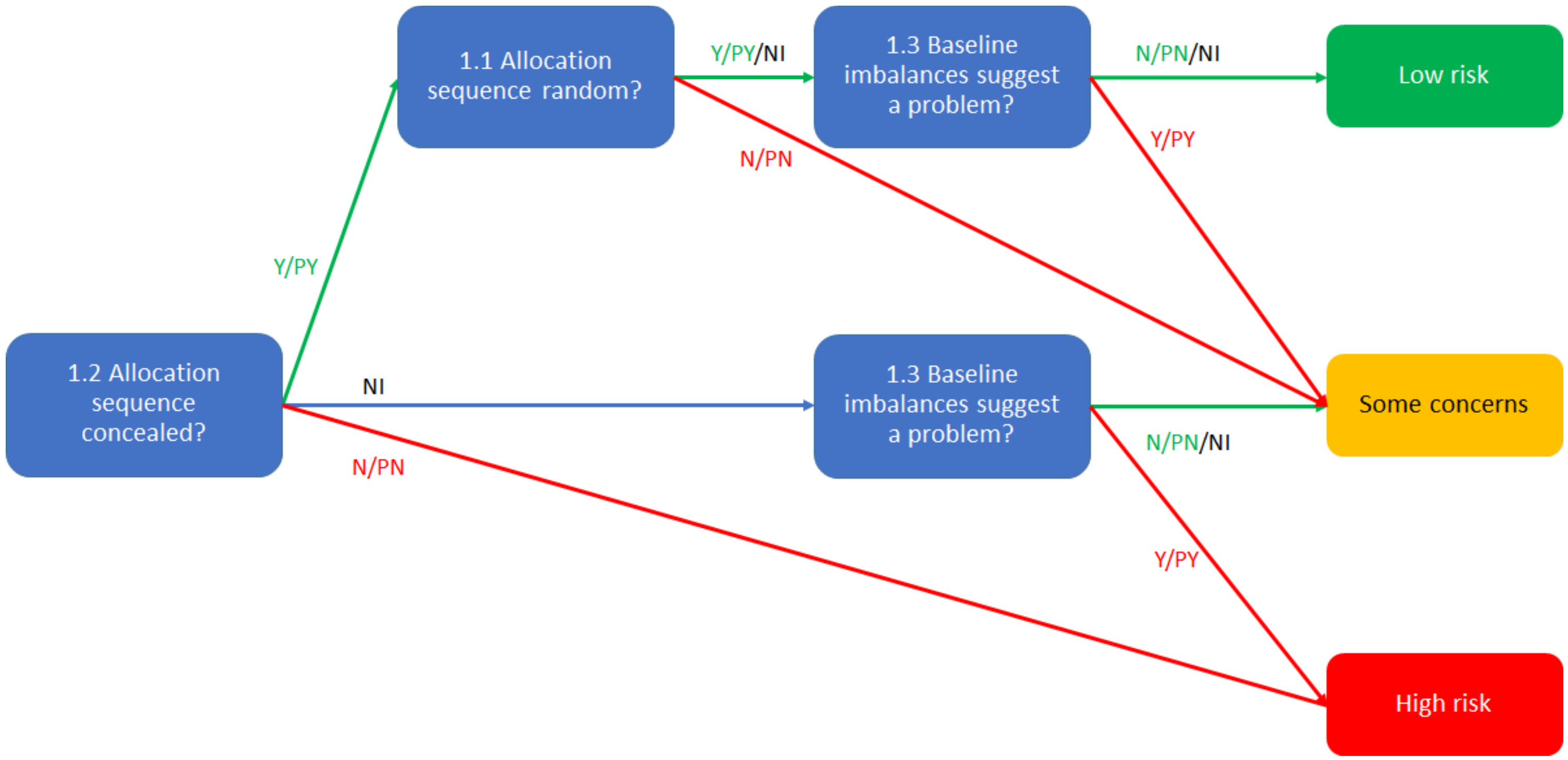

Algorithm for suggested judgement of risk of bias arising from the randomization process

Domain 2: Risk of bias due to deviations from the intended interventions (*effect of assignment to intervention*)

Signalling questions	Elaboration	Response options
**2.1. Were participants aware of their assigned intervention during the trial?**	If participants are aware of their assigned intervention it is more likely that health-related behaviours will differ between the intervention groups. Blinding participants, most commonly through use of a placebo or sham intervention, may prevent such differences.<br><br>Answer 'No' if it the control group is a credible placebo group.<br>Answer 'Yes' in all other cases, since blinding is not possible in this context.	Y/PY/PN/N/NI

**2.2. Were carers and people delivering the interventions aware of participants' assigned intervention during the trial?**	Answer always 'Yes'.	Y

**2.3. If Y/PY/NI to 2.1 or 2.2: Were there deviations from the intended intervention that arose because of the trial context?**	Answer 'Yes' or 'Probably yes' **only** if there is evidence, or strong reason to believe, that the trial context led to failure to implement the protocol interventions or to implementation of interventions not allowed by the protocol.<br><br>Answer 'Probably No' in all other cases, when changes to interventions are not suspicious.	NA/Y/PY/PN/N/NI
**2.4 If Y/PY to 2.3: Were these deviations likely to have affected the outcome?**	Always answer 'Probably yes'.	PY

**2.5. If Y/PY/NI to 2.4: Were these deviations from intended intervention balanced between groups?**	Always answer 'Probably no'.	PN
**2.6 Was an appropriate analysis used to estimate the effect of assignment to intervention?**	Both intention-to-treat (ITT) analyses and modified intention-to-treat (mITT) analyses excluding participants with missing outcome data should be considered appropriate. Both naïve 'per-protocol' analyses (excluding trial participants who did not receive their assigned intervention) and 'as treated' analyses (in which trial participants are grouped according to the intervention that they received, rather than according to their assigned intervention) should be considered inappropriate. Analyses excluding eligible trial participants post-randomization should also be considered inappropriate, but post- randomization exclusions of ineligible participants (when eligibility was not confirmed until after randomization, and could not have been influenced by intervention group assignment) can be considered appropriate.	Y/PY/PN/N/NI
**2.7 If N/PN/NI to 2.6: Was there potential for a substantial impact (on the result) of the failure to analyse participants in the group to which they were randomized?**	This question addresses whether the number of participants who were analysed in the wrong intervention group, or excluded from the analysis, was sufficient that there could have been a substantial impact on the result. It is not possible to specify a precise rule: there may be potential for substantial impact even if fewer than 5% of participants were analysed in the wrong group or excluded, if the outcome is rare or if exclusions are strongly related to prognostic factors.	NA/Y/PY/PN/N/NI
**Risk-of-bias judgement**	See algorithm.	Low / High / Some concerns

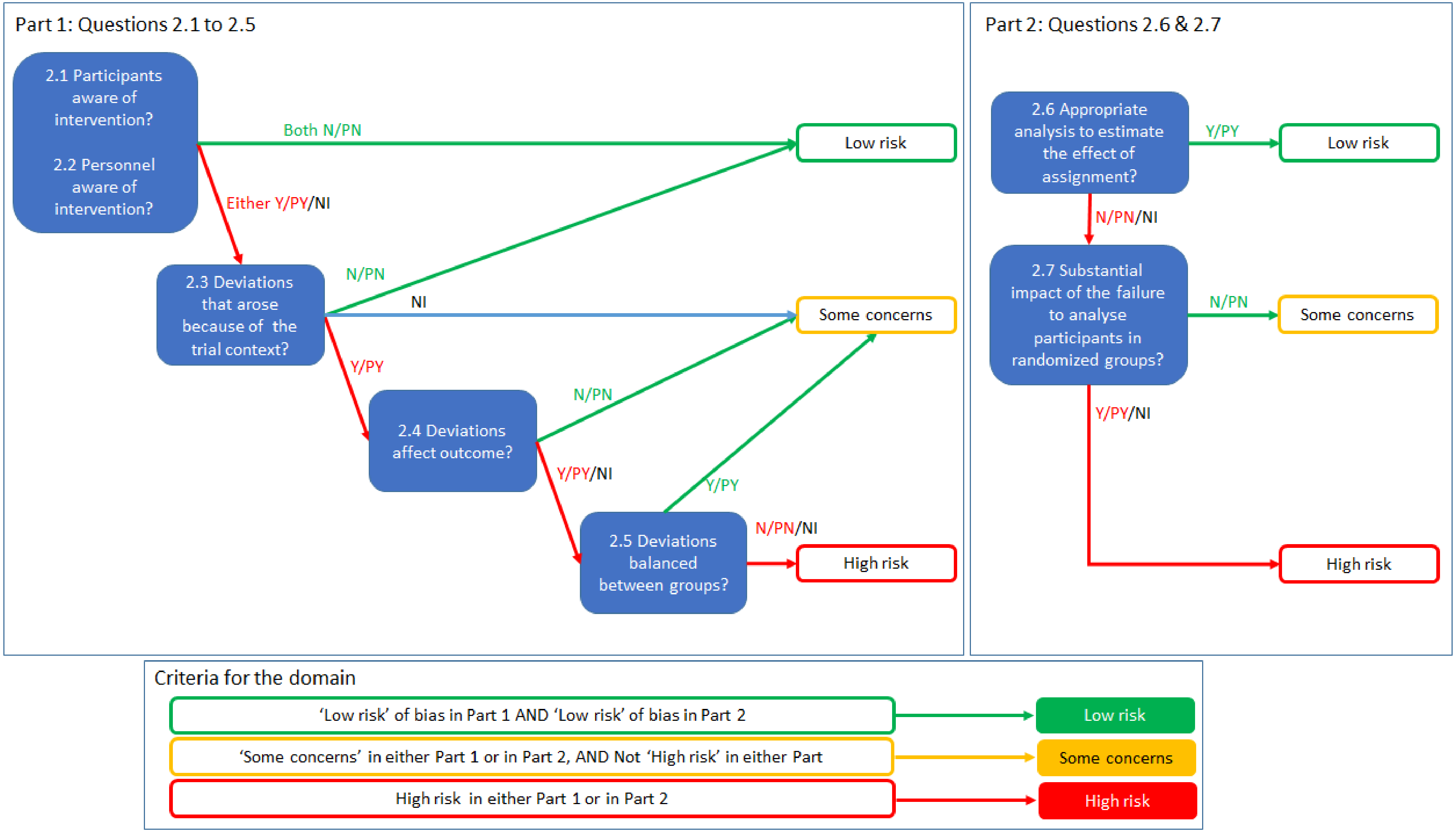

Algorithm for suggested judgement of risk of bias due to deviations from the intended interventions (effect of assignment to intervention)

Domain 3: Risk of bias due to missing outcome data

Signaling questions	Elaboration	Response options
**3.1 Were data for this outcome available for all, or nearly all, participants randomized?**	Answer 'Yes' if the trial authors accounted for a % amount of dropouts/missing data in their power calculation (and pain intensity was the primary outcome) and the actual amount of missing data is below that. If no power calculation occurred (e.g. in pilot trials) or the trial was powered for another outcome, >90% of participants should have reported data.<br>Answer 'No' if either the threshold of the power calculation has not been met or data from 90% or less participants is available (if no power calculation has been done).<br>Only answer 'No information' if the trial report provides no information about the extent of missing outcome data. This situation will usually lead to a judgement that there is a high risk of bias due to missing outcome data.<br>Note that imputed data should be regarded as missing data, and not considered as 'outcome data' in the context of this question.	Y/PY/PN/N/NI
**3.2 If N/PN/NI to 3.1: Is there evidence that the result was not biased by missing outcome data?**	Evidence that the result was not biased by missing outcome data may come from: (1) analysis methods that correct for bias; or (2) sensitivity analyses showing that results are little changed under a range of plausible assumptions about the relationship between missingness in the outcome and its true value. However, imputing the outcome variable, either through methods such as 'last-observation-carried- forward' or via multiple imputation based only on intervention group, should not be assumed to correct for bias due to missing outcome data.	NA/Y/PY/PN/N
**3.3 If N/PN to 3.2: Could missingness in the outcome depend on its true value?**	If loss to follow up, or withdrawal from the study, could be related to participants' health status, then it is possible that missingness in the outcome was influenced by its true value. However, if all missing outcome data occurred for documented reasons that are unrelated to the outcome then the risk of bias due to missing outcome data will be low (for example, failure of a measuring device or interruptions to routine data collection).	NA/Y/PY/PN/N/NI

**3.4 If Y/PY/NI to 3.3: Is it likely that missingness in the outcome depended on its true value?**	Answer 'Probably yes' if: 1) proportion of missing data are unbalanced for lack of efficacy or adverse events; 2) if reasons for missing are reported and depend on the true value (i.e. lack of efficacy); 3) if reasons are different between the groups (e.g. more patients in the one group dropped out for AE and more patients in the other group dropped out for lack of efficacy).<br>Answer 'No' if the analysis accounted for participant characteristics that are likely to explain the relationship between missingness in the outcome and its true value.	NA/Y/PY/PN/N/NI
**Risk-of-bias judgement**	See algorithm.	Low / High / Some concerns

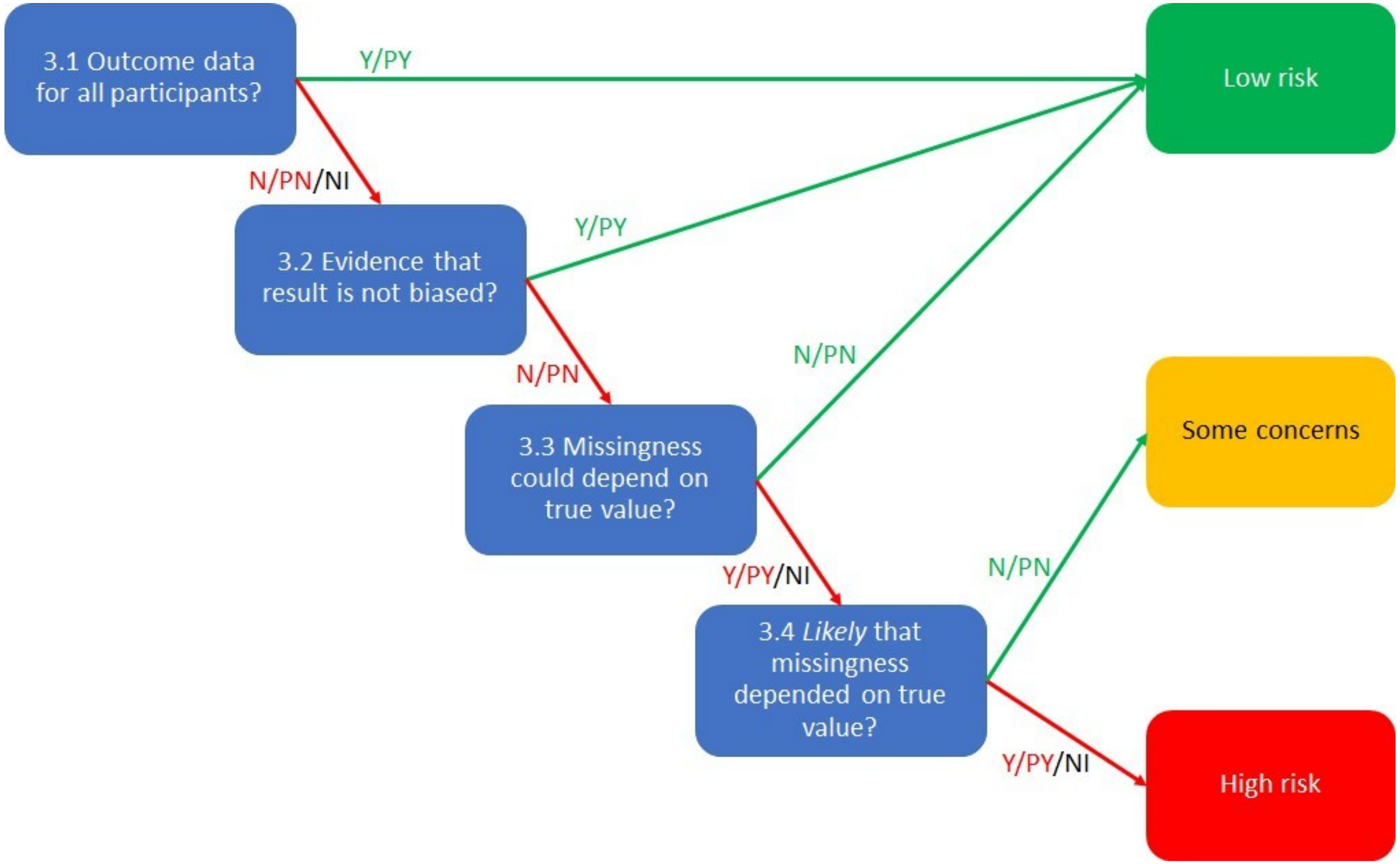

Algorithm for suggested judgement of risk of bias due to missing outcome data

Domain 4: Risk of bias in measurement of the outcome

Signalling questions	Elaboration	Response options
**4.1 Was the method of measuring the outcome inappropriate?**	This question aims to identify methods of outcome measurement (data collection) that are unsuitable for the outcome they are intended to evaluate. The question *does not* aim to assess whether the choice of outcome being evaluated was sensible (e.g. because it is a surrogate or proxy for the main outcome of interest). In most circumstances, for pre-specified outcomes, the answer to this question will be 'No' or 'Probably no'. <br>Answer 'Yes' or 'Probably yes' if the method of measuring the outcome is inappropriate, for example because the measurement instrument has been demonstrated to have poor validity. <br>Note: All outcomes in the extraction sheet are considered to result in a 'Yes'.	Y/PY/PN/N/NI
**4.2 Could measurement or ascertainment of the outcome have differed between intervention groups?**	Comparable methods of outcome measurement (data collection) involve the same measurement methods and thresholds, used at comparable time points. Differences between intervention groups may arise because of 'diagnostic detection bias' in the context of passive collection of outcome data, or if an intervention involves additional visits to a healthcare provider, leading to additional opportunities for outcome events to be identified.	Y/PY/PN/N/NI
**4.3 If N/PN/NI to 4.1 and 4.2: Were outcome assessors aware of the intervention received by study participants?**	Answer 'No' if outcome assessors were blinded to intervention status. For participant-reported outcomes, the outcome assessor is the study participant. Answer only 'No' if blinding was indicated in 2.1.	NA/Y/PY/PN/N/NI

**4.4 If Y/PY/NI to 4.3: Could assessment of the outcome have been influenced by knowledge of intervention received?**	Knowledge of the assigned intervention could influence participant-reported outcomes (such as level of pain). 'No' only possible if participants were blinded (s. 2.1).	NA/Y/PY/PN/N/NI

**4.5 <u>If Y/PY/NI to 4.4</u>: Is it likely that assessment of the outcome was influenced by knowledge of intervention received?**	Answer 'Yes' if the control group was no intervention, waiting list or other minimal intervention. Answer 'No' in all other cases.	NA/Y/PY/<u>PN/N</u>/NI
**Risk-of-bias judgement**	See algorithm.	Low / High / Some concerns

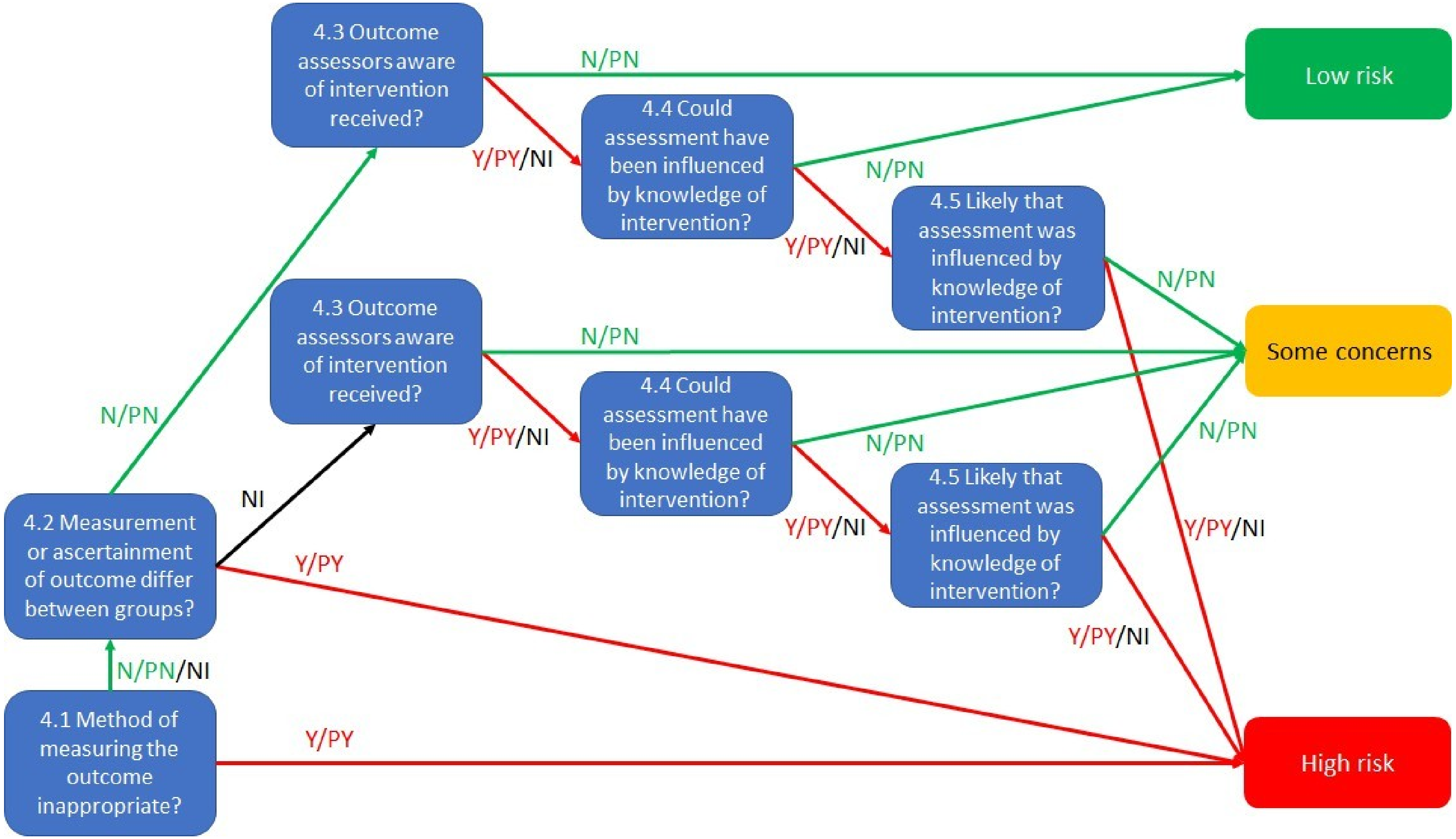

Algorithm for suggested judgement of risk of bias in measurement of the outcome

Domain 5: Risk of bias in selection of the reported result

Signalling questions	Elaboration	Response options
**5.1 Were the data that produced this result analysed in accordance with a pre-specified analysis plan that was finalized before unblinded outcome data were available for analysis?**	If the researchers' pre-specified intentions are available in sufficient detail, then planned outcome measurements can be compared with those presented in the published report(s). To avoid the possibility of selection of the reported result, finalization of the intentions must precede availability of unblinded outcome data to the trial investigators.<br>Changes to analysis plans that were made before unblinded outcome data were available, or that were clearly unrelated to the results (e.g. due to a broken machine making data collection impossible) do not raise concerns about bias in selection of the reported result.<br>Answer 'No' if the trial was not registered or retrospectively registered and/or no protocol with a plan for analysis was published.	Y/PY/PN/N/NI
**Is the numerical result being assessed likely to have been selected, on the basis of the results, from...**		
**5.2.....multiple eligible outcome measurements (e.g. scales, definitions, time points) within the outcome domain?**	A particular outcome domain (i.e. a true state or endpoint of interest) may be **measured** in multiple ways. For example, the domain pain may be measured using multiple scales (e.g. a visual analogue scale and the McGill Pain Questionnaire), each at multiple time points (e.g. 3, 6 and 12 weeks post- treatment). If multiple measurements were made, but only one or a subset is reported on the basis of the results (e.g. statistical significance), there is a high risk of bias in the fully reported result.<br>Attention should be restricted to outcome measurements that are eligible for consideration by the RoB 2 tool user. For example, if only a result using a specific measurement scale is eligible for inclusion in a meta-analysis (e.g. Hamilton Depression Rating Scale), and this is reported by the trial, then there would not be an issue of selection even if this result was reported (on the basis of the results) in preference to the result from a different measurement scale (e.g. Beck Depression Inventory).<br>Answer 'Yes' or 'Probably yes' if:<br>There is clear evidence (usually through examination of a trial protocol or statistical analysis plan) that a domain was measured in multiple eligible ways, but data for only one or a subset of measures is fully reported (without justification), and the fully reported result	Y/PY/PN/N/NI

	is likely to have been selected on the basis of the results. Selection on the basis of the results can arise from a desire for findings to be newsworthy, sufficiently noteworthy to merit publication, or to confirm a prior<br>hypothesis. For example, trialists who have a preconception, or vested interest in showing, that an	
	experimental intervention is beneficial may be inclined to report outcome measurements selectively that are favourable to the experimental intervention.<br>Answer 'No' or 'Probably no' if:<br>There is clear evidence (usually through examination of a trial protocol or statistical analysis plan) that all eligible reported results for the outcome domain correspond to all intended outcome measurements.<br>or<br>There is only one possible way in which the outcome domain can be measured (hence there is no opportunity to select from multiple measures).<br>or<br>Outcome measurements are inconsistent across different reports on the same trial, but the trialists have provided the reason for the inconsistency and it is not related to the nature of the results.<br>Answer 'No information' if:<br>Analysis intentions are not available, or the analysis intentions are not reported in sufficient detail to enable an assessment, and there is more than one way in which the outcome domain could have been measured.	
**5.3 ... multiple eligible analyses of the data?**	A particular outcome measurement may be analysed in multiple ways. Examples include: unadjusted and adjusted models; final value vs change from baseline vs analysis of covariance; transformations of variables; different definitions of composite outcomes (e.g. 'major adverse event'); conversion of continuously scaled outcome to categorical data with different cut-points; different sets of covariates for adjustment; and different strategies for dealing with missing data. Application of multiple methods generates multiple effect estimates for a specific outcome measurement. If multiple estimates are generated but only one or a subset is reported on the basis of the results (e.g. statistical significance), there is a high risk of bias in the fully reported result. Attention should be restricted to analyses that are eligible for consideration by the RoB 2 tool user. For example, if only the result from an	Y/PY/PN/N/NI

	analysis of post-intervention values is eligible for inclusion in a meta-analysis (e.g. at 12 weeks after randomization), and this is reported by the trial, then there would not be an issue of selection even if this result was reported (on the basis of the results) in preference to the result from an analysis of changes from baseline.<br>Answer ‘Yes’ or ‘Probably yes’ if:	
	There is clear evidence (usually through examination of a trial protocol or statistical analysis plan) that a measurement was analysed in multiple eligible ways, but data for only one or a subset of analyses is fully reported (without justification), and the fully reported result is likely to have been selected on the basis of the results. Selection on the basis of the results arises from a desire for findings to be newsworthy, sufficiently noteworthy to merit publication, or to confirm a prior hypothesis. For example, trialists who have a preconception or vested interest in showing that an experimental intervention is beneficial may be inclined to selectively report analyses that are favourable to the experimental intervention.<br>Answer ‘No’ or ‘Probably no’ if:<br>There is clear evidence (usually through examination of a trial protocol or statistical analysis plan) that all eligible reported results for the outcome measurement correspond to all intended analyses.<br>or<br>There is only one possible way in which the outcome measurement can be analysed (hence there is no opportunity to select from multiple analyses).<br>or<br>Analyses are inconsistent across different reports on the same trial, but the trialists have provided the reason for the inconsistency and it is not related to the nature of the results.<br>Answer ‘No information’ if:<br>Analysis intentions are not available, or the analysis intentions are not reported in sufficient detail to enable an assessment, and there is more than one way in which the outcome measurement could have been analysed.	
**Risk-of-bias judgement**	See algorithm.	Low / High / Some concerns

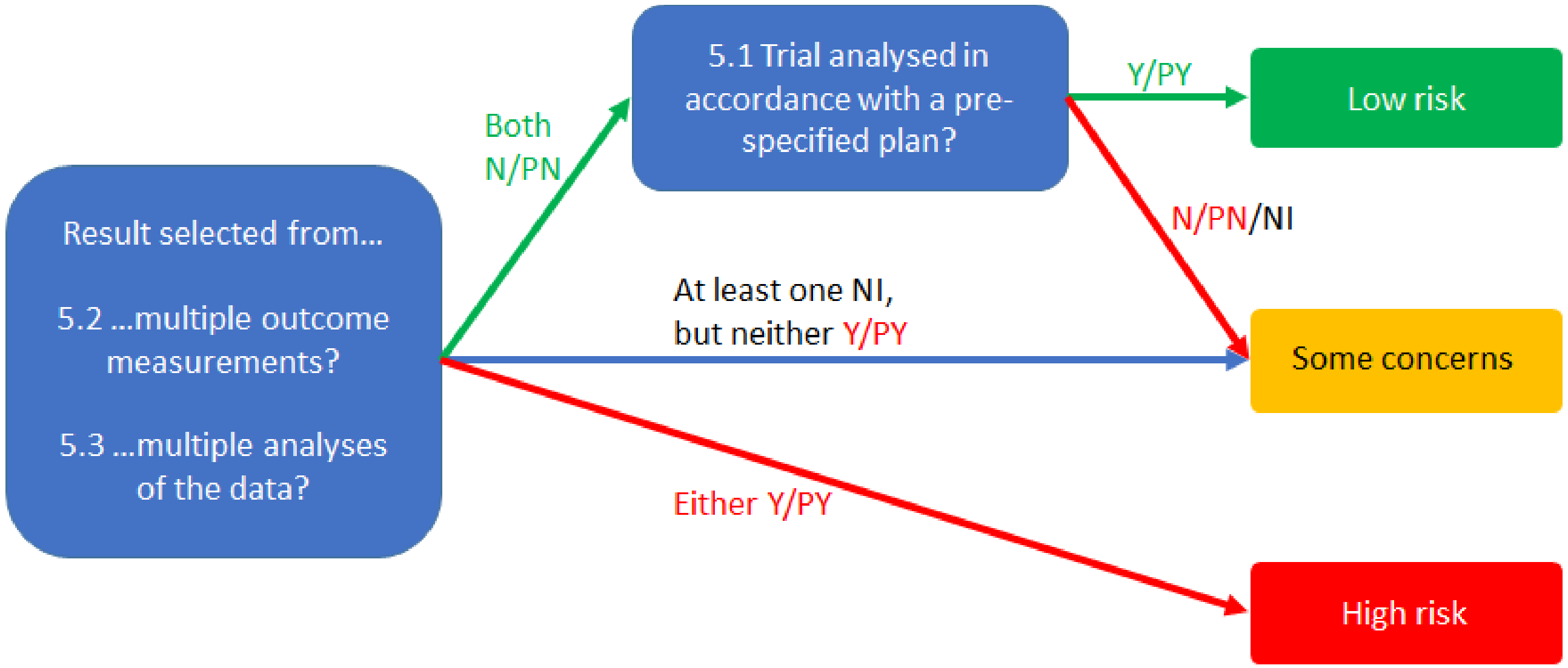

Algorithm for suggested judgement of risk of bias in selection of the reported result

Supplemental Data 11 – List of components

One or more of the treatment components		One of delivery components
education	+	digital
psychological		
exercise		
usual care		in-person
physiotherapy		
placebo		
no intervention		
waiting list		

Grey marked components can only be used as a component in the comparator group and thus will not be linked to a delivery component. Exercise can be classified by delivery component "home" "digital" and "in-person".

Supplemental Data 12 – Excluded studies at full text screening

Supplemental Table 4 – Excluded studies at full text screening

Covidence ID	Author year	Title	Journal	DOI	Reason for exclusion
#389	Fanuscu 2023	THE EFFECTS OF SPINAL STABILIZATION EXERCISES VIA TELEREHABILITATION ON INDIVIDUALS WITH CHRONIC LOW BACK PAIN: A RANDOMIZED CONTROLLED STUDY	Annals of the Rheumatic Diseases	https://dx.doi.org/10.1136/annrheumdis-2023-eular.2467	Conference abstract
#433	Domenech 2018	Cognitive behavioural therapy supported with information and communication technologies in the treatment of chronic low back pain: A randomized clinical trial	European Spine Journal	https://dx.doi.org/10.1007/s00586-018-5691-0	Conference abstract
#436	DomenechFernendez 2017	Information and communication technologies-supported cognitive behavioural therapy in the treatment of chronic low back pain: Randomized clinical trial	European Spine Journal	https://dx.doi.org/10.1007/s00586-017-5270-9	Conference abstract
#450	Slater 2012	Six-month follow-up of a telehealth intervention for chronic back pain	Journal of Pain	https://dx.doi.org/10.1016/j.jpain.2012.01.401	Conference abstract
#453	Chiauzzi 2010	PainACTION.com: An interactive self-management website for chronic back pain patients	Journal of Pain	https://dx.doi.org/10.1016/j.jpain.2010.01.231	Conference abstract
#658	Mbada 2017	Comparative efficacy of clinic-based and telerehabilitation application of mckenzie therapy in low-back pain		10.1016/j.apmr.2017.08.143	Conference abstract
#83	Weise 2023	Correction: The Effect of an App-Based Home Exercise Program on Self-reported Pain Intensity in Unspecific and Degenerative Back Pain: Pragmatic Open-label Randomized Controlled Trial.	J Med Internet Res	10.2196/46512	Correction (content not relevant)

#877	Özden 2022	Correction to: The effect of video exerciseâ€‘based telerehabilitation on clinical outcomes, expectation, satisfaction, and motivation in patients with chronic low back pain	Ir J Med Sci	10.1007/s11845-021-02797-8	Correction (content not relevant)
#736	Guhl 2019	Telemedicine: can In-Person Pre-treatment Communication be Expanded by Video Consultation?		10.1007/s00270-019-02337-z	Grey literature
#873		Internet intervention proves beneficial, according to randomized controlled trial.	Back Letter		Grey literature
#38	Özden 2024	The effect of visual feedback-based clinical monitoring application in patients with chronic low back pain: a randomized controlled trial.	Eur Spine J	10.1007/s00586-023-08098-1	Wrong intervention
#81	VandeWinckel 2023	Identifying body awareness-related brain network changes after Spring Forest Qigongâ„¢ practice or P.Volve low-intensity exercise in adults with chronic low back pain: a feasibility Phase I Randomized Clinical Trial.	medRxiv	10.1101/2023.02.11.23285808	Wrong intervention
#108	JonesSMW 2022	An experimental study to inform adoption of mindfulness-based stress reduction in chronic low back pain.	Implement Sci Commun	10.1186/s43058-022-00335-w	Wrong intervention
#186	Cimarras-Otal 2020	Adapted exercises versus general exercise recommendations on chronic low back pain in industrial workers: A randomized control pilot study.	Work	10.3233/WOR-203322	Wrong intervention
#231	Shebib 2019	Randomized controlled trial of a 12-week digital care program in improving low back pain.	NPJ Digit Med	10.1038/s41746-018-0076-7	Wrong intervention
#267	BaroneGibbs 2018	Reducing sedentary behaviour to decrease chronic low back pain: the stand back randomised trial.	Occup Environ Med	10.1136/oemed-2017-104732	Wrong intervention
#307	Diers 2016	Watching your pain site reduces pain intensity in chronic back pain patients.	Eur J Pain	10.1002/ejp.765	Wrong intervention
#729	Bartholomaeus 2019	Positive Aging: the Impact of a Community Wellbeing and Resilience Program		10.1080/07317115.2018.1561582	Wrong intervention
#919	Alp 2014	Efficacy of Core-Stabilization Exercise and Its Comparison with Home-Based	Turk J Phys Med Rehab	10.5152/tftrd.2014.26817	Wrong intervention

		Conventional Exercise in Low Back Pain Patients			
#948	Dadarkhah 2020	Remote Versus in-Person Exercise Instruction for Chronic Nonspecific Low Back Pain Lasting 12 Weeks or Longer: A Randomized Clinical Trial	Journal of the National Medical Association	10.1016/j.jnma.2020.11.016	Wrong intervention
#113	HoKKN 2022	Efficacy of a digital cognitive behavioral therapy for insomnia in people with low back pain: a feasibility randomized co-twin and singleton-controlled trial.	Pilot Feasibility Stud	10.1186/s40814-022-01087-z	Wrong outcomes
#122	Paganini 2022	Internet- and mobile-based intervention for depression in adults with chronic back pain: A health economic evaluation.	J Affect Disord	10.1016/j.jad.2022.04.004	Wrong outcomes
#147	Rhon 2021	The influence of a MOBile-based video Instruction for Low back pain (MOBIL) on initial care decisions made by primary care providers: a randomized controlled trial.	BMC Fam Pract	10.1186/s12875-021-01549-y	Wrong outcomes
#316	Weymann 2015	Effectiveness of a Web-based tailored interactive health communication application for patients with type 2 diabetes or chronic low back pain: randomized controlled trial.	J Med Internet Res	10.2196/jmir.3904	Wrong outcomes
#332	delPozo-Cruz 2013	Reanalysis of a tailored web-based exercise programme for office workers with sub-acute low back pain: assessing the stage of change in behaviour.	Psychol Health Med	10.1080/13548506.2013.765019	Wrong outcomes
#62	Villatoro-Luque 2023	Telerehabilitation for the treatment in chronic low back pain: A randomized controlled trial.	J Telemed Telecare	10.1177/1357633X231195091	Wrong outcomes
#721	Lorig 2002	Can a Back Pain E-mail Discussion Group improve health status and lower health care costs?: a randomized study		10.1001/archinte.162.7.792	Wrong patient population
#106	Piette 2022	Patient-Centered Pain Care Using Artificial Intelligence and Mobile Health Tools: A Randomized Comparative Effectiveness Trial.	JAMA Intern Med	10.1001/jamainternmed.2022.3178	Wrong patient population
#149	Ashar 2022	Effect of Pain Reprocessing Therapy vs Placebo and Usual Care for Patients With Chronic Back Pain: A Randomized Clinical	JAMA Psychiatry	10.1001/jamapsychiatry.2021.2669	Wrong patient population

		Trial.			
#218	Suman 2019	Effectiveness and cost-utility of a multifaceted eHealth strategy to improve back pain beliefs of patients with non-specific low back pain: a cluster randomised trial.	BMJ Open	10.1136/bmjopen-2019-030879	Wrong patient population
#269	Hüppe 2019	[Evaluation of a Proactive Health Programme for Insured Persons with Persistent Back Pain: One-year Follow-up of a Randomised Controlled Trial].	Gesundheitswesen	10.1055/s-0043-121696	Wrong patient population
#321	Riva 2014	Interactive sections of an Internet-based intervention increase empowerment of chronic back pain patients: randomized controlled trial.	J Med Internet Res	10.2196/jmir.3474	Wrong patient population
#326	Moessner 2014	[Efficacy of an internet-delivered aftercare program for patients with chronic back pain].	Psychother Psychosom Med Psychol	10.1055/s-0033-1351266	Wrong patient population
#897	Priebe 2020	Digital Treatment of Back Pain versus Standard of Care: The Cluster-Randomized Controlled Trial, Rise-uP	JPR	10.2147/JPR.S260761	Wrong patient population
#946	Ayensa 2023	Online-Schmerzedukation verstÃ¤rkt die positiven Auswirkungen eines Online-Trainingsprogramms gegen RÃ¼ckenschmerzen	B&G Bewegungstherapie und Gesundheitssport	10.1055/a-2107-7402	Wrong patient population
#954	Withers 2024	Remotely delivered physiotherapy is as effective as face-to-face physiotherapy for musculoskeletal conditions (REFORM): a randomised trial	Journal of Physiotherapy	10.1016/j.jphys.2024.02.016	Wrong patient population
#7	Dagenais 2021	Economic Evaluation of an Extended Telehealth Worksite Exercise Intervention to Reduce Lost Work Time from Low Back Pain in Career Firefighters.	Journal of Occupational Rehabilitation	10.1007/s10926-020-09933-8	Wrong patient population
#21	Espin 2024	Videoconference-Supervised Group Exercise Reduces Low Back Pain in Eldercare Workers: Results from the ReViEEW Randomised Controlled Trial.	J Occup Rehabil	10.1007/s10926-024-10182-2	Wrong patient population
#68	Marcuzzi 2023	Effect of an Artificial Intelligence-Based Self-Management App on Musculoskeletal Health in Patients With Neck and/or Low	JAMA Netw Open	10.1001/jamanetworkopen.2023.20400	Wrong patient population

		Back Pain Referred to Specialist Care: A Randomized Clinical Trial.			
#100	Weise 2022	The Effect of an App-Based Home Exercise Program on Self-reported Pain Intensity in Unspecific and Degenerative Back Pain: Pragmatic Open-label Randomized Controlled Trial.	J Med Internet Res	10.2196/41899	Wrong patient population
#105	Moshe 2022	Predictors of Dropout in a Digital Intervention for the Prevention and Treatment of Depression in Patients With Chronic Back Pain: Secondary Analysis of Two Randomized Controlled Trials.	J Med Internet Res	10.2196/38261	Wrong patient population
#150	Anan 2021	Effects of an Artificial Intelligence-Assisted Health Program on Workers With Neck/Shoulder Pain/Stiffness and Low Back Pain: Randomized Controlled Trial.	JMIR Mhealth Uhealth	10.2196/27535	Wrong patient population
#180	Baumeister 2021	Effectiveness of a Guided Internet- and Mobile-Based Intervention for Patients with Chronic Back Pain and Depression (WARD-BP): A Multicenter, Pragmatic Randomized Controlled Trial.	Psychother Psychosom	10.1159/000511881	Wrong patient population
#202	Sander 2020	Effectiveness of a Guided Web-Based Self-help Intervention to Prevent Depression in Patients With Persistent Back Pain: The PROD-BP Randomized Clinical Trial.	JAMA Psychiatry	10.1001/jamapsychiatry.2020.1021	Wrong patient population
#223	Hüppe 2019	Feasibility and long-term efficacy of a proactive health program in the treatment of chronic back pain: a randomized controlled trial.	BMC Health Serv Res	10.1186/s12913-019-4561-8	Wrong patient population
#247	Gannon 2019	Telehealth Therapy Effects of Nurses and Mental Health Professionals From 2 Randomized Controlled Trials for Chronic Back Pain.	Clin J Pain	10.1097/AJP.0000000000000678	Wrong patient population
#261	Malfliet 2018	Patients With Chronic Spinal Pain Benefit From Pain Neuroscience Education Regardless the Self-Reported Signs of Central Sensitization: Secondary Analysis of a Randomized Controlled Multicenter Trial.	PM R	10.1016/j.pmrj.2018.04.010	Wrong patient population
#263	Rutledge 2018	Randomized Controlled Trial of Nurse-	J Pain	10.1016/j.jpain.2018.03.017	Wrong patient population

		Delivered Cognitive-Behavioral Therapy Versus Supportive Psychotherapy Telehealth Interventions for Chronic Back Pain.			
#264	Malfliet 2018	Blended-Learning Pain Neuroscience Education for People With Chronic Spinal Pain: Randomized Controlled Multicenter Trial.	Phys Ther	10.1093/ptj/pzx092	Wrong patient population
#312	Wilson 2015	Empowering Patients with Persistent Pain Using an Internet-based Self-Management Program.	Pain Manag Nurs	10.1016/j.pmn.2014.09.009	Wrong patient population
#317	Irvine 2015	Mobile-Web app to self-manage low back pain: randomized controlled trial.	J Med Internet Res	10.2196/jmir.3130	Wrong patient population
#338	Moessner 2012	Internet-based aftercare for patients with back pain-a pilot study.	Telemed J E Health	10.1089/tmj.2011.0221	Wrong patient population
#348	Buhrman 2011	Guided internet-based cognitive behavioural treatment for chronic back pain reduces pain catastrophizing: a randomized controlled trial.	J Rehabil Med	10.2340/16501977-0805	Wrong patient population
#359	Buhrman 2004	Controlled trial of Internet-based treatment with telephone support for chronic back pain.	Pain	10.1016/j.pain.2004.07.021	Wrong patient population
#392	Harrer 2023	Predicting heterogeneous treatment effects of an Internet-based depression intervention for patients with chronic back pain: Secondary analysis of two randomized controlled trials	Internet Interventions	https://dx.doi.org/10.1016/j.invent.2023.100634	Wrong patient population
#417	Schlicker 2020	A web- And mobile-based intervention for comorbid, recurrent depression in patients with chronic back pain on sick leave (get.back): Pilot randomized controlled trial on feasibility, user satisfaction, and effectiveness	JMIR Mental Health	https://dx.doi.org/10.2196/16398	Wrong patient population
#419	Li 2020	An online pain education program for working adults: Pilot randomized controlled trial	Journal of Medical Internet Research	https://dx.doi.org/10.2196/15071	Wrong patient population
#558	Rutledge 2018	Randomized Controlled Trial of Telephone-delivered Cognitive Behavioral Therapy Versus Supportive Care for Chronic Back		10.1097/AJP.0000000000000555	Wrong patient population

		Pain			
#709	Ziadni 2021	Efficacy of a Single-Session "Empowered Relief" Zoom-Delivered Group Intervention for Chronic Pain: randomized Controlled Trial Conducted During the COVID-19 Pandemic		10.2196/29672	Wrong patient population
#892	Iles 2011	Telephone coaching can increase activity levels for people with non-chronic low back pain: a randomised trial	Journal of Physiotherapy	10.1016/S1836-9553(11)70053-4	Wrong patient population
#908	Buhrman 2013	Guided Internetâ€ delivered cognitive behavioural therapy for chronic pain patients who have residual symptoms after rehabilitation treatment: Randomized controlled trial	European Journal of Pain	10.1002/j.1532-2149.2012.00244.x	Wrong patient population
#909	Buhrman 2013	Guided internet-delivered acceptance and commitment therapy for chronic pain patients: A randomized controlled trial	Behaviour Research and Therapy	10.1016/j.brat.2013.02.010	Wrong patient population
#910	Paganini 2019	A guided and unguided internet- and mobile-based intervention for chronic pain: health economic evaluation alongside a randomised controlled trial	BMJ Open	10.1136/bmjopen-2018-023390	Wrong patient population
#911	Calner 2017	Effects of a selfâ€ guided, webâ€ based activity programme for patients with persistent musculoskeletal pain in primary healthcare: A randomized controlled trial	European Journal of Pain	10.1002/ejp.1012	Wrong patient population
#912	Johnston 2010	The Effectiveness of an Acceptance and Commitment Therapy Self-help Intervention for Chronic Pain	The Clinical Journal of Pain	10.1097/AJP.0b013e3181cf59ce	Wrong patient population
#913	Leveille 2009	Health Coaching via an Internet Portal for Primary Care Patients With Chronic Conditions: A Randomized Controlled Trial	Medical Care	10.1097/MLR.0b013e3181844dd0	Wrong patient population
#914	Ruehlman 2012	A randomized controlled evaluation of an online chronic pain self management program	Pain	10.1016/j.pain.2011.10.025	Wrong patient population
#915	Dear 2013	The Pain Course : A randomised controlled trial of a clinician-guided Internet-delivered cognitive behaviour therapy program for managing chronic pain and emotional well-being	Pain	10.1016/j.pain.2013.03.005	Wrong patient population

#916	Naylor 2008	Therapeutic Interactive Voice Response for chronic pain reduction and relapse prevention	Pain	10.1016/j.pain.2007.11.001	Wrong patient population
#918	Hauser-Ulrich 2020	A Smartphone-Based Health Care Chatbot to Promote Self-Management of Chronic Pain (SELMA): Pilot Randomized Controlled Trial	JMIR Mhealth Uhealth	10.2196/15806	Wrong patient population
#921	Berman 2009	The Effectiveness of an Online Mind-Body Intervention for Older Adults With Chronic Pain	The Journal of Pain	10.1016/j.jpain.2008.07.006	Wrong patient population
#922	Boselie 2018	Filling the glass: Effects of a positive psychology intervention on executive task performance in chronic pain patients	European Journal of Pain	10.1002/ejp.1214	Wrong patient population
#923	DeBoer 2014	A randomized controlled trial of an I internetâ€ based cognitiveâ€“behavioural intervention for nonâ€ specific chronic pain: An effectiveness and costâ€ effectiveness study	European Journal of Pain	10.1002/ejp.509	Wrong patient population
#924	Dear 2015	The Pain Course: a randomised controlled trial examining an internet-delivered pain management program when provided with different levels of clinician support	Pain	10.1097/j.pain.0000000000000251	Wrong patient population
#925	Gardner-Nix 2008	Evaluating distance education of a mindfulness-based meditation programme for chronic pain management	J Telemed Telecare	10.1258/jtt.2007.070811	Wrong patient population
#927	Herbert 2017	Telehealth Versus In-Person Acceptance and Commitment Therapy for Chronic Pain: A Randomized Noninferiority Trial	The Journal of Pain	10.1016/j.jpain.2016.10.014	Wrong patient population
#928	Lin 2017	An Internet-Based Intervention for Chronic Pain	Dtsch Arztebl Int	10.3238/arztebl.2017.0681	Wrong patient population
#929	Peters 2017	Happy Despite Pain: A Randomized Controlled Trial of an 8-Week Internet-delivered Positive Psychology Intervention for Enhancing Well-being in Patients With Chronic Pain	The Clinical Journal of Pain	10.1097/AJP.0000000000000494	Wrong patient population
#930	Rickardsson 2021	Internetâ€ delivered acceptance and commitment therapy as microlearning for chronic pain: A randomized controlled trial	European Journal of Pain	10.1002/ejp.1723	Wrong patient population

		with 1â€‹ year followâ€‹ up			
#933	Scott 2018	Feasibility randomizedâ€‹ controlled trial of online Acceptance and Commitment Therapy for patients with complex chronic pain in the United Kingdom	European Journal of Pain	10.1002/ejp.1236	Wrong patient population
#934	Smith 2019	Reboot Online: A Randomized Controlled Trial Comparing an Online Multidisciplinary Pain Management Program with Usual Care for Chronic Pain	Pain Medicine	10.1093/pm/pnz208	Wrong patient population
#935	Trompetter 2015	Internet-based guided self-help intervention for chronic pain based on Acceptance and Commitment Therapy: A randomized controlled trial	J Behav Med	10.1007/s10865-014-9579-0	Wrong patient population
#938	Nordin 2016	Effects of the Web Behavior Change Program for Activity and Multimodal Pain Rehabilitation: Randomized Controlled Trial	J Med Internet Res	10.2196/jmir.5634	Wrong patient population
#939	Henriksson 2016	Effects of Eight-Week-Web-Based Mindfulness Training on Pain Intensity, Pain Acceptance, and Life Satisfaction in Individuals With Chronic Pain	Psychol Rep	10.1177/0033294116675086	Wrong patient population
#940	Guillory 2015	Piloting a Text Messageâ€“based Social Support Intervention for Patients With Chronic Pain: Establishing Feasibility and Preliminary Efficacy	The Clinical Journal of Pain	10.1097/AJP.0000000000000193	Wrong patient population
#941	Dowd 2015	Comparison of an Online Mindfulness-based Cognitive Therapy Intervention With Online Pain Management Psychoeducation: A Randomized Controlled Study	The Clinical Journal of Pain	10.1097/AJP.0000000000000201	Wrong patient population
#949	Devorski 2024	Gamified delivery of at-home rehabilitation for individuals with nonspecific low back pain: a randomized controlled trial	Disability and rehabilitation	10.1080/09638288.2024.2368694	Wrong patient population
#281	Heapy 2017	Interactive Voice Response-Based Self-management for Chronic Back Pain: The COPES Noninferiority Randomized Trial.	JAMA Intern Med	10.1001/jamainternmed.2017.0223	Wrong patient population
#932	Schulz 2007	An internet-based approach to enhance self-management of chronic low back pain in the Italian-speaking population of Switzerland: results from a pilot study	Int J Public Health	10.1007/s00038-007-5127-9	Wrong study design
#970	Monreal-Bartolomé	Efficacy of a Blended Low-Intensity	J Med Internet Res	10.2196/56203	Wrong patient population

	2025	Internet-Delivered Psychological Program in Patients With Multimorbidity in Primary Care: Randomized Controlled Trial.			
#992	Albers 2024	Non-inferiority of hybrid outpatient telerehabilitation for patients with back pain: 3-month follow-up of a randomized controlled trial.	Eur J Phys Rehabil Med	10.23736/S1973-9087.24.08458-2	Wrong patient population
#1493	Abdala 2025	Effect of thigh muscle strength training through telerehabilitation in volunteers with low back pain. A controlled and randomized clinical trial	Journal of Bodywork and Movement Therapies	10.1016/j.jbmt.2025.04.016	Wrong outcomes
#1006	Priebe 2024	Medical App Treatment of Non-Specific Low Back Pain in the 12-month Cluster-Randomized Controlled Trial Rise-uP: Where Clinical Superiority Meets Cost Savings.	J Pain Res	10.2147/JPR.S473250	Wrong patient population
#1719	Allworth 2025	Exploring participant satisfaction with an eHealth intervention for low back pain and knee osteoarthritis: enhancing physiotherapy access in rural Australia	Musculoskeletal Science and Practice	10.1016/j.msksp.2024.103252	Wrong outcomes
#1007	Holden 2024	Embedded motivational interviewing combined with a smartphone application to increase physical activity in people with sub-acute low back pain: a cluster randomised controlled trial.	Braz J Phys Ther	10.1016/j.bjpt.2024.101091	Wrong patient population
#1000	Shi 2024	The Physical and Psychological Effects of Telerehabilitation-Based Exercise for Patients With Nonspecific Low Back Pain: Prospective Randomized Controlled Trial.	JMIR Mhealth Uhealth	10.2196/56580	Wrong intervention
#964	Lindner 2025	Digitally supported physical activity counselling for people with chronic back pain: a randomised controlled parallel feasibility study.	BMC Prim Care	10.1186/s12875-025-02742-z	Wrong patient population
#1009	Lovo 2024	Effectiveness of an interprofessional assessment and management approach for people with chronic low back disorders delivered via virtual care: A randomized controlled trial pilot intervention.	Digit Health	10.1177/20552076241260569	Wrong intervention
#1399	Barğı 2024	Effects of short-term upper extremity	IOS Press	10.3233/WOR-230190	Wrong patient population

		exercise training in office workers during COVID-19 restrictions: A randomized controlled trial.			
#996	Son 2024	Efficacy of Self-Natural Posture Exercise (SNPE) programs on chronic low back pain: A randomized controlled feasibility trial with waitlist control.	J Back Musculoskelet Rehabil	10.3233/BMR-230441	Wrong study design
#1361	Park 2024	Assessing a Qigong Intervention for Military Veterans with Chronic Low Back Pain.	Integrative & Complementary Therapies	10.1089/ict.2024.27207.jyp	Wrong intervention

Supplemental Data 13 – Authors contacted for further information

Supplemental Table 5 – Authors contacted for further information

Covidence ID	Author, Year	Manuscript title	Which data/information is needed?	1st contact	2nd contact	3rd contact	Data/answer received?
#222	Petrozzi, 2019	Addition of MoodGYM to physical treatments for chronic low back pain: A randomized controlled trial	Number of adverse events per group	08.05.24	15.05.24		Yes
#149	Ashar, 2022	Effect of Pain Reprocessing Therapy vs Placebo and Usual Care for Patients With Chronic Back Pain: A Randomized Clinical Trial.	Eligibility criteria: low back pain only?	08.05.24			Yes
#321	Riva, 2014	Interactive sections of an Internet-based intervention increase empowerment of chronic back pain patients: randomized controlled trial.	Eligibility criteria: low back pain only?	08.05.24	15.05.24	22.05.24	No
#897	Priebe, 2020	Digital Treatment of Back Pain versus Standard of Care: The Cluster-Randomized Controlled Trial, Rise-uP.	Included sample - pain duration	13.05.24	21.05.24	24.06.24	Yes
#721	Lorig, 2002	Can a Back Pain E-mail Discussion Group improve health status and lower health care costs?: a randomized study	Eligibility criteria: low back pain only? + included sample: pain duration	13.05.24			Yes
#23	Day, 2024	The effects of telehealth-delivered mindfulness meditation, cognitive therapy, and behavioral activation for chronic low back pain: a randomized clinical trial	Baseline NRS and PROMIS physical function; 3 month follow-up = 3 months after baseline or post-treatment?; adverse events per group (new: 21.05.)	16.05.24			Yes

#340	del Pozo-Cruz, 2013	Clinical effects of a nine-month web-based intervention in subacute non-specific low back pain patients: a randomized controlled trial	VAS and ODI raw scores for follow-up	21.05.24	18.06.24		Yes
#21	Espin, 2024	Videoconference-Supervised Group Exercise Reduces Low Back Pain in Eldercare Workers: Results from the ReViEEW Randomised Controlled Trial	Adverse events per participant per group	21.05.24			Yes
#63	Garreta-Catala, 2023	Feasibility of a multidisciplinary group videoconferencing approach for chronic low back pain: a randomized, open-label, controlled, pilot clinical trial (EN-FORMA)	Data for SF-36 physical function and ODI (Fig. 3 &4) as raw data	23.05.24	18.06.24	25.06.24	No
#942	Goode, 2018	Effects of a Home-Based Telephone-Supported Physical Activity Program for Older Adult Veterans With Chronic Low Back Pain	Baseline values for RMDQ all groups	24.05.24	18.06.24	25.06.24	No
#281	Heapy, 2017	Interactive Voice Response–Based Self-management for Chronic Back PainThe COPES Noninferiority Randomized Trial	Seperate values for non-specific low back pain (pain intensity, RMDQ, adverse events)	18.06.24	25.06.24	03.07.24	No
#167	Kazemi, 2020	The effectiveness of social media and in-person interventions for low back pain conditions in nursing personnel (SMILE)	Intervention duration	19.06.24	27.06.24	03.07.24	No
#691	Mesa-Castrillon, 2023	Effectiveness of an eHealth-Delivered Program to Empower People With Musculoskeletal Pain in Rural Australia: A Randomized	Age and N female for low back pain subgroup	20.06.24			Yes

		Controlled Trial					
#147	Rhon, 2021	The influence of a MOBile-based video Instruction for Low back pain (MOBIL) on initial care decisions made by primary care providers: a randomized controlled trial	Raw values baseline and follow-up for PROMIS pain and physical function	21.06.24	28.06.24	05.07.24	No
#880	Sandal, 2021	Effectiveness of App-Delivered, Tailored Self-management Support for Adults With Lower Back Pain–Related DisabilityA selfBACK Randomized Clinical Trial	Did participants have access to the app after 3 months?	21.06.24			Yes
#317	Irvine, 2015	Mobile-Web app to self-manage low back pain: randomized controlled trial.	Were patients without pain included in the pain intensity measure? Which scale was used?	24.06.24			Yes
#218	Suman, 2019	Effectiveness and cost-utility of a multifaceted eHealth strategy to improve back pain beliefs of patients with non-specific low back pain: a cluster randomised trial.	Pain duration of included sample	25.06.24	03.07.24	11.07.24	No
#100	Weise, 2022	The Effect of an App-Based Home Exercise Program on Self-reported Pain Intensity in Unspecific and Degenerative Back Pain: Pragmatic Open-label Randomized Controlled Trial	Pain duration of included sample	25.06.24	03.07.24	11.07.24	No
#708	Kohns, 2020	The Effects of a Pain Psychology and Neuroscience Self-Evaluation Internet Intervention: a Randomized Controlled Trial	Demographics (age, pain duration, N female, N randomized) from sample without fibromyalgia	04.07.24			Yes
#992	Albers,	Non-inferiority of hybrid	Were patients with back pain in general	22.05.20			Yes

2024	outpatient telerehabilitation for patients with back pain: 3-month follow-up of a randomized controlled trial.	included or was it restricted to low back pain?	25

NRS = numeric rating scale, PROMIS = patient reported outcome information system, VAS = visual analogue scale, ODI = Oswestry Disability Index, SF-36 = short form 36 health survey, RMDQ = Roland Morris disability questionnaire

Supplemental Data 14 – Network plots of follow-up timepoints

Disability

>1 week < 3 months

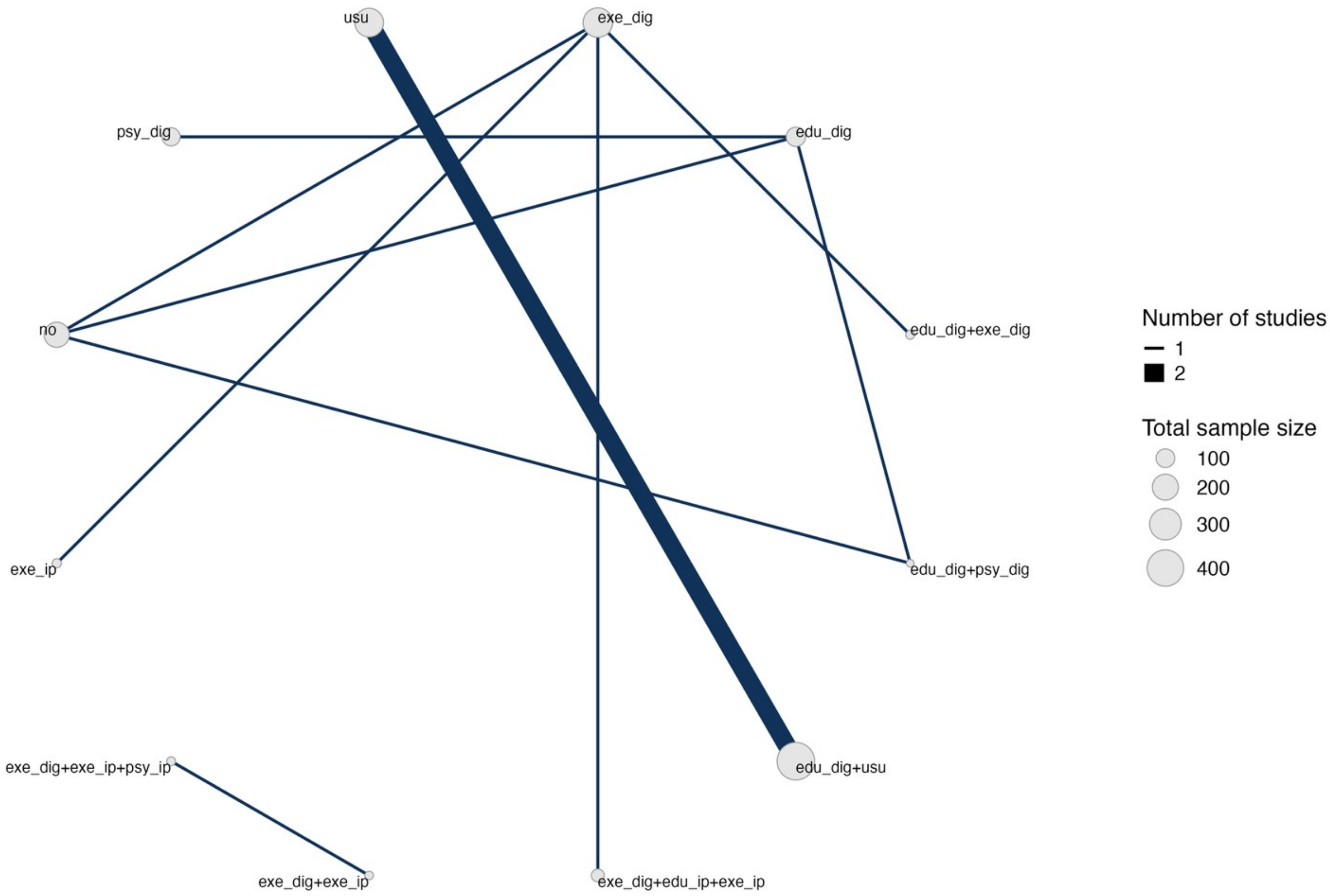

Supplemental Figure 1 - Network plot disability >1 week < 3months. Circles represent the total sample size at the node; thickness of the lines represents the number of studies per contrast; edu = education, exe = exercise, psy = psychological, phy = physiotherapy, usu = usual care, no = no intervention, dig = digital, ip = in-person, home = home program

> 3 months < 1 year

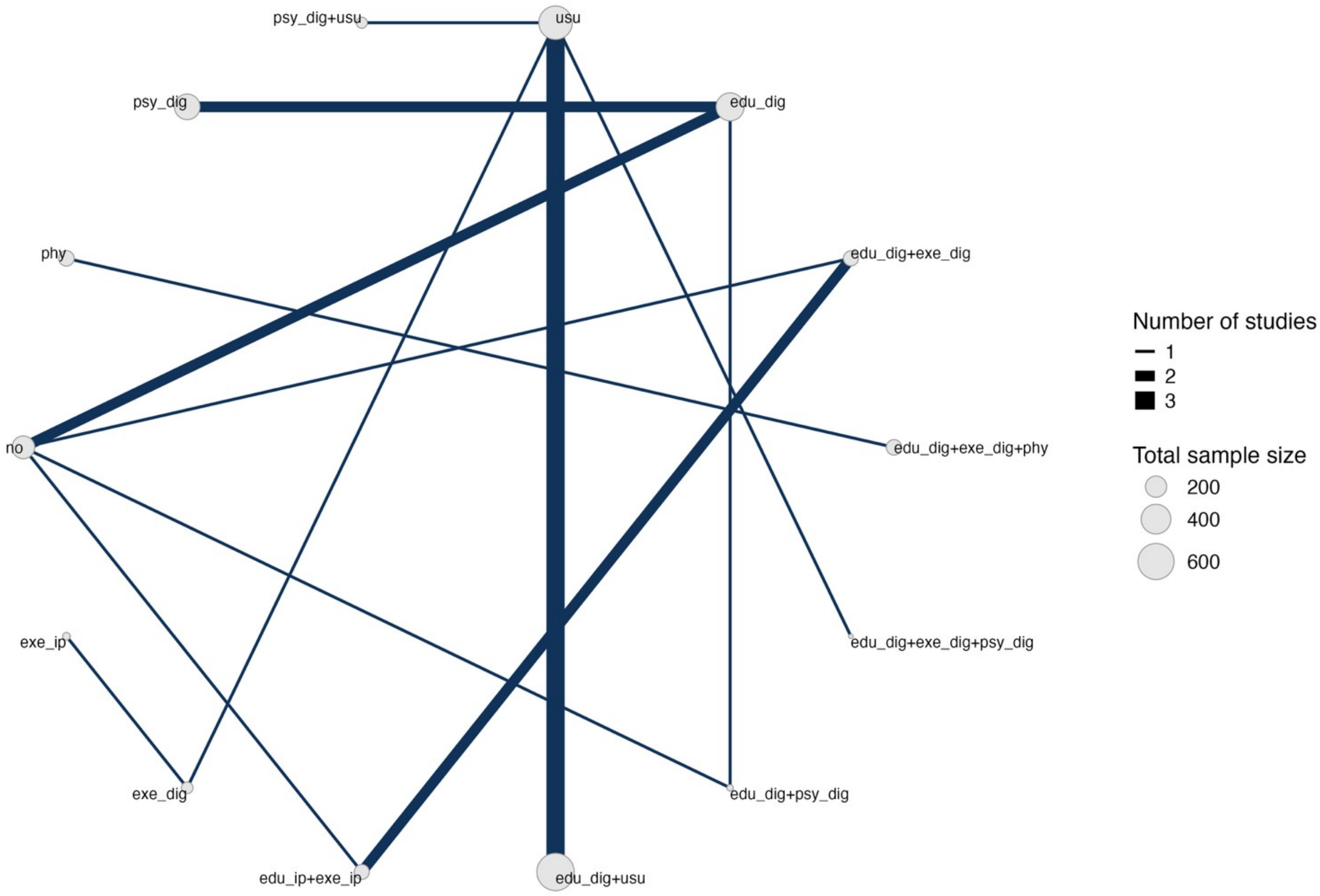

Supplemental Figure 2 - Network plot disability >3 months < 1 year. Circles represent the total sample size at the node; thickness of the lines represents the number of studies per contrast; edu = education, exe = exercise, psy = psychological, phy = physiotherapy, usu = usual care, no = no intervention, dig = digital, ip = in-person, home = home program

Pain intensity

>1 week < 3 months

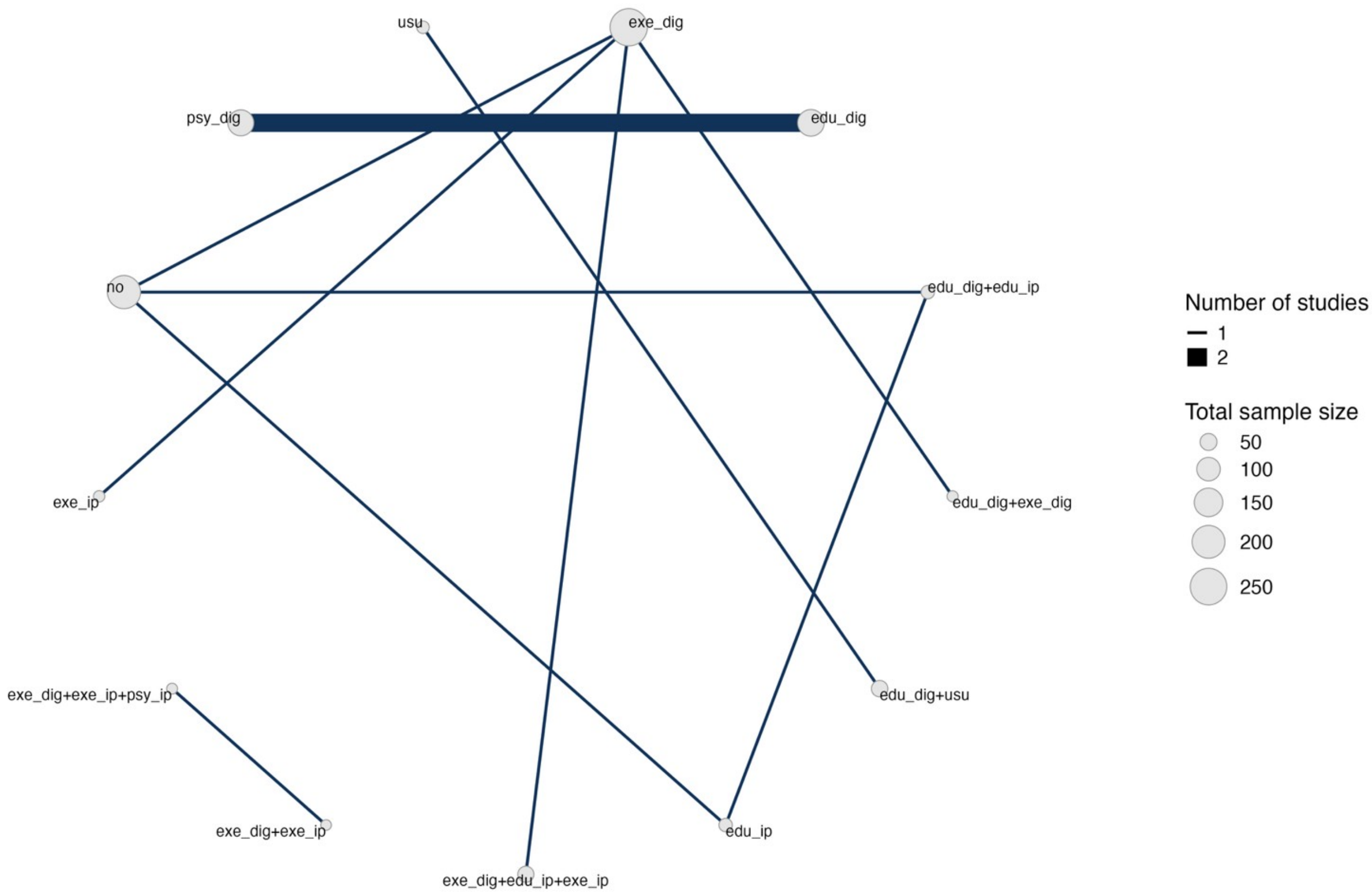

Supplemental Figure 3 - Network plot pain intensity >1 week < 3months. Circles represent the total sample size at the node; thickness of the lines represents the number of studies per contrast; edu = education, exe = exercise, psy = psychological, phy = physiotherapy, usu = usual care, no = no intervention, dig = digital, ip = in-person, home = home program

> 3 months < 1 year

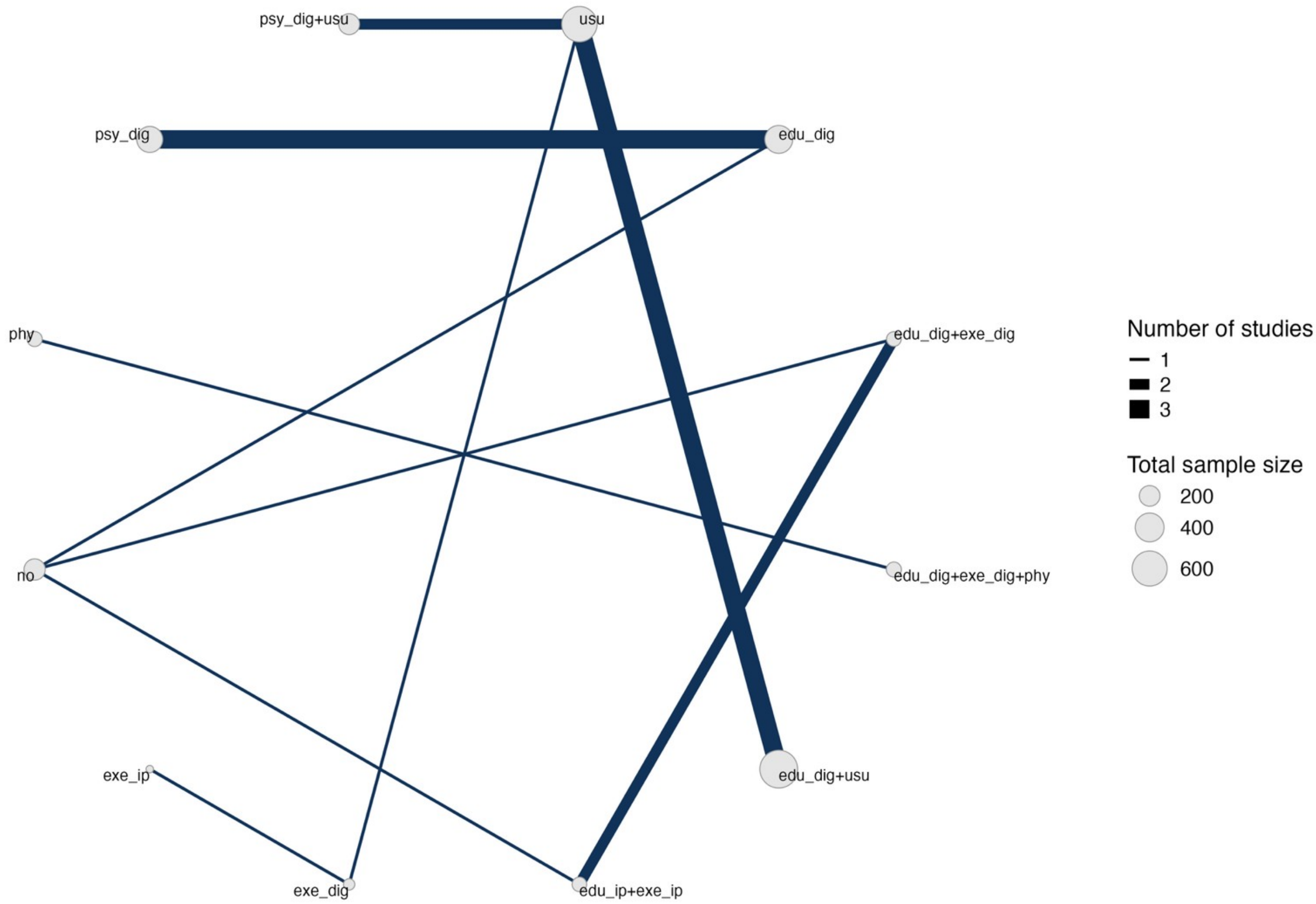

Supplemental Figure 4 - Network plot pain intensity >3 months < 1 year. Circles represent the total sample size at the node; thickness of the lines represents the number of studies per contrast; edu = education, exe = exercise, psy = psychological, phy = physiotherapy, usu = usual care, no = no intervention, dig = digital, ip = in-person, home = home program

Supplemental Data 15 – Studies per node

Disability immediate

Supplemental Table 6 – Studies per node in the disability immediate network

Intervention	N studies per node
education_digital	3
education_digital+education_inperson	1
education_digital+exercise_digital	6
education_digital+exercise_digital+physiotherapy	1
education_digital+exercise_digital+psychological_digital	3
education_digital+exercise_digital+usual_care	3
education_digital+exercise_home	2
education_digital+physiotherapy	1
education_digital+psychological_digital	1
education_digital+psychological_digital+exercise_home	2
education_digital+usual_care	1
education_inperson	1
education_inperson+exercise_inperson	2
education_inperson+exercise_inperson+physiotherapy	1
exercise_digital	12
exercise_digital+education_inperson	1
exercise_digital+exercise_inperson	1
exercise_digital+exercise_inperson+psychological_inperson	1
exercise_digital+physiotherapy	2
exercise_digital+usual_care	1
exercise_home	3
exercise_home+physiotherapy	1
exercise_inperson	5
no_intervention	7
physiotherapy	3
psychological_digital	2
psychological_digital+usual_care	1
usual_care	10

Pain intensity immediate

Supplemental Table 7 – Studies per node in the pain intensity immediate network

Intervention	N studies per node
eduation_digital	4
eduation_digital+eduation_inperson	4
eduation_digital+exercise_digital	6
eduation_digital+exercise_digital+physiotherapy	1
eduation_digital+exercise_digital+psychological_digital	2
eduation_digital+exercise_digital+usual_care	2
eduation_digital+physiotherapy	1
eduation_digital+psychological_digital	1
eduation_inperson	3
eduation_inperson+exercise_inperson	2
eduation_inperson+exercise_inperson+physiotherapy	1
exercise_digital	13
exercise_digital+eduation_inperson	1
exercise_digital+exercise_inperson	1
exercise_digital+exercise_inperson+psychological_inperson	1
exercise_digital+physiotherapy	2
exercise_digital+usual_care	1
exercise_home	3
exercise_home+physiotherapy	1
exercise_inperson	5
no_intervention	7
physiotherapy	5
psychological_digital	2
psychological_digital+usual_care	2
usual_care	9

Supplemental Data 16 – Risk of bias within studies

Traffic plot parallel trials

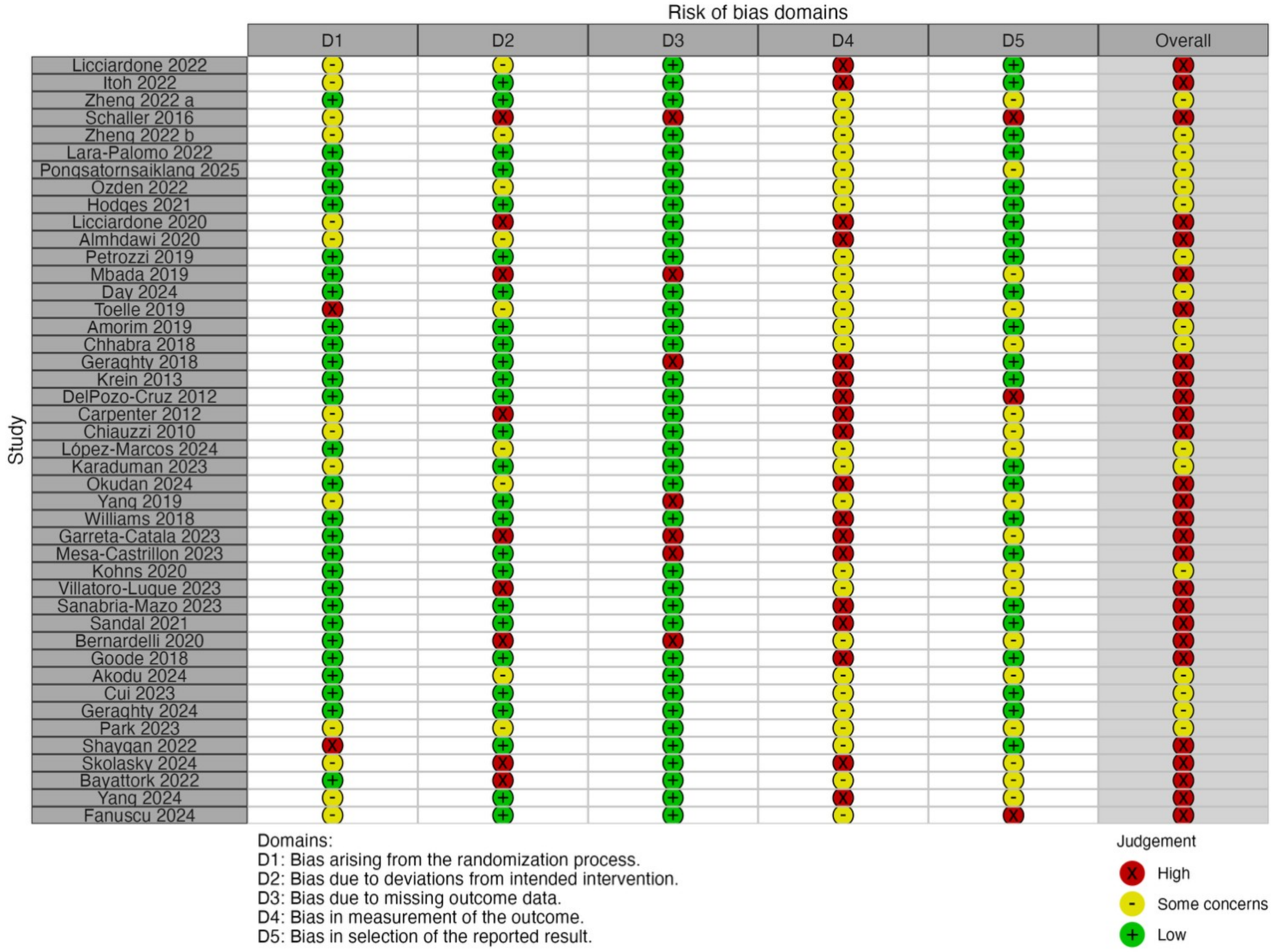

Supplemental Figure 5 – Traffic plot of Risk of Bias 2 ratings for parallel trials

Traffic plot cluster trials

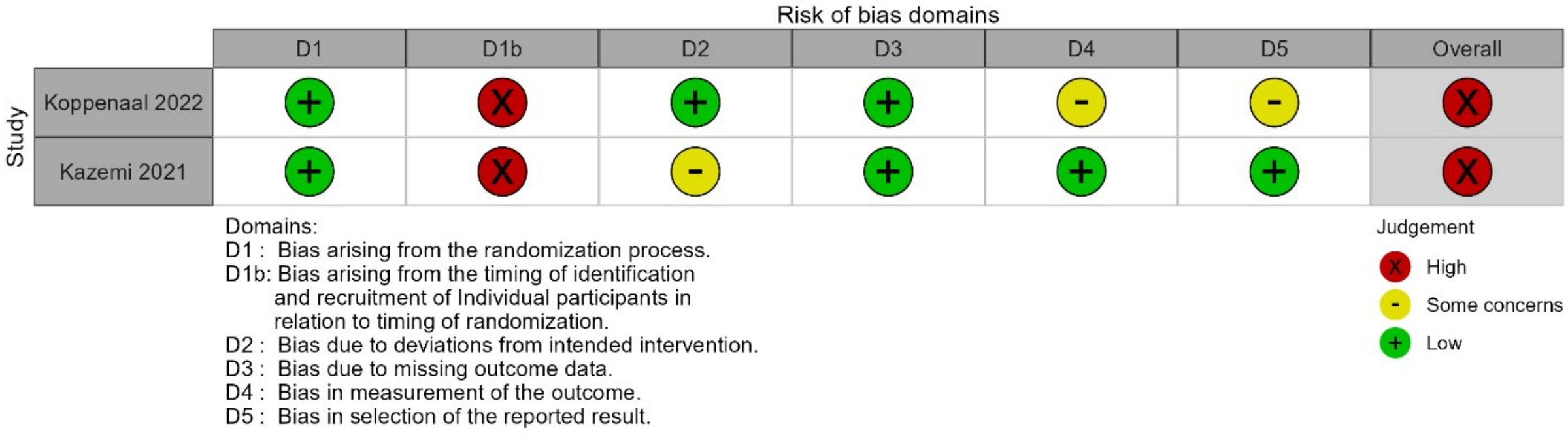

Supplemental Figure 6 - Traffic plot of Risk of Bias 2 ratings for cluster trials

Risk of bias individual ratings

Parallel trials, data stored on OSF

Cluster trials, data stored on OSF

Supplemental Data 17 – Pairwise meta-analyses

Disability immediate

Network 1

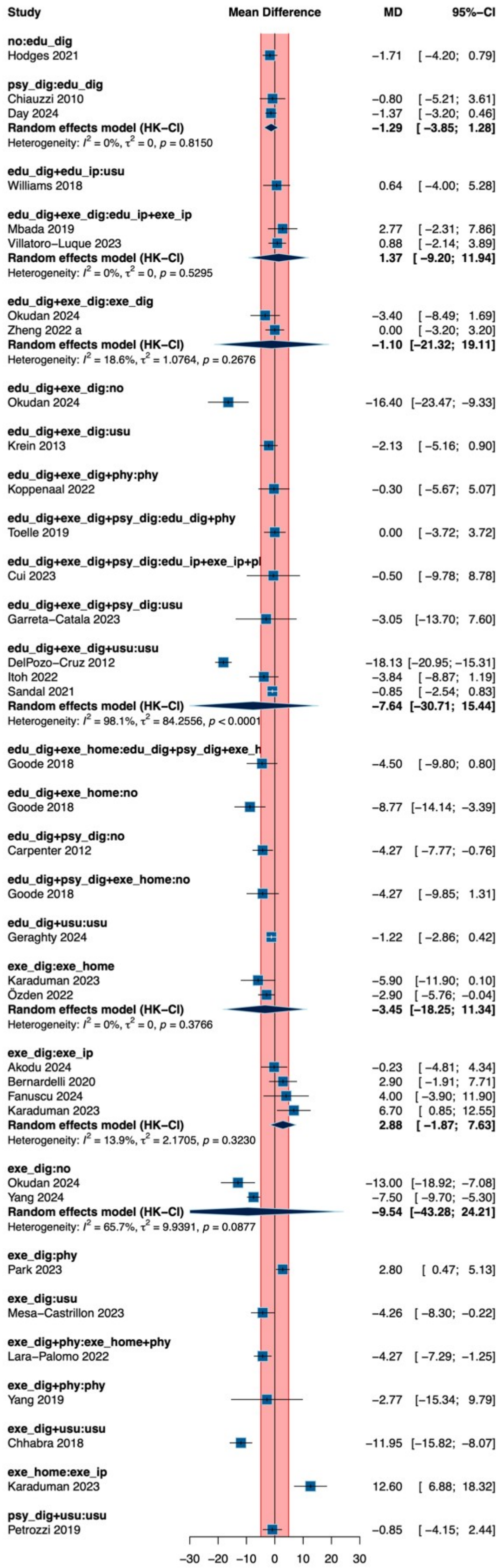

Supplemental Figure 7 - Forest plot of pairwise meta-analyses for network 1 of disability immediate. Red lines represent MCID based on 0.5 SD. Data is backtransformed using the Oswestry Disability Index. edu = education, exe = exercise, psy = psychological, phy = physiotherapy, usu = usual care, no = no intervention, dig = digital, ip = in-person, home = home program

Network 2

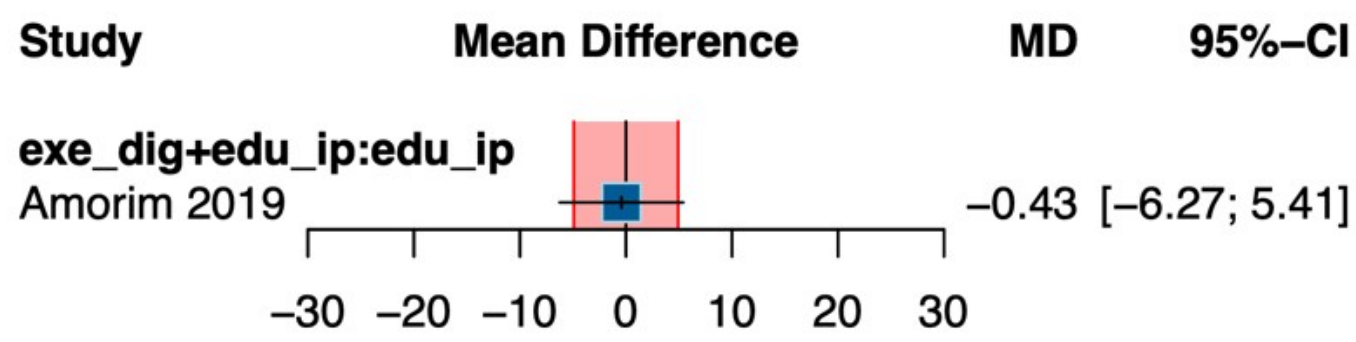

Supplemental Figure 8 - Forest plot of pairwise meta-analyses for network 2 of disability immediate. Red lines represent MCID based on 0.5 SD. Data is backtransformed using the Oswestry Disability Index. edu = education, exe = exercise, psy = psychological, phy = physiotherapy, usu = usual care, no = no intervention, dig = digital, ip = in-person, home = home program

Network 3

Supplemental Figure 9 - Forest plot of pairwise meta-analyses for network 3 of disability immediate. Red lines represent MCID based on 0.5 SD. Data is backtransformed using the Oswestry Disability Index. edu = education, exe = exercise, psy = psychological, phy = physiotherapy, usu = usual care, no = no intervention, dig = digital, ip = in-person, home = home program

Disability >1 week <3 months

Network 1

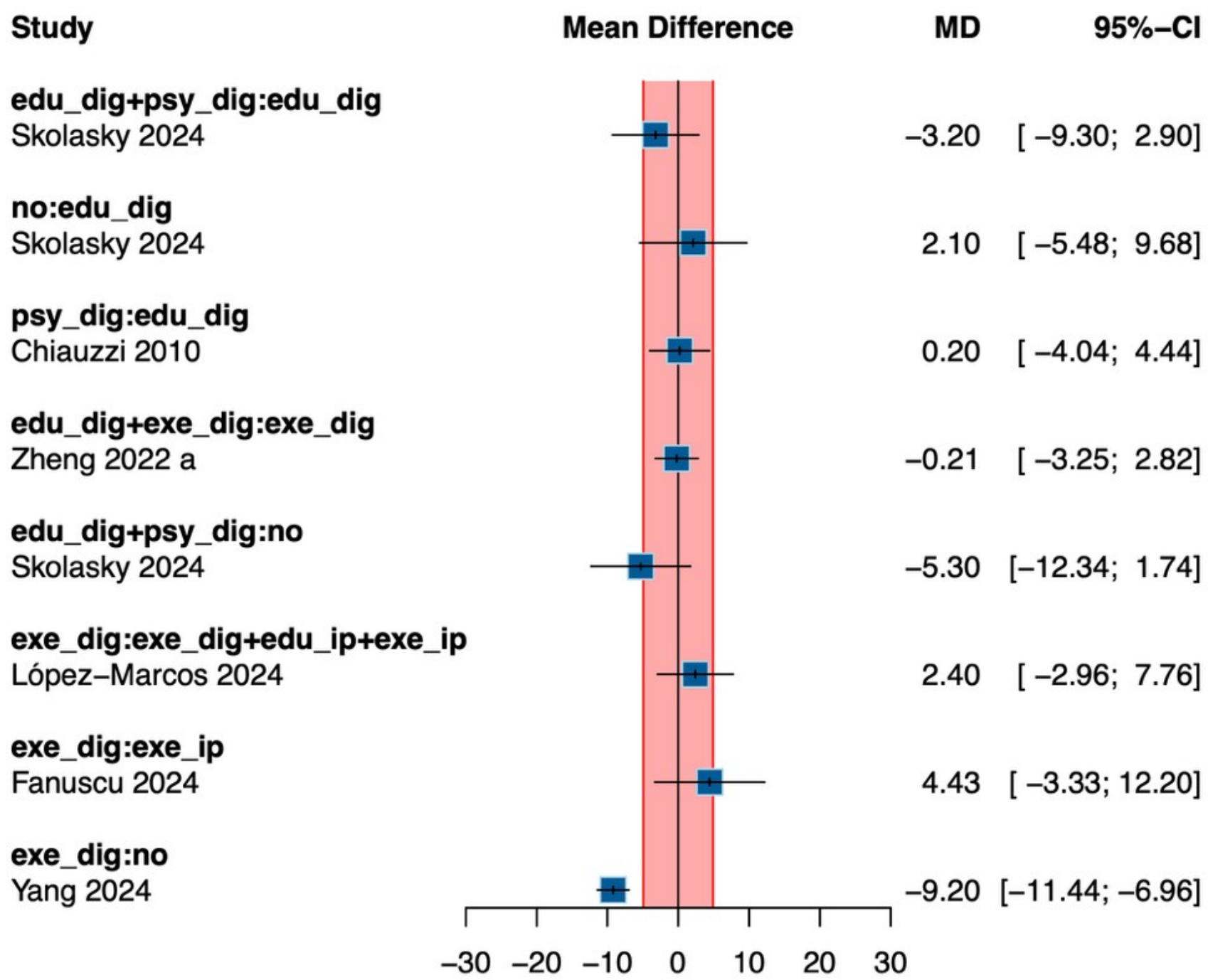

Supplemental Figure 10 - Forest plot of pairwise meta-analyses for network 1 of disability >1 week <3months. Red lines represent MCID based on 0.5 SD. Data is backtransformed using the Oswestry Disability Index. edu = education, exe = exercise, psy = psychological, phy = physiotherapy, usu = usual care, no = no intervention, dig = digital, ip = in-person, home = home program

Network 2

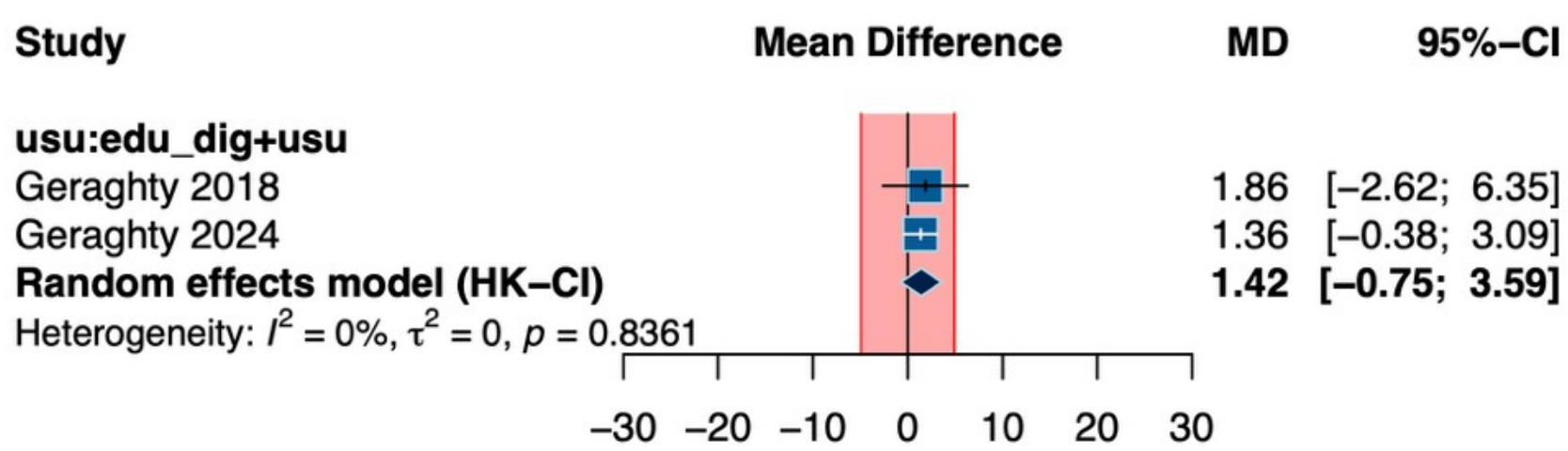

Supplemental Figure 11 - Forest plot of pairwise meta-analyses for network 2 of disability >1 week <3months. Red lines represent MCID based on 0.5 SD. Data is backtransformed using the Oswestry Disability Index. edu = education, exe = exercise, psy = psychological, phy = physiotherapy, usu = usual care, no = no intervention, dig = digital, ip = in-person, home = home program

Network 3

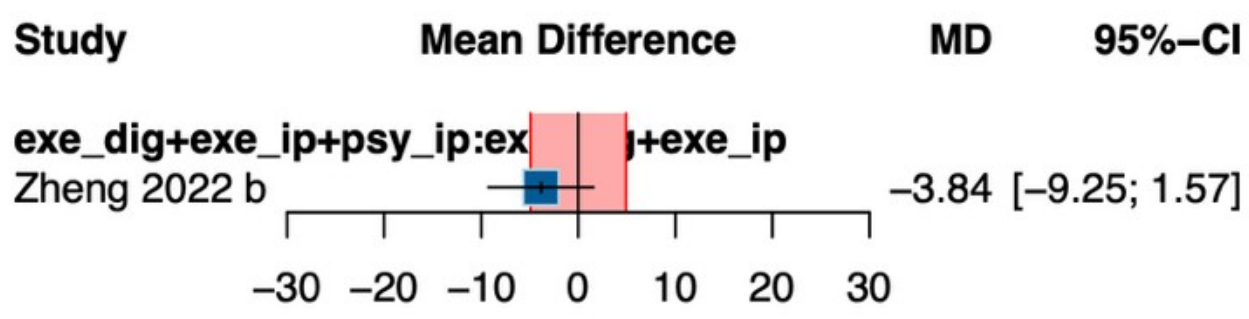

Supplemental Figure 12 - Forest plot of pairwise meta-analyses for network 3 of disability >1 week <3months. Red lines represent MCID based on 0.5 SD. Data is backtransformed using the Oswestry Disability Index. edu = education, exe = exercise, psy = psychological, phy = physiotherapy, usu = usual care, no = no intervention, dig = digital, ip = in-person, home = home program

Disability >3months <1 year

Network 1

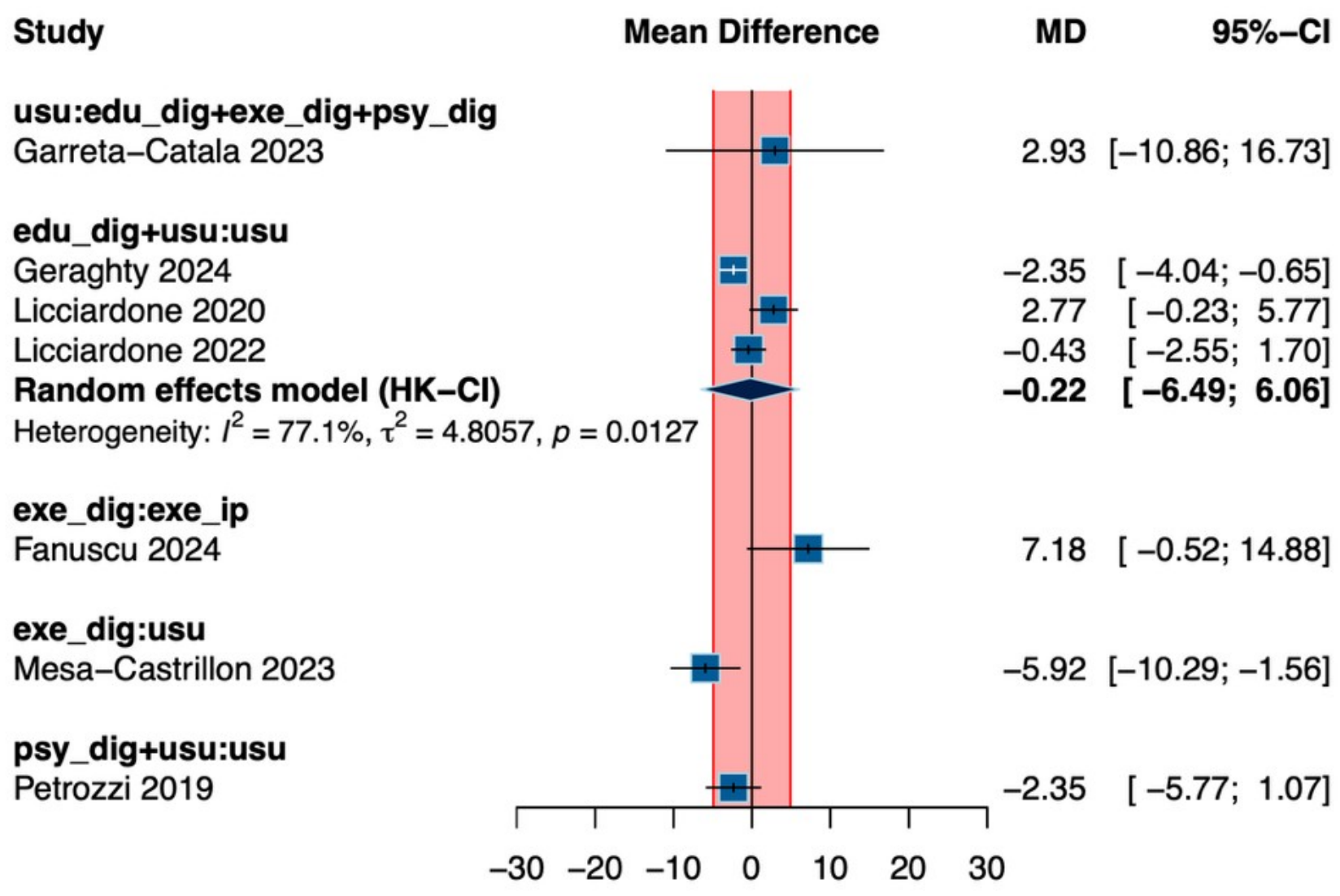

Supplemental Figure 13 - Forest plot of pairwise meta-analyses for network 1 of disability >3 months <1 year. Red lines represent MCID based on 0.5 SD. Data is backtransformed using the Oswestry Disability Index. edu = education, exe = exercise, psy = psychological, phy = physiotherapy, usu = usual care, no = no intervention, dig = digital, ip = in-person, home = home program

Network 2

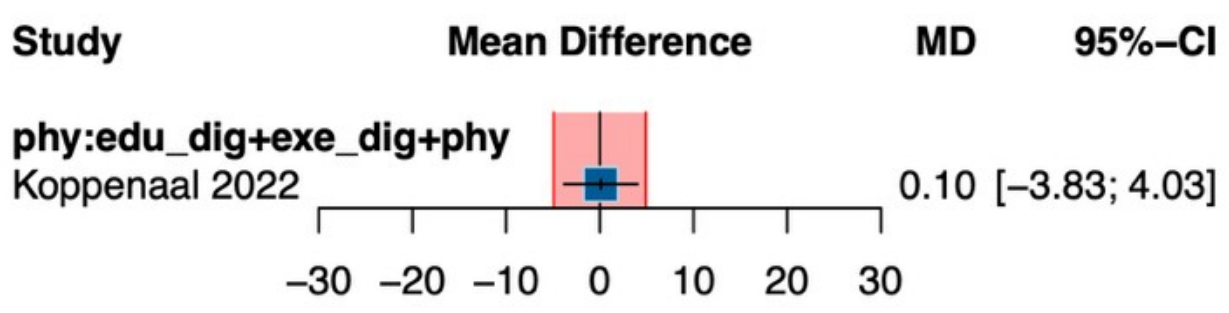

Supplemental Figure 14 - Forest plot of pairwise meta-analyses for network 2 of disability >3 months <1 year. Red lines represent MCID based on 0.5 SD. Data is backtransformed using the Oswestry Disability Index. edu = education, exe = exercise, psy = psychological, phy = physiotherapy, usu = usual care, no = no intervention, dig = digital, ip = in-person, home = home program

Network 3

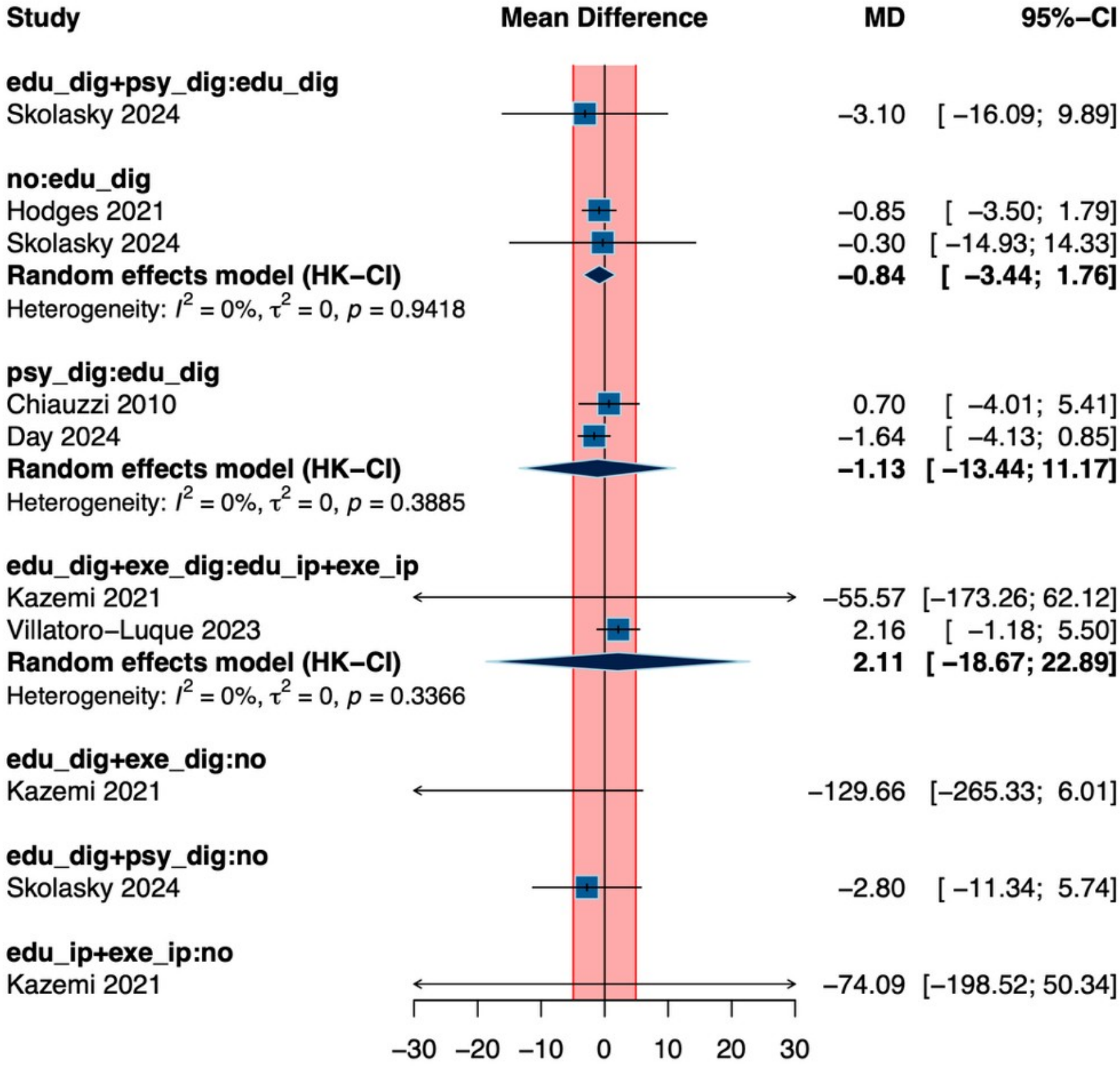

Supplemental Figure 15 - Forest plot of pairwise meta-analyses for network 3 of disability >3 months <1 year. Red lines represent MCID based on 0.5 SD. Data is backtransformed using the Oswestry Disability Index. edu = education, exe = exercise, psy = psychological, phy = physiotherapy, usu = usual care, no = no intervention, dig = digital, ip = in-person, home = home program

Pain intensity immediate

Network 1

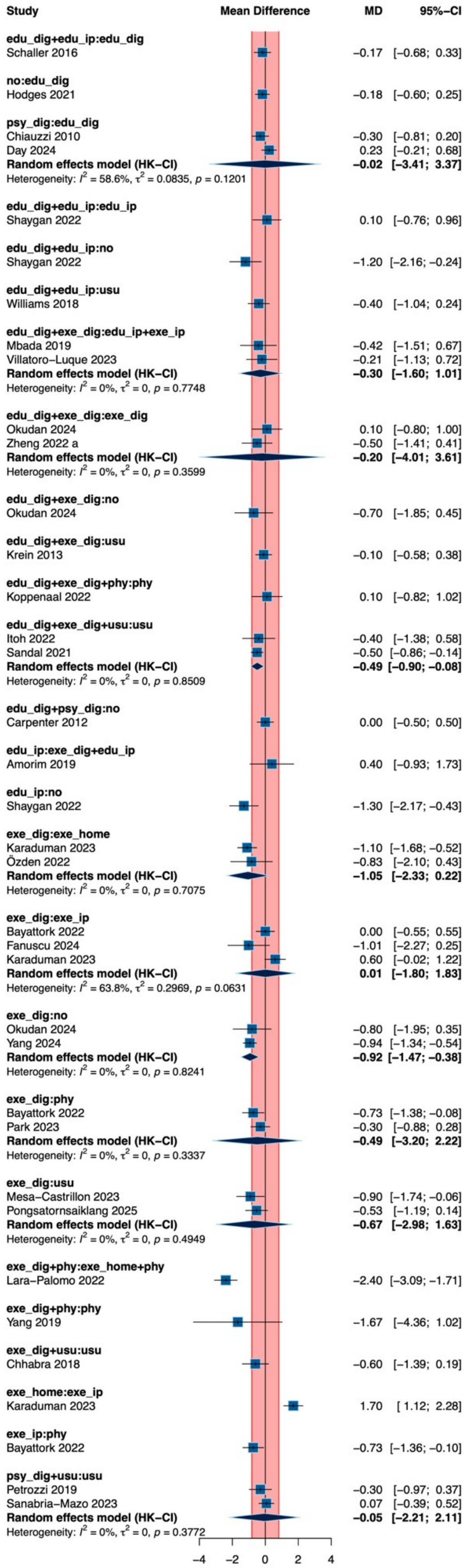

Supplemental Figure 16 - Forest plot of pairwise meta-analyses for network 1 of pain intensity immediate. Red lines represent MCID based on 0.5 SD. Data is backtransformed using the 0-10 Numeric Rating Scale. edu = education, exe = exercise, psy = psychological, phy = physiotherapy, usu = usual care, no = no intervention, dig = digital, ip = in-person, home = home program

Network 2

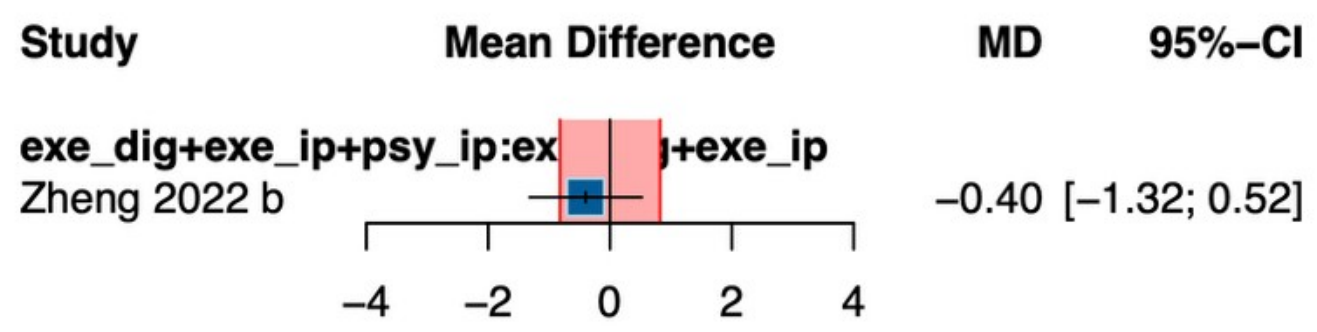

Supplemental Figure 17 - Forest plot of pairwise meta-analyses for network 2 of pain intensity immediate. Red lines represent MCID based on 0.5 SD. Data is backtransformed using the 0-10 Numeric Rating Scale. edu = education, exe = exercise, psy = psychological, phy = physiotherapy, usu = usual care, no = no intervention, dig = digital, ip = in-person, home = home program

Network 3

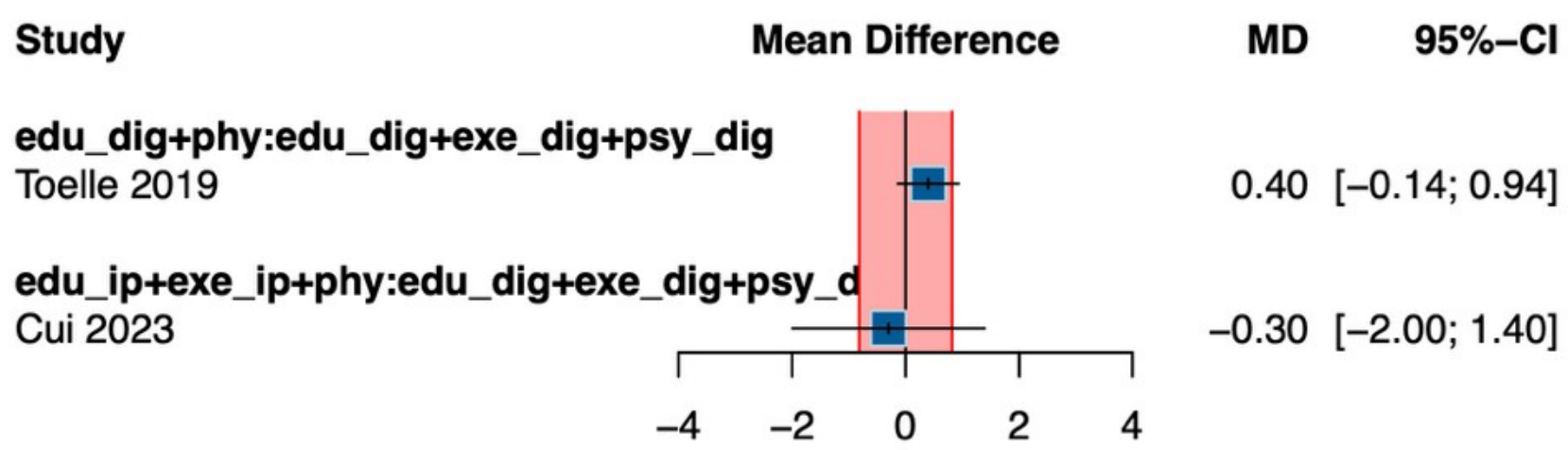

Supplemental Figure 18 - Forest plot of pairwise meta-analyses for network 3 of pain intensity immediate. Red lines represent MCID based on 0.5 SD. Data is backtransformed using the 0-10 Numeric Rating Scale. edu = education, exe = exercise, psy = psychological, phy = physiotherapy, usu = usual care, no = no intervention, dig = digital, ip = in-person, home = home program

Pain intensity >1 week <3months

Network 1

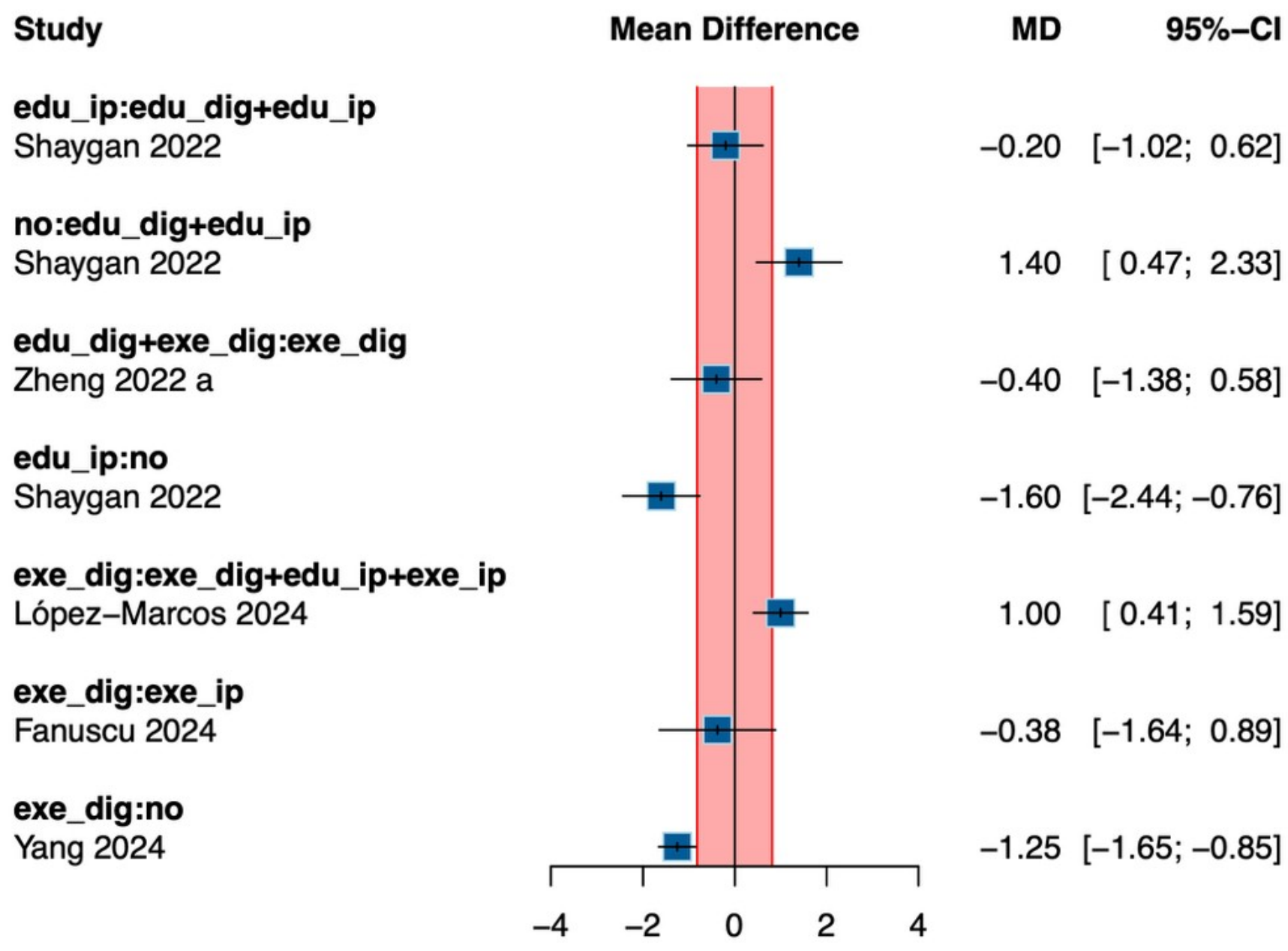

Supplemental Figure 19 - Forest plot of pairwise meta-analyses for network 1 of pain intensity >1 week <3 months. Red lines represent MCID based on 0.5 SD. Data is backtransformed using the 0-10 Numeric Rating Scale. edu = education, exe = exercise, psy = psychological, phy = physiotherapy, usu = usual care, no = no intervention, dig = digital, ip = in-person, home = home program

Network 2

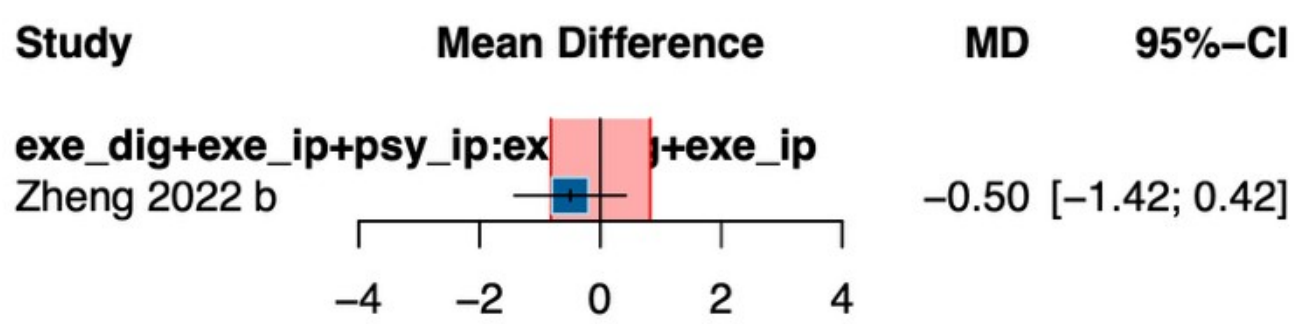

Supplemental Figure 20 - Forest plot of pairwise meta-analyses for network 2 of pain intensity >1 week <3 months. Red lines represent MCID based on 0.5 SD. Data is backtransformed using the 0-10 Numeric Rating Scale. edu = education, exe = exercise, psy = psychological, phy = physiotherapy, usu = usual care, no = no intervention, dig = digital, ip = in-person, home = home program

Network 3

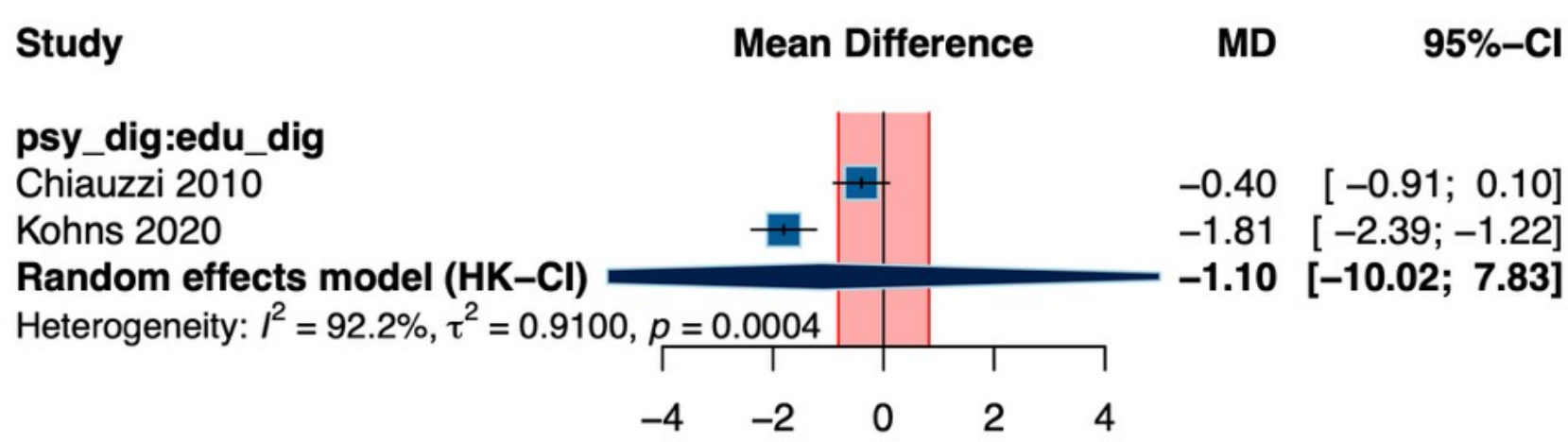

Supplemental Figure 21 - Forest plot of pairwise meta-analyses for network 3 of pain intensity >1 week <3 months. Red lines represent MCID based on 0.5 SD. Data is backtransformed using the 0-10 Numeric Rating Scale. edu = education, exe =

exercise, psy = psychological, phy = physiotherapy, usu = usual care, no = no intervention, dig = digital, ip = in-person, home = home program

Network 4

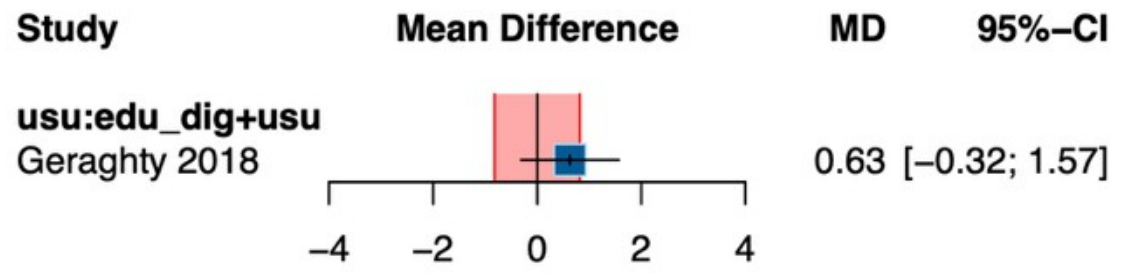

Supplemental Figure 22 - Forest plot of pairwise meta-analyses for network 4 of pain intensity >1 week <3 months. Red lines represent MCID based on 0.5 SD. Data is backtransformed using the 0-10 Numeric Rating Scale. edu = education, exe = exercise, psy = psychological, phy = physiotherapy, usu = usual care, no = no intervention, dig = digital, ip = in-person, home = home program

Pain intensity >3 months <1 year

Network 1

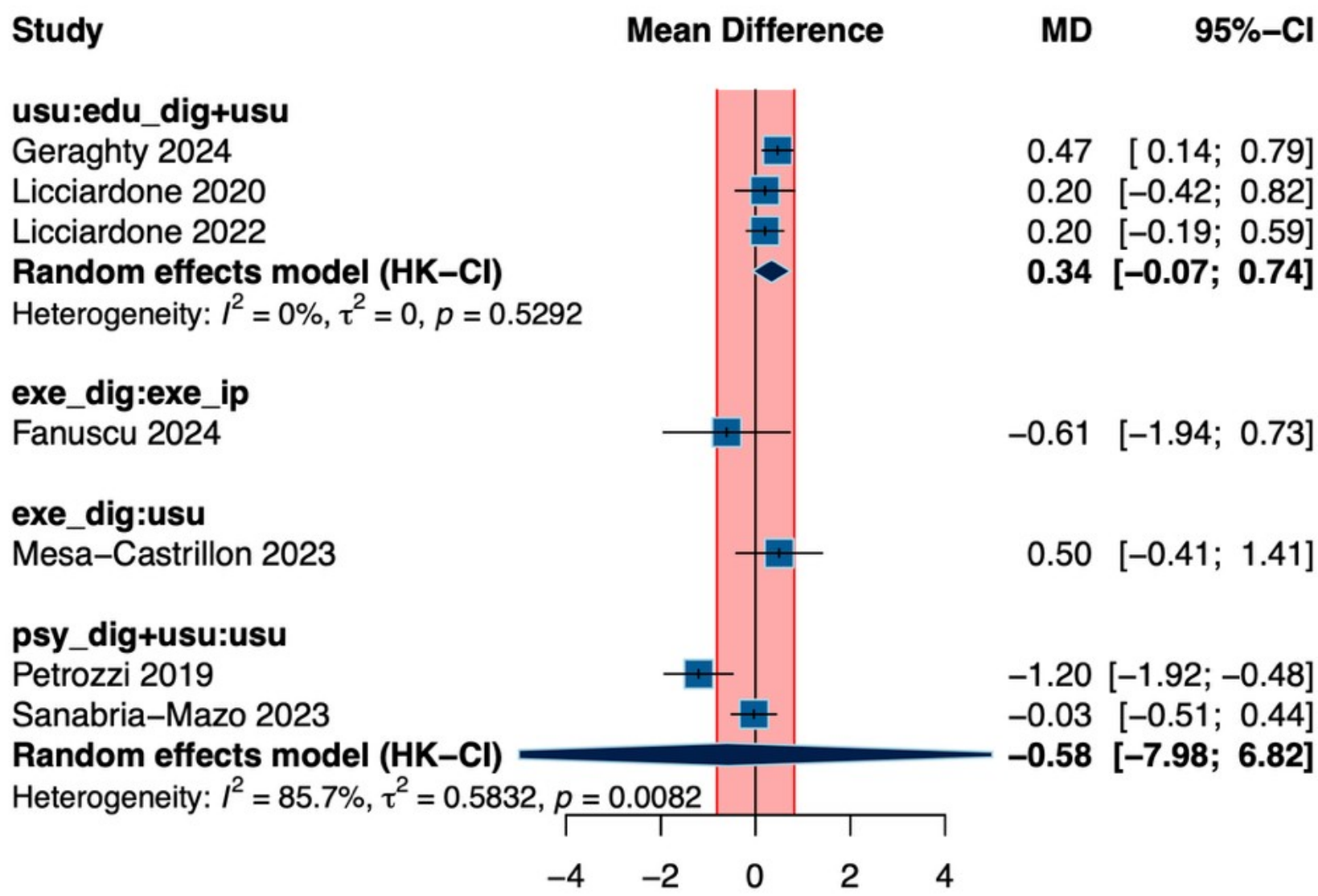

Supplemental Figure 23 - Forest plot of pairwise meta-analyses for network 1 of pain intensity >3 months <1 year. Red lines represent MCID based on 0.5 SD. Data is backtransformed using the 0-10 Numeric Rating Scale. edu = education, exe = exercise, psy = psychological, phy = physiotherapy, usu = usual care, no = no intervention, dig = digital, ip = in-person, home = home program

Network 2

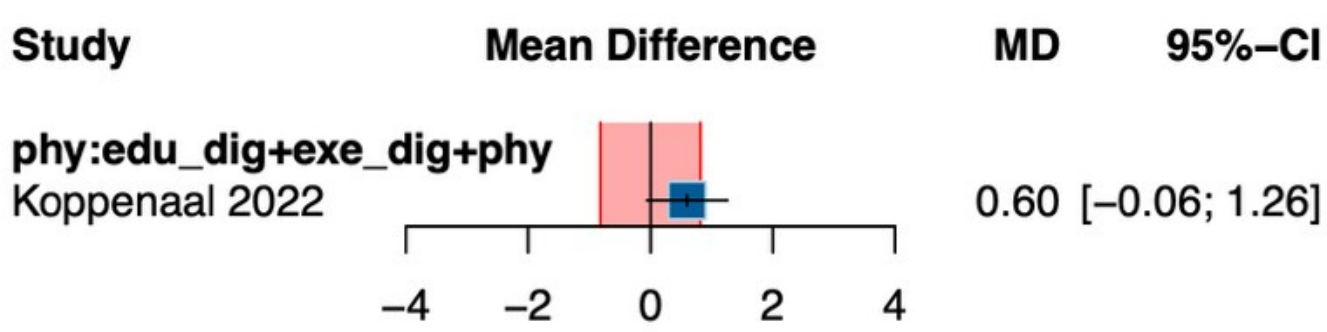

Supplemental Figure 24 - Forest plot of pairwise meta-analyses for network 2 of pain intensity >3 months <1 year. Red lines represent MCID based on 0.5 SD. Data is backtransformed using the 0-10 Numeric Rating Scale. edu = education, exe = exercise, psy = psychological, phy = physiotherapy, usu = usual care, no = no intervention, dig = digital, ip = in-person, home = home program

Network 3

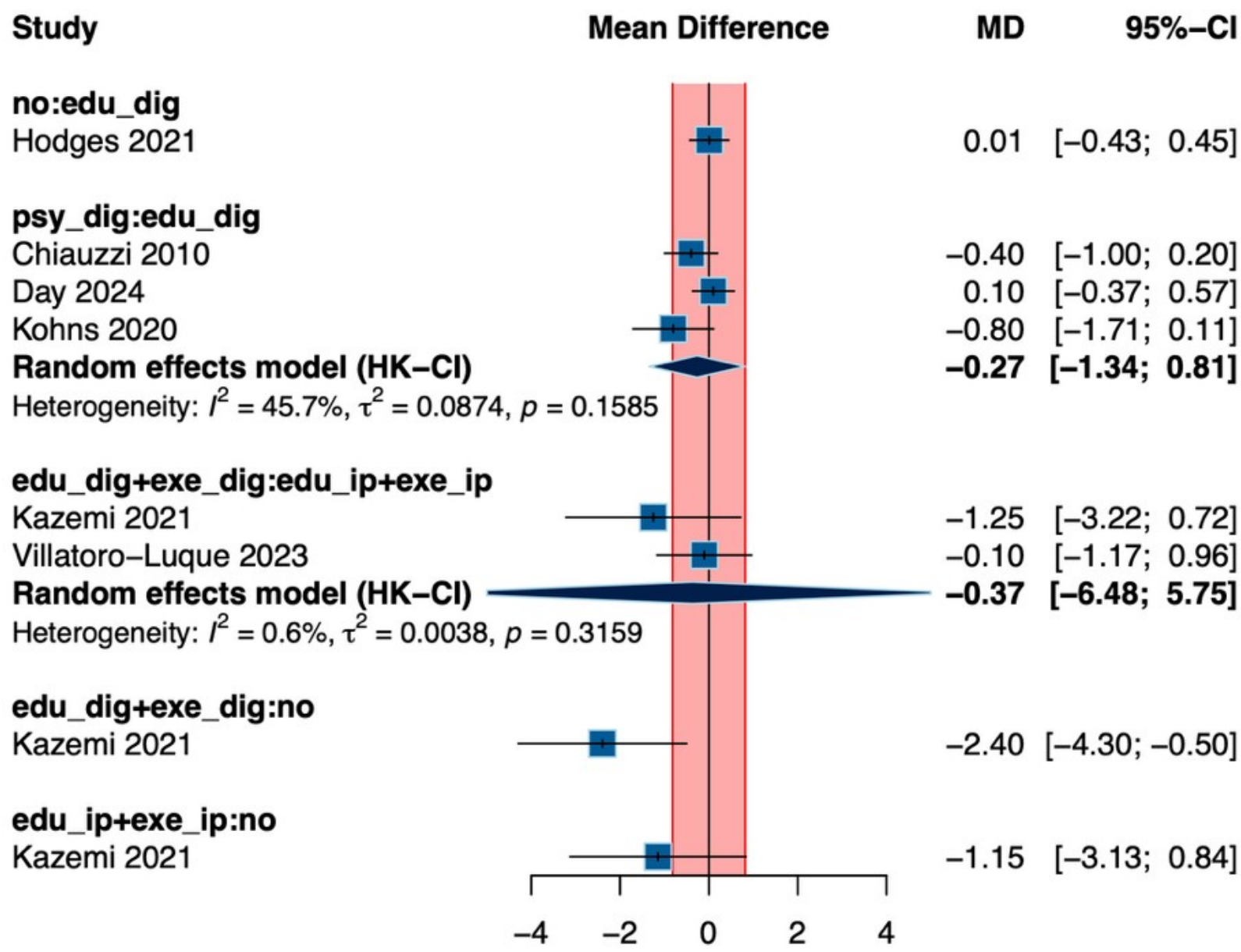

Supplemental Figure 25 - Forest plot of pairwise meta-analyses for network 3 of pain intensity >3 months <1 year. Red lines represent MCID based on 0.5 SD. Data is backtransformed using the 0-10 Numeric Rating Scale. edu = education, exe = exercise, psy = psychological, phy = physiotherapy, usu = usual care, no = no intervention, dig = digital, ip = in-person, home = home program

Supplemental Data 18 – Model fit

Disability immediate

Supplemental Table 8 – Model fit parameters for the disability immediate network

Model	Number of effective parameters	Posterior mean of between-study SD	Residual Deviance	DIC
Standard model	67.20	0.53	68.21	255.89
Additive model	62.38	0.41	68.38	252.07
Two-way Interaction model	64.03	0.46	68.68	253.31

DIC = deviance information criterion

Pain intensity immediate

Supplemental Table 9 – Model fit parameters for the disability immediate network

Model	Number of effective parameters	Posterior mean of between-study SD	Residual Deviance	DIC
Standard model	59.78	0.14	68.34	78.40
Additive model	49.54	0.13	69.73	68.63
Two-way Interaction model	50.12	0.10	65.93	65.62

DIC = deviance information criterion

Supplemental Data 19 – Forest plot of single-effect, additive and interaction model

Disability immediate

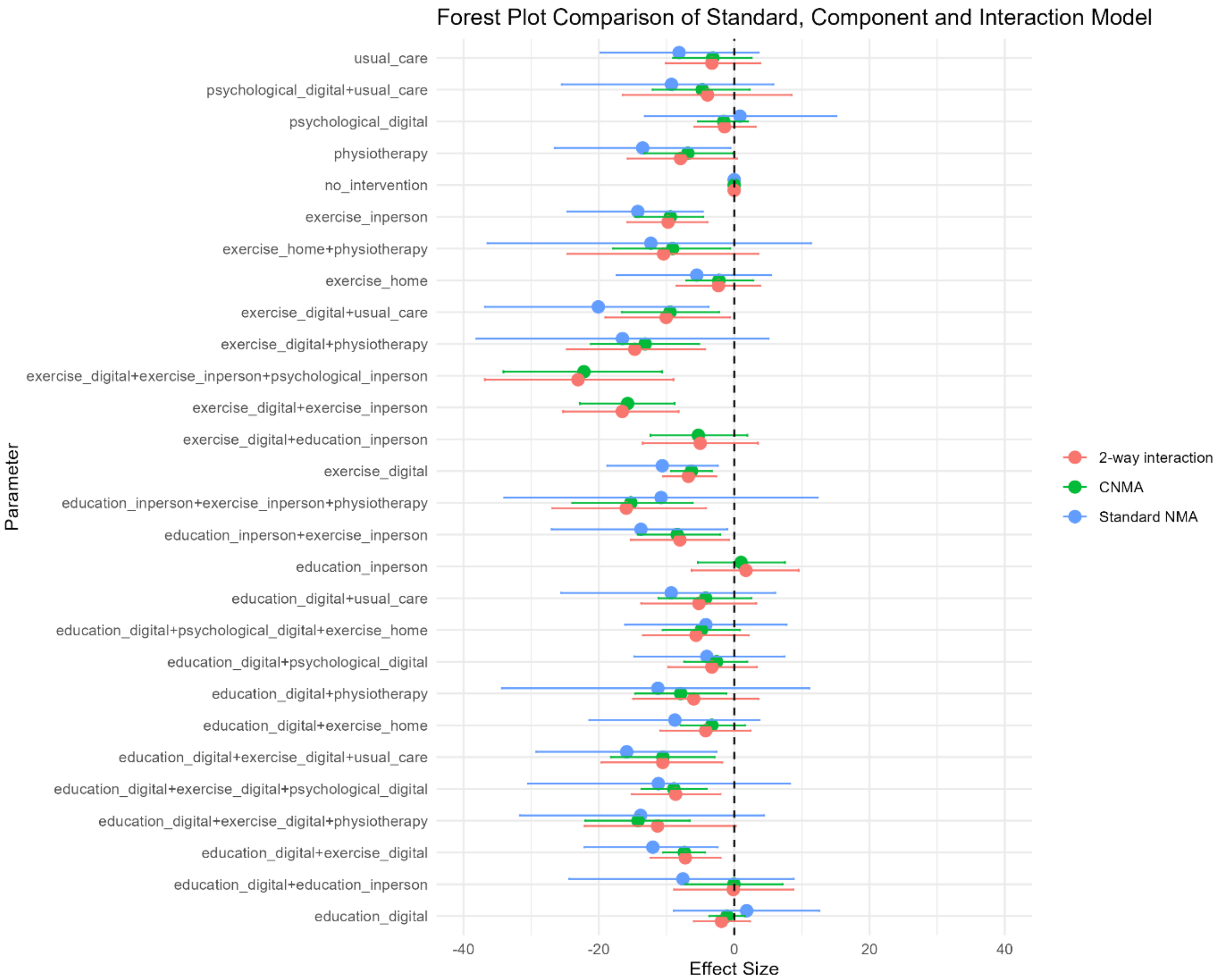

Supplemental Figure 26 – Forest plot of single-effect NMA, additive model CNMA and two-way interaction CNMA estimates vs. no intervention for disability immediate. In cases where no estimate is displayed for the single-effect NMA estimate, the network was disconnected for this intervention and thus the effect was not estimable.

Pain intensity immediate

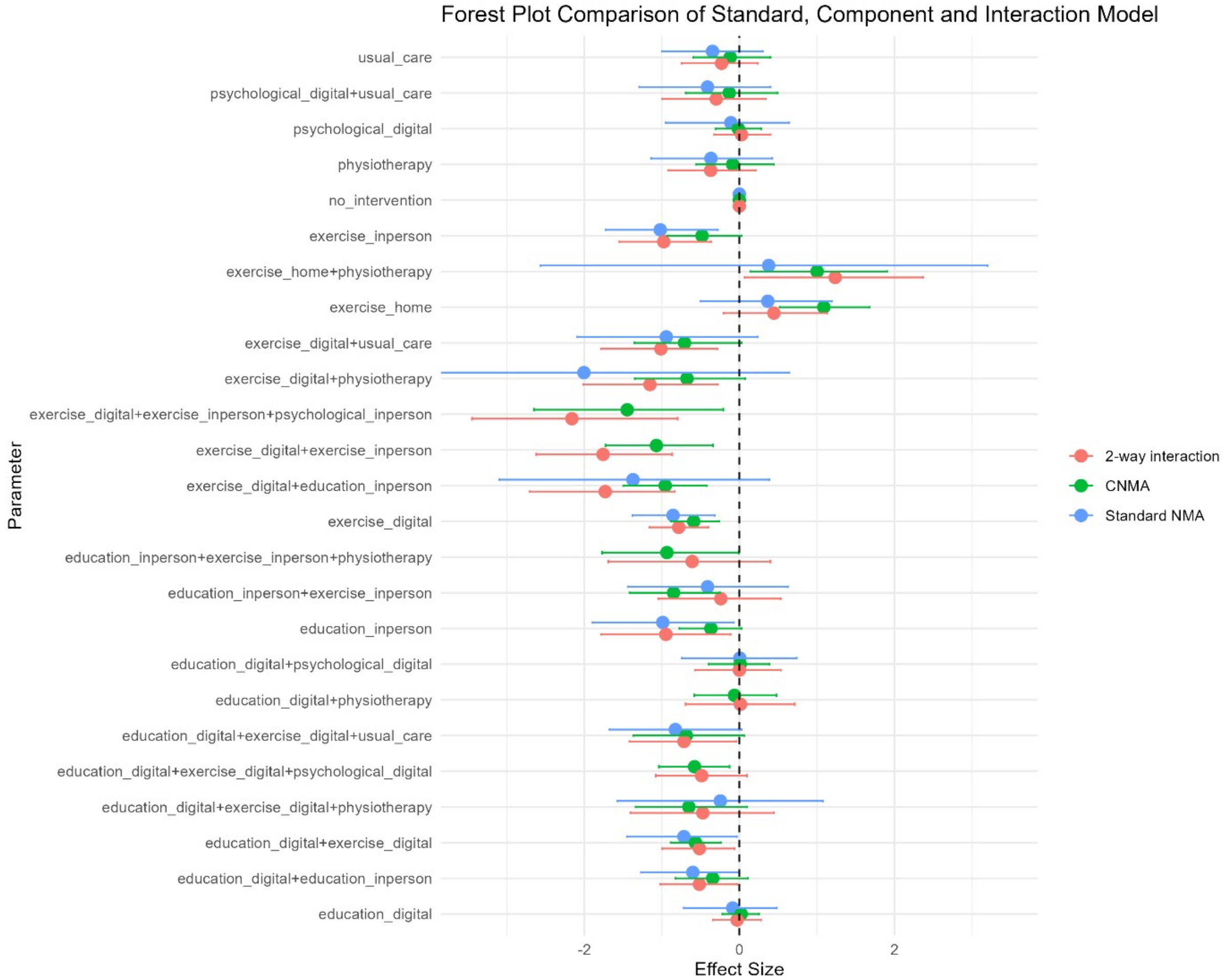

Supplemental Figure 27 – Forest plot of single-effect NMA, additive model CNMA and two-way interaction CNMA estimates vs. no intervention for pain intensity immediate. In cases where no estimate is displayed for the single-effect NMA estimate, the network was disconnected for this intervention and thus the effect was not estimable.

Supplemental Data 20 – Network meta-analysis results

Disability immediate

Component level

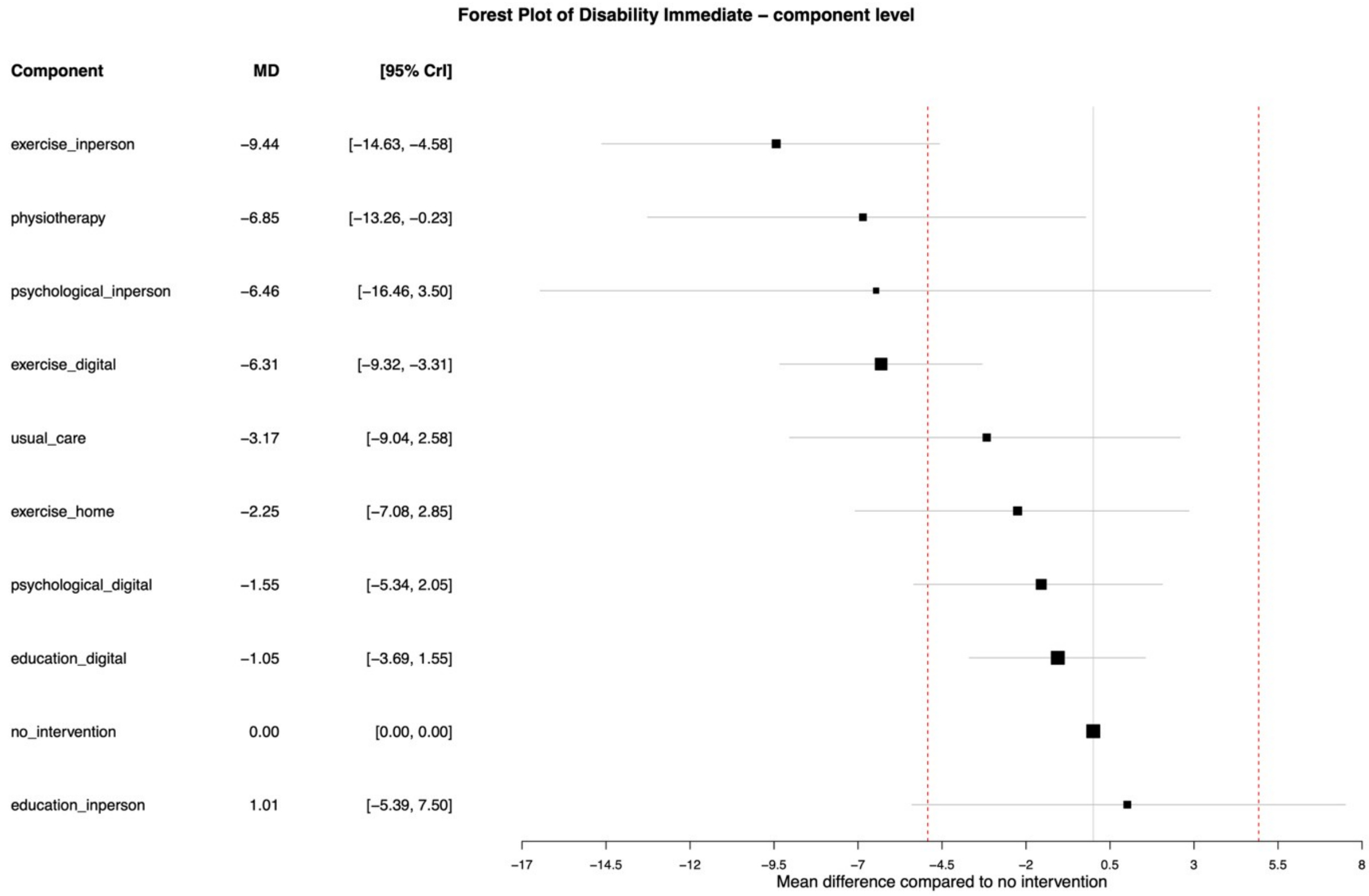

Supplemental Figure 28 – Forest plot of relative effects for the additive disability immediate network on the component level with 95%-credible intervals; red dotted lines represent the MCID of 4.92 for this outcome.

SUCRA table

Supplemental Table 10 – SUCRA table of the additive disability immediate network on the component level

Intervention	Mean Rank	2.5% CI	97.5% CI	SUCRA
exercise_inperson	1.72	1.00	4.00	0.93
physiotherapy	3.13	1.00	8.00	0.79
exercise_digital	3.22	2.00	5.00	0.78
psychological_inperson	3.75	1.00	10.00	0.73
usual_care	5.53	2.00	10.00	0.55
exercise_home	6.35	3.00	10.00	0.47
psychological_digital	6.87	4.00	10.00	0.41
education_digital	7.26	4.00	10.00	0.37
no_intervention	8.55	6.00	10.00	0.24

Intervention	Mean Rank	2.5% CI	97.5% CI	SUCRA
education_inperson	8.63	4.00	10.00	0.24

Intervention level

SUCRA table

Supplemental Table 11 - SUCRA table of the additive disability immediate network on the intervention level

Intervention	Mean Rank	2.5% CI	97.5% CI	SUCRA
exercise_digital+exercise_inperson+psychological_inperson	1.59	1.00	6.00	0.98
exercise_digital+exercise_inperson	3.37	1.00	9.00	0.92
education_inperson+exercise_inperson+physiotherapy	3.52	1.00	9.00	0.91
education_digital+exercise_digital+physiotherapy	4.13	1.00	10.00	0.89
exercise_digital+physiotherapy	5.52	2.00	13.00	0.84
education_digital+exercise_digital+usual_care	7.82	2.00	17.00	0.76
exercise_digital+usual_care	9.58	4.00	18.00	0.69
exercise_inperson	9.59	3.00	20.00	0.69
education_digital+exercise_digital+psychological_digital	9.91	5.00	16.00	0.68
exercise_home+physiotherapy	10.70	4.00	24.00	0.65
education_inperson+exercise_inperson	10.97	4.00	20.00	0.64
education_digital+physiotherapy	12.11	5.00	22.00	0.60
education_digital+exercise_digital	12.62	8.00	17.00	0.59
physiotherapy	14.37	7.00	25.00	0.52
exercise_digital	14.91	9.00	21.00	0.50
exercise_digital+education_inperson	16.48	7.00	26.00	0.45
education_digital+psychological_digital+exercise_home	17.06	8.00	25.00	0.43
psychological_digital+usual_care	17.39	8.00	26.00	0.41
education_digital+usual_care	18.21	8.00	26.00	0.39
education_digital+exercise_home	19.96	12.00	27.00	0.32
usual_care	20.59	13.00	27.00	0.30
education_digital+psychological_digital	20.97	13.00	27.00	0.29
exercise_home	21.97	13.00	28.00	0.25
psychological_digital	23.14	16.00	28.00	0.21
education_digital	23.77	18.00	28.00	0.19

Intervention	Mean Rank	2.5% CI	97.5% CI	SUCRA
education_digital+education_inperson	24.28	14.00	28.00	0.17
no_intervention	25.55	20.00	28.00	0.12
education_inperson	25.93	18.00	28.00	0.11

All pairwise comparisons – CNMA estimates

Data stored on OSF

All clinically plausible combinations of components

For all clinically plausible combinations of components, the top five ranked combinations of components that exceed the MCID compared to no intervention are combined digital and in-person exercise training with physiotherapy (MD: -22.57; 95%-CrI: [-33.24; -11.88]), combined digital and in-person exercise training with in-person psychological intervention (MD: -22.27; 95%-CrI: [-34.09; -10.27]), combined digital exercise training with in-person psychological intervention and physiotherapy (MD:-19.7, 95%-CrI: [-31.84; -6.55]), combined digital and in-person exercise training with usual care (MD: -18.98; 95%-CrI: [-28.35; -9.58]), and combined digital psychological intervention with in-person exercise training and physiotherapy (MD: -17.79; 95%-CrI: [-28.06; -8.12]).

Forest Plot of Disability Immediate – All Possible Combinations

Intervention	MD	[95% CrI]
exercise_digital+exercise_inperson+physiotherapy	−22.57	[−33.24, −11.88]
exercise_digital+exercise_inperson+psychological_inperson	−22.27	[−34.09, −10.27]
exercise_digital+psychological_inperson+physiotherapy	−19.70	[−31.84, −6.55]
exercise_digital+exercise_inperson+usual_care	−18.98	[−28.35, −9.58]
exercise_digital+exercise_inperson+exercise_home	−18.00	[−28.07, −7.55]
psychological_digital+exercise_inperson+physiotherapy	−17.79	[−28.06, −8.12]
psychological_digital+exercise_inperson+psychological_inperson	−17.49	[−28.65, −6.20]
education_digital+exercise_inperson+physiotherapy	−17.39	[−25.78, −8.80]
exercise_digital+psychological_digital+exercise_inperson	−17.25	[−24.96, −9.47]
education_digital+exercise_inperson+psychological_inperson	−17.09	[−27.83, −5.82]
education_digital+exercise_digital+exercise_inperson	−16.85	[−23.57, −10.08]
exercise_digital+physiotherapy+usual_care	−16.42	[−26.82, −5.49]
exercise_digital+psychological_inperson+usual_care	−16.12	[−28.22, −3.99]
exercise_digital+exercise_inperson	−15.72	[−22.50, −8.45]
exercise_digital+physiotherapy+exercise_home	−15.43	[−25.69, −4.53]
education_inperson+exercise_inperson+physiotherapy	−15.26	[−23.86, −6.40]
exercise_digital+psychological_inperson+exercise_home	−15.13	[−26.57, −3.32]
psychological_digital+psychological_inperson+physiotherapy	−14.93	[−26.96, −1.76]
exercise_digital+education_inperson+exercise_inperson	−14.72	[−22.39, −7.00]
exercise_digital+psychological_digital+physiotherapy	−14.68	[−23.91, −4.78]
education_digital+psychological_inperson+physiotherapy	−14.53	[−25.65, −2.09]
exercise_digital+psychological_digital+psychological_inperson	−14.38	[−24.57, −3.67]
education_digital+exercise_digital+physiotherapy	−14.28	[−21.88, −6.36]
psychological_digital+exercise_inperson+usual_care	−14.20	[−23.04, −5.64]
education_digital+exercise_digital+psychological_inperson	−13.98	[−23.90, −3.63]
education_digital+exercise_inperson+usual_care	−13.80	[−22.46, −5.59]
psychological_digital+exercise_inperson+exercise_home	−13.22	[−21.75, −4.66]
exercise_digital+physiotherapy	−13.15	[−20.75, −4.91]
exercise_digital+psychological_inperson	−12.86	[−22.50, −2.59]
education_digital+exercise_inperson+exercise_home	−12.82	[−20.69, −4.84]
exercise_digital+education_inperson+physiotherapy	−12.16	[−21.45, −1.85]
education_digital+psychological_digital+exercise_inperson	−12.07	[−18.81, −5.50]
exercise_digital+education_inperson+psychological_inperson	−11.86	[−23.86, 0.64]
exercise_digital+usual_care+exercise_home	−11.84	[−21.46, −1.92]
psychological_digital+physiotherapy+usual_care	−11.64	[−22.19, −1.05]
psychological_digital+psychological_inperson+usual_care	−11.34	[−23.20, 0.54]
education_digital+physiotherapy+usual_care	−11.24	[−20.87, −1.68]
exercise_digital+psychological_digital+usual_care	−11.09	[−19.44, −3.16]
psychological_digital+exercise_inperson	−10.94	[−17.17, −4.71]
education_digital+psychological_inperson+usual_care	−10.94	[−22.91, 1.05]
education_digital+exercise_digital+usual_care	−10.69	[−18.34, −3.11]
psychological_digital+physiotherapy+exercise_home	−10.65	[−20.48, −0.66]
education_digital+exercise_inperson	−10.54	[−15.98, −5.13]
psychological_digital+psychological_inperson+exercise_home	−10.36	[−21.24, 0.87]
education_digital+physiotherapy+exercise_home	−10.25	[−18.36, −1.46]
exercise_digital+psychological_digital+exercise_home	−10.11	[−17.56, −2.24]
education_digital+psychological_inperson+exercise_home	−9.95	[−20.27, 1.07]
psychological_digital+education_inperson+exercise_inperson	−9.94	[−17.33, −2.61]
education_digital+exercise_digital+exercise_home	−9.71	[−16.07, −3.01]
exercise_digital+usual_care	−9.57	[−16.64, −2.45]
education_digital+education_inperson+exercise_inperson	−9.54	[−16.68, −2.65]
education_digital+psychological_digital+physiotherapy	−9.51	[−18.15, −0.80]
exercise_inperson	−9.41	[−14.41, −4.34]
education_digital+psychological_digital+psychological_inperson	−9.21	[−19.98, 1.50]
exercise_home+physiotherapy	−9.13	[−17.58, −0.43]
education_digital+exercise_digital+psychological_digital	−8.96	[−13.93, −4.06]
exercise_digital+exercise_home	−8.58	[−15.63, −1.31]
exercise_digital+education_inperson+usual_care	−8.57	[−19.29, 2.31]
education_inperson+exercise_inperson	−8.41	[−14.55, −2.09]
psychological_digital+physiotherapy	−8.38	[−16.77, 0.15]
psychological_digital+psychological_inperson	−8.08	[−18.12, 2.27]
education_digital+physiotherapy	−7.98	[−14.69, −1.24]
exercise_digital+psychological_digital	−7.83	[−12.45, −3.27]
education_digital+psychological_inperson	−7.68	[−17.59, 2.43]
exercise_digital+education_inperson+exercise_home	−7.58	[−16.58, 1.56]
education_digital+exercise_digital	−7.43	[−10.57, −4.37]
psychological_digital+education_inperson+physiotherapy	−7.38	[−17.51, 3.45]
psychological_digital+education_inperson+psychological_inperson	−7.08	[−19.28, 5.37]
psychological_digital+usual_care+exercise_home	−7.07	[−15.89, 1.78]
education_digital+education_inperson+physiotherapy	−6.98	[−16.09, 2.94]
physiotherapy	−6.85	[−12.97, −0.34]
exercise_digital+psychological_digital+education_inperson	−6.83	[−14.76, 1.38]
education_digital+education_inperson+psychological_inperson	−6.68	[−19.00, 5.84]
education_digital+usual_care+exercise_home	−6.67	[−14.74, 1.69]
education_digital+exercise_digital+education_inperson	−6.43	[−13.87, 1.19]
exercise_digital	−6.31	[−9.27, −3.16]
education_digital+psychological_digital+usual_care	−5.92	[−14.20, 2.28]
exercise_digital+education_inperson	−5.31	[−12.22, 1.78]
education_digital+psychological_digital+exercise_home	−4.93	[−10.68, 1.13]
psychological_digital+usual_care	−4.79	[−12.20, 2.17]
education_digital+usual_care	−4.39	[−11.28, 2.58]
psychological_digital+exercise_home	−3.80	[−9.51, 2.37]
psychological_digital+education_inperson+usual_care	−3.79	[−14.82, 7.40]
education_digital+exercise_home	−3.40	[−8.30, 1.67]
education_digital+education_inperson+usual_care	−3.39	[−14.19, 7.59]
usual_care	−3.26	[−9.14, 2.38]
psychological_digital+education_inperson+exercise_home	−2.81	[−11.08, 5.89]
education_digital+psychological_digital	−2.66	[−7.54, 2.08]
education_digital+education_inperson+exercise_home	−2.41	[−10.64, 6.12]
exercise_home	−2.28	[−7.26, 2.99]
education_digital+psychological_digital+education_inperson	−1.66	[−10.31, 7.19]
psychological_digital	−1.53	[−5.13, 2.16]
education_digital	−1.13	[−3.83, 1.51]
psychological_digital+education_inperson	−0.53	[−8.21, 7.29]
education_digital+education_inperson	−0.13	[−7.52, 7.42]
no_intervention	0.00	[0.00, 0.00]
education_inperson	1.00	[−5.41, 7.49]

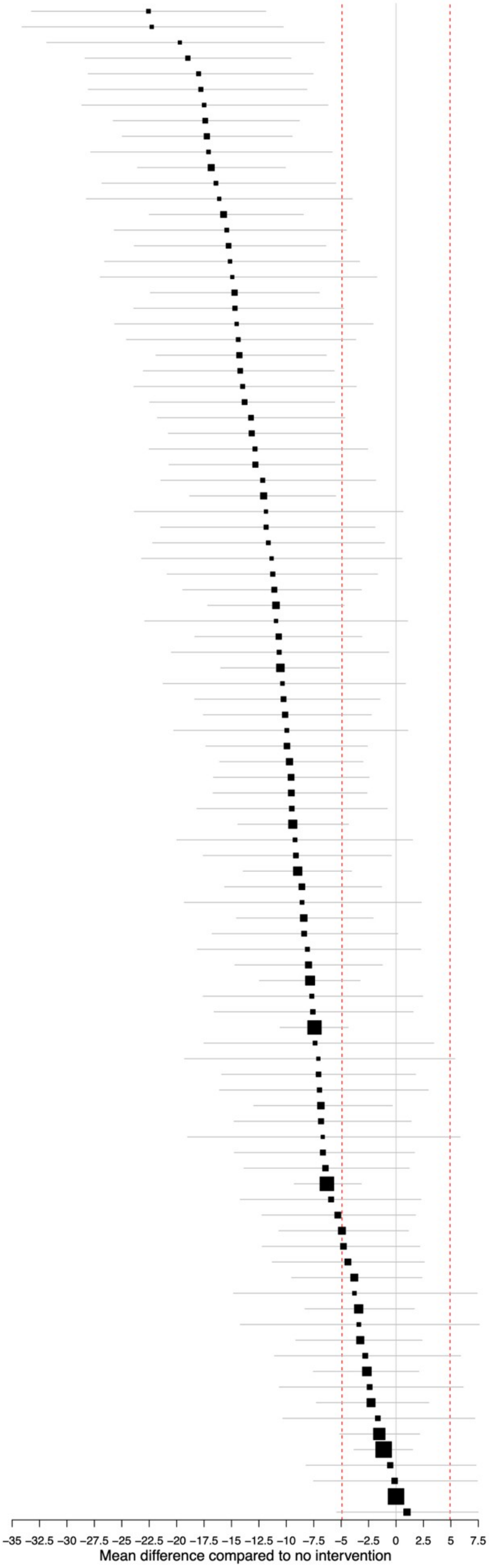

Supplemental Figure 29– Forest plot of relative effects for the additive disability immediate network for all clinically plausible interventions with 95%-credible intervals; red dotted lines represent the MCID of 4.92 for this outcome.

SUCRA table

Supplemental Table 12 - SUCRA table of the additive disability immediate network for all clinically plausible interventions

Intervention	Mean Rank	2.5% CI	97.5% CI	SUCRA
exercise_digital+exercise_inperson+physiotherapy	4.54	1.00	23.00	0.96
exercise_digital+exercise_inperson+psychological_inperson	5.74	1.00	33.00	0.95
exercise_digital+psychological_inperson+physiotherapy	10.48	1.00	47.03	0.90
exercise_digital+exercise_inperson+usual_care	10.75	1.00	36.03	0.90
psychological_digital+exercise_inperson+physiotherapy	14.21	2.00	46.00	0.86
exercise_digital+exercise_inperson+exercise_home	14.76	1.00	53.00	0.86
education_digital+exercise_inperson+physiotherapy	14.83	2.00	42.00	0.86
exercise_digital+psychological_digital+exercise_inperson	14.97	3.00	42.00	0.86
education_digital+exercise_digital+exercise_inperson	16.28	3.00	43.00	0.84
psychological_digital+exercise_inperson+psychological_inperson	16.43	2.00	60.00	0.84
education_digital+exercise_inperson+psychological_inperson	17.92	2.00	63.03	0.83
exercise_digital+physiotherapy+usual_care	18.28	2.00	52.00	0.82
exercise_digital+psychological_inperson+usual_care	20.62	2.00	63.00	0.80
exercise_digital+exercise_inperson	21.28	6.00	53.00	0.79
education_inperson+exercise_inperson+physiotherapy	21.80	4.00	51.00	0.79
exercise_digital+physiotherapy+exercise_home	22.92	3.00	66.00	0.77
exercise_digital+education_inperson+exercise_inperson	24.10	4.00	54.00	0.76
exercise_digital+psychological_digital+physiotherapy	24.92	5.00	61.00	0.75
exercise_digital+psychological_inperson+exercise_home	25.27	3.00	75.00	0.75
education_digital+exercise_digital+physiotherapy	25.79	7.00	55.00	0.74
psychological_digital+psychological_inperson+physiotherapy	26.33	3.00	79.00	0.74
psychological_digital+exercise_inperson+usual_care	26.60	5.00	59.00	0.74
exercise_digital+psychological_digital+psychological_inperson	27.32	6.00	70.03	0.73
education_digital+psychological_inperson+physiotherapy	27.51	4.00	76.00	0.73
education_digital+exercise_inperson+usual_care	28.64	5.00	65.00	0.72
education_digital+exercise_digital+psychological_inperson	28.96	6.00	72.00	0.71
exercise_digital+physiotherapy	31.94	11.00	67.03	0.68
psychological_digital+exercise_inperson+exercise_home	32.14	8.00	73.00	0.68
education_digital+exercise_inperson+exercise_home	34.03	8.00	73.00	0.66

Intervention	Mean Rank	2.5% CI	97.5% CI	SUCRA
exercise_digital+psychological_inperson	34.94	9.00	80.00	0.65
exercise_digital+education_inperson+physiotherapy	36.09	6.00	74.03	0.64
education_digital+psychological_digital+exercise_inperson	36.71	11.00	70.03	0.63
exercise_digital+usual_care+exercise_home	38.03	8.00	78.00	0.62
exercise_digital+education_inperson+psychological_inperson	38.96	4.00	85.00	0.61
psychological_digital+physiotherapy+usual_care	39.23	7.00	79.00	0.61
education_digital+physiotherapy+usual_care	40.95	9.00	77.00	0.59
exercise_digital+psychological_digital+usual_care	40.97	13.00	70.00	0.59
psychological_digital+psychological_inperson+usual_care	41.79	8.00	86.00	0.58
education_digital+exercise_digital+usual_care	42.99	12.98	73.00	0.57
psychological_digital+exercise_inperson	43.12	16.00	78.00	0.57
education_digital+psychological_inperson+usual_care	43.84	7.00	88.00	0.56
psychological_digital+physiotherapy+exercise_home	44.78	12.00	85.00	0.55
education_digital+exercise_inperson	45.15	16.00	80.00	0.54
education_digital+physiotherapy+exercise_home	46.51	17.00	83.00	0.53
psychological_digital+education_inperson+exercise_inperson	46.78	17.00	74.00	0.53
exercise_digital+psychological_digital+exercise_home	47.12	19.00	79.00	0.52
psychological_digital+psychological_inperson+exercise_home	47.35	12.00	90.00	0.52
education_digital+education_inperson+exercise_inperson	48.77	16.00	77.00	0.51
education_digital+exercise_digital+exercise_home	49.11	20.00	78.00	0.50
education_digital+psychological_inperson+exercise_home	49.23	13.00	91.00	0.50
exercise_digital+usual_care	49.80	21.00	78.00	0.50
education_digital+psychological_digital+physiotherapy	50.16	16.00	84.00	0.49
exercise_inperson	51.84	20.00	85.03	0.48
education_digital+psychological_digital+psychological_inperson	52.38	14.00	91.00	0.47
education_digital+exercise_digital+psychological_digital	52.76	29.00	72.00	0.47
exercise_home+physiotherapy	53.34	20.00	90.00	0.46
exercise_digital+education_inperson+usual_care	53.46	9.00	89.00	0.46
education_inperson+exercise_inperson	55.65	25.00	81.00	0.44
exercise_digital+exercise_home	55.90	22.98	88.00	0.43
psychological_digital+physiotherapy	56.96	22.00	90.00	0.42

Intervention	Mean Rank	2.5% CI	97.5% CI	SUCRA
psychological_digital+psychological_inperson	58.60	19.00	95.00	0.41
education_digital+physiotherapy	58.99	27.98	87.00	0.40
exercise_digital+education_inperson+exercise_home	59.47	18.98	90.00	0.40
exercise_digital+psychological_digital	59.82	36.00	81.00	0.39
education_digital+psychological_inperson	60.33	19.98	95.00	0.39
psychological_digital+education_inperson+physiotherapy	60.49	19.00	92.00	0.39
education_digital+exercise_digital	61.75	38.98	81.00	0.37
psychological_digital+education_inperson+psychological_inperso n	61.81	15.00	96.00	0.37
education_digital+education_inperson+physiotherapy	62.43	21.00	91.00	0.37
exercise_digital+psychological_digital+education_inperson	63.12	27.00	86.00	0.36
psychological_digital+usual_care+exercise_home	63.28	30.00	90.00	0.36
education_digital+education_inperson+psychological_inperson	63.45	14.00	96.00	0.36
education_digital+exercise_digital+education_inperson	64.95	28.00	86.00	0.34
education_digital+usual_care+exercise_home	64.99	29.00	91.00	0.34
physiotherapy	65.54	34.00	93.00	0.33
education_digital+psychological_digital+usual_care	67.94	29.00	91.00	0.31
exercise_digital	68.28	43.00	88.00	0.31
exercise_digital+education_inperson	71.43	38.00	90.00	0.27
education_digital+psychological_digital+exercise_home	73.86	47.00	92.00	0.25
psychological_digital+usual_care	74.47	44.00	93.00	0.24
education_digital+usual_care	75.67	42.00	94.00	0.23
psychological_digital+education_inperson+usual_care	76.00	29.00	96.00	0.23
education_digital+education_inperson+usual_care	77.03	29.00	97.00	0.22
psychological_digital+exercise_home	78.99	51.00	95.00	0.20
education_digital+exercise_home	80.28	54.00	95.00	0.18
usual_care	81.42	57.00	96.00	0.17
psychological_digital+education_inperson+exercise_home	81.77	51.00	96.00	0.17
education_digital+psychological_digital	82.74	57.98	95.03	0.16
education_digital+education_inperson+exercise_home	82.94	51.00	96.00	0.16
exercise_home	84.34	56.00	97.00	0.14
education_digital+psychological_digital+education_inperson	84.69	50.00	96.00	0.14

Intervention	Mean Rank	2.5% CI	97.5% CI	SUCRA
psychological_digital	87.06	68.00	97.00	0.11
education_digital	87.98	67.00	97.00	0.10
psychological_digital+education_inperson	89.08	65.00	97.00	0.09
education_digital+education_inperson	89.72	65.00	97.00	0.09
no_intervention	91.23	74.00	97.00	0.07
education_inperson	92.98	75.00	97.00	0.05

Pain intensity immediate

Component level

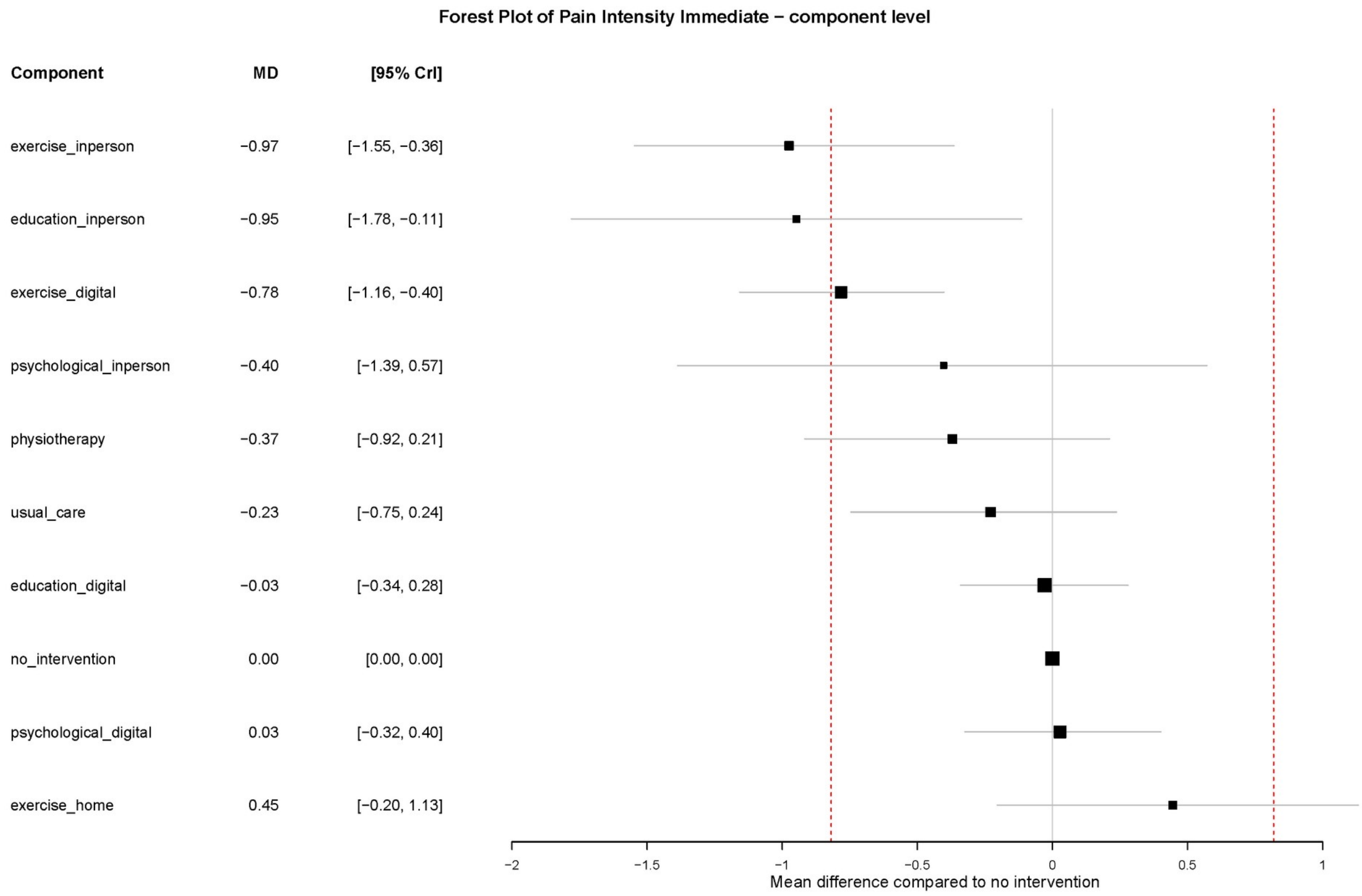

Supplemental Figure 30 – Forest plot of relative effects for the interaction pain intensity network on the component level with 95%-credible intervals; red dotted lines represent the MCID of 0.82 for this outcome.

SUCRA table

Supplemental Table 13 - SUCRA table of the interaction pain intensity immediate network on the component level

Intervention	Mean Rank	2.5% CI	97.5% CI	SUCRA
exercise_inperson	1.87	1.00	4.00	0.91
education_inperson	2.30	1.00	6.00	0.87
exercise_digital	2.76	1.00	4.00	0.82
psychological_inperson	5.01	1.00	10.00	0.60
physiotherapy	5.02	3.00	9.00	0.60
usual_care	5.83	4.00	9.00	0.52
education_digital	7.29	5.00	10.00	0.37
no_intervention	7.59	5.00	10.00	0.34
psychological_digital	7.78	5.00	10.00	0.32
exercise_home	9.56	6.00	10.00	0.14

Intervention level

SUCRA table

Supplemental Table 14 - SUCRA table of the interaction pain intensity immediate network on the intervention level

Intervention	Mean Rank	2.5% CI	97.5% CI	SUCRA
exercise_digital+exercise_inperson+psychological_inperson	1.73	1.00	6.00	0.97
exercise_digital+exercise_inperson	2.42	1.00	4.00	0.94
exercise_digital+education_inperson	2.61	1.00	6.00	0.94
exercise_digital+physiotherapy	5.51	3.00	12.00	0.82
exercise_digital+usual_care	6.44	3.00	12.00	0.78
exercise_inperson	6.85	4.00	14.00	0.77
education_inperson	7.77	2.00	18.00	0.73
exercise_digital	8.79	6.00	14.00	0.69
education_digital+exercise_digital+usual_care	9.79	4.00	18.00	0.65
education_inperson+exercise_inperson+physiotherapy	11.53	4.00	22.00	0.58
education_digital+exercise_digital	12.30	8.00	17.00	0.55
education_digital+education_inperson	12.35	5.00	19.00	0.55
education_digital+exercise_digital+psychological_digital	12.74	6.00	18.00	0.53
education_digital+exercise_digital+physiotherapy	13.24	5.00	23.00	0.51
physiotherapy	14.70	8.00	22.00	0.45
psychological_digital+usual_care	15.69	8.00	23.00	0.41
education_inperson+exercise_inperson	16.58	7.00	24.00	0.38
usual_care	16.90	11.00	23.00	0.36
education_digital	19.41	13.00	23.00	0.26
education_digital+psychological_digital	19.59	11.00	24.00	0.26
education_digital+physiotherapy	19.89	12.00	24.00	0.24
no_intervention	19.95	14.00	24.00	0.24
psychological_digital	20.23	14.00	24.00	0.23
exercise_home	23.20	18.00	25.00	0.11
exercise_home+physiotherapy	24.78	22.98	25.00	0.05

All pairwise comparisons – CNMA estimates

Data stored on OSF

All clinically plausible combinations of components

For all clinically plausible combinations of components, no combination exceeded the MCID compared to no intervention. The interventions combined digital and in-person exercise training with in-person education (MD: -1.47; 95%-CrI: [-2.71; -0.17]), combined digital and in-person exercise with in-person psychological intervention (MD: -1.41; 95%-CrI: [-2.18; -0.59]), combined digital exercise training with in-person education and in-person psychological intervention (MD: -1.36; 95%-CrI: [-2.56; -0.17]), combined digital and in-person exercise training with usual care (MD: -1.15; 95%-CrI: [-2.13; -0.10]), and combined digital and in-person exercise training with physiotherapy (MD: -1.12; 95%-CrI: [-2.17; -0.02]) were the five highest ranked interventions among those with at least one digital component.

Forest Plot of Pain Intensity Immediate

Intervention	MD	[95% CrI]
exercise_digital+exercise_inperson+psychological_inperson	−1.47	[−2.71, −0.17]
exercise_digital+education_inperson+exercise_inperson	−1.41	[−2.18, −0.59]
exercise_digital+education_inperson+psychological_inperson	−1.36	[−2.56, −0.17]
exercise_digital+exercise_inperson+usual_care	−1.15	[−2.13, −0.10]
exercise_digital+exercise_inperson+physiotherapy	−1.12	[−2.17, −0.02]
exercise_digital+psychological_inperson+usual_care	−1.10	[−2.35, 0.17]
exercise_digital+psychological_inperson+physiotherapy	−1.07	[−2.38, 0.24]
exercise_digital+psychological_digital+exercise_inperson	−1.05	[−1.80, −0.26]
exercise_digital+exercise_inperson	−1.05	[−1.74, −0.30]
exercise_digital+education_inperson+usual_care	−1.05	[−1.92, −0.07]
education_digital+exercise_digital+exercise_inperson	−1.02	[−1.72, −0.29]
exercise_digital+education_inperson+physiotherapy	−1.02	[−1.87, −0.03]
exercise_digital+psychological_digital+psychological_inperson	−1.00	[−2.14, 0.13]
exercise_digital+psychological_inperson	−1.00	[−2.09, 0.14]
education_digital+exercise_digital+psychological_inperson	−0.97	[−2.07, 0.14]
exercise_digital+psychological_digital+education_inperson	−0.95	[−1.59, −0.26]
exercise_digital+education_inperson	−0.94	[−1.49, −0.32]
education_digital+exercise_digital+education_inperson	−0.92	[−1.47, −0.36]
education_inperson+exercise_inperson+physiotherapy	−0.91	[−1.79, 0.00]
psychological_digital+exercise_inperson+psychological_inperson	−0.89	[−2.08, 0.30]
education_digital+exercise_inperson+psychological_inperson	−0.87	[−2.04, 0.32]
psychological_digital+education_inperson+exercise_inperson	−0.84	[−1.50, −0.19]
education_inperson+exercise_inperson	−0.84	[−1.41, −0.24]
education_digital+education_inperson+exercise_inperson	−0.81	[−1.41, −0.19]
psychological_digital+education_inperson+psychological_inperson	−0.79	[−1.97, 0.38]
education_digital+education_inperson+psychological_inperson	−0.76	[−1.91, 0.37]
exercise_digital+physiotherapy+usual_care	−0.76	[−1.78, 0.37]
exercise_digital+psychological_digital+usual_care	−0.68	[−1.43, 0.11]
exercise_digital+usual_care	−0.68	[−1.37, 0.04]
exercise_digital+psychological_digital+physiotherapy	−0.66	[−1.50, 0.26]
education_digital+exercise_digital+usual_care	−0.66	[−1.36, 0.05]
exercise_digital+physiotherapy	−0.65	[−1.40, 0.18]
education_digital+exercise_digital+physiotherapy	−0.63	[−1.36, 0.11]
exercise_digital+psychological_digital	−0.58	[−1.00, −0.12]
exercise_digital	−0.58	[−0.89, −0.22]
psychological_digital+exercise_inperson+usual_care	−0.58	[−1.40, 0.26]
education_digital+exercise_digital+psychological_digital	−0.56	[−1.02, −0.11]
education_digital+exercise_digital	−0.55	[−0.88, −0.23]
psychological_digital+exercise_inperson+physiotherapy	−0.55	[−1.48, 0.43]
education_digital+exercise_inperson+usual_care	−0.55	[−1.36, 0.30]
psychological_digital+psychological_inperson+usual_care	−0.53	[−1.72, 0.67]
education_digital+exercise_inperson+physiotherapy	−0.52	[−1.35, 0.33]
psychological_digital+psychological_inperson+physiotherapy	−0.50	[−1.75, 0.76]
education_digital+psychological_inperson+usual_care	−0.50	[−1.65, 0.72]
psychological_digital+exercise_inperson	−0.47	[−1.04, 0.14]
education_digital+psychological_inperson+physiotherapy	−0.47	[−1.66, 0.73]
psychological_digital+education_inperson+usual_care	−0.47	[−1.28, 0.32]
exercise_inperson	−0.47	[−0.95, 0.06]
education_digital+psychological_digital+exercise_inperson	−0.45	[−1.08, 0.21]
education_digital+exercise_inperson	−0.45	[−0.98, 0.13]
psychological_digital+education_inperson+physiotherapy	−0.44	[−1.21, 0.38]
education_digital+education_inperson+usual_care	−0.44	[−1.22, 0.31]
psychological_digital+psychological_inperson	−0.42	[−1.51, 0.65]
education_digital+education_inperson+physiotherapy	−0.42	[−1.09, 0.26]
education_digital+psychological_digital+psychological_inperson	−0.40	[−1.50, 0.72]
education_digital+psychological_inperson	−0.40	[−1.44, 0.67]
psychological_digital+education_inperson	−0.37	[−0.89, 0.13]
education_inperson	−0.37	[−0.77, 0.04]
education_digital+psychological_digital+education_inperson	−0.34	[−0.92, 0.20]
education_digital+education_inperson	−0.34	[−0.80, 0.12]
psychological_digital+physiotherapy+usual_care	−0.18	[−1.08, 0.71]
education_digital+physiotherapy+usual_care	−0.15	[−0.97, 0.66]
psychological_digital+usual_care	−0.11	[−0.70, 0.46]
usual_care	−0.10	[−0.58, 0.38]
education_digital+psychological_digital+usual_care	−0.08	[−0.76, 0.55]
psychological_digital+physiotherapy	−0.08	[−0.74, 0.58]
education_digital+usual_care	−0.08	[−0.64, 0.49]
physiotherapy	−0.08	[−0.61, 0.44]
education_digital+psychological_digital+physiotherapy	−0.05	[−0.74, 0.63]
education_digital+physiotherapy	−0.05	[−0.61, 0.49]
psychological_digital	−0.00	[−0.31, 0.28]
no_intervention	0.00	[0.00, 0.00]
education_digital+psychological_digital	0.02	[−0.39, 0.39]
education_digital	0.02	[−0.22, 0.26]
exercise_digital+exercise_inperson+exercise_home	0.05	[−1.05, 1.19]
exercise_digital+psychological_inperson+exercise_home	0.10	[−1.20, 1.37]
exercise_digital+education_inperson+exercise_home	0.15	[−0.73, 1.16]
exercise_digital+usual_care+exercise_home	0.42	[−0.63, 1.58]
exercise_digital+physiotherapy+exercise_home	0.44	[−0.68, 1.68]
exercise_digital+psychological_digital+exercise_home	0.51	[−0.31, 1.43]
exercise_digital+exercise_home	0.52	[−0.25, 1.36]
education_digital+exercise_digital+exercise_home	0.54	[−0.22, 1.30]
psychological_digital+exercise_inperson+exercise_home	0.62	[−0.31, 1.58]
education_digital+exercise_inperson+exercise_home	0.65	[−0.24, 1.58]
psychological_digital+psychological_inperson+exercise_home	0.67	[−0.60, 1.87]
education_digital+psychological_inperson+exercise_home	0.70	[−0.50, 1.91]
psychological_digital+education_inperson+exercise_home	0.73	[−0.03, 1.55]
education_digital+education_inperson+exercise_home	0.76	[0.05, 1.46]
psychological_digital+usual_care+exercise_home	0.99	[0.11, 1.89]
psychological_digital+physiotherapy+exercise_home	1.02	[0.04, 2.04]
education_digital+usual_care+exercise_home	1.02	[0.19, 1.84]
exercise_home+physiotherapy	1.02	[0.12, 1.96]
education_digital+physiotherapy+exercise_home	1.04	[0.15, 1.90]
psychological_digital+exercise_home	1.09	[0.47, 1.72]
exercise_home	1.10	[0.54, 1.64]
education_digital+psychological_digital+exercise_home	1.12	[0.47, 1.80]
education_digital+exercise_home	1.12	[0.54, 1.69]

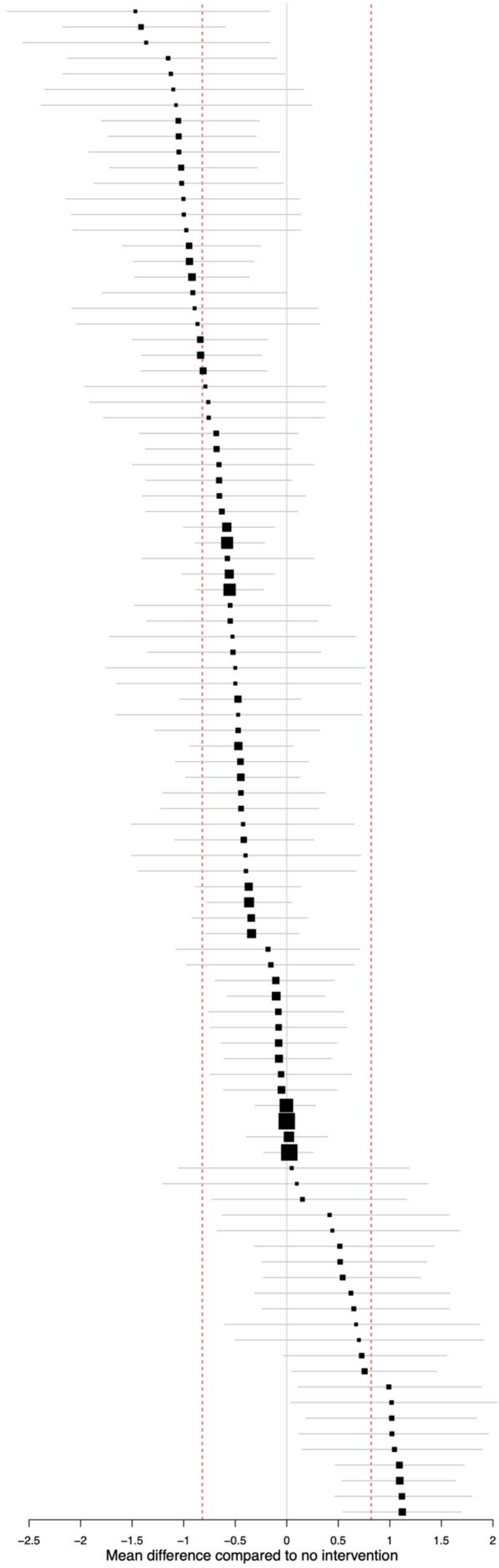

Supplemental Figure 31 – Forest plot of relative effects for the additive pain intensity immediate network for all clinically plausible interventions with 95%-credible intervals; red dotted lines represent the MCID of 0.82 for this outcome.

SUCRA table

Supplemental Table 15 - SUCRA table of the additive pain intensity immediate network for all clinically plausible interventions

Intervention	Mean Rank	2.5% CI	97.5% CI	SUCRA
exercise_digital+education_inperson+exercise_inperson	5.80	1.00	22.00	0.95
exercise_digital+exercise_inperson+psychological_inperson	7.45	1.00	45.03	0.93
exercise_digital+education_inperson+psychological_inperson	9.49	1.00	47.00	0.91
exercise_digital+exercise_inperson+usual_care	13.51	1.00	47.03	0.87
exercise_digital+exercise_inperson+physiotherapy	15.11	1.00	52.00	0.85
exercise_digital+psychological_digital+exercise_inperson	15.67	2.00	42.03	0.85
exercise_digital+exercise_inperson	15.93	3.00	42.00	0.85
education_digital+exercise_digital+exercise_inperson	16.66	3.00	43.00	0.84
exercise_digital+education_inperson+usual_care	16.83	1.00	49.00	0.84
exercise_digital+psychological_inperson+usual_care	17.43	2.00	61.00	0.83
exercise_digital+education_inperson+physiotherapy	17.49	2.00	51.00	0.83
exercise_digital+psychological_inperson+physiotherapy	18.79	2.00	64.00	0.82
exercise_digital+psychological_digital+education_inperson	19.35	3.00	43.00	0.81
exercise_digital+education_inperson	19.70	4.00	46.00	0.81
exercise_digital+psychological_digital+psychological_inperson	20.44	3.00	60.00	0.80
education_digital+exercise_digital+education_inperson	20.58	3.00	45.00	0.80
exercise_digital+psychological_inperson	20.74	4.00	62.00	0.80
education_digital+exercise_digital+psychological_inperson	21.56	4.00	63.00	0.79
education_inperson+exercise_inperson+physiotherapy	22.09	4.00	53.00	0.78
psychological_digital+education_inperson+exercise_inperson	24.23	6.00	49.00	0.76
education_inperson+exercise_inperson	24.61	7.00	50.00	0.76
psychological_digital+exercise_inperson+psychological_inperson	25.33	3.00	68.00	0.75
education_digital+education_inperson+exercise_inperson	25.70	6.00	51.00	0.75
education_digital+exercise_inperson+psychological_inperson	26.68	2.00	70.00	0.74
exercise_digital+physiotherapy+usual_care	29.92	5.00	70.00	0.70
psychological_digital+education_inperson+psychological_inperso n	30.05	3.00	73.00	0.70
education_digital+education_inperson+psychological_inperson	31.35	3.00	74.03	0.69
exercise_digital+psychological_digital+usual_care	32.39	9.00	58.00	0.68

Intervention	Mean Rank	2.5% CI	97.5% CI	SUCRA
exercise_digital+usual_care	32.88	10.00	60.00	0.67
education_digital+exercise_digital+usual_care	33.98	9.00	60.00	0.66
exercise_digital+psychological_digital+physiotherapy	34.22	8.00	65.00	0.66
exercise_digital+physiotherapy	34.58	10.00	63.00	0.65
education_digital+exercise_digital+physiotherapy	35.64	11.00	60.00	0.64
exercise_digital+psychological_digital	37.64	15.00	57.00	0.62
exercise_digital	38.07	16.00	60.00	0.62
psychological_digital+exercise_inperson+usual_care	38.57	11.00	68.03	0.61
education_digital+exercise_digital+psychological_digital	38.84	15.00	57.00	0.61
education_digital+exercise_digital	39.26	17.00	58.00	0.61
education_digital+exercise_inperson+usual_care	40.01	11.00	71.00	0.60
psychological_digital+exercise_inperson+physiotherapy	40.21	10.00	72.00	0.60
education_digital+exercise_inperson+physiotherapy	41.68	12.00	71.00	0.58
psychological_digital+psychological_inperson+usual_care	42.41	11.00	80.00	0.57
education_digital+psychological_inperson+usual_care	43.70	10.00	82.00	0.56
psychological_digital+psychological_inperson+physiotherapy	43.78	10.00	81.00	0.56
psychological_digital+education_inperson+usual_care	43.88	12.00	73.00	0.56
psychological_digital+exercise_inperson	44.02	17.00	68.00	0.56
exercise_inperson	44.34	19.00	69.00	0.55
education_digital+psychological_inperson+physiotherapy	45.06	12.00	81.00	0.55
education_digital+education_inperson+usual_care	45.26	11.00	75.00	0.54
education_digital+psychological_digital+exercise_inperson	45.27	16.00	71.00	0.54
psychological_digital+education_inperson+physiotherapy	45.51	14.98	71.00	0.54
education_digital+exercise_inperson	45.61	16.00	70.00	0.54
education_digital+education_inperson+physiotherapy	47.06	18.98	70.00	0.53
psychological_digital+psychological_inperson	47.35	12.00	83.00	0.52
education_digital+psychological_digital+psychological_inperson	48.49	12.00	85.00	0.51
education_digital+psychological_inperson	48.57	13.00	86.00	0.51
psychological_digital+education_inperson	49.27	18.00	73.00	0.50
education_inperson	49.46	19.00	74.00	0.50
education_digital+psychological_digital+education_inperson	50.46	17.00	76.00	0.49

Intervention	Mean Rank	2.5% CI	97.5% CI	SUCRA
education_digital+education_inperson	50.68	18.00	75.00	0.49
psychological_digital+physiotherapy+usual_care	58.81	29.00	80.00	0.40
education_digital+physiotherapy+usual_care	60.32	30.00	80.03	0.39
usual_care	63.03	39.00	79.00	0.36
psychological_digital+usual_care	63.04	40.00	79.00	0.36
psychological_digital+physiotherapy	63.74	38.00	80.00	0.35
education_digital+psychological_digital+usual_care	63.89	36.00	83.00	0.35
physiotherapy	64.04	40.98	78.00	0.35
education_digital+usual_care	64.10	38.00	82.00	0.35
exercise_digital+exercise_inperson+exercise_home	64.58	25.00	83.00	0.34
education_digital+psychological_digital+physiotherapy	64.92	37.00	82.00	0.34
education_digital+physiotherapy	65.43	43.00	80.00	0.34
exercise_digital+psychological_inperson+exercise_home	66.49	23.00	93.00	0.32
no_intervention	67.11	44.00	83.00	0.32
psychological_digital	67.19	44.00	83.00	0.32
education_digital+psychological_digital	68.17	42.00	85.00	0.31
education_digital	68.22	41.00	85.00	0.31
exercise_digital+education_inperson+exercise_home	69.25	38.00	83.00	0.30
exercise_digital+usual_care+exercise_home	76.78	49.00	91.00	0.22
exercise_digital+physiotherapy+exercise_home	77.04	46.00	93.00	0.22
exercise_digital+psychological_digital+exercise_home	79.78	62.98	87.00	0.19
exercise_digital+exercise_home	79.87	62.00	88.00	0.19
education_digital+exercise_digital+exercise_home	80.66	66.00	87.00	0.18
psychological_digital+exercise_inperson+exercise_home	82.08	61.00	93.00	0.16
psychological_digital+psychological_inperson+exercise_home	82.61	50.00	97.00	0.16
education_digital+exercise_inperson+exercise_home	82.87	65.00	94.00	0.16
education_digital+psychological_inperson+exercise_home	83.24	52.00	97.00	0.15
psychological_digital+education_inperson+exercise_home	84.78	72.00	94.00	0.14
education_digital+education_inperson+exercise_home	85.42	73.00	95.00	0.13
psychological_digital+usual_care+exercise_home	90.35	80.00	97.00	0.08
psychological_digital+physiotherapy+exercise_home	90.66	79.00	97.00	0.08

Intervention	Mean Rank	2.5% CI	97.5% CI	SUCRA
exercise_home+physiotherapy	90.82	81.00	97.00	0.07
education_digital+usual_care+exercise_home	90.83	80.00	97.00	0.07
education_digital+physiotherapy+exercise_home	91.44	82.00	97.00	0.07
psychological_digital+exercise_home	92.46	86.00	97.00	0.06
exercise_home	92.47	86.00	97.00	0.06
education_digital+psychological_digital+exercise_home	92.89	85.00	97.00	0.05
education_digital+exercise_home	93.01	86.00	97.00	0.05

Supplemental Data 21 – Narrative summary of adverse events

Supplemental Table 16 – Narrative summary of adverse events

Study	Definition	Outcome
Petrozzi 2019	"A serious adverse event from physical treatment was defined as any untoward occurrence resulting in hospitalization, life threatening injury that results in persistent significant disability or incapacity or death."<br><br>"An enquiry for any minor, moderate or severe physical treatment adverse event was made by the practitioner at each treatment session investigating: 1) a new related complaint which was not present at baseline or previous visit, or 2) a worsening of the presenting complaint. Adverse events from physical treatments were measured at each treatment by two questions which the participant self-scored on a 10-point scale from 1(Not at all) to 10 (Extreme increased pain)."	No serious adverse event was reported.<br><br>Mild to moderate temporarily limited pain increase was noted in 51 cases in the intervention group and 72 cases in the control group.<br><br>No participant discontinued the intervention due to adverse events.<br><br>(Information obtained from the authors upon request)
Rutledge 2018	Not defined	The authors reported no adverse events.
Amorim 2019	Not defined	The authors reported no adverse events.
Day 2024	"any negative effects that they attributed to the treatment practice or homework"	Two adverse events (falls with increased pain) related to the intervention (education digital). All other reported adverse events (278) are not reported per group.
del Pozo-Cruz 2012	Not defined	The authors reported no adverse events.
Garreta-Catala 2013	Not defined	The authors reported no adverse events.
Geraghty 2018	Not defined	Two hospital admissions in each group, unclear whether they were related to the intervention.
Goode 2018	"Adverse events were defined by protocol as any untoward physical or psychological occurrence by participating in research."	The authors reported no study-related adverse events.
Krein 2013	"Both groups were encouraged to report any health problems via the website, email, or phone. Four weeks after randomization and every 8 weeks	"During the study, approximately 600 adverse events were reported by participants (250 by those

	thereafter, participants were prompted to complete a survey that asked about specific adverse events (eg, heart attack) and symptoms such as shortness of breath."	in usual care and nearly 350 by those in the intervention). These events ranged from calluses to chest pain. Worsening back pain, the most frequently reported event, accounted for 29% of events reported by the usual care group and 25% of those reported by the intervention group. Overall, more musculoskeletal events (n=112) were reported than cardiovascular events (n=85), and musculoskeletal injuries were more likely to be reported by participants in the intervention group compared to those in usual care. However, no major study-related adverse events (eg, heart attack) were identified for either group."
López-Marcos 2024	Not defined	The authors reported no adverse events.
Sanabria-Mazo 2023	"The Adverse Effects of Treatments was used to measure the potential presence of negative effects of ACT and BATD. This ad hoc instrument is composed of 1 item ("Have you experienced, during the psychological treatment, any unwanted symptom that you think might be directly or indirectly associated with the psychological intervention?"), with a "yes" or "no" answer option. Previous RCTs have used this question to explore adverse events (eg, headaches, dizziness, sleep problems, etc.) across the interventions.	"In total, 6 patients in the ACT group and 2 in the BATD group reported unpleasant events at posttreatment.<br>In the ACT group, 5 patients described increased emotional distress (depressive, anxiety, or stress symptoms) during body awareness exercises and 1 patient reported increased pain at the end of one therapeutic exercise.<br>In the case of the BATD group, 2 patients mentioned an increase in depressive and anxiety symptoms after the end of the therapy sessions."
Sandal 2021	Not defined	The authors reported no adverse events.
Toelle 2019	Not defined	One patient in the intervention group had a new diagnosis of lumbar disc herniation, which was an incidental finding without new symptoms.
Williams 2018	"any new medical conditions or an exacerbation of another condition"	32 participants in the intervention group and 45 participants in the control group showed an adverse event, reported with ICD-10 codes, not clear whether intervention-related or not.
Zheng 2022 a	Not defined	The authors reported no adverse events.

Zheng 2022 b	Not defined	The authors reported no adverse events.
Licciardone 2020	Not defined	The authors reported no adverse events.
Licciardone 2022	Not defined	The authors reported no serious adverse events.
Cui 2023	Serious adverse events were defined based on the U.S. Food and Drug Administration.	Based on our own definitions in this review, the intervention group had 1 serious adverse event (brain bleeding) and 10 non-serious adverse events, while the control group had 6 non-serious adverse events. None of the adverse events were related to the intervention.
Geraghty 2024	Not defined	"There were seven unrelated serious adverse events reported during the trial; three in the usual care group, one in the SupportBack group, and three in the SupportBack with telephone-support group (…).<br>These serious adverse events included an unrelated death in the SupportBack with telephone-support group. There were also seven adverse events reported that did not meet seriousness criteria. These included five operations deemed non-serious by a lead clinician at the practice (three in the SupportBack with telephone-support group and two in the SupportBack group), one report of increased back pain following a car accident (SupportBack group), and one report of increased leg-to-ankle pain which the participant stated stopped them taking part in the intervention as they would have liked (SupportBack with telephone-support)."
Yang 2024	Not defined	"During the treatment period, 2 (1.6%) of 124 participants attending1or more of the 24 sessions reported an adverse event: one started having pain in the forearm after treatment started; the other had shoulder pain possibly due to overdoing shoulder rotations. The pain in both cases was minor and transient."

Supplemental Data 22 – Transitivity assessment

Disability immediate

Boxplot of baseline pain intensity by treatment

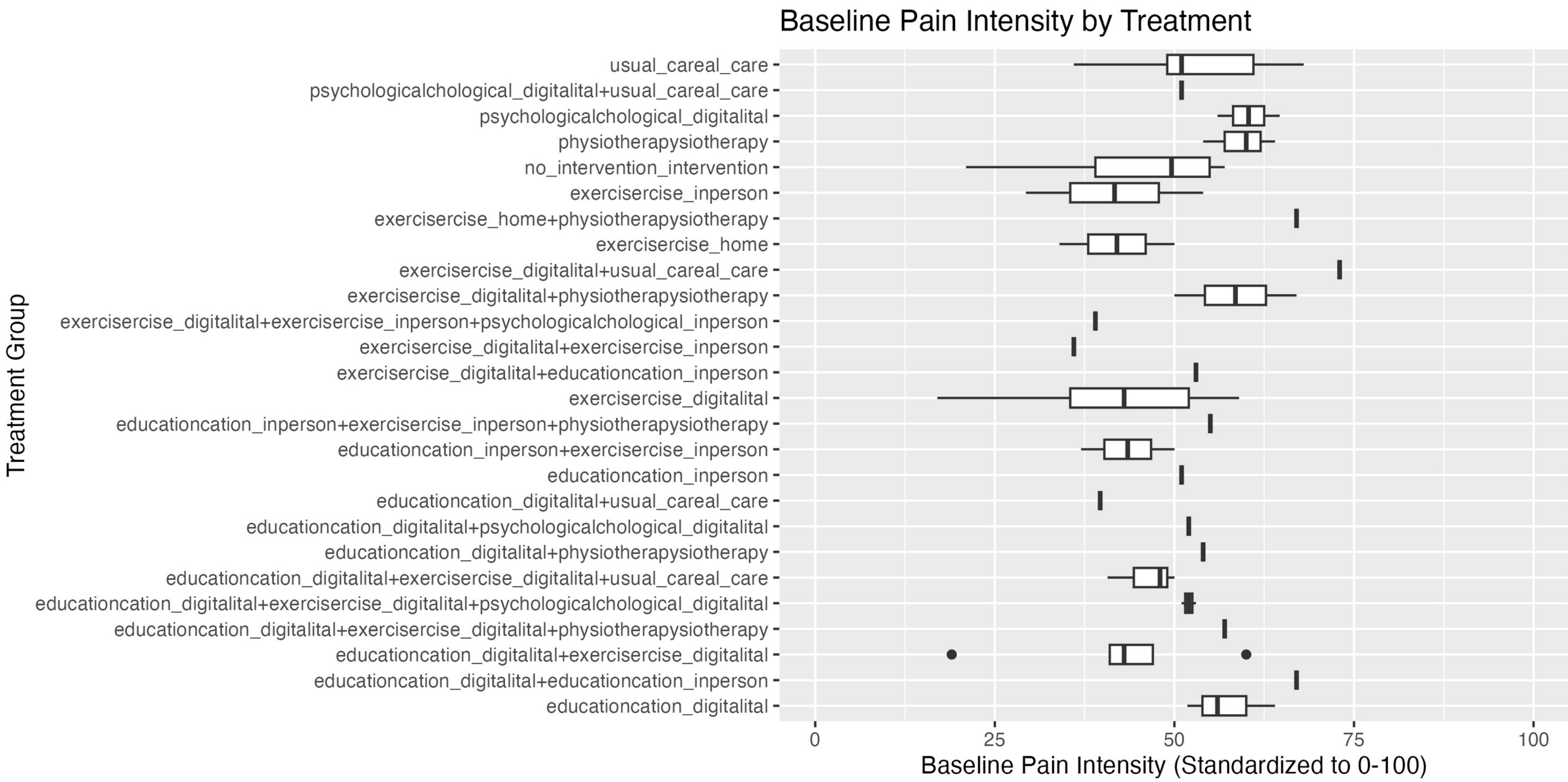

*Supplemental Figure 32 – Boxplot of baseline pain intensity by treatment for the disability immediate network. Values are standardized to a 0-100 scale. Boxes represent the median (vertical line) and quartiles (ends of boxes). The notches display the confidence interval around the median which is based on the median +/- 1.58*IQR/sqrt(n).*

Boxplot of baseline pain intensity by treatment comparison

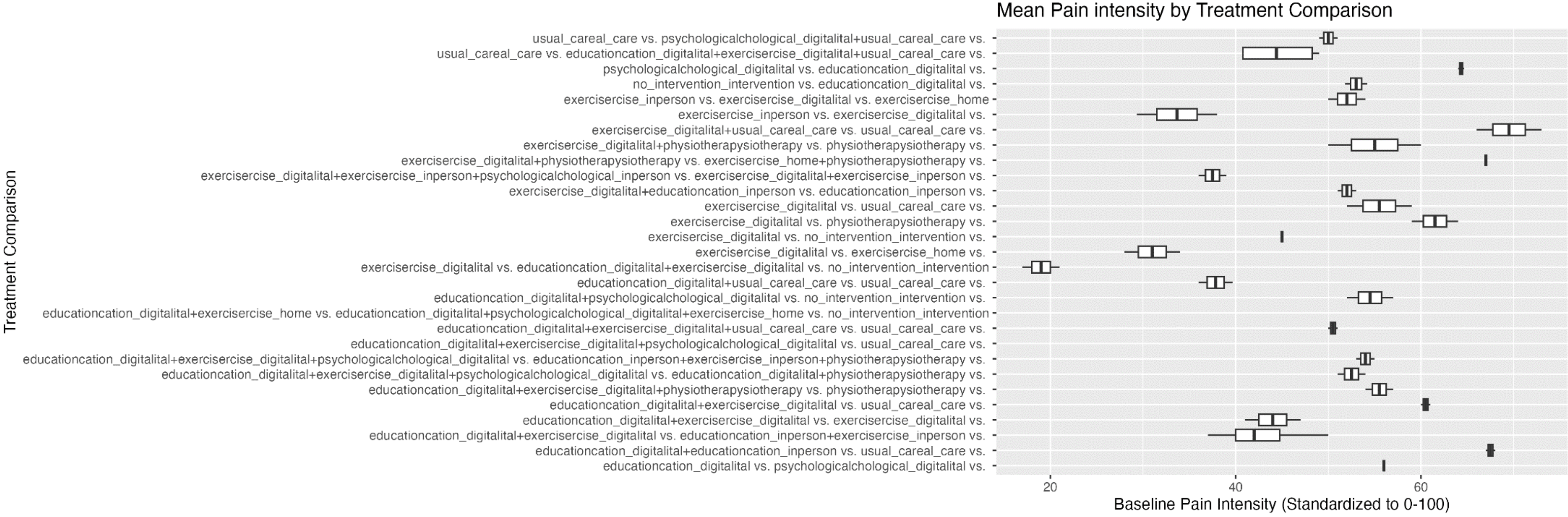

*Supplemental Figure 33 – Boxplot of baseline pain intensity by treatment comparison for the disability immediate network. Values are standardized to a 0-100 scale. Boxes represent the median (vertical line) and quartiles (ends of boxes). The notches display the confidence interval around the median which is based on the median +/- 1.58*IQR/sqrt(n).*

Boxplot of baseline disability by treatment

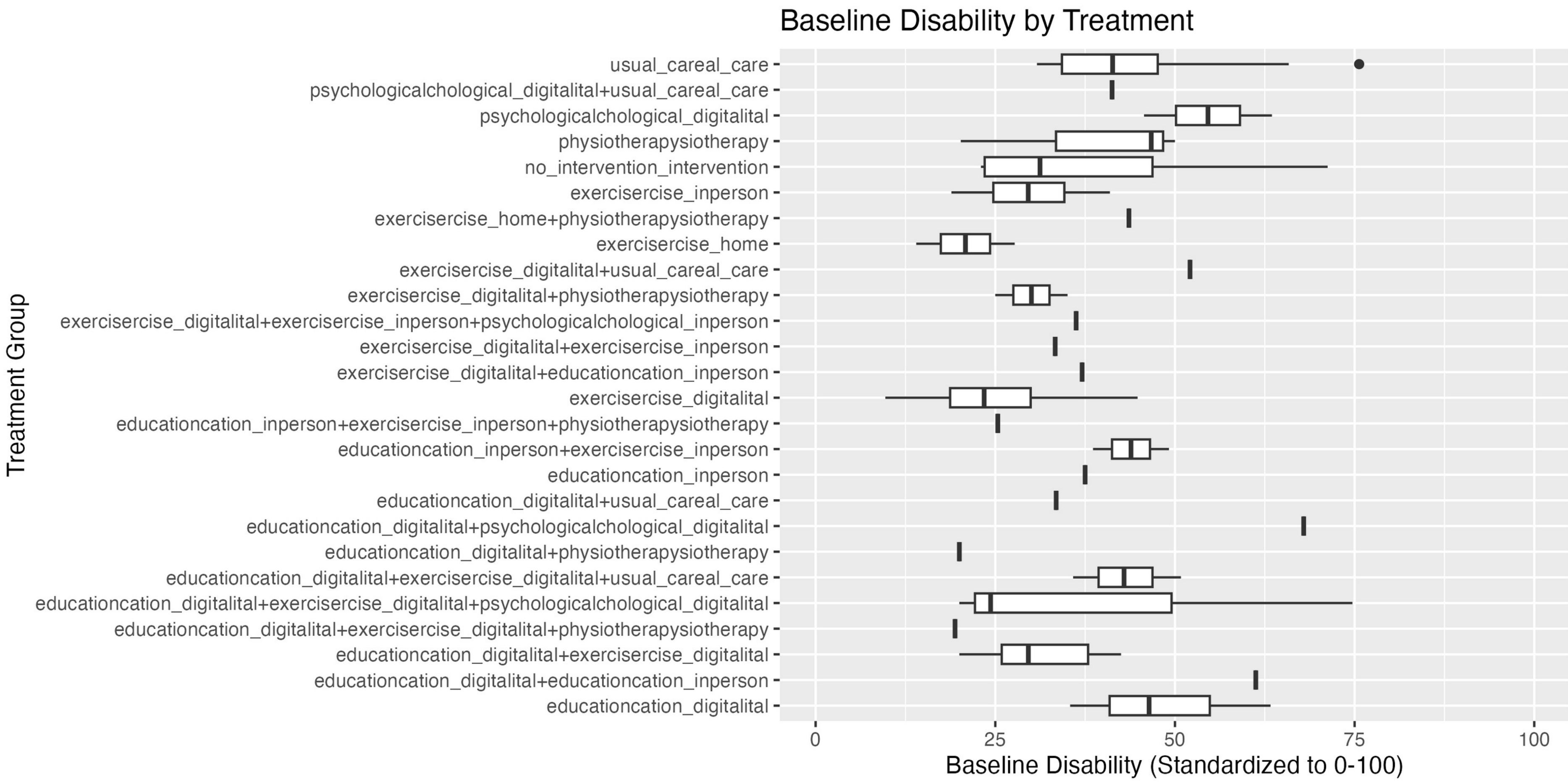

*Supplemental Figure 34 – Boxplot of baseline disability by treatment for the disability immediate network. Values are standardized to a 0-100 scale. Boxes represent the median (vertical line) and quartiles (ends of boxes). The notches display the confidence interval around the median which is based on the median +/- 1.58*IQR/sqrt(n).*

Boxplot of baseline disability by treatment comparison

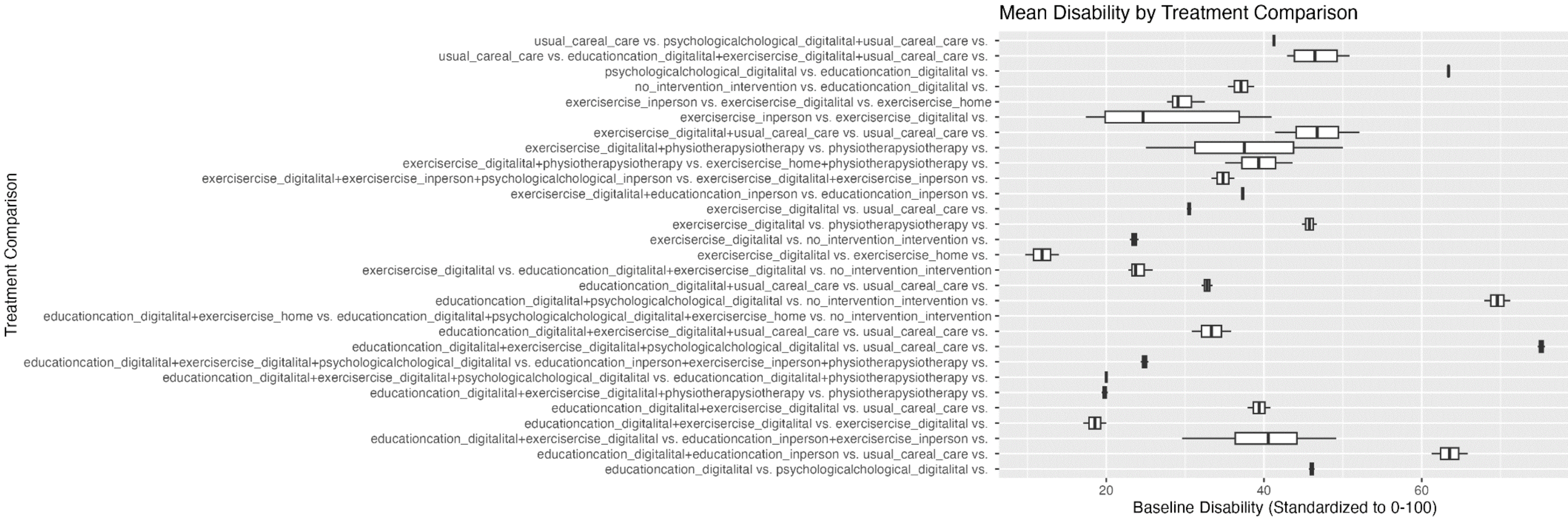

*Supplemental Figure 35 – Boxplot of baseline disability by treatment comparison for the disability immediate network. Values are standardized to a 0-100 scale. Boxes represent the median (vertical line) and quartiles (ends of boxes). The notches display the confidence interval around the median which is based on the median +/- 1.58*IQR/sqrt(n).*

Boxplot of mean age by treatment

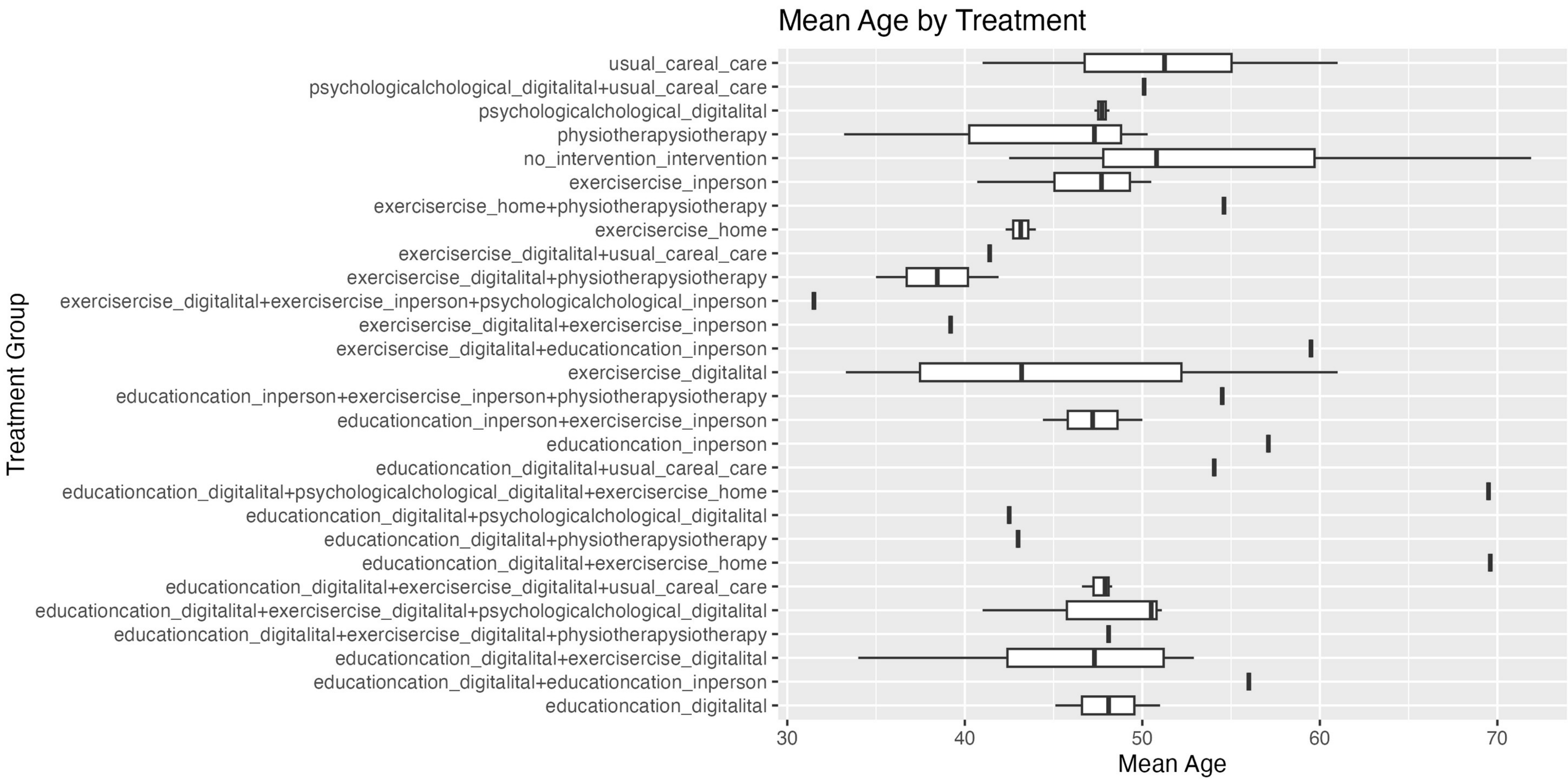

*Supplemental Figure 36 – Boxplot of mean age by treatment for the disability immediate network. Boxes represent the median (vertical line) and quartiles (ends of boxes). The notches display the confidence interval around the median which is based on the median +/- 1.58*IQR/sqrt(n).*

Boxplot of mean age by treatment comparison

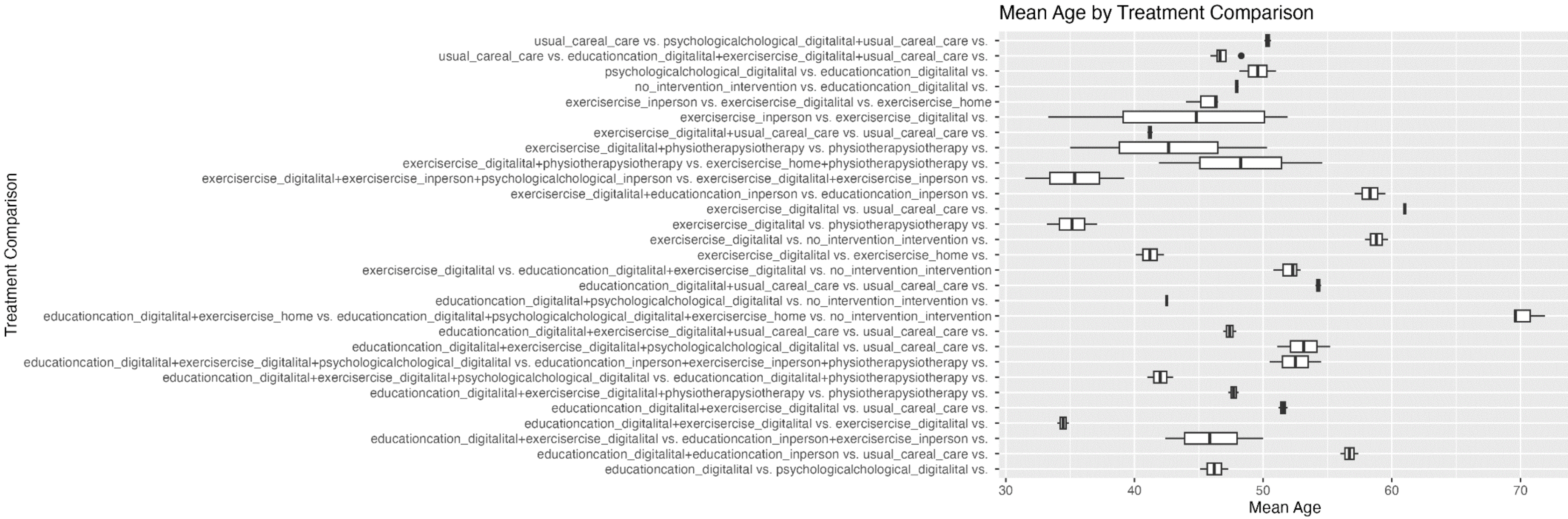

*Supplemental Figure 37 – Boxplot of mean age by treatment comparison for the disability immediate network. Boxes represent the median (vertical line) and quartiles (ends of boxes). The notches display the confidence interval around the median which is based on the median +/- 1.58*IQR/sqrt(n).*

Network meta-regression

Supplemental Table 17 – Network meta-regression results of the disability immediate network, incl. comparison of modelfit paramters

Model	Number of effective parameters	Posterior mean of between-study SD	Residual Deviance	DIC	B	95% CrI of B
CNMA Base Model	62.38	0.41	68.38	252.07		
Meta-Regression Baseline Disability	62.79	0.42	68.80	252.39	0.00	-0.02;0.02
digital					0.01	-0.01;0.03
hybrid					-0.01	-0.04;0.03
inperson					0.01	-0.02;0.03
Meta-Regression Age	62.33	0.42	68.69	251.83	0.02	-0.08;0.11
digital					0.02	-0.07;0.1
hybrid					-0.02	-0.11;0.08
inperson					0.03	-0.06;0.12

DIC = deviance information criterion, CrI = credible interval

Tau-plot of random-effect pairwise meta-analyses

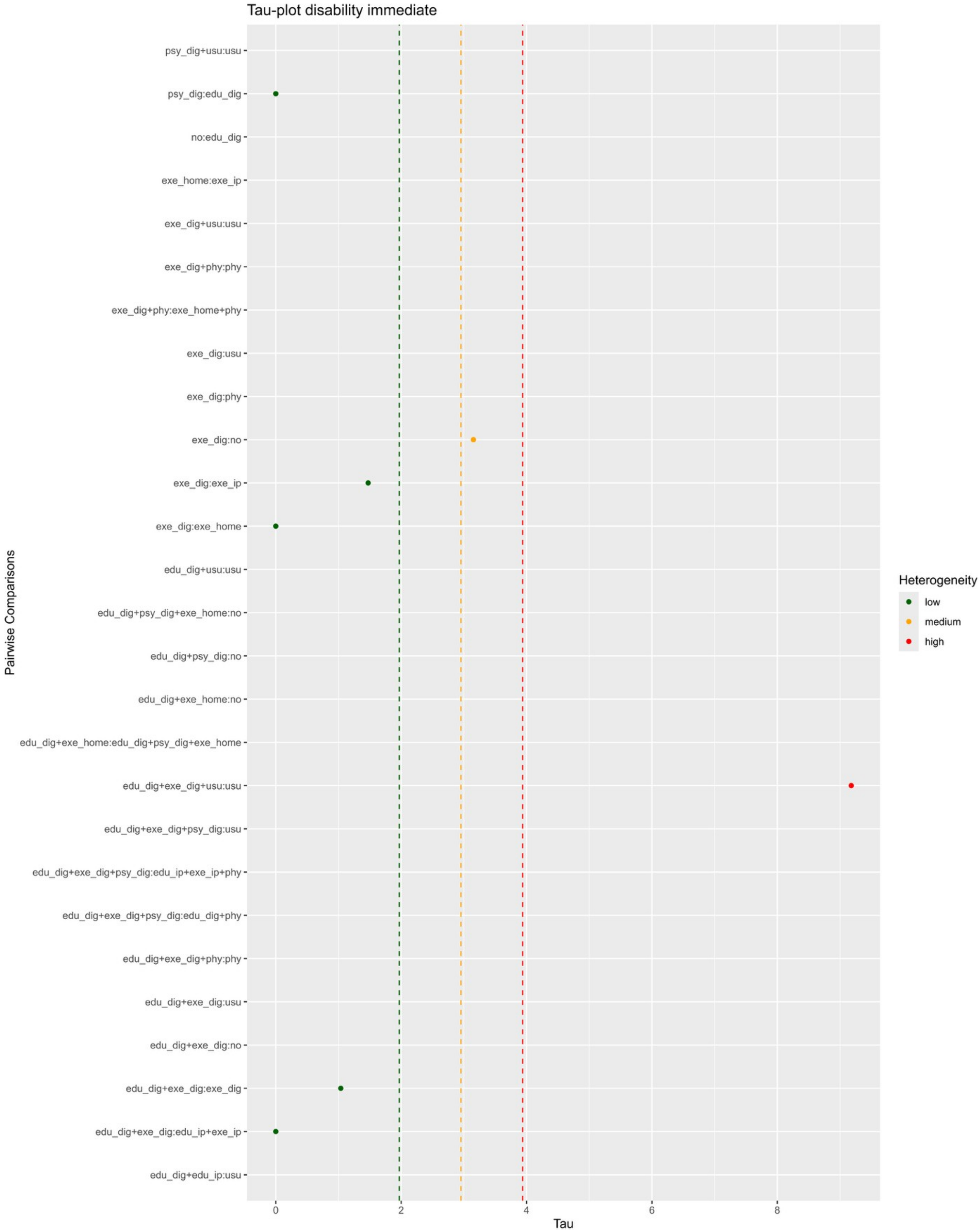

Supplemental Figure 38 – Tau plot of pairwise comparisons in the disability immediate network. Data is back transformed to the scale of the Oswestry Disability Index. Colored lines represent limits for low, medium and high heterogeneity.

Pain intensity immediate

Boxplot of baseline pain intensity by treatment

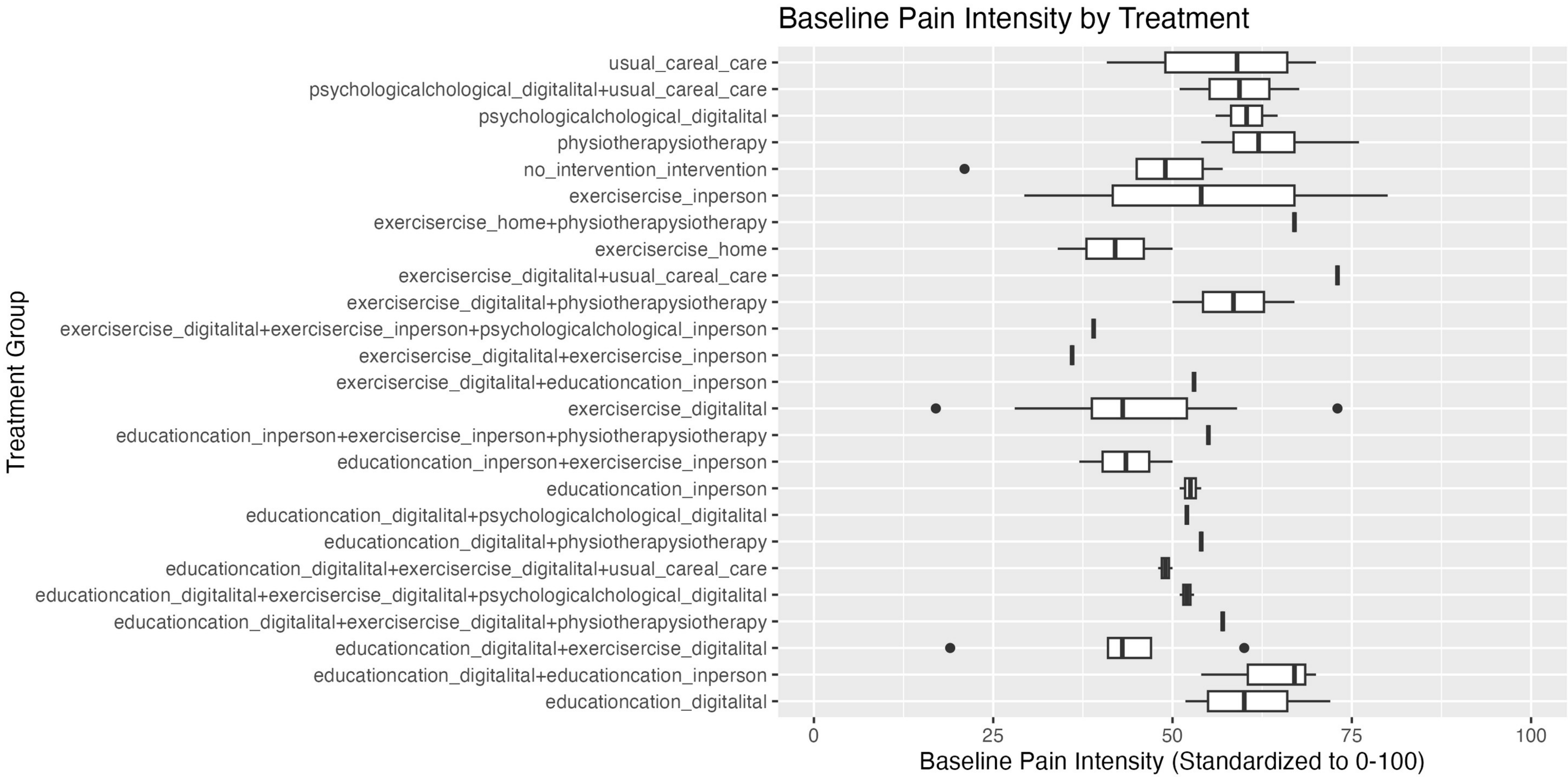

*Supplemental Figure 39 – Boxplot of baseline pain intensity by treatment for the pain intensity immediate network. Values are standardized to a 0-100 scale. Boxes represent the median (vertical line) and quartiles (ends of boxes). The notches display the confidence interval around the median which is based on the median +/- 1.58*IQR/sqrt(n).*

Boxplot of baseline pain intensity by treatment comparison

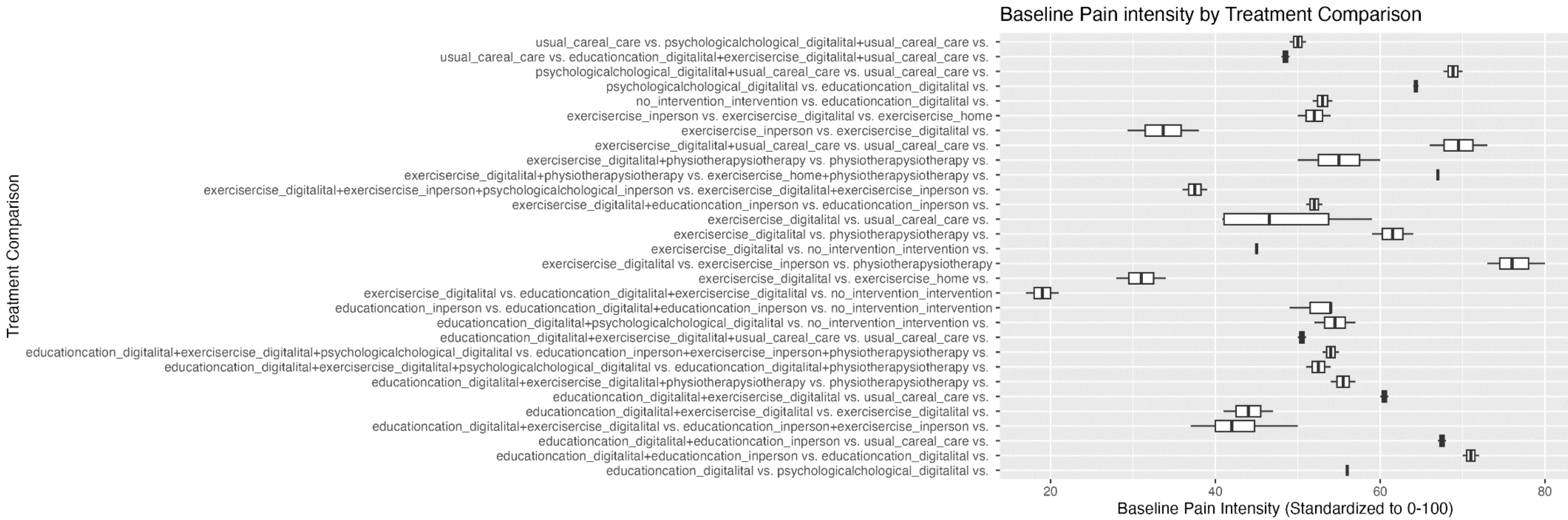

*Supplemental Figure 40 – Boxplot of baseline pain intensity by treatment comparison for the pain intensity immediate network. Values are standardized to a 0-100 scale. Boxes represent the median (vertical line) and quartiles (ends of boxes). The notches display the confidence interval around the median which is based on the median +/- 1.58*IQR/sqrt(n).*

Boxplot of baseline disability by treatment

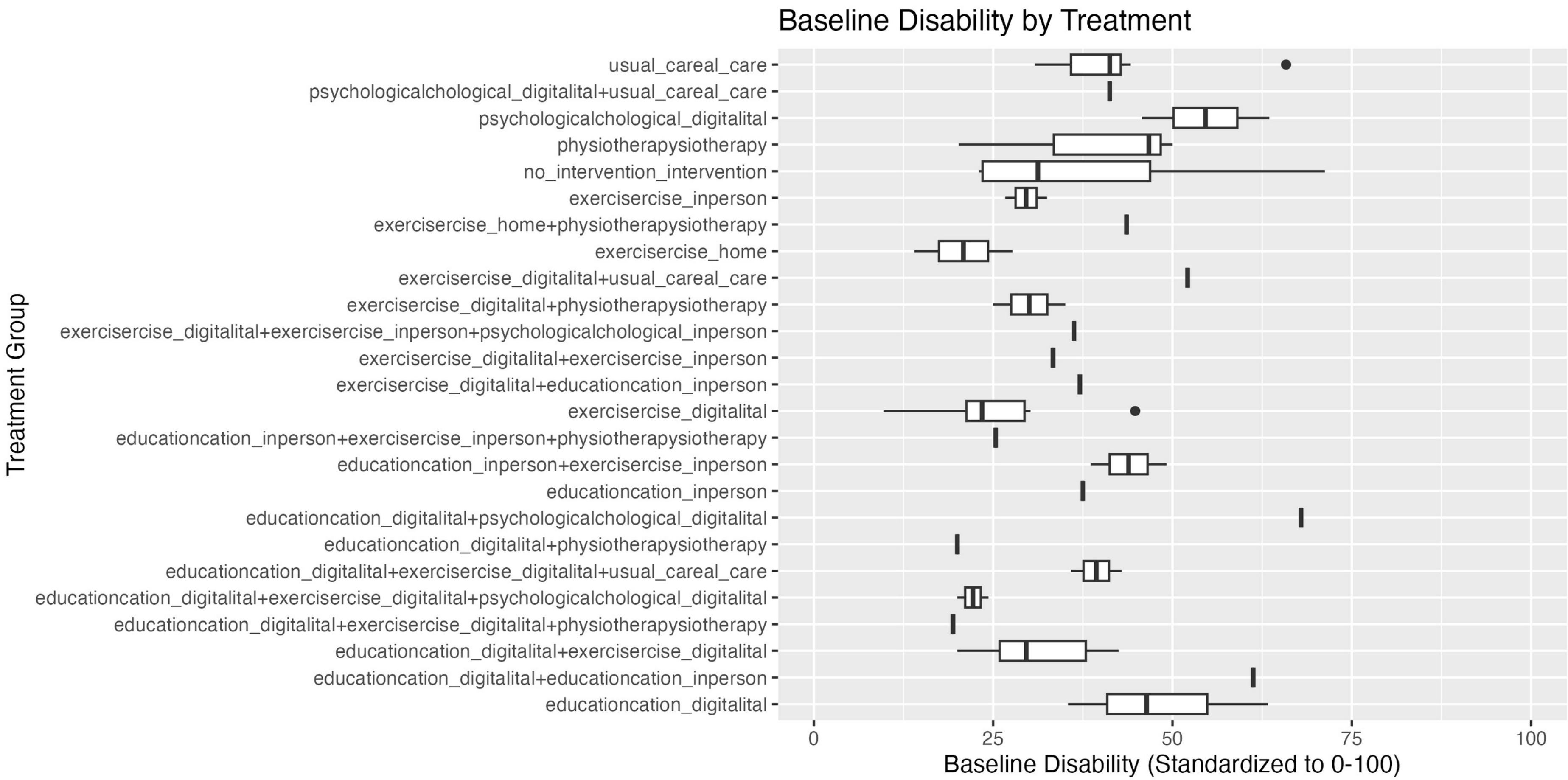

*Supplemental Figure 41 – Boxplot of baseline disability by treatment for the pain intensity immediate network. Values are standardized to a 0-100 scale. Boxes represent the median (vertical line) and quartiles (ends of boxes). The notches display the confidence interval around the median which is based on the median +/- 1.58*IQR/sqrt(n).*

Boxplot of baseline disability by treatment comparison

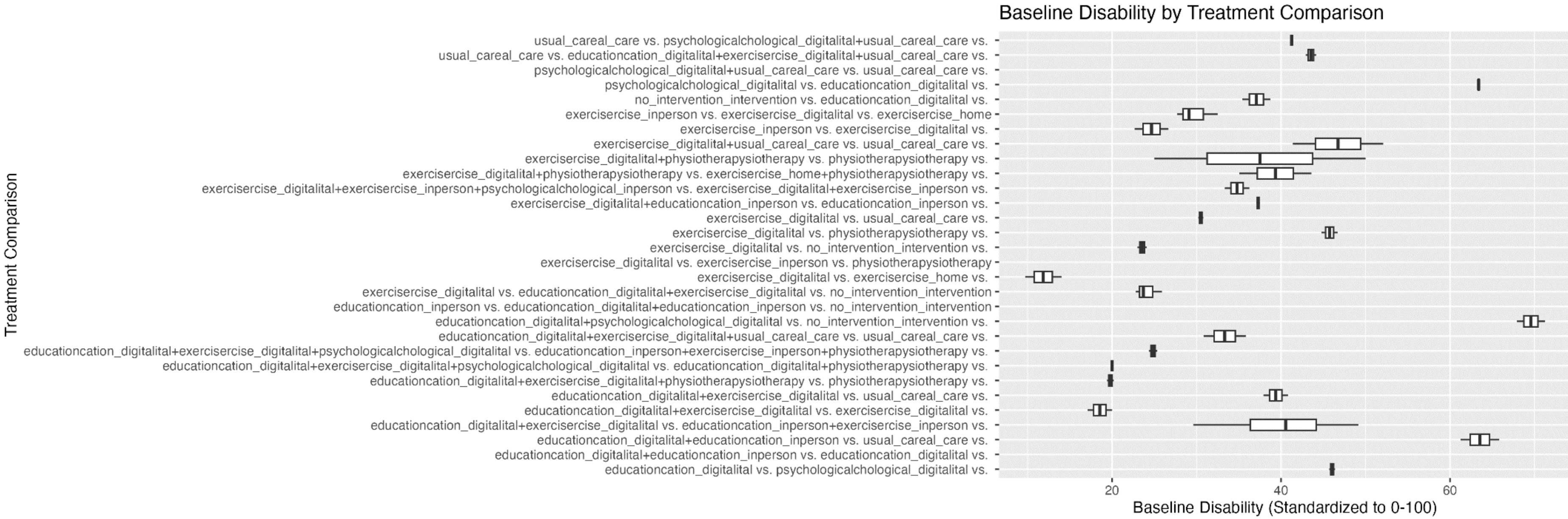

*Supplemental Figure 42 – Boxplot of baseline disability by treatment comparison for the pain intensity immediate network. Values are standardized to a 0-100 scale. Boxes represent the median (vertical line) and quartiles (ends of boxes). The notches display the confidence interval around the median which is based on the median +/- 1.58*IQR/sqrt(n).*

Boxplot of mean age by treatment

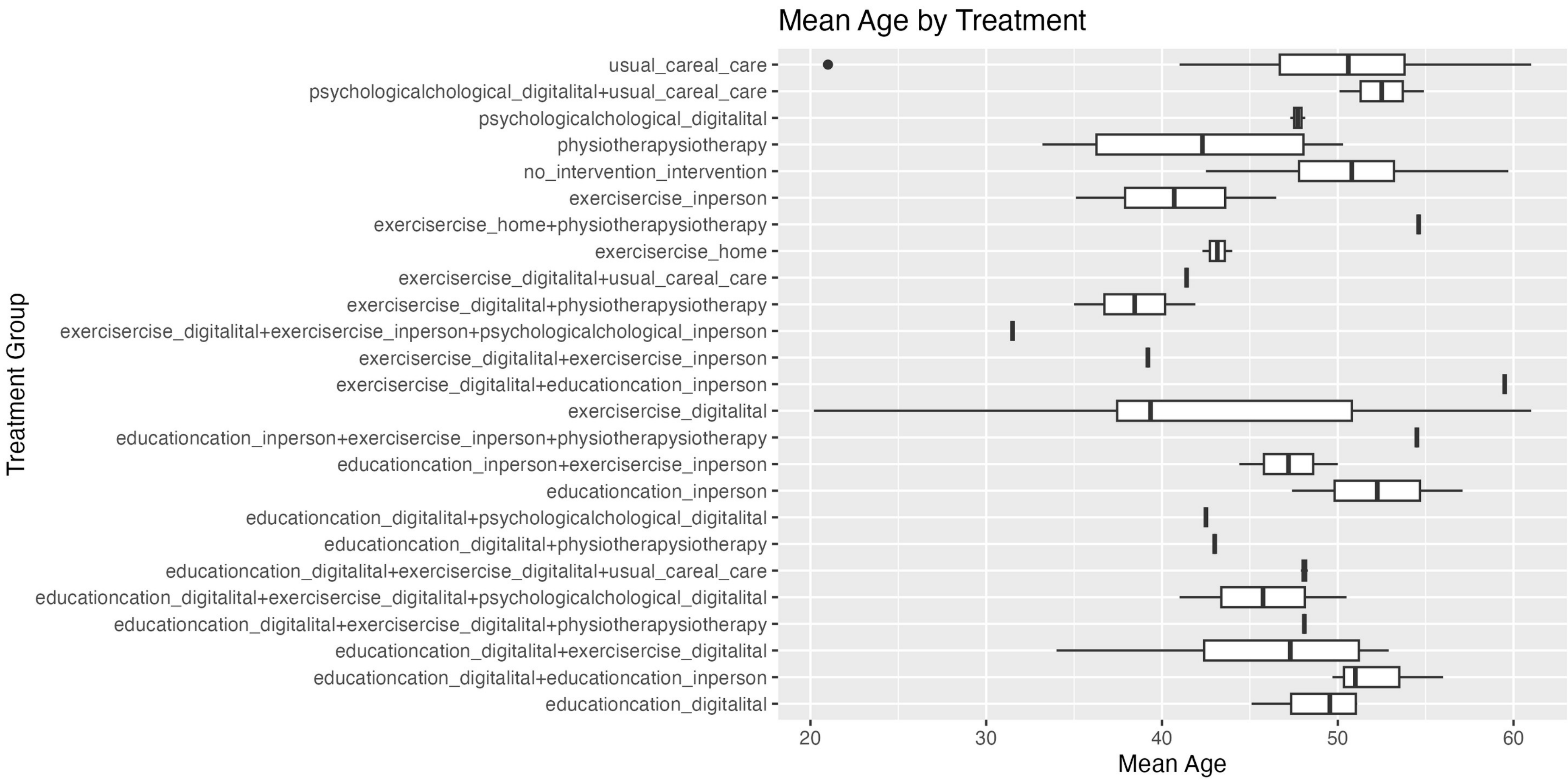

*Supplemental Figure 43 – Boxplot of mean age by treatment for the pain intensity immediate network. Boxes represent the median (vertical line) and quartiles (ends of boxes). The notches display the confidence interval around the median which is based on the median +/- 1.58*IQR/sqrt(n).*

Boxplot of mean age by treatment comparison

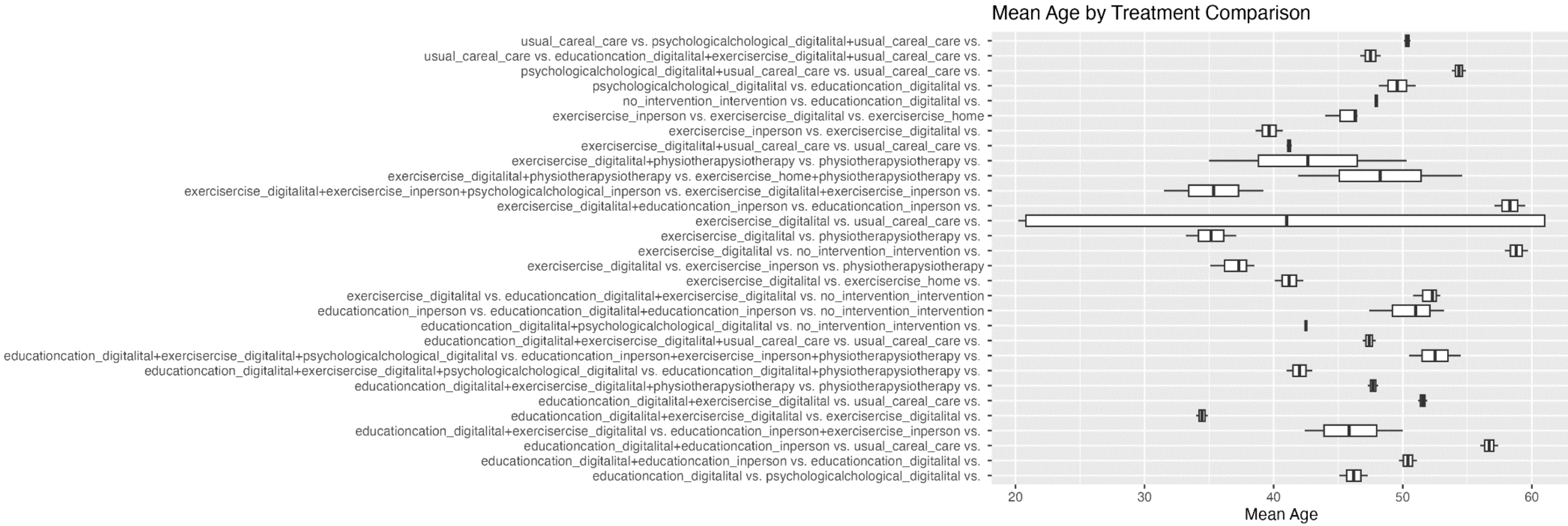

*Supplemental Figure 44 – Boxplot of mean age by treatment for the pain intensity immediate network. Boxes represent the median (vertical line) and quartiles (ends of boxes). The notches display the confidence interval around the median which is based on the median +/- 1.58*IQR/sqrt(n).*

Network meta-regression

Supplemental Table 18 – Network meta-regression results of the pain intensity immediate network, incl. comparison of modelfit paramters

Model	Number of effective parameters	Posterior mean of between-study SD	Residual Deviance	DIC	B	95% CrI of B
CNMA Base Model	49.54	0.13	69.73	68.63		
Meta-Regression Baseline Pain Intensity	50.42	0.14	70.11	69.70	-0.00	-0.03;0.02
digital					-0.00	-0.03;0.02
hybrid					-0.01	-0.04;0.02
inperson					-0.01	-0.04;0.02
Meta-Regression Age	50.87	0.14	69.56	69.68	0.01	-0.03;0.05
digital					0.02	-0.02;0.06
hybrid					0.04	-0.01;0.1
inperson					0.04	-0.01;0.08

DIC = deviance information criterion, CrI = credible interval

Tau plot of random effect pairwise meta-analyses

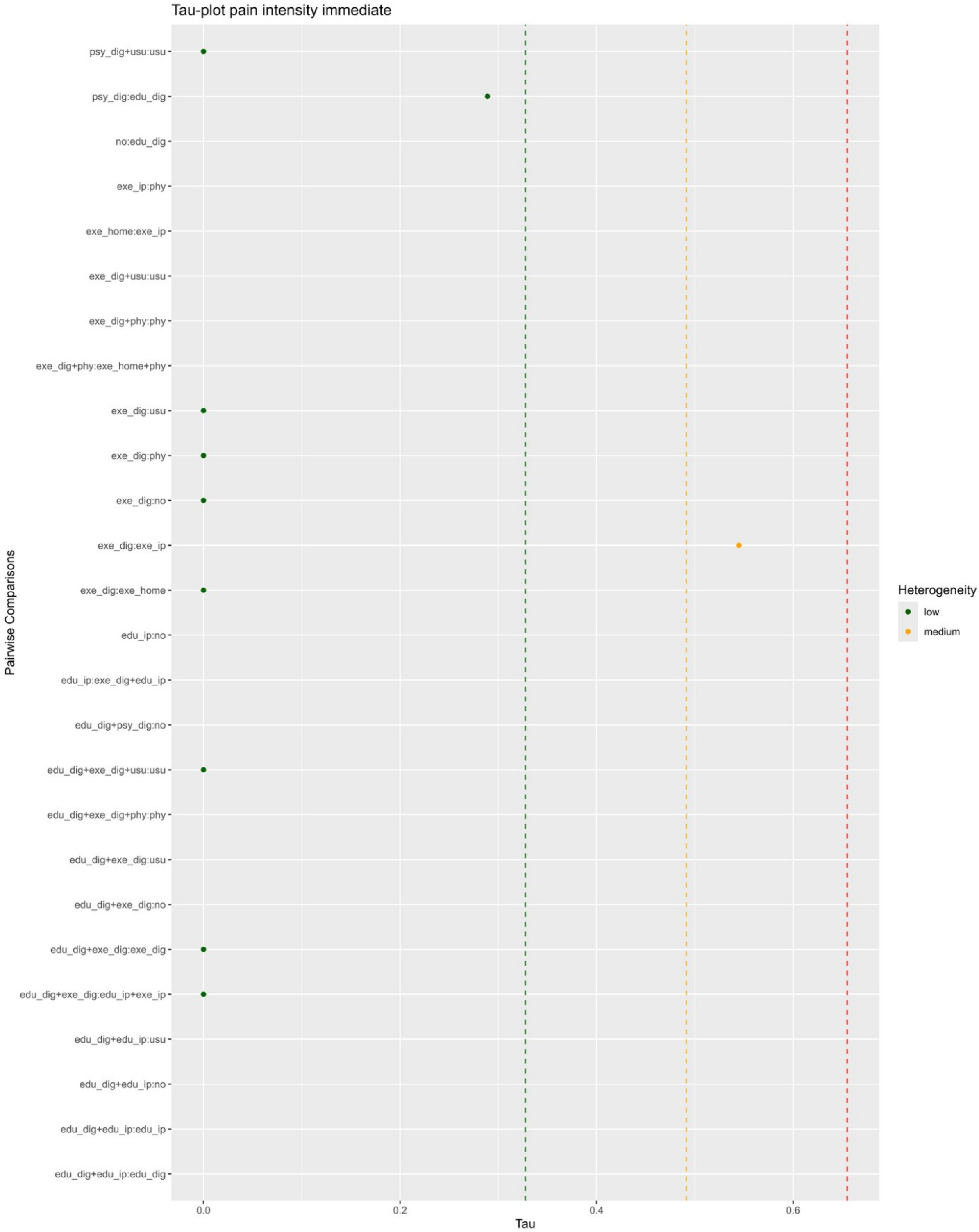

Supplemental Figure 45 – Tau plot of pairwise comparisons in the pain intensity immediate network. Data is back transformed to the scale of the Oswestry Disability Index. Colored lines represent limits for low heterogeneity

Supplemental Data 23 – Inconsistency assessment

Disability immediate

Comparison between UME and standard NMA model

Supplemental Table 19 – Comparison of modelfit parameters between the unrelated mean effects and standard NMA model

Model	Posterior mean of between-study SD	Residual Deviance	DIC	Difference in Residual Deviances	Difference in DICs
NMA	0.532	64.502	128.081	-0.227	-0.645
UME	0.569	64.729	128.726		

UME = unrelated mean effects, DIC = deviance information criterion

dev-dev plot

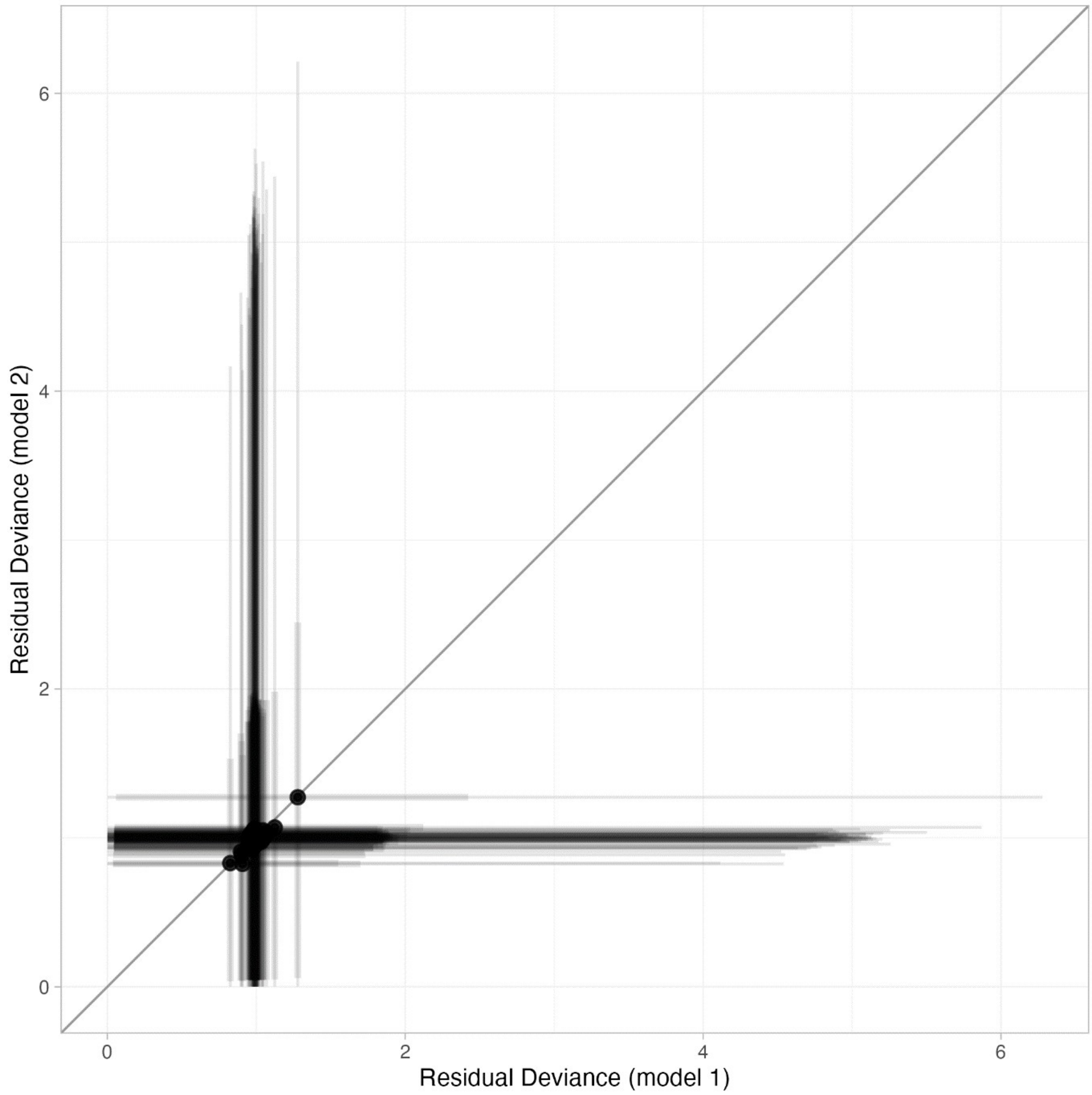

Supplemental Figure 46 – Deviance contribution plot for the disability immediate network. Model 1 represents the single-effect NMA model, model 2 represents the unrelated mean effects model. If the points are close to the line of equity, consistency between the two models is assumed.

resdev-plot

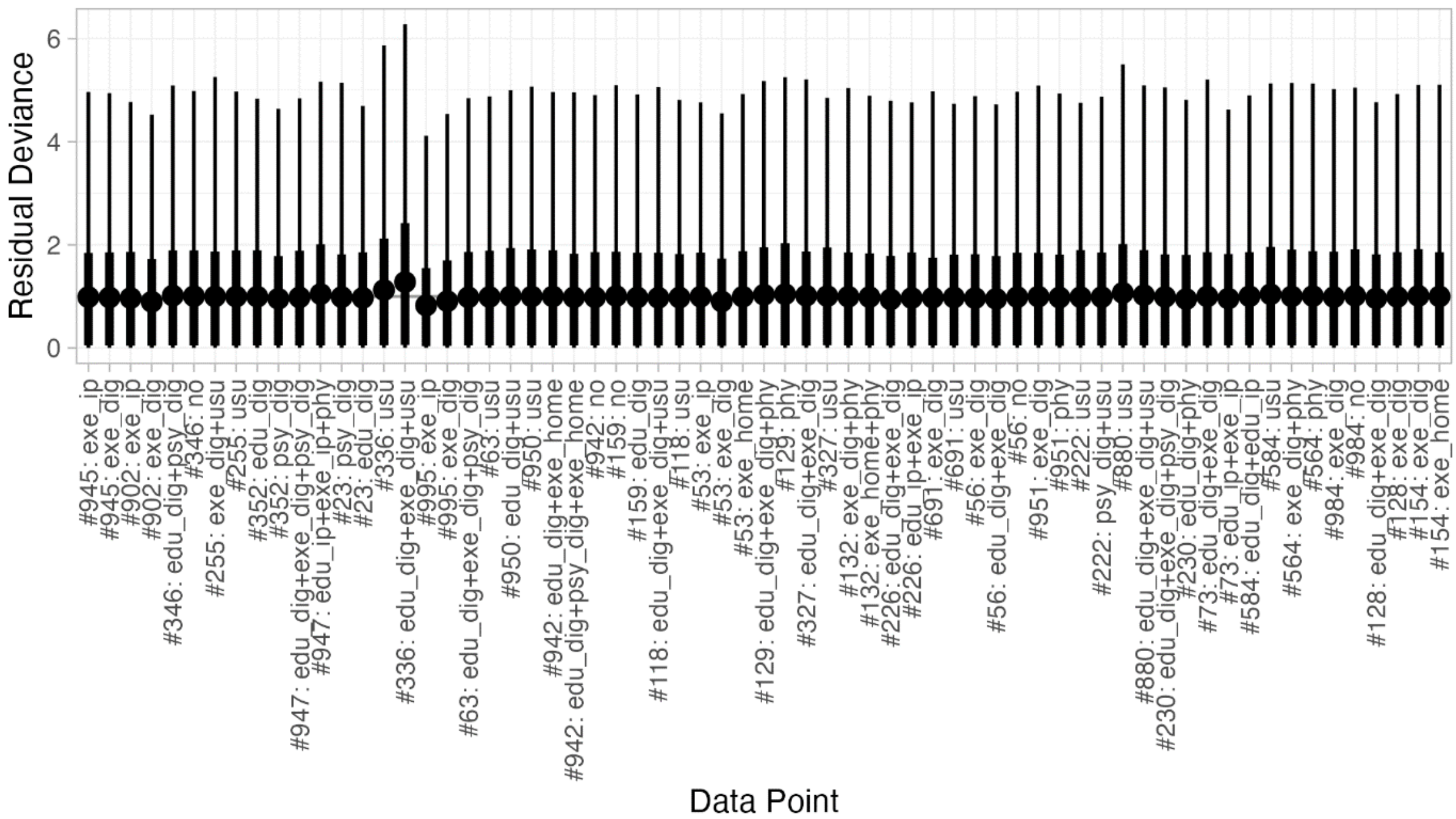

Supplemental Figure 47 – Residual deviance plot for the disability immediate network showing the residual deviance contribution for each datapoint. edu = education, exe = exercise, psy = psychological, phy = physiotherapy, usu = usual care, no = no intervention, dig = digital, ip = in-person, home = home program.

Local inconsistency - nodesplitting

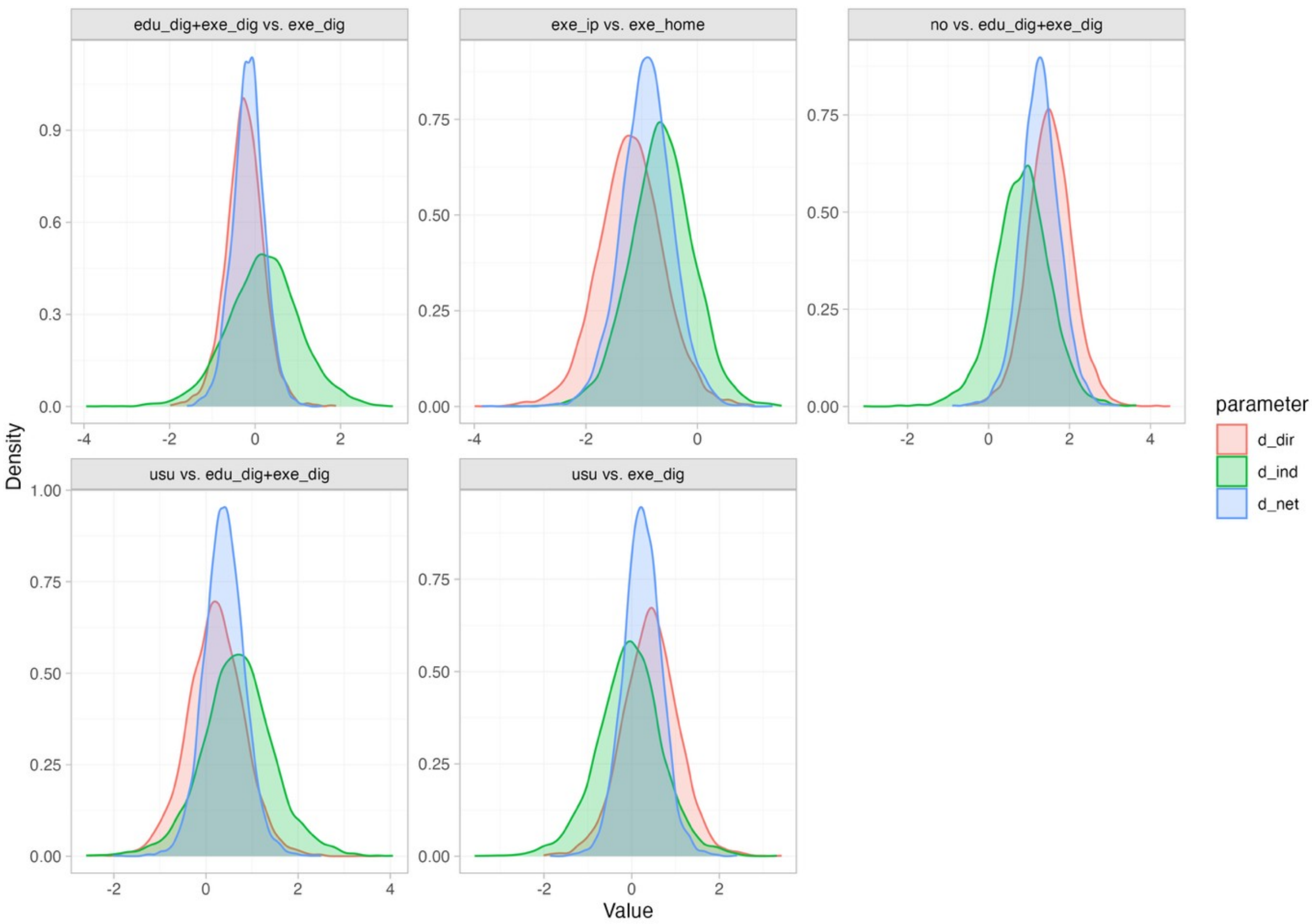

Supplemental Figure 48 – Nodesplitting plots of the disability immediate network. d_dir = direct estimate, d_ind = indirect estimate, d_net = network estimate. edu = education, exe = exercise, psy = psychological, phy = physiotherapy, usu = usual care, no = no intervention, dig = digital, ip = in-person, home = home program.

Supplemental Table 20 – Nodesplitting table displaying results of local inconsistency assessment in the disability immediate network

Comparison	Parameter	SMD	Lower 95%CrI	Upper 95%CrI	Rhat	Bayesian p-value	DIC	Residual Deviance	pD
edu_dig+exe_dig vs. exe_dig	d_net[edu_dig+exe_dig vs. exe_dig]	-0.154	-0.895	0.578	1.000	0.620	127.850	64.293	63.556
	d_dir[edu_dig+exe_dig vs. exe_dig]	-0.247	-1.133	0.625	1.001				
	d_ind[edu_dig+exe_dig vs. exe_dig]	0.207	-1.498	1.922	1.002				
	omega	-0.454	-2.323	1.453	1.002				
	tau	0.564	0.335	0.967	1.002				
	tau_consistency	0.532	0.324	0.884	1.003				
usu vs. exe_dig	d_net[usu vs. exe_dig]	0.243	-0.621	1.121	1.001	0.600	127.761	64.263	63.499
	d_dir[usu vs. exe_dig]	0.430	-0.866	1.679	1.001				
	d_ind[usu vs. exe_dig]	-0.051	-1.579	1.449	1.004				
	omega	0.481	-1.455	2.470	1.004				
	tau	0.567	0.337	0.965	1.002				
	tau_consistency	0.532	0.324	0.884	1.003				
no vs. edu_dig+exe_dig	d_net[no vs. edu_dig+exe_dig]	1.274	0.322	2.232	1.00	0.352	127.74	64.275	63.47

Comparison	Parameter	SMD	Lower 95%CrI	Upper 95%CrI	Rhat	Bayesian p-value	DIC	Residual Deviance	pD
					0		7		2
	d_dir[no vs. edu_dig+exe_dig]	1.519	0.423	2.637	1.00 1				
	d_ind[no vs. edu_dig+exe_dig]	0.829	-0.584	2.185	1.00 2				
	omega	0.690	-0.842	2.270	1.00 1				
	tau	0.546	0.317	0.949	1.00 2				
	tau_consistency	0.532	0.324	0.884	1.00 3				
usu vs. edu_dig+exe_dig	d_net[usu vs. edu_dig+exe_dig]	0.398	-0.451	1.279	1.00 1	0.612	127.82 7	64.268	63.56 0
	d_dir[usu vs. edu_dig+exe_dig]	0.211	-1.035	1.428	1.00 1				
	d_ind[usu vs. edu_dig+exe_dig]	0.688	-0.884	2.312	1.00 3				
	omega	-0.47 7	-2.572	1.554	1.00 2				
	tau	0.575	0.343	0.975	1.00 3				
	tau_consistency	0.532	0.324	0.884	1.00 3				
exe_ip vs. exe_home	d_net[exe_ip vs. exe_home]	-0.90 7	-1.825	0.024	1.00 0	0.409	126.74 5	63.743	63.00 1
	d_dir[exe_ip vs. exe_home]	-1.19	-2.363	-0.011	1.00				

Comparison	Parameter	SMD	Lower 95%CrI	Upper 95%CrI	Rhat	Bayesian p-value	DIC	Residual Deviance	pD
		2			0				
	d_ind[exe_ip vs. exe_home]	-0.63 9	-1.774	0.479	1.00 1				
	omega	-0.55 3	-1.931	0.827	1.00 0				
	tau	0.551	0.324	0.932	1.00 2				
	tau_consistency	0.532	0.324	0.884	1.00 3				

SMD = standardized mean difference, CrI = credible interval, DIC = deviance information criterion, pD = effective number of parameters

Pain intensity immediate

Comparison between UME and standard NMA model

Supplemental Table 21 – Comparison of modelfit parameters between the unrelated mean effects and standard NMA model

Model	Posterior mean of between-study SD	Residual Deviance	DIC	Difference in Residual Deviances	Difference in DICs
NMA	0.144	62.571	115.629	1.589	0.687
UME	0.121	60.982	114.941		

UME = unrelated mean effects, DIC = deviance information criterion

devdev-plot

model 1 = Standard NMA model, model 2 = unrelated mean effects model

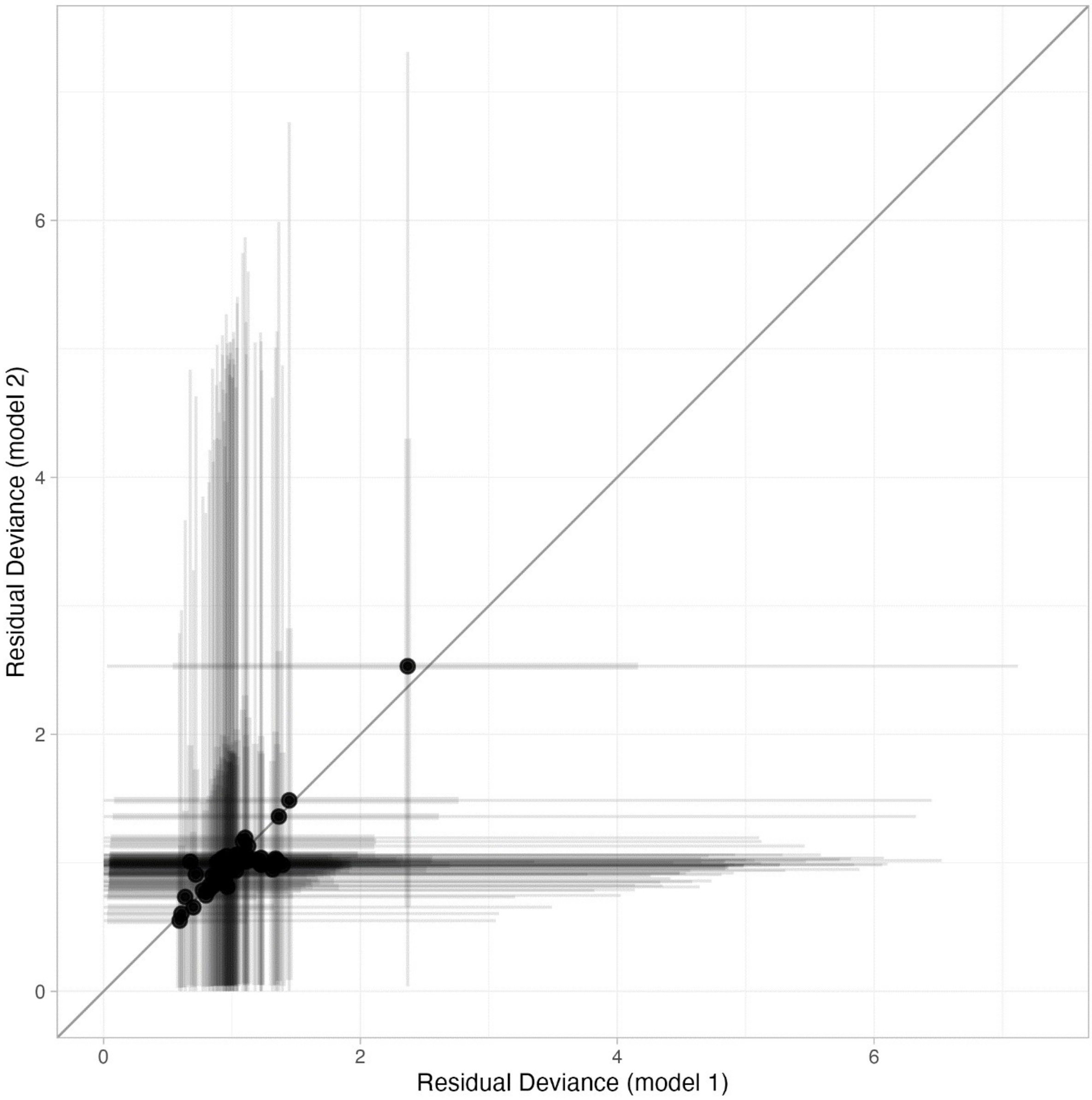

Supplemental Figure 49 – Deviance contribution plot for the pain intensity immediate network. Model 1 represents the single-effect NMA model, model 2 represents the unrelated mean effects model. If the points are close to the line of equity, consistency between the two models is assumed.

resdev plot

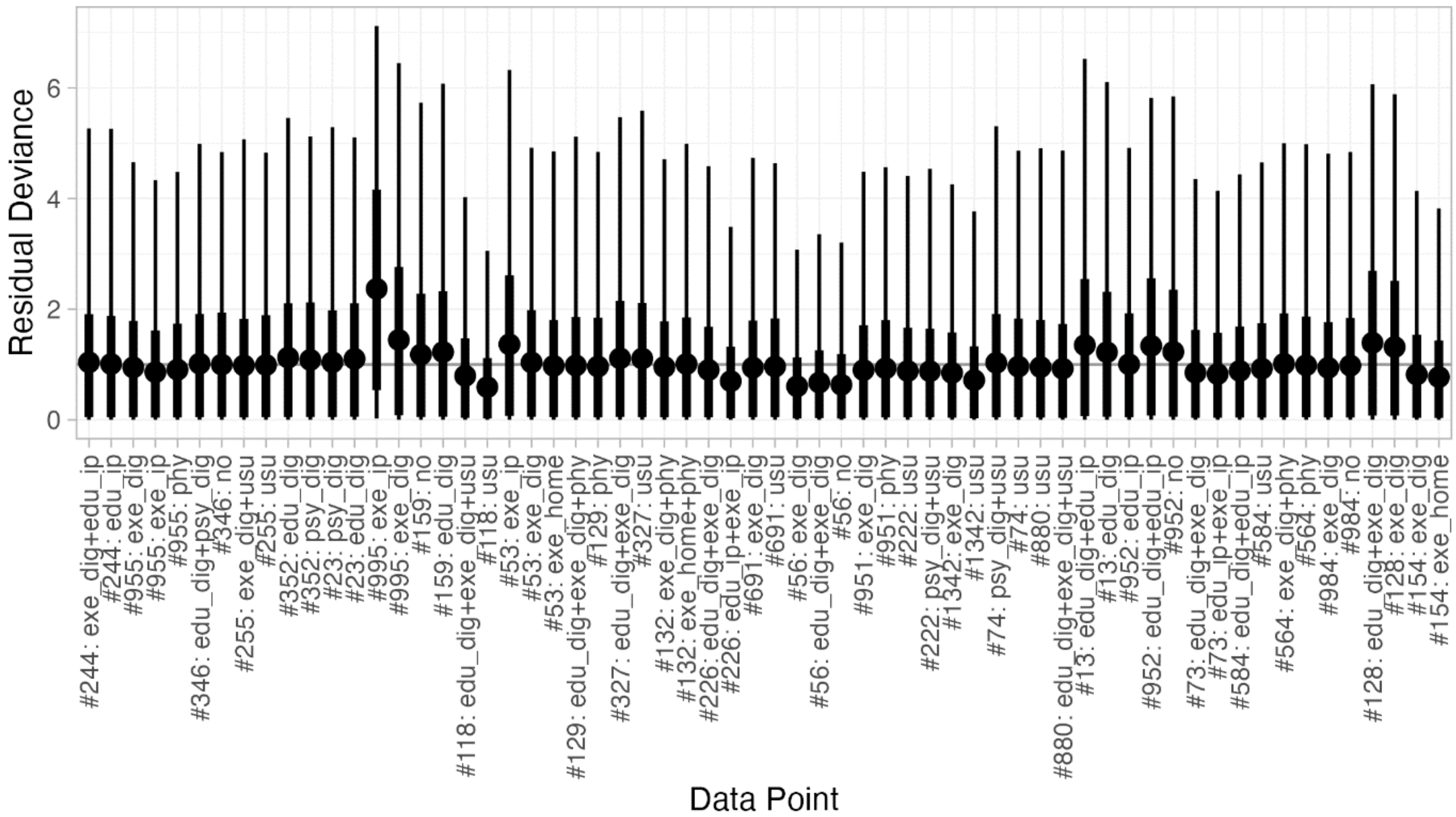

Supplemental Figure 50 – Residual deviance plot for the pain intensity immediate network showing the residual deviance contribution for each datapoint. edu = education, exe = exercise, psy = psychological, phy = physiotherapy, usu = usual care, no = no intervention, dig = digital, ip = in-person, home = home program.

Local inconsistency - nodesplitting

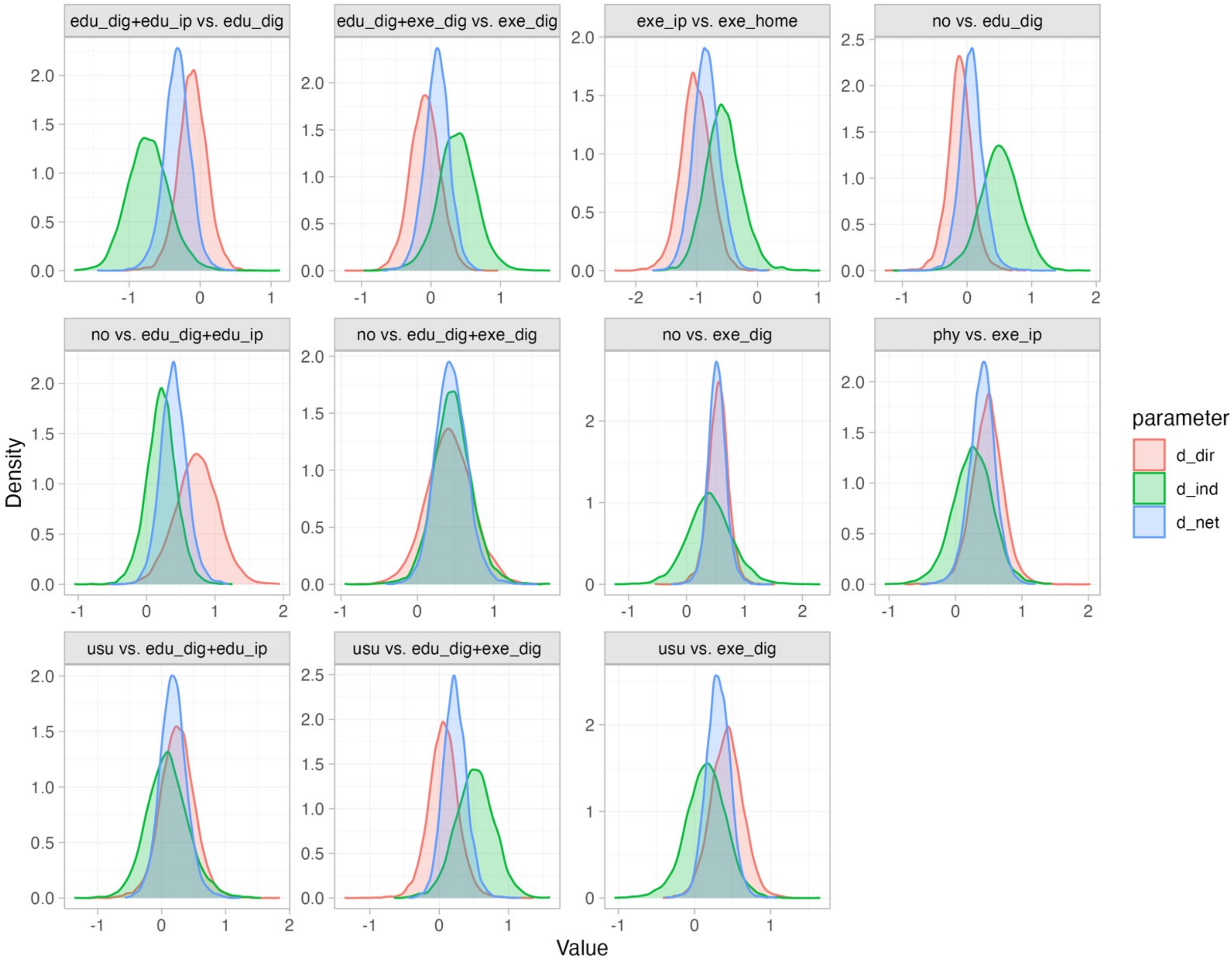

Supplemental Figure 51 – Nodesplitting plots of the pain intensity network. d_dir = direct estimate, d_ind = indirect estimate, d_net = network estimate. edu = education, exe = exercise, psy = psychological, phy = physiotherapy, usu = usual care, no = no intervention, dig = digital, ip = in-person, home = home program.

Supplemental Table 22 - Nodesplitting table displaying results of local inconsistency assessment in the pain intensity immediate network

Comparison	Parameter	SMD	Lower 95%CrI	Upper 95%CrI	Rhat	Bayesian p-value	DIC	Residual Deviance	pD
edu_dig+exe_dig vs. exe_dig	d_net[edu_dig+exe_dig vs. exe_dig]	0.085	-0.277	0.430	1.002	0.172	115.728	62.279	53.450
	d_dir[edu_dig+exe_dig vs. exe_dig]	-0.085	-0.519	0.335	1.001				
	d_ind[edu_dig+exe_dig vs. exe_dig]	0.372	-0.198	0.934	1.001				
	omega	-0.457	-1.150	0.221	1.001				
	tau	0.135	0.008	0.344	1.004				
	tau_consistency	0.144	0.009	0.347	1.004				
no vs. exe_dig	d_net[no vs. exe_dig]	0.526	0.217	0.848	1.001	0.685	116.718	62.928	53.790
	d_dir[no vs. exe_dig]	0.547	0.183	0.893	1.001				
	d_ind[no vs. exe_dig]	0.404	-0.354	1.185	1.003				
	omega	0.143	-0.745	0.973	1.003				
	tau	0.150	0.009	0.359	1.004				
	tau_consistency	0.144	0.009	0.347	1.004				
usu vs. exe_dig	d_net[usu vs. exe_dig]	0.312	0.009	0.622	1.00	0.407	115.48	61.535	53.95

Comparison	Parameter	SMD	Lower 95%CrI	Upper 95%CrI	Rhat	Bayesian p-value	DIC	Residual Deviance	pD
					5		8		3
	d_dir[usu vs. exe_dig]	0.425	0.009	0.848	1.000				
	d_ind[usu vs. exe_dig]	0.151	-0.411	0.673	1.002				
	omega	0.274	-0.396	0.989	1.000				
	tau	0.168	0.017	0.380	1.004				
	tau_consistency	0.144	0.009	0.347	1.004				
edu_dig+edu_ip vs. edu_dig	d_net[edu_dig+edu_ip vs. edu_dig]	-0.324	-0.712	0.046	1.000	0.094	114.013	61.462	52.551
	d_dir[edu_dig+edu_ip vs. edu_dig]	-0.106	-0.524	0.303	1.000				
	d_ind[edu_dig+edu_ip vs. edu_dig]	-0.725	-1.272	-0.120	1.001				
	omega	0.620	-0.121	1.312	1.001				
	tau	0.115	0.005	0.309	1.003				
	tau_consistency	0.144	0.009	0.347	1.004				
no vs. edu_dig	d_net[no vs. edu_dig]	0.072	-0.273	0.450	1.001	0.085	113.707	61.355	52.352
	d_dir[no vs. edu_dig]	-0.10	-0.499	0.284	1.00				

Comparison	Parameter	SMD	Lower 95%CrI	Upper 95%CrI	Rhat	Bayesian p-value	DIC	Residual Deviance	pD
		9			1				
	d_ind[no vs. edu_dig]	0.512	-0.105	1.102	1.00 1				
	omega	-0.62 0	-1.328	0.096	1.00 0				
	tau	0.112	0.005	0.311	1.00 4				
	tau_consistency	0.144	0.009	0.347	1.00 4				
no vs. edu_dig+edu_ip	d_net[no vs. edu_dig+edu_ip]	0.396	0.027	0.794	1.00 1	0.171	114.88 8	61.794	53.09 4
	d_dir[no vs. edu_dig+edu_ip]	0.733	0.119	1.350	1.00 1				
	d_ind[no vs. edu_dig+edu_ip]	0.218	-0.231	0.659	1.00 0				
	omega	0.515	-0.254	1.263	1.00 1				
	tau	0.133	0.009	0.322	1.00 2				
	tau_consistency	0.144	0.009	0.347	1.00 4				
usu vs. edu_dig+edu_ip	d_net[usu vs. edu_dig+edu_ip]	0.182	-0.227	0.617	1.00 1	0.690	116.29 4	62.693	53.60 1
	d_dir[usu vs. edu_dig+edu_ip]	0.246	-0.287	0.773	1.00 0				
	d_ind[usu vs. edu_dig+edu_ip]	0.094	-0.520	0.794	1.00				

Comparison	Parameter	SMD	Lower 95%CrI	Upper 95%CrI	Rhat	Bayesian p-value	DIC	Residual Deviance	pD
					2				
	omega	0.152	-0.697	0.950	1.001				
	tau	0.151	0.009	0.371	1.008				
	tau_consistency	0.144	0.009	0.347	1.004				
no vs. edu_dig+exe_dig	d_net[no vs. edu_dig+exe_dig]	0.441	0.040	0.875	1.000	0.920	117.303	63.066	54.237
	d_dir[no vs. edu_dig+exe_dig]	0.419	-0.183	1.033	1.000				
	d_ind[no vs. edu_dig+exe_dig]	0.460	-0.021	0.987	1.001				
	omega	-0.040	-0.745	0.646	1.001				
	tau	0.155	0.012	0.359	1.008				
	tau_consistency	0.144	0.009	0.347	1.004				
usu vs. edu_dig+exe_dig	d_net[usu vs. edu_dig+exe_dig]	0.227	-0.097	0.577	1.001	0.195	115.160	62.043	53.118
	d_dir[usu vs. edu_dig+exe_dig]	0.060	-0.377	0.499	1.001				
	d_ind[usu vs. edu_dig+exe_dig]	0.511	-0.047	1.050	1.001				
	omega	-0.45	-1.149	0.264	1.00				

Comparison	Parameter	SMD	Lower 95%CrI	Upper 95%CrI	Rhat	Bayesian p-value	DIC	Residual Deviance	pD
		1			0				
	tau	0.134	0.006	0.341	1.00 5				
	tau_consistency	0.144	0.009	0.347	1.00 4				
exe_ip vs. exe_home	d_net[exe_ip vs. exe_home]	-0.84 4	-1.267	-0.409	1.00 2	0.144	115.00 2	61.419	53.58 3
	d_dir[exe_ip vs. exe_home]	-1.01 8	-1.517	-0.543	1.00 0				
	d_ind[exe_ip vs. exe_home]	-0.55 9	-1.105	0.047	1.00 0				
	omega	-0.45 9	-1.120	0.174	1.00 1				
	tau	0.141	0.008	0.337	1.00 2				
	tau_consistency	0.144	0.009	0.347	1.00 4				
phy vs. exe_ip	d_net[phy vs. exe_ip]	0.404	0.009	0.786	1.00 0	0.505	116.17 8	62.165	54.01 3
	d_dir[phy vs. exe_ip]	0.481	0.017	0.952	1.00 0				
	d_ind[phy vs. exe_ip]	0.260	-0.361	0.859	1.00 1				
	omega	0.221	-0.489	0.948	1.00 1				
	tau	0.161	0.014	0.376	1.00				

Comparison	Parameter	SMD	Lower 95%CrI	Upper 95%CrI	Rhat	Bayesian p-value	DIC	Residual Deviance	pD
					6				
	tau_consistency	0.144	0.009	0.347	1.004				

SMD = standardized mean difference, CrI = credible interval, DIC = deviance information criterion, pD = effective number of parameters

Supplemental Data 24 – Results of additional analyses

Disability immediate

Subgroup analysis: chronic pain patients

Component level

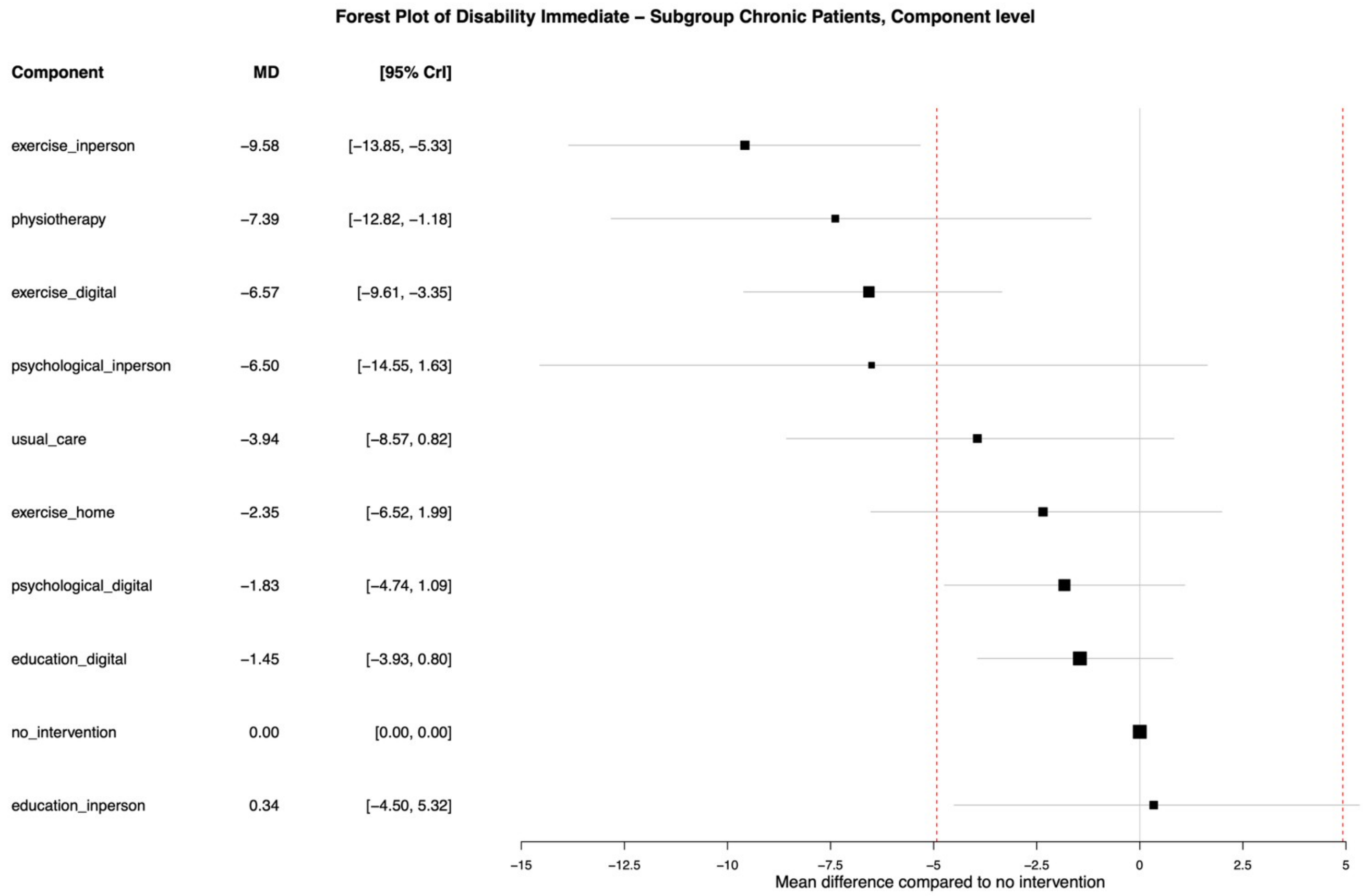

Supplemental Figure 52 – Forest plot of relative effects for the additive disability immediate subgroup chronic pain network on the component level with 95%-credible intervals; red dotted lines represent the MCID of 4.92 for this outcome.

SUCRA table

Supplemental Table 23 - SUCRA table of the additive disability immediate subgroup chronic patients network on the component level

Intervention	Mean Rank	2.5% CI	97.5% CI	SUCRA
exercise_inperson	1.58	1.00	4.00	0.94
physiotherapy	2.87	1.00	7.00	0.81
exercise_digital	3.26	2.00	5.00	0.77
psychological_inperson	3.66	1.00	10.00	0.73
usual_care	5.15	2.00	9.00	0.58
exercise_home	6.55	4.00	10.00	0.44
psychological_digital	6.89	4.00	10.00	0.41
education_digital	7.28	4.00	10.00	0.37
education_inperson	8.75	5.00	10.00	0.23
no_intervention	9.02	7.00	10.00	0.20

Intervention level

Forest Plot of Disability Immediate – Subgroup Chronic Patients

Intervention	MD	[95% CrI]
exercise_digital+exercise_inperson+psychological_inperson	−22.65	[−32.98, −12.37]
education_inperson+exercise_inperson+physiotherapy	−16.63	[−24.50, −7.63]
exercise_digital+exercise_inperson	−16.15	[−22.60, −9.62]
exercise_digital+physiotherapy	−13.96	[−21.13, −5.86]
education_digital+exercise_digital+usual_care	−11.96	[−19.13, −4.55]
exercise_digital+usual_care	−10.51	[−16.92, −3.47]
education_digital+exercise_digital+psychological_digital	−9.85	[−14.49, −5.44]
exercise_home+physiotherapy	−9.73	[−16.99, −0.99]
exercise_inperson	−9.58	[−13.85, −5.33]
education_inperson+exercise_inperson	−9.24	[−14.40, −3.96]
education_digital+exercise_digital	−8.02	[−11.46, −4.65]
physiotherapy	−7.39	[−12.82, −1.18]
exercise_digital	−6.57	[−9.61, −3.35]
exercise_digital+education_inperson	−6.23	[−12.17, 0.09]
psychological_digital+usual_care	−5.77	[−11.35, 0.06]
education_digital+psychological_digital+exercise_home	−5.63	[−10.56, −0.59]
usual_care	−3.94	[−8.57, 0.82]
education_digital+exercise_home	−3.80	[−8.00, 0.32]
education_digital+psychological_digital	−3.28	[−7.27, 0.63]
exercise_home	−2.35	[−6.52, 1.99]
psychological_digital	−1.83	[−4.74, 1.09]
education_digital	−1.45	[−3.93, 0.80]
education_digital+education_inperson	−1.12	[−7.16, 4.75]
no_intervention	0.00	[0.00, 0.00]
education_inperson	0.34	[−4.50, 5.32]

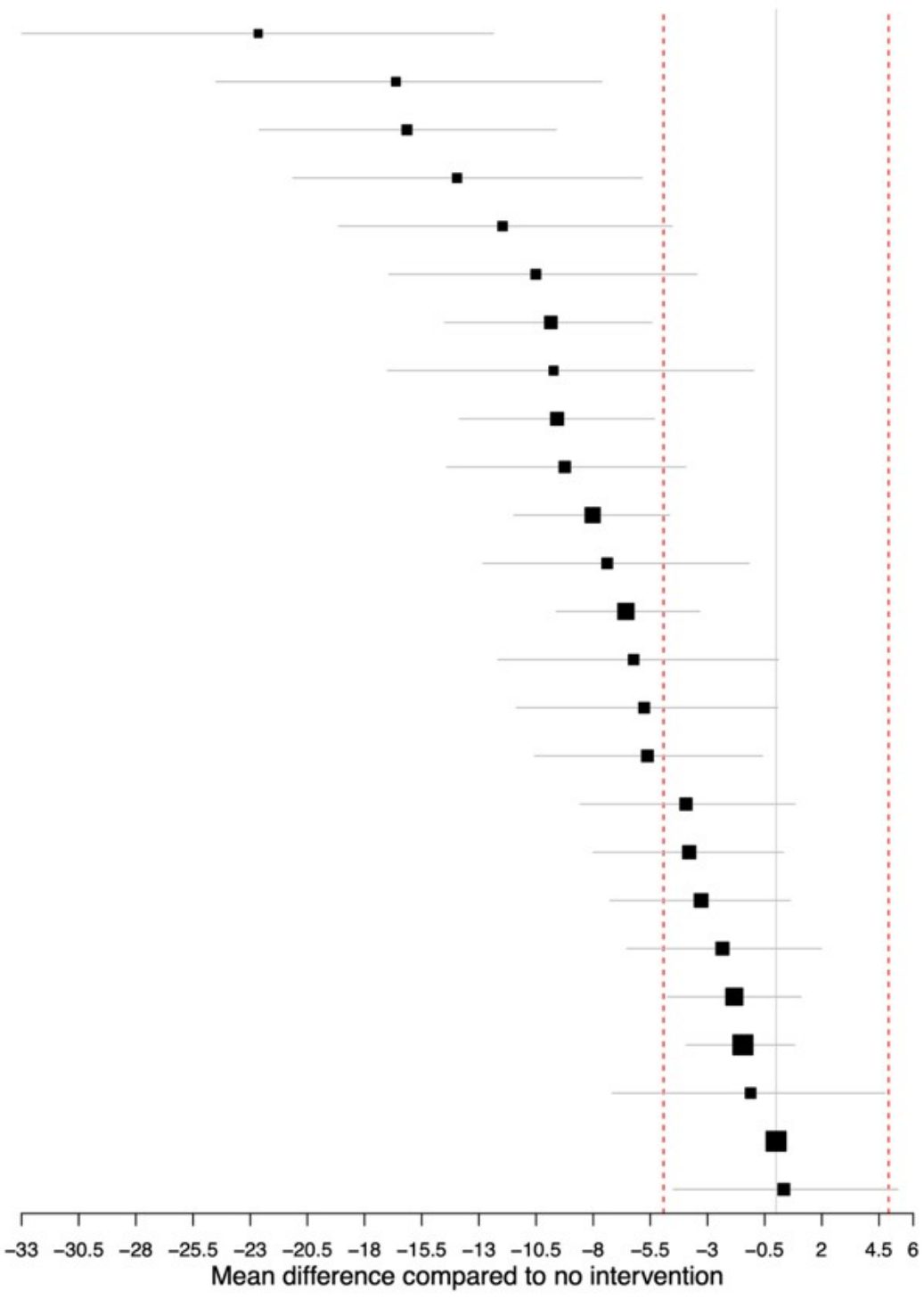

Supplemental Figure 53 – Forest plot of relative effects for the additive disability immediate subgroup chronic pain network on the intervention level with 95%-credible intervals; red dotted lines represent the MCID of 4.92 for this outcome.

SUCRA table

Supplemental Table 24 - SUCRA table of the additive disability immediate subgroup chronic patients network on the intervention level

Intervention	Mean Rank	2.5% CI	97.5% CI	SUCRA
exercise_digital+exercise_inperson+psychological_inperson	1.29	1.00	4.00	0.99
education_inperson+exercise_inperson+physiotherapy	2.71	1.00	6.00	0.93
exercise_digital+exercise_inperson	3.00	2.00	6.00	0.92
exercise_digital+physiotherapy	4.61	2.00	11.00	0.86
education_digital+exercise_digital+usual_care	5.78	2.00	12.00	0.81
exercise_digital+usual_care	7.77	4.00	14.00	0.73
education_digital+exercise_digital+psychological_digital	8.06	4.00	13.00	0.72
exercise_inperson	8.72	4.00	15.00	0.69
exercise_home+physiotherapy	9.05	4.00	21.00	0.68
education_inperson+exercise_inperson	9.13	4.00	15.00	0.67
education_digital+exercise_digital	11.02	7.00	14.00	0.60
physiotherapy	12.35	6.00	22.00	0.55
exercise_digital	13.63	9.00	18.00	0.49
exercise_digital+education_inperson	14.07	7.00	22.00	0.48
psychological_digital+usual_care	14.55	8.00	22.00	0.46
education_digital+psychological_digital+exercise_home	14.70	9.00	20.00	0.45
usual_care	17.80	12.00	23.00	0.33
education_digital+exercise_home	17.80	13.00	23.00	0.33
education_digital+psychological_digital	18.22	11.00	23.00	0.31
exercise_home	20.27	15.00	25.00	0.23
psychological_digital	20.85	16.00	25.00	0.21
education_digital+education_inperson	21.22	13.00	25.00	0.19
education_digital	21.37	16.00	24.00	0.19
education_inperson	23.45	17.00	25.00	0.10
no_intervention	23.55	20.00	25.00	0.10

Subgroup analysis: Fully digital interventions

Component level

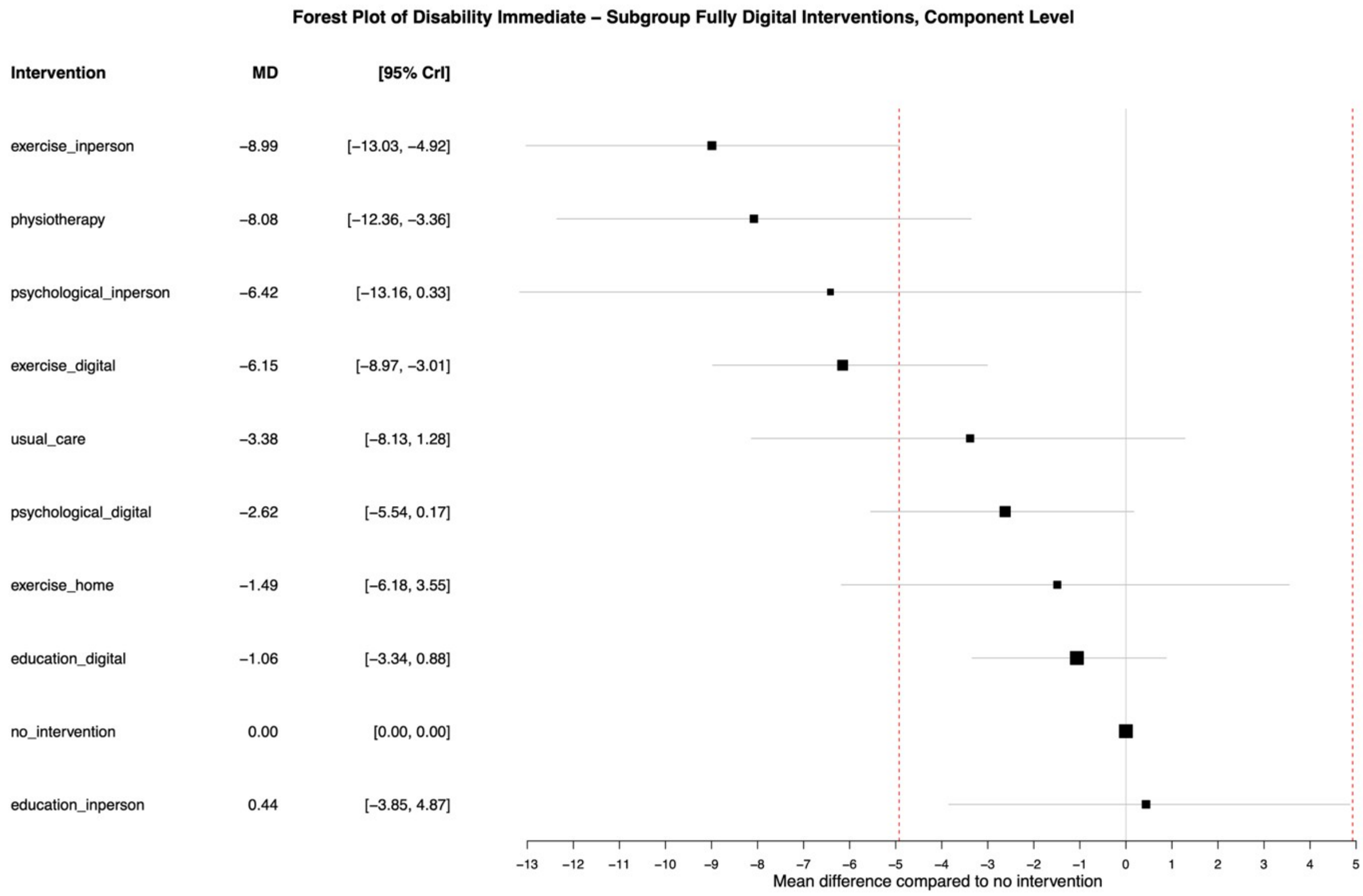

Supplemental Figure 54– Forest plot of relative effects for the additive disability immediate subgroup fully digital interventions network on the component level with 95%-credible intervals; red dotted lines represent the MCID of 4.92 for this outcome.

SUCRA table

Supplemental Table 25 -- SUCRA table of the additive disability immediate subgroup fully digital interventions network on the component level

Intervention	Mean Rank	2.5% CI	97.5% CI	SUCRA
exercise_inperson	1.70	1.00	4.00	0.93
physiotherapy	2.19	1.00	5.00	0.88
exercise_digital	3.50	2.00	5.00	0.75
psychological_inperson	3.50	1.00	9.00	0.75
usual_care	5.51	3.00	9.00	0.55
psychological_digital	6.02	4.00	9.00	0.50
exercise_home	7.21	4.00	10.00	0.38
education_digital	7.62	5.00	10.00	0.34
education_inperson	8.86	5.00	10.00	0.21
no_intervention	8.89	7.00	10.00	0.21

Intervention level

Forest Plot of Disability Immediate – Subgroup Fully Digital Interventions

Intervention	MD	[95% CrI]
exercise_digital+exercise_inperson+psychological_inperson	−21.56	[−30.81, −12.41]
education_inperson+exercise_inperson+physiotherapy	−16.63	[−24.44, −8.63]
exercise_digital+exercise_inperson	−15.14	[−21.33, −8.84]
education_digital+exercise_digital+psychological_digital	−9.84	[−15.18, −4.86]
education_digital+physiotherapy	−9.14	[−14.23, −4.11]
exercise_inperson	−8.99	[−13.03, −4.92]
education_inperson+exercise_inperson	−8.55	[−13.84, −3.32]
physiotherapy	−8.08	[−12.36, −3.36]
education_digital+exercise_digital	−7.22	[−11.15, −3.55]
exercise_digital	−6.15	[−8.97, −3.01]
exercise_digital+education_inperson	−5.72	[−11.60, 0.26]
education_digital+psychological_digital	−3.69	[−7.79, −0.05]
usual_care	−3.38	[−8.13, 1.28]
psychological_digital	−2.62	[−5.54, 0.17]
exercise_home	−1.49	[−6.18, 3.55]
education_digital	−1.06	[−3.34, 0.88]
education_digital+education_inperson	−0.63	[−5.98, 4.67]
no_intervention	0.00	[0.00, 0.00]
education_inperson	0.44	[−3.85, 4.87]

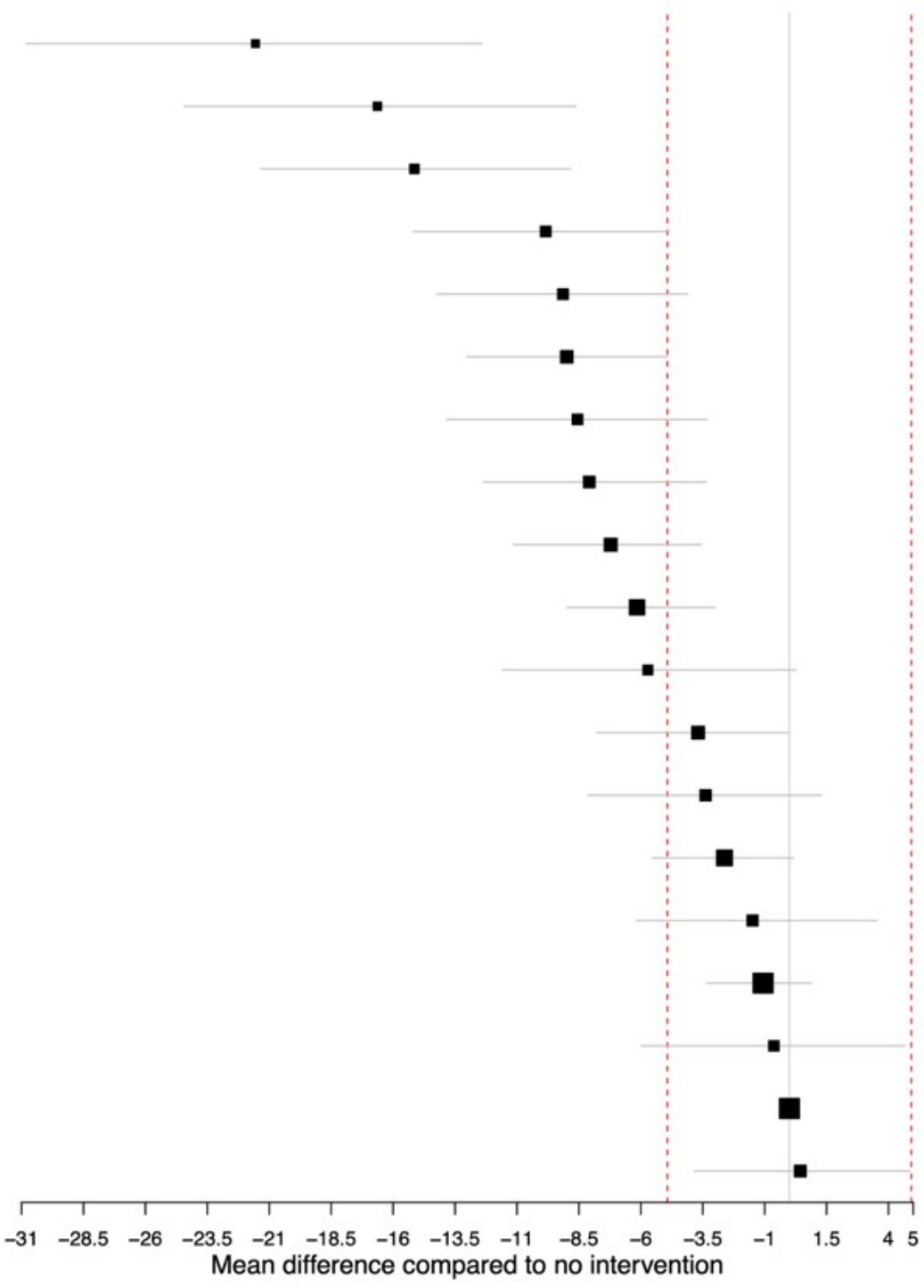

Supplemental Figure 55 – Forest plot of relative effects for the additive disability immediate subgroup fully digital interventions network on the intervention level with 95%-credible intervals; red dotted lines represent the MCID of 4.92 for this outcome.

SUCRA table

Supplemental Table 26 - SUCRA table of the additive disability immediate subgroup fully digital interventions network on the intervention level

Intervention	Mean Rank	2.5% CI	97.5% CI	SUCRA
exercise_digital+exercise_inperson+psychological_inperson	1.19	1.00	3.00	0.99
education_inperson+exercise_inperson+physiotherapy	2.20	1.00	3.00	0.94
exercise_digital+exercise_inperson	2.77	2.00	4.00	0.91
education_digital+exercise_digital+psychological_digital	5.25	3.00	9.00	0.78
education_digital+physiotherapy	6.00	4.00	11.00	0.74
exercise_inperson	6.36	4.00	11.00	0.72
education_inperson+exercise_inperson	6.77	4.00	11.00	0.70
physiotherapy	7.61	4.00	13.00	0.65
education_digital+exercise_digital	8.66	6.00	11.00	0.60
exercise_digital	10.22	7.00	13.00	0.51
exercise_digital+education_inperson	10.50	5.00	16.00	0.50
education_digital+psychological_digital	12.47	8.00	16.00	0.40
usual_care	13.19	9.98	18.00	0.36
psychological_digital	14.06	10.00	18.00	0.31
exercise_home	15.37	11.00	19.00	0.24
education_digital	16.00	13.00	18.00	0.21
education_digital+education_inperson	16.20	12.00	19.00	0.20
no_intervention	17.47	14.00	19.00	0.13
education_inperson	17.72	14.00	19.00	0.12

Sensitivity analysis: Excluding studies with less than 10 participants in one arm

Component level

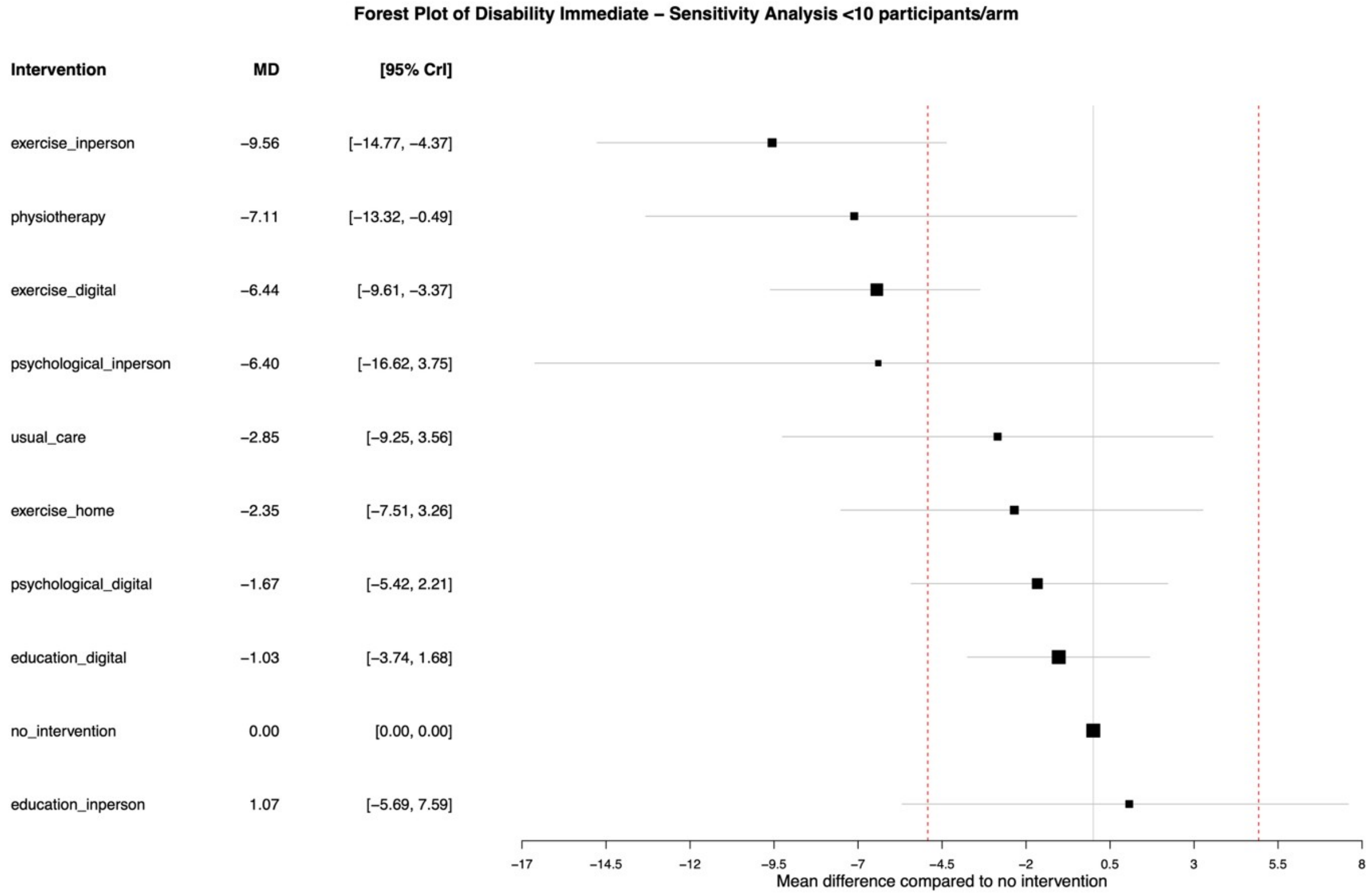

Supplemental Figure 56 – Forest plot of relative effects for the additive disability immediate sensitivity analysis <10 participants in one arm network on the component level with 95%-credible intervals; red dotted lines represent the MCID of 4.92 for this outcome.

SUCRA table

Supplemental Table 27 - SUCRA table of the additive disability immediate sensitivity analysis <10 participants in one arm network on the component level

Intervention	Mean Rank	2.5% CI	97.5% CI	SUCRA
exercise_inperson	1.76	1.00	5.00	0.92
physiotherapy	3.02	1.00	7.00	0.80
exercise_digital	3.23	2.00	5.00	0.78
psychological_inperson	3.84	1.00	10.00	0.72
usual_care	5.80	2.00	10.00	0.52
exercise_home	6.31	3.00	10.00	0.47
psychological_digital	6.73	4.00	10.00	0.43
education_digital	7.24	4.00	10.00	0.38
no_intervention	8.47	6.00	10.00	0.25
education_inperson	8.61	4.00	10.00	0.24

Intervention level

Forest Plot of Disability Immediate – Sensitivity Analysis <10 participants/arm

Intervention	MD	[95% CrI]
exercise_digital+exercise_inperson+psychological_inperson	−22.40	[−34.97, −9.67]
exercise_digital+exercise_inperson	−16.00	[−23.15, −8.74]
education_inperson+exercise_inperson+physiotherapy	−15.60	[−24.43, −6.36]
education_digital+exercise_digital+physiotherapy	−14.58	[−21.96, −6.39]
exercise_digital+physiotherapy	−13.56	[−21.19, −5.24]
education_digital+exercise_digital+usual_care	−10.32	[−18.53, −2.19]
exercise_inperson	−9.56	[−14.77, −4.37]
exercise_home+physiotherapy	−9.46	[−18.13, −0.20]
exercise_digital+usual_care	−9.29	[−17.05, −1.39]
education_digital+exercise_digital+psychological_digital	−9.13	[−13.91, −4.00]
education_inperson+exercise_inperson	−8.49	[−14.86, −2.28]
education_digital+physiotherapy	−8.14	[−14.58, −1.10]
education_digital+exercise_digital	−7.47	[−10.65, −4.22]
physiotherapy	−7.11	[−13.32, −0.49]
exercise_digital	−6.44	[−9.61, −3.37]
exercise_digital+education_inperson	−5.37	[−12.94, 2.10]
education_digital+psychological_digital+exercise_home	−5.04	[−11.04, 1.10]
psychological_digital+usual_care	−4.51	[−12.03, 3.15]
education_digital+usual_care	−3.88	[−11.34, 3.41]
education_digital+exercise_home	−3.38	[−8.35, 2.03]
usual_care	−2.85	[−9.25, 3.56]
education_digital+psychological_digital	−2.69	[−7.53, 2.00]
exercise_home	−2.35	[−7.51, 3.26]
psychological_digital	−1.67	[−5.42, 2.21]
education_digital	−1.03	[−3.74, 1.68]
no_intervention	0.00	[0.00, 0.00]
education_digital+education_inperson	0.04	[−7.49, 7.81]
education_inperson	1.07	[−5.69, 7.59]

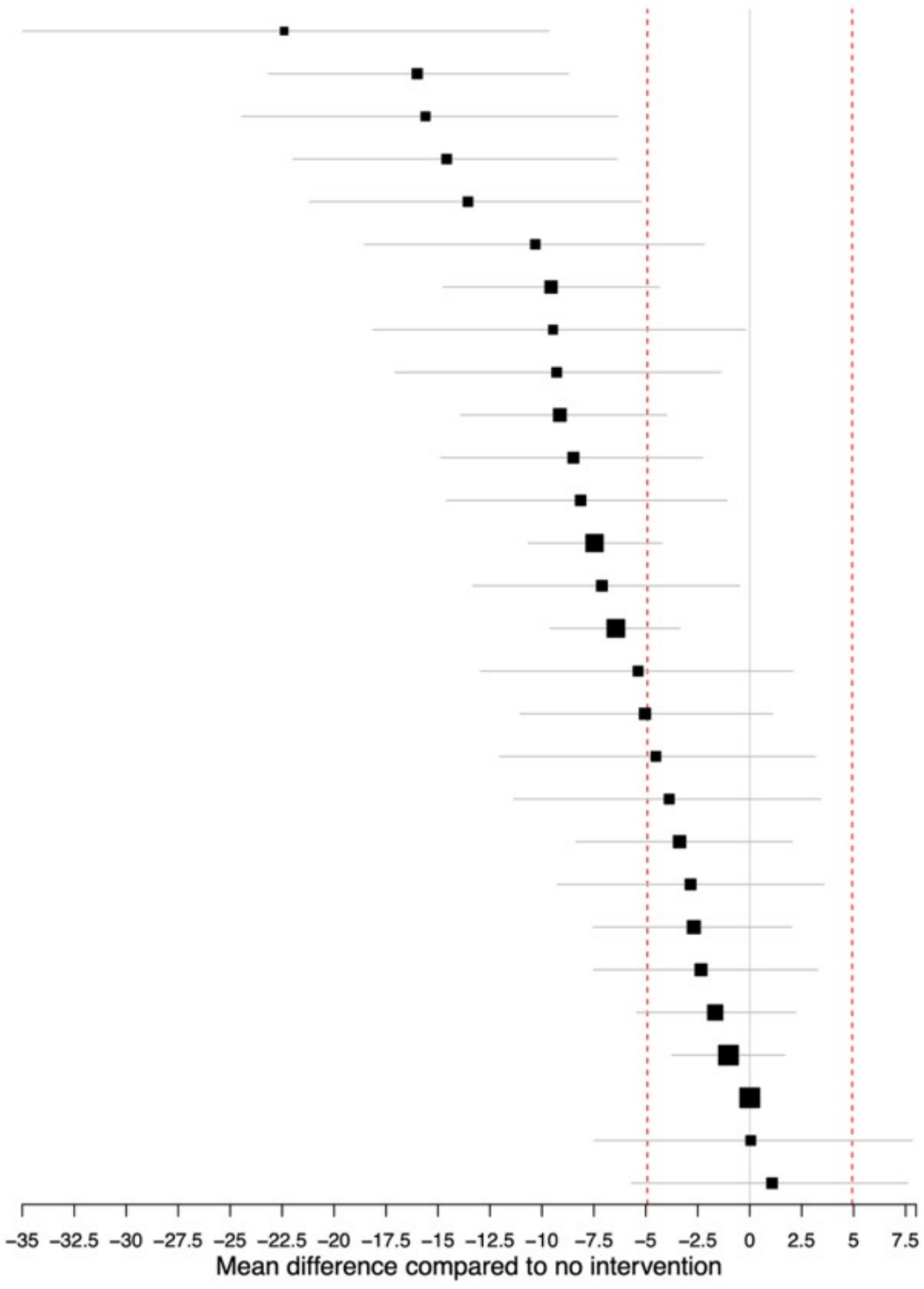

Supplemental Figure 57 - Forest plot of relative effects for the additive disability immediate sensitivity analysis <10 participants in one arm network on the intervention level with 95%-credible intervals; red dotted lines represent the MCID of 4.92 for this outcome.

SUCRA table

Supplemental Table 28 - SUCRA table of the additive disability immediate sensitivity analysis <10 participants in one arm network on the intervention level

Intervention	Mean Rank	2.5% CI	97.5% CI	SUCRA
exercise_digital+exercise_inperson+psychological_inperson	1.70	1.00	7.00	0.98
exercise_digital+exercise_inperson	3.43	1.00	8.00	0.91
education_inperson+exercise_inperson+physiotherapy	3.53	1.00	9.00	0.91
education_digital+exercise_digital+physiotherapy	4.07	1.00	9.00	0.89
exercise_digital+physiotherapy	5.37	2.00	13.00	0.84
education_digital+exercise_digital+usual_care	8.39	2.00	18.00	0.74
exercise_inperson	9.67	3.00	19.00	0.69
education_digital+exercise_digital+psychological_digital	9.79	4.00	16.00	0.69
exercise_digital+usual_care	10.09	4.00	20.00	0.68
exercise_home+physiotherapy	10.44	4.00	24.00	0.66
education_inperson+exercise_inperson	11.08	4.00	20.00	0.64
education_digital+physiotherapy	11.87	5.00	21.00	0.61
education_digital+exercise_digital	12.62	8.00	18.00	0.58
physiotherapy	13.98	7.00	24.00	0.54
exercise_digital	14.79	9.00	21.00	0.51
exercise_digital+education_inperson	16.43	6.00	26.00	0.45
education_digital+psychological_digital+exercise_home	16.83	8.00	25.00	0.43
psychological_digital+usual_care	17.76	8.00	26.03	0.40
education_digital+usual_care	18.74	8.00	27.00	0.37
education_digital+exercise_home	19.91	12.00	27.00	0.32
education_digital+psychological_digital	20.75	12.00	27.00	0.29
usual_care	21.00	12.00	28.00	0.29
exercise_home	21.81	13.00	28.00	0.26
psychological_digital	22.80	16.00	28.00	0.22
education_digital	23.68	17.00	28.00	0.19
education_digital+education_inperson	24.22	14.00	28.00	0.17
no_intervention	25.41	20.00	28.00	0.13
education_inperson	25.84	17.00	28.00	0.11

Network meta-regression sample size

Supplemental Table 29 – Results of the sample size network meta-regression, incl. modelfit parameters

Model	Number of effective parameters	Posterior mean of between-study SD	Residual Deviance	DIC	B	95% CrI of B
Meta-Regression Sample Size	63.05	0.42	68.22	252.45	0.00	-0.01;0.03

DIC = deviance information criterion, CrI = credible interval

Sensitivity analysis: No intervention and waiting list as separate nodes

Intervention level

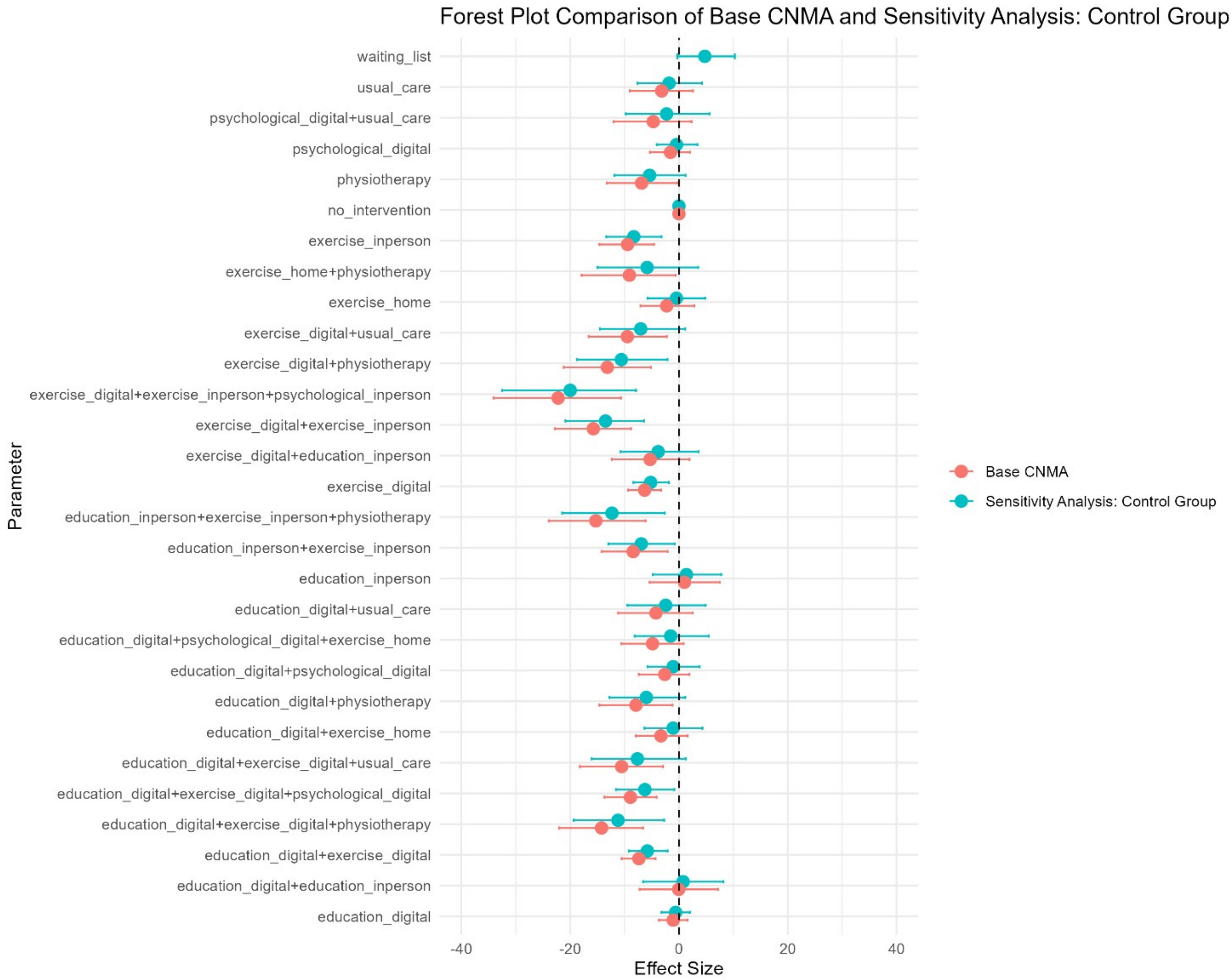

Supplemental Figure 58 - Forest plot of relative effects for the additive disability immediate sensitivity analysis "no intervention and waiting list as separate nodes" network on the intervention level with 95%-credible intervals compared to the base CNMA model

SUCRA table

Supplemental Table 30 - SUCRA table of the additive disability immediate sensitivity analysis analysis "no intervention and waiting list as separate nodes" network on the intervention level

Intervention	Mean Rank	2.5% CI	97.5% CI	SUCRA
exercise_digital+exercise_inperson+psychological_inperson	1.58	1.00	7.00	0.98
exercise_digital+exercise_inperson	3.12	1.00	8.00	0.93
education_inperson+exercise_inperson+physiotherapy	3.90	1.00	11.00	0.90
education_digital+exercise_digital+physiotherapy	4.71	2.00	12.00	0.87
exercise_digital+physiotherapy	5.72	2.00	15.00	0.84
exercise_inperson	8.22	3.00	18.00	0.75
education_digital+exercise_digital+usual_care	8.95	2.00	20.00	0.73
education_inperson+exercise_inperson	10.13	4.00	19.00	0.69
exercise_digital+usual_care	10.15	4.00	20.00	0.68
education_digital+exercise_digital+psychological_digital	11.02	5.00	18.00	0.65
education_digital+exercise_digital	11.90	6.00	17.00	0.62
education_digital+physiotherapy	12.07	5.00	23.00	0.62
exercise_home+physiotherapy	12.87	5.00	27.00	0.59
exercise_digital	13.39	7.00	20.00	0.57
physiotherapy	13.52	6.00	25.00	0.57
exercise_digital+education_inperson	15.94	5.00	26.00	0.48
education_digital+usual_care	18.51	8.00	28.00	0.40
psychological_digital+usual_care	18.98	9.00	28.00	0.38
usual_care	20.11	12.00	27.00	0.34
education_digital+psychological_digital+exercise_home	20.32	11.00	28.00	0.33
education_digital+psychological_digital	21.08	12.00	28.00	0.31
education_digital+exercise_home	21.23	13.00	28.00	0.30
education_digital	22.06	14.00	28.00	0.27
exercise_home	22.48	13.00	28.00	0.26
psychological_digital	22.55	14.00	28.00	0.26
no_intervention	23.42	15.00	28.00	0.23
education_digital+education_inperson	23.74	12.00	29.00	0.22
education_inperson	25.00	15.00	29.00	0.17
waiting_list	28.33	24.00	29.00	0.06

Sensitivity Analysis: Correlation coefficient for imputing missing SDs – 0.40

Intervention level

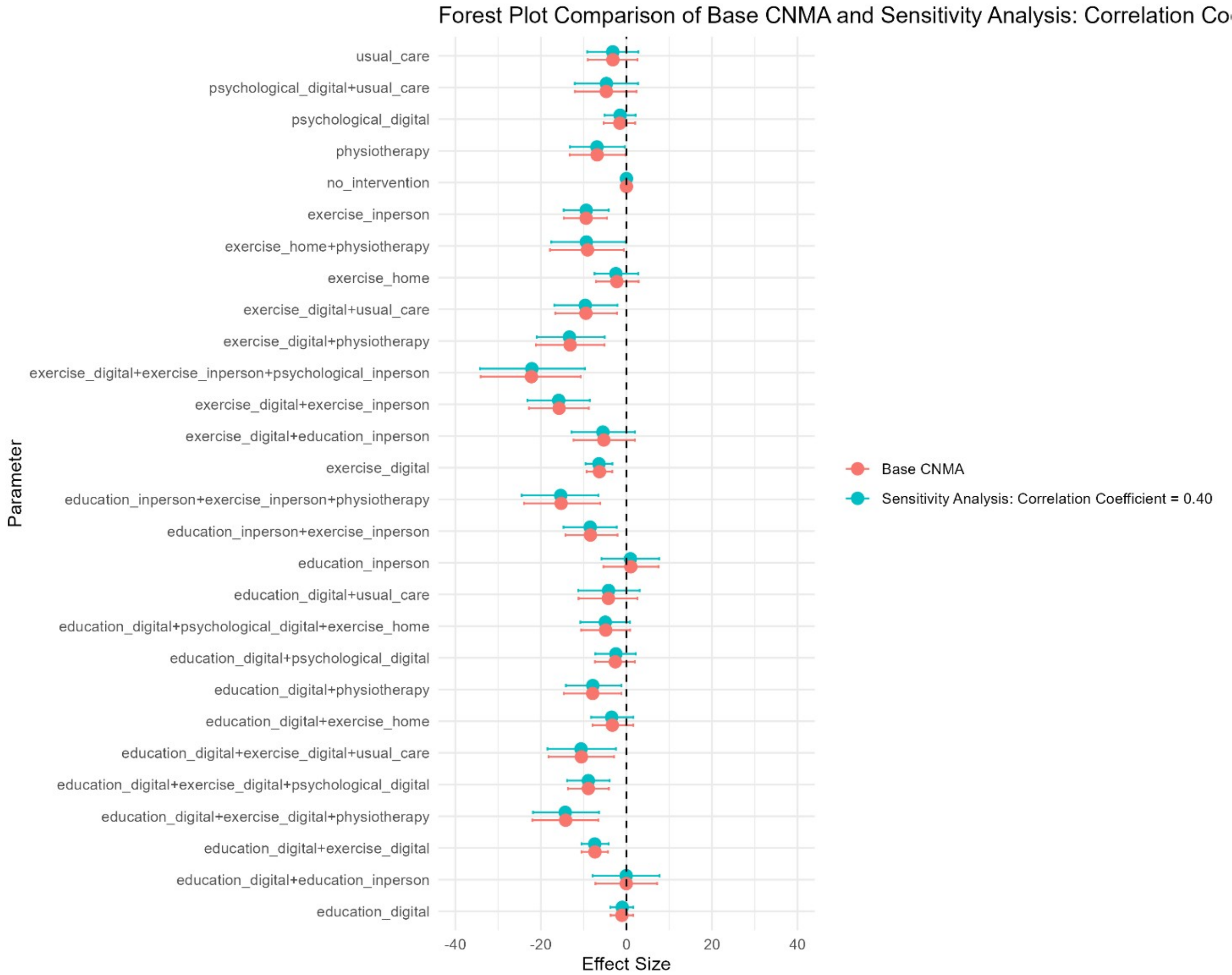

Supplemental Figure 59 - Forest plot of relative effects for the additive disability immediate sensitivity analysis "correlation coefficient for imputing missing SDs – 0.40" network on the intervention level with 95%-credible intervals compared to the base CNMA model

Sensitivity Analysis: Correlation coefficient for imputing missing SDs – 0.81

Intervention level

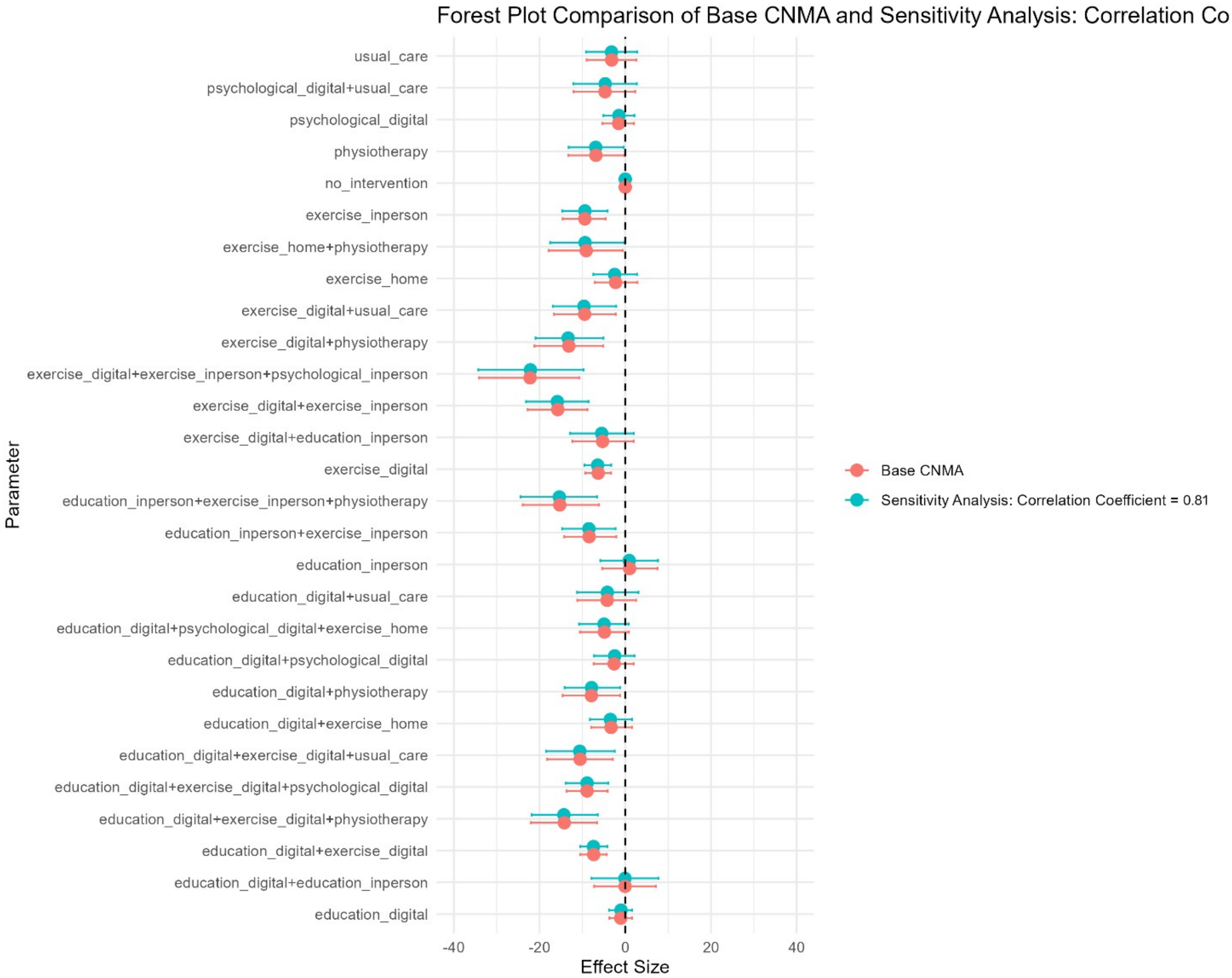

Supplemental Figure 60 - Forest plot of relative effects for the additive disability immediate sensitivity analysis "correlation coefficient for imputing missing SDs – 0.81" network on the intervention level with 95%-credible intervals compared to the base CNMA model

Sensitivity Analysis: Informative prior for between-trial standard deviation

Intervention level

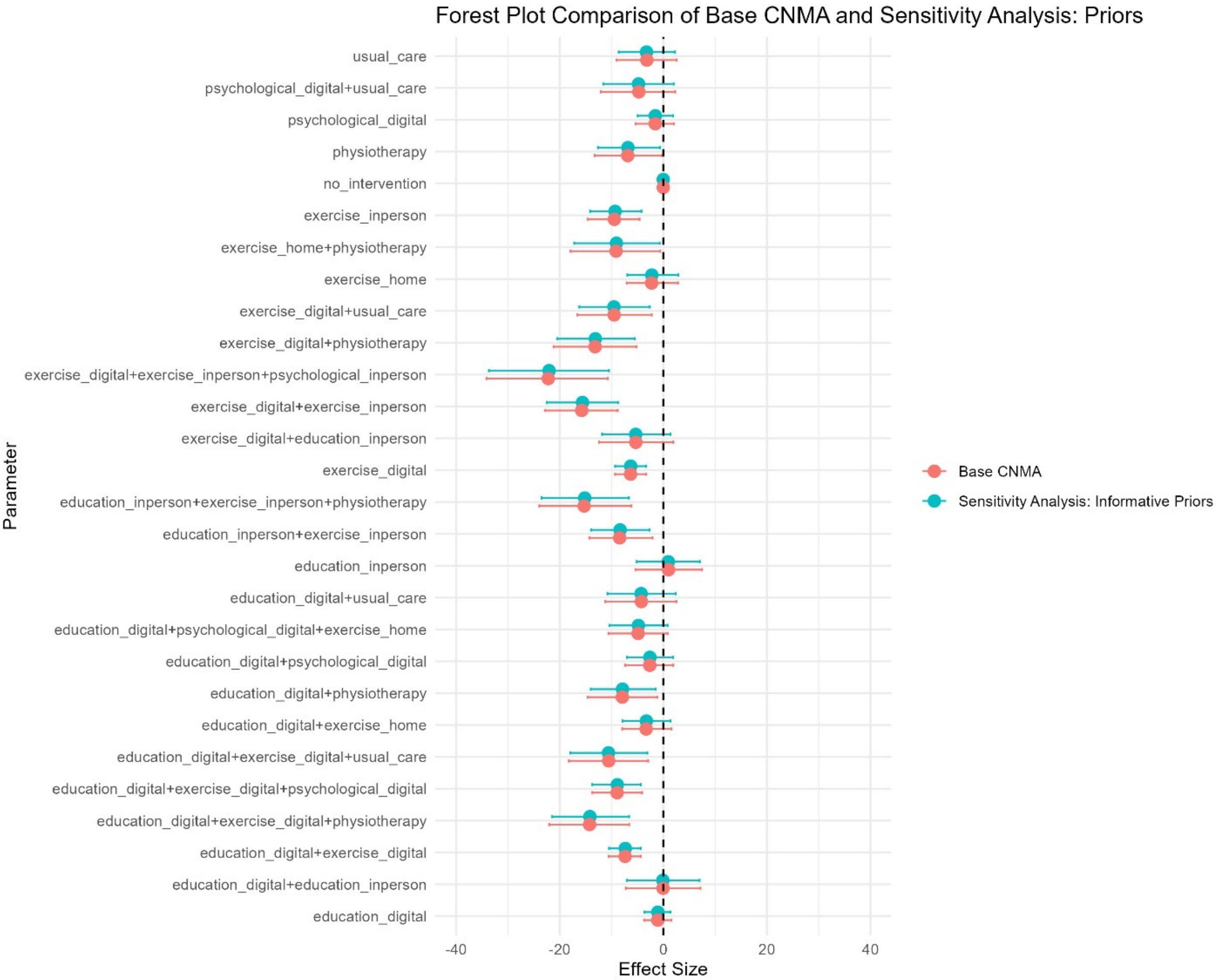

Supplemental Figure 61 - Forest plot of relative effects for the additive disability immediate sensitivity analysis "informative prior for tau" network on the intervention level with 95%-credible intervals compared to the base CNMA model

Pain intensity immediate

Subgroup analysis: chronic pain patients

Component level

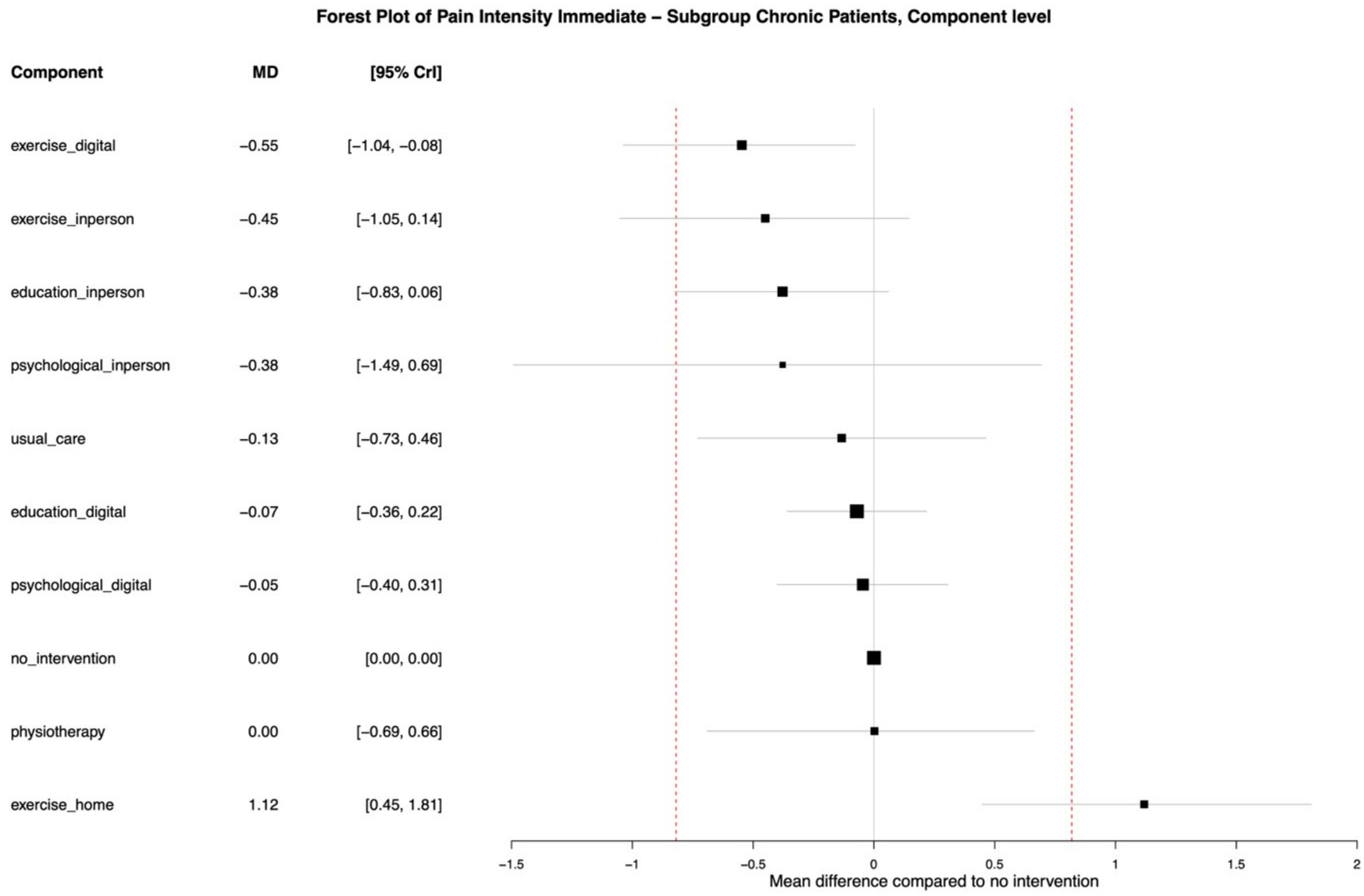

Supplemental Figure 62 – Forest plot of relative effects for the additive pain intensity immediate subgroup chronic pain network on the component level with 95%-credible intervals; red dotted lines represent the MCID of 0.82 for this outcome.

SUCRA table

Supplemental Table 31 - SUCRA table of the additive pain intensity immediate subgroup chronic patients network on the component level

Intervention	Mean Rank	2.5% CI	97.5% CI	SUCRA
exercise_digital	2.16	1.00	5.00	0.88
exercise_inperson	3.10	1.00	8.00	0.79
education_inperson	3.45	1.00	8.00	0.76
psychological_inperson	4.12	1.00	9.00	0.69
usual_care	5.75	2.00	9.00	0.53
education_digital	6.17	3.00	9.00	0.48
psychological_digital	6.42	3.00	9.00	0.46
physiotherapy	6.74	2.00	9.00	0.43
no_intervention	7.10	4.00	9.00	0.39
exercise_home	9.98	10.00	10.00	0.10

Intervention level

Forest Plot of Pain Intensity Immediate – Subgroup Chronic Patients

Intervention	MD	[95% CrI]
exercise_digital+exercise_inperson+psychological_inperson	−1.37	[−2.86, 0.09]
exercise_digital+exercise_inperson	−1.00	[−2.03, −0.02]
exercise_digital+education_inperson	−0.93	[−1.64, −0.24]
education_inperson+exercise_inperson	−0.83	[−1.54, −0.13]
education_inperson+exercise_inperson+physiotherapy	−0.83	[−2.02, 0.33]
education_digital+exercise_digital+usual_care	−0.75	[−1.79, 0.24]
exercise_digital+usual_care	−0.68	[−1.68, 0.27]
education_digital+exercise_digital+psychological_digital	−0.66	[−1.35, −0.03]
education_digital+exercise_digital	−0.62	[−1.17, −0.10]
exercise_digital	−0.55	[−1.04, −0.08]
exercise_digital+physiotherapy	−0.54	[−1.61, 0.46]
exercise_inperson	−0.45	[−1.05, 0.14]
education_digital+education_inperson	−0.45	[−0.98, 0.07]
education_inperson	−0.38	[−0.83, 0.06]
psychological_digital+usual_care	−0.18	[−0.87, 0.55]
usual_care	−0.13	[−0.73, 0.46]
education_digital+psychological_digital	−0.12	[−0.63, 0.36]
education_digital	−0.07	[−0.36, 0.22]
psychological_digital	−0.05	[−0.40, 0.31]
no_intervention	0.00	[0.00, 0.00]
physiotherapy	0.00	[−0.69, 0.66]
exercise_home	1.12	[0.45, 1.81]
exercise_home+physiotherapy	1.12	[−0.04, 2.28]

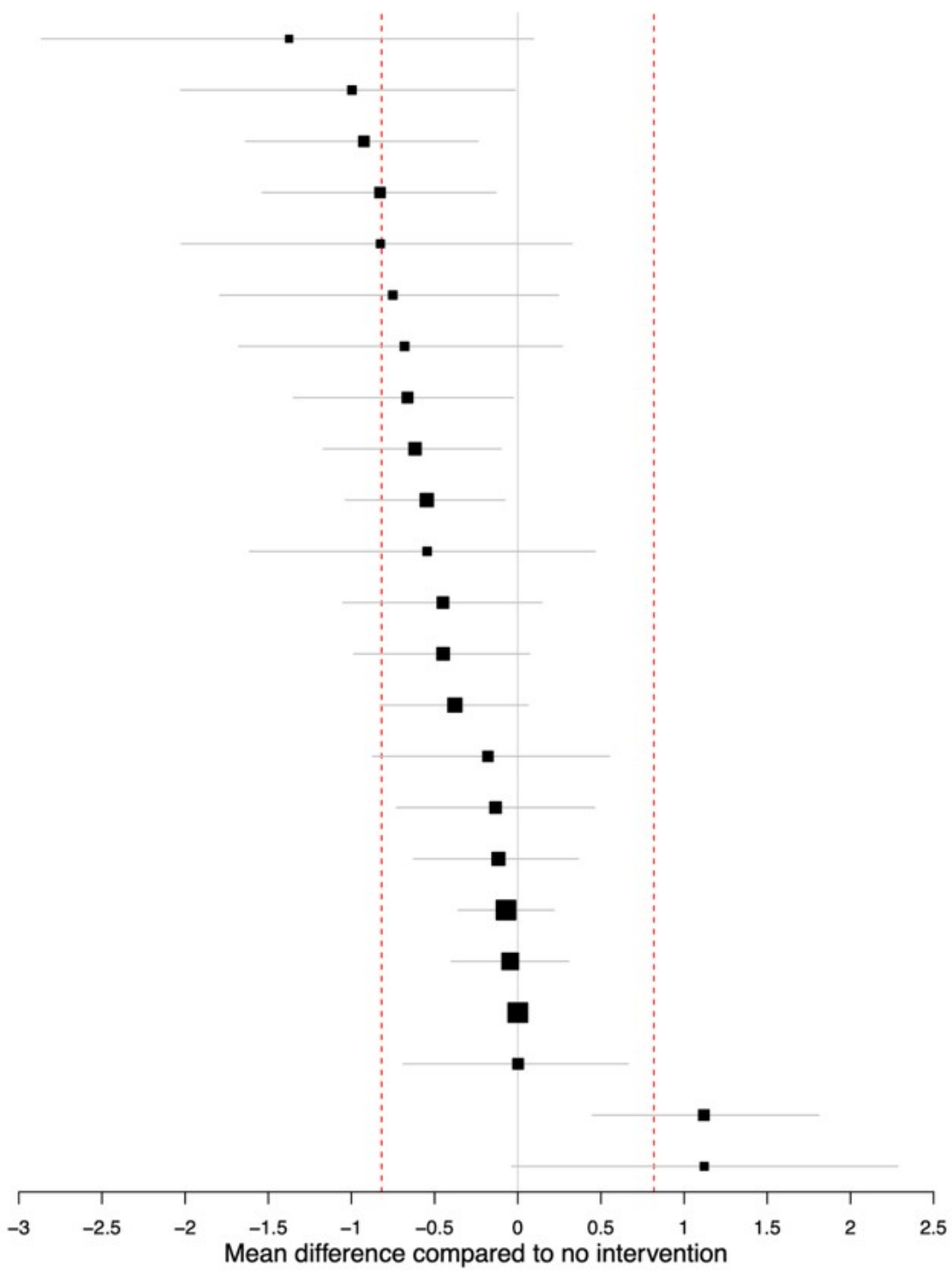

Supplemental Figure 63 – Forest plot of relative effects for the additive pain intensity immediate subgroup chronic pain network on the intervention level with 95%-credible intervals; red dotted lines represent the MCID of 0.82 for this outcome.

SUCRA table

Supplemental Table 32 - SUCRA table of the additive pain intensity immediate subgroup chronic patients network on the intervention level

Intervention	Mean Rank	2.5% CI	97.5% CI	SUCRA
exercise_digital+exercise_inperson+psychological_inperson	3.26	1.00	18.00	0.90
exercise_digital+exercise_inperson	4.12	1.00	14.00	0.86
exercise_digital+education_inperson	4.39	1.00	11.00	0.85
education_inperson+exercise_inperson	5.76	2.00	12.00	0.79
education_inperson+exercise_inperson+physiotherapy	6.72	1.00	19.00	0.75
education_digital+exercise_digital+usual_care	7.21	1.00	18.00	0.73
education_digital+exercise_digital+psychological_digital	7.88	2.00	14.00	0.70
exercise_digital+usual_care	8.44	2.00	18.00	0.68
education_digital+exercise_digital	8.62	4.00	13.00	0.67
exercise_digital	10.02	4.00	15.00	0.61
exercise_digital+physiotherapy	10.68	3.00	20.00	0.58
education_digital+education_inperson	11.01	2.00	18.00	0.56
exercise_inperson	11.72	5.00	19.00	0.53
education_inperson	12.30	3.00	20.00	0.51
psychological_digital+usual_care	15.71	8.00	21.00	0.36
education_digital+psychological_digital	16.12	6.98	21.00	0.34
usual_care	16.62	11.00	21.00	0.32
education_digital	17.03	9.00	21.00	0.30
psychological_digital	17.40	8.00	21.00	0.29
physiotherapy	17.95	11.00	21.00	0.26
no_intervention	18.21	11.00	21.00	0.25
exercise_home+physiotherapy	22.34	20.00	23.00	0.07
exercise_home	22.49	22.00	23.00	0.07

Subgroup analysis: Fully digital interventions

Component level

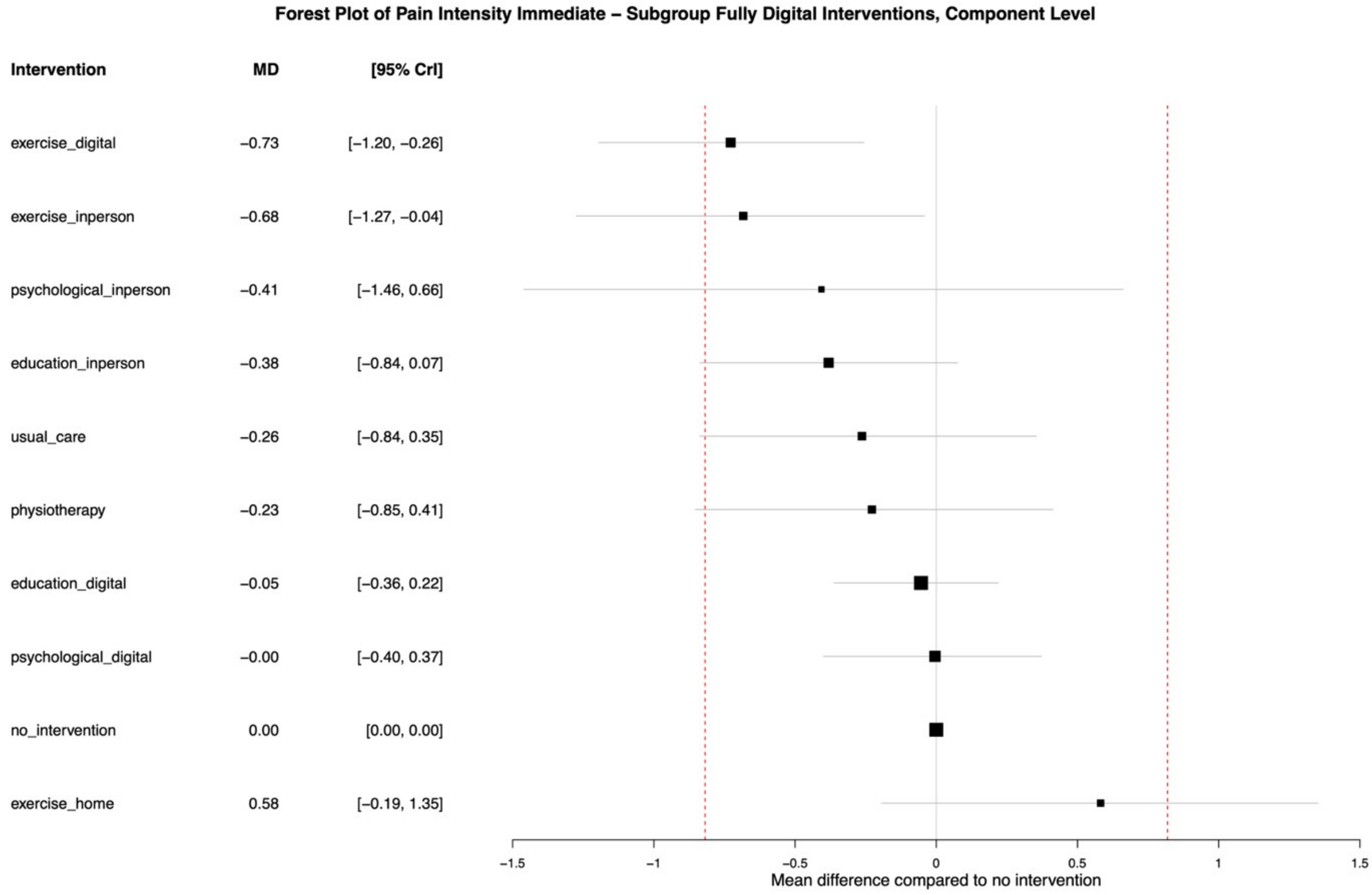

Supplemental Figure 64 – Forest plot of relative effects for the additive pain intensity immediate subgroup fully digital interventions network on the component level with 95%-credible intervals; red dotted lines represent the MCID of 0.82 for this outcome.

SUCRA table

Supplemental Table 33 - SUCRA table of the additive pain intensity immediate subgroup fully digital interventions network on the component level

Intervention	Mean Rank	2.5% CI	97.5% CI	SUCRA
exercise_digital	1.93	1.00	4.00	0.91
exercise_inperson	2.36	1.00	6.00	0.86
education_inperson	4.17	1.00	8.00	0.68
psychological_inperson	4.47	1.00	10.00	0.65
usual_care	5.21	2.00	9.00	0.58
physiotherapy	5.49	2.00	9.00	0.55
education_digital	6.87	4.00	9.00	0.41
psychological_digital	7.32	4.00	10.00	0.37
no_intervention	7.49	5.00	9.00	0.35
exercise_home	9.70	6.00	10.00	0.13

Intervention level

Forest Plot of Pain Intensity Immediate – Subgroup Fully Digital Interventions

Intervention	MD	[95% CrI]
exercise_digital+exercise_inperson+psychological_inperson	−1.82	[−3.21, −0.31]
exercise_digital+exercise_inperson	−1.41	[−2.34, −0.35]
education_inperson+exercise_inperson+physiotherapy	−1.29	[−2.42, −0.06]
exercise_digital+education_inperson	−1.11	[−1.80, −0.38]
education_inperson+exercise_inperson	−1.06	[−1.78, −0.33]
education_digital+exercise_digital+psychological_digital	−0.79	[−1.51, −0.06]
education_digital+exercise_digital	−0.78	[−1.35, −0.23]
exercise_digital	−0.73	[−1.20, −0.26]
exercise_inperson	−0.68	[−1.27, −0.04]
education_digital+education_inperson	−0.44	[−0.99, 0.07]
education_inperson	−0.38	[−0.84, 0.07]
education_digital+physiotherapy	−0.28	[−1.02, 0.44]
usual_care	−0.26	[−0.84, 0.35]
physiotherapy	−0.23	[−0.85, 0.41]
education_digital+psychological_digital	−0.06	[−0.61, 0.46]
education_digital	−0.05	[−0.36, 0.22]
psychological_digital	−0.00	[−0.40, 0.37]
no_intervention	0.00	[0.00, 0.00]
exercise_home	0.58	[−0.19, 1.35]

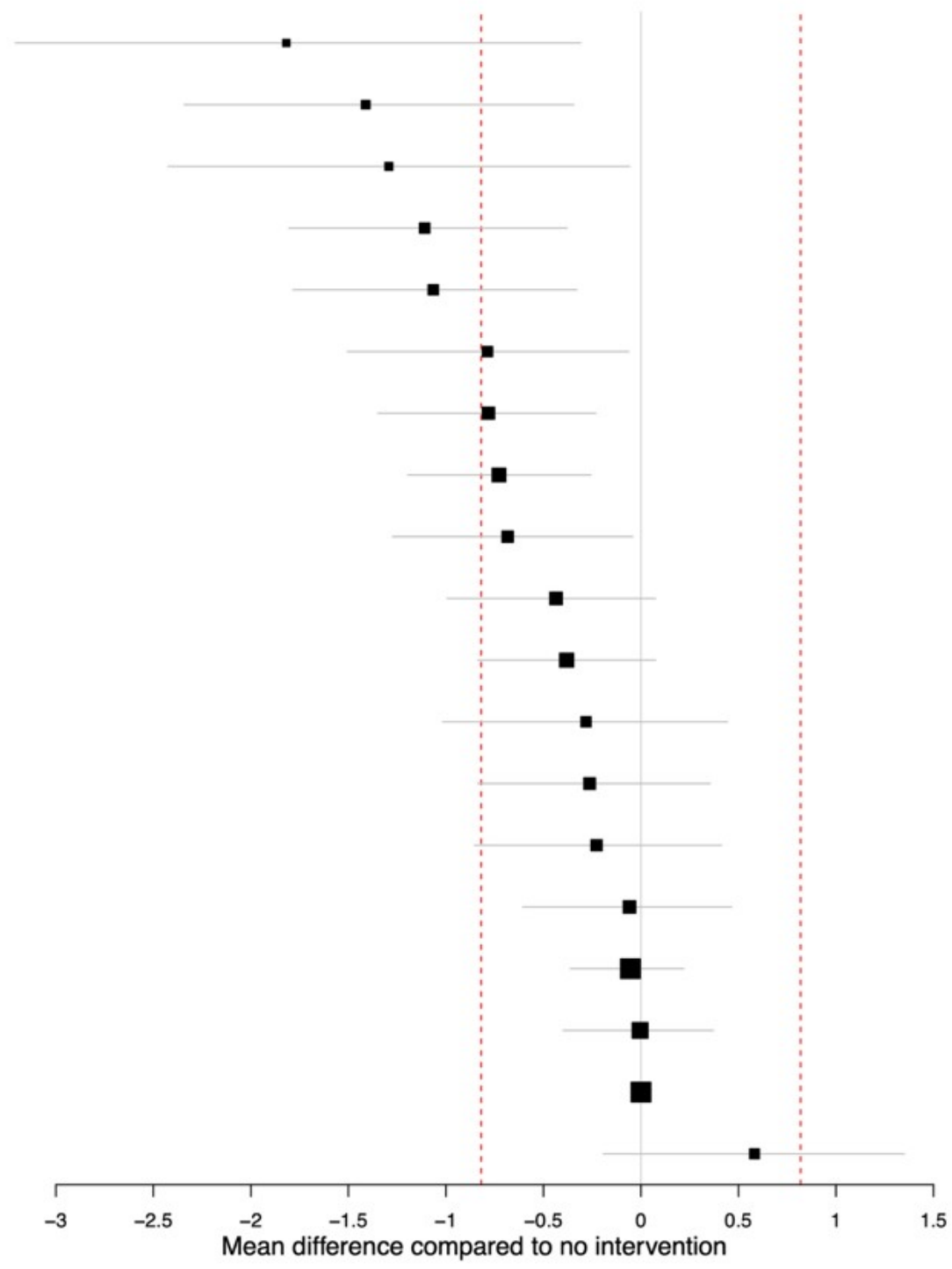

Supplemental Figure 65 – Forest plot of relative effects for the additive pain intensity immediate subgroup fully digital interventions network on the component level with 95%-credible intervals; red dotted lines represent the MCID of 0.82 for this outcome.

SUCRA table

Supplemental Table 34 - SUCRA table of the additive pain intensity immediate subgroup fully digital interventions network on the intervention level

Intervention	Mean Rank	2.5% CI	97.5% CI	SUCRA
exercise_digital+exercise_inperson+psychological_inperson	2.04	1.00	11.00	0.95
exercise_digital+exercise_inperson	2.68	1.00	7.00	0.91
education_inperson+exercise_inperson+physiotherapy	3.66	1.00	12.00	0.86
exercise_digital+education_inperson	4.29	1.00	9.00	0.83
education_inperson+exercise_inperson	4.72	2.00	9.00	0.80
education_digital+exercise_digital	7.05	4.00	10.00	0.68
education_digital+exercise_digital+psychological_digital	7.15	3.00	12.00	0.68
exercise_digital	7.83	4.00	11.00	0.64
exercise_inperson	8.44	4.00	14.00	0.61
education_digital+education_inperson	10.67	4.00	16.00	0.49
education_inperson	11.47	5.00	17.00	0.45
education_digital+physiotherapy	12.70	7.00	18.00	0.38
usual_care	13.00	8.00	18.00	0.37
physiotherapy	13.47	9.00	18.00	0.34
education_digital+psychological_digital	15.07	9.00	19.00	0.26
education_digital	15.28	11.00	18.00	0.25
psychological_digital	15.85	10.00	19.00	0.22
no_intervention	15.96	11.00	18.00	0.21
exercise_home	18.66	15.00	19.00	0.07

Sensitivity analysis: Excluding studies with less than 10 participants in one arm

Component level

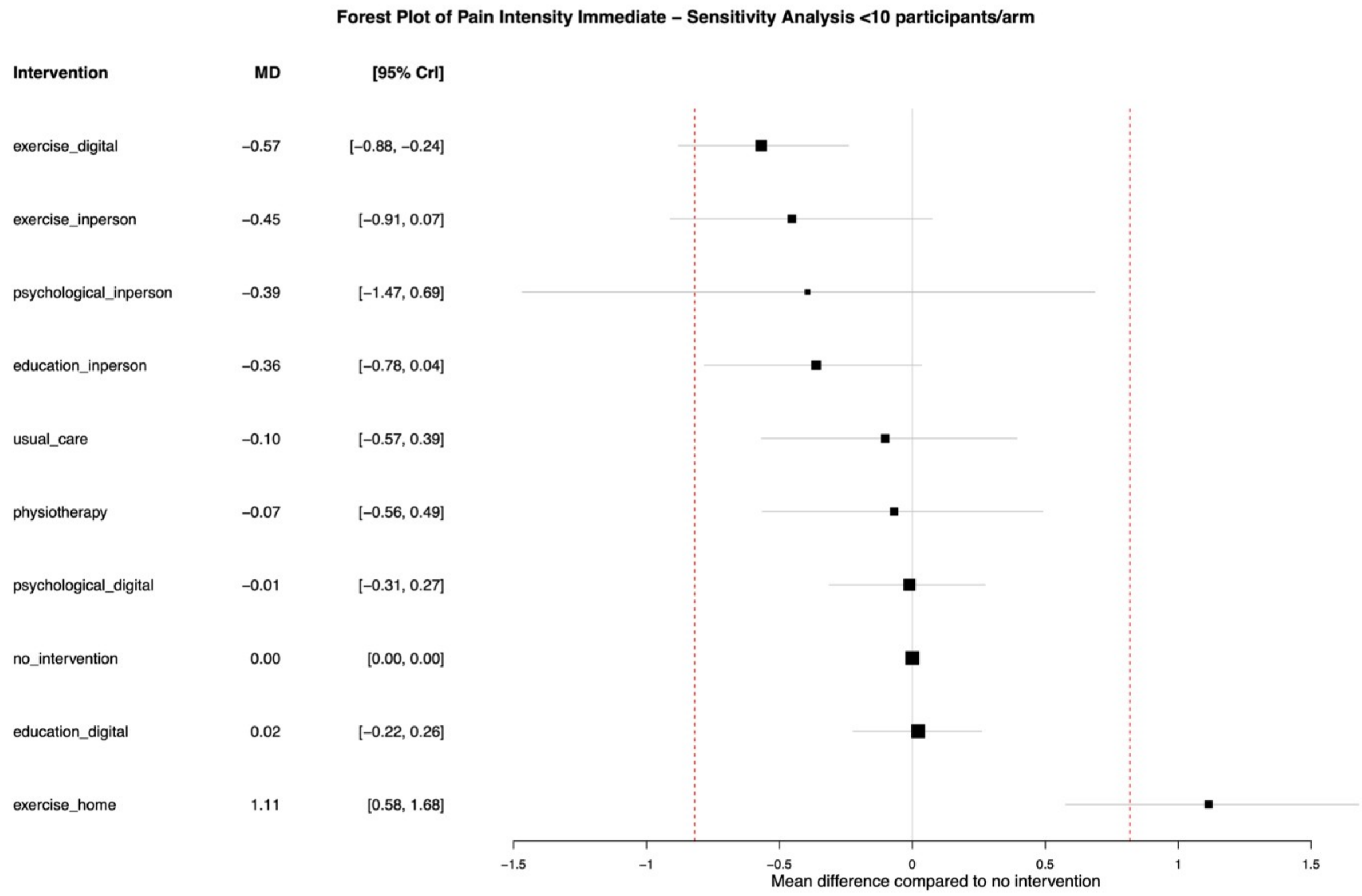

Supplemental Figure 66 - Forest plot of relative effects for the additive pain intensity immediate sensitivity analysis <10 participants in one arm network on the component level with 95%-credible intervals; red dotted lines represent the MCID of 0.82 for this outcome.

SUCRA table

Supplemental Table 35 - SUCRA table of the additive pain intensity immediate sensitivity analysis <10 participants in one arm network on the component level

Intervention	Mean Rank	2.5% CI	97.5% CI	SUCRA
exercise_digital	1.93	1.00	4.00	0.91
exercise_inperson	2.91	1.00	7.00	0.81
education_inperson	3.47	1.00	7.00	0.75
psychological_inperson	3.93	1.00	9.00	0.71
usual_care	5.84	2.98	9.00	0.52
physiotherapy	6.12	3.00	9.00	0.49
psychological_digital	6.75	3.00	9.00	0.43
no_intervention	6.90	4.00	9.00	0.41
education_digital	7.16	4.00	9.00	0.38
exercise_home	9.99	10.00	10.00	0.10

Intervention level

Forest Plot of Pain Intensity Immediate – Sensitivity Analysis <10 participants/arm

Intervention	MD	[95% CrI]
exercise_digital+exercise_inperson+psychological_inperson	−1.42	[−2.70, −0.16]
exercise_digital+exercise_inperson	−1.02	[−1.71, −0.27]
exercise_digital+education_inperson	−0.93	[−1.48, −0.37]
education_inperson+exercise_inperson+physiotherapy	−0.88	[−1.74, 0.10]
education_inperson+exercise_inperson	−0.81	[−1.39, −0.19]
exercise_digital+usual_care	−0.67	[−1.33, 0.02]
education_digital+exercise_digital+usual_care	−0.65	[−1.33, 0.08]
exercise_digital+physiotherapy	−0.64	[−1.35, 0.16]
education_digital+exercise_digital+physiotherapy	−0.61	[−1.31, 0.14]
exercise_digital	−0.57	[−0.88, −0.24]
education_digital+exercise_digital+psychological_digital	−0.56	[−0.99, −0.09]
education_digital+exercise_digital	−0.55	[−0.88, −0.22]
exercise_inperson	−0.45	[−0.91, 0.07]
education_inperson	−0.36	[−0.78, 0.04]
education_digital+education_inperson	−0.34	[−0.83, 0.12]
psychological_digital+usual_care	−0.11	[−0.68, 0.47]
usual_care	−0.10	[−0.57, 0.39]
physiotherapy	−0.07	[−0.56, 0.49]
education_digital+physiotherapy	−0.05	[−0.56, 0.52]
psychological_digital	−0.01	[−0.31, 0.27]
no_intervention	0.00	[0.00, 0.00]
education_digital+psychological_digital	0.01	[−0.40, 0.39]
education_digital	0.02	[−0.22, 0.26]
exercise_home+physiotherapy	1.05	[0.17, 1.97]
exercise_home	1.11	[0.58, 1.68]

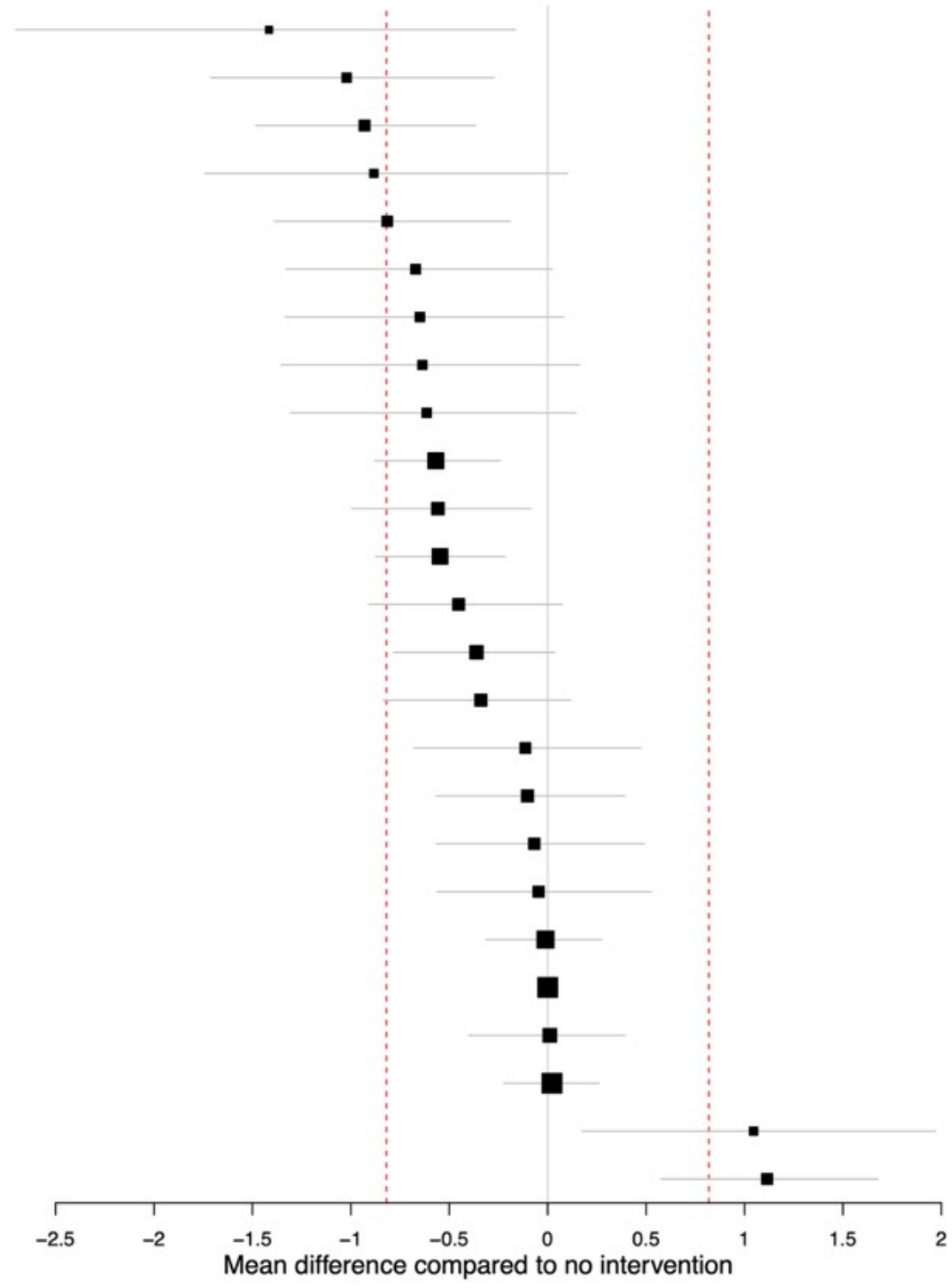

Supplemental Figure 67 - Forest plot of relative effects for the additive pain intensity immediate sensitivity analysis <10 participants in one arm network on the intervention level with 95%-credible intervals; red dotted lines represent the MCID of 0.82 for this outcome.

SUCRA table

Supplemental Table 36 - SUCRA table of the additive pain intensity immediate sensitivity analysis <10 participants in one arm network on the intervention level

Intervention	Mean Rank	2.5% CI	97.5% CI	SUCRA
exercise_digital+exercise_inperson+psychological_inperson	2.79	1.00	15.00	0.93
exercise_digital+exercise_inperson	3.49	1.00	11.00	0.90
exercise_digital+education_inperson	4.17	1.00	10.03	0.87
education_inperson+exercise_inperson+physiotherapy	5.40	1.00	18.00	0.82
education_inperson+exercise_inperson	5.86	2.00	13.00	0.81
exercise_digital+usual_care	8.17	2.00	17.00	0.71
education_digital+exercise_digital+usual_care	8.55	2.00	17.00	0.70
exercise_digital+physiotherapy	8.93	3.00	19.00	0.68
education_digital+exercise_digital+physiotherapy	9.30	3.00	19.00	0.67
exercise_digital	9.72	4.00	15.00	0.65
education_digital+exercise_digital+psychological_digital	9.82	3.00	15.00	0.65
education_digital+exercise_digital	10.08	5.00	14.00	0.64
exercise_inperson	12.07	5.00	20.00	0.56
education_inperson	13.20	4.00	20.00	0.51
education_digital+education_inperson	13.61	4.00	21.00	0.50
psychological_digital+usual_care	17.63	10.00	23.00	0.33
usual_care	17.96	12.00	23.00	0.32
physiotherapy	18.33	12.00	23.00	0.31
education_digital+physiotherapy	18.74	12.00	23.00	0.29
psychological_digital	19.23	12.00	23.00	0.27
no_intervention	19.54	14.00	23.00	0.26
education_digital+psychological_digital	19.57	11.00	23.00	0.26
education_digital	19.91	13.00	23.00	0.24
exercise_home+physiotherapy	24.31	23.00	25.00	0.07
exercise_home	24.61	24.00	25.00	0.06

Network meta-regression sample size

Supplemental Table 37 – Results of the sample size network meta-regression, incl. modelfit parameters

Model	Number of effective parameters	Posterior mean of between-study SD	Residual Deviance	DIC	B	95% CrI of B
Meta-Regression Sample Size	50.03	0.12	69.54	68.96	0.00	0;0.06

DIC = deviance information criterion, CrI = credible interval

Sensitivity analysis: No intervention and waiting list as separate nodes

Intervention level

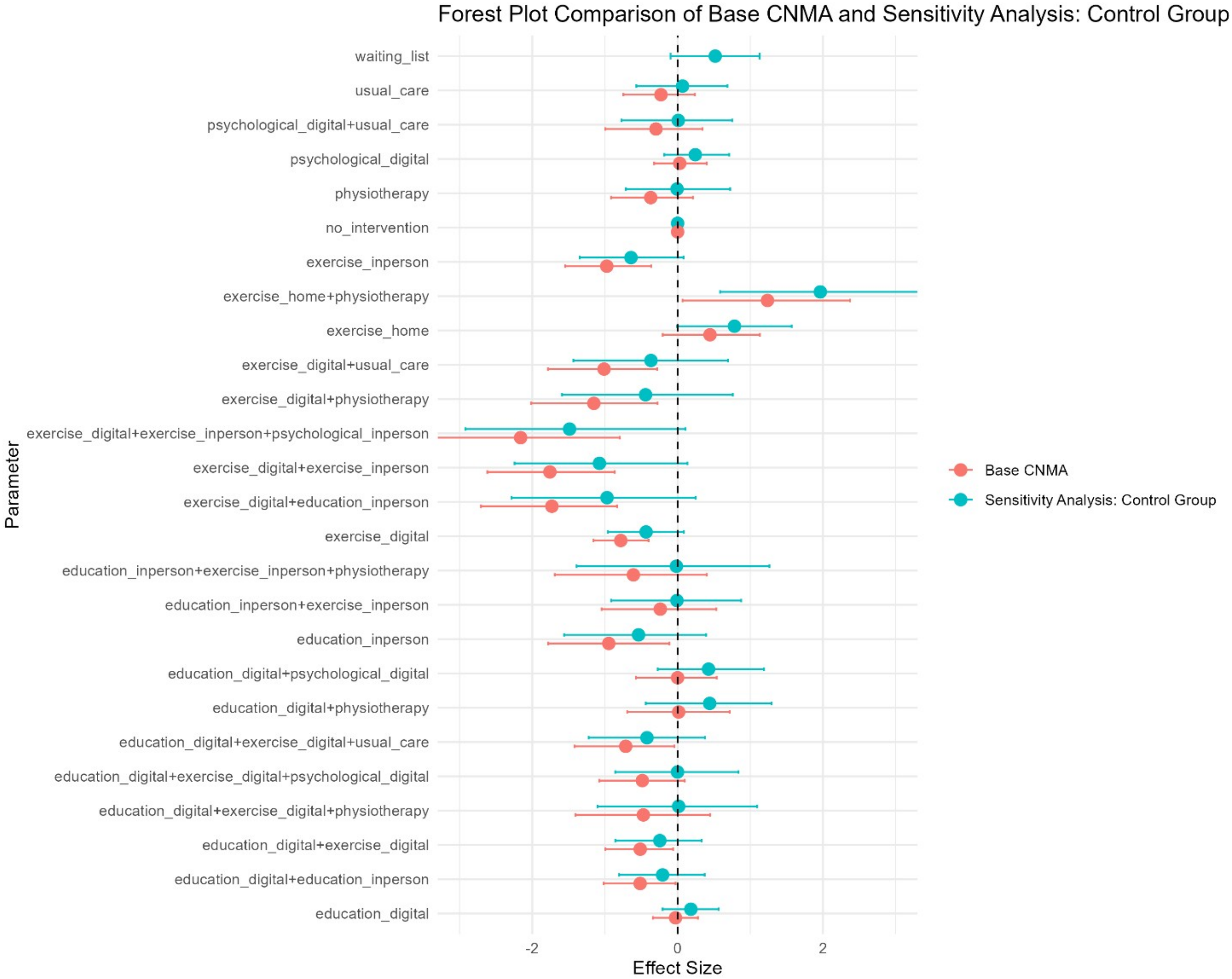

Supplemental Figure 68 - Forest plot of relative effects for the interaction pain intensity immediate sensitivity analysis "no intervention and waiting list as separate nodes" network on the intervention level with 95%-credible intervals compared to the base CNMA model

Sensitivity Analysis: Correlation coefficient for imputing missing SDs – 0.40

Intervention level

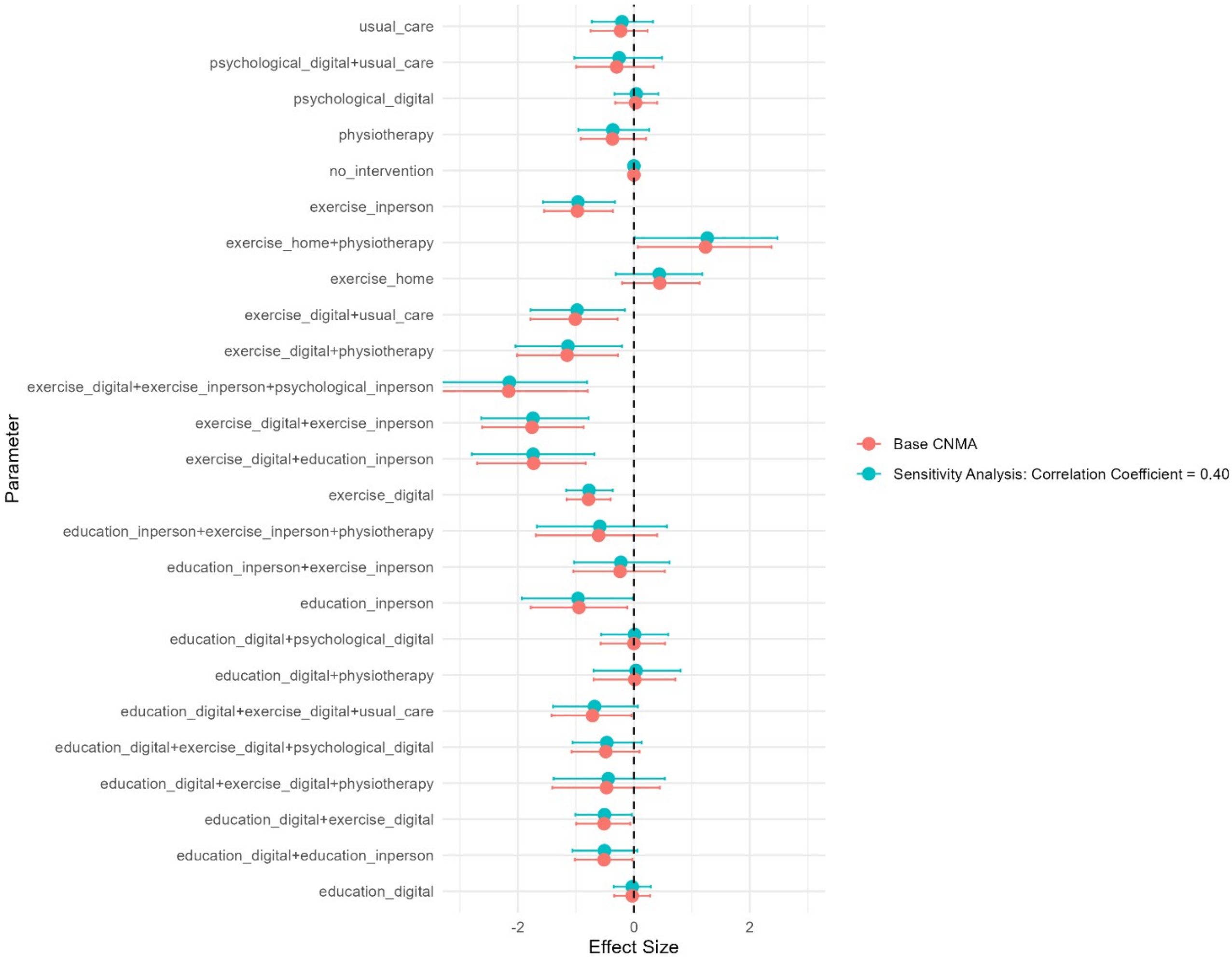

Supplemental Figure 69 - Forest plot of relative effects for the interaction pain intensity immediate sensitivity analysis "correlation coefficient for imputing missing SDs – 0.40" network on the intervention level with 95%-credible intervals compared to the base CNMA model

Sensitivity Analysis: Correlation coefficient for imputing missing SDs – 0.81

Intervention level

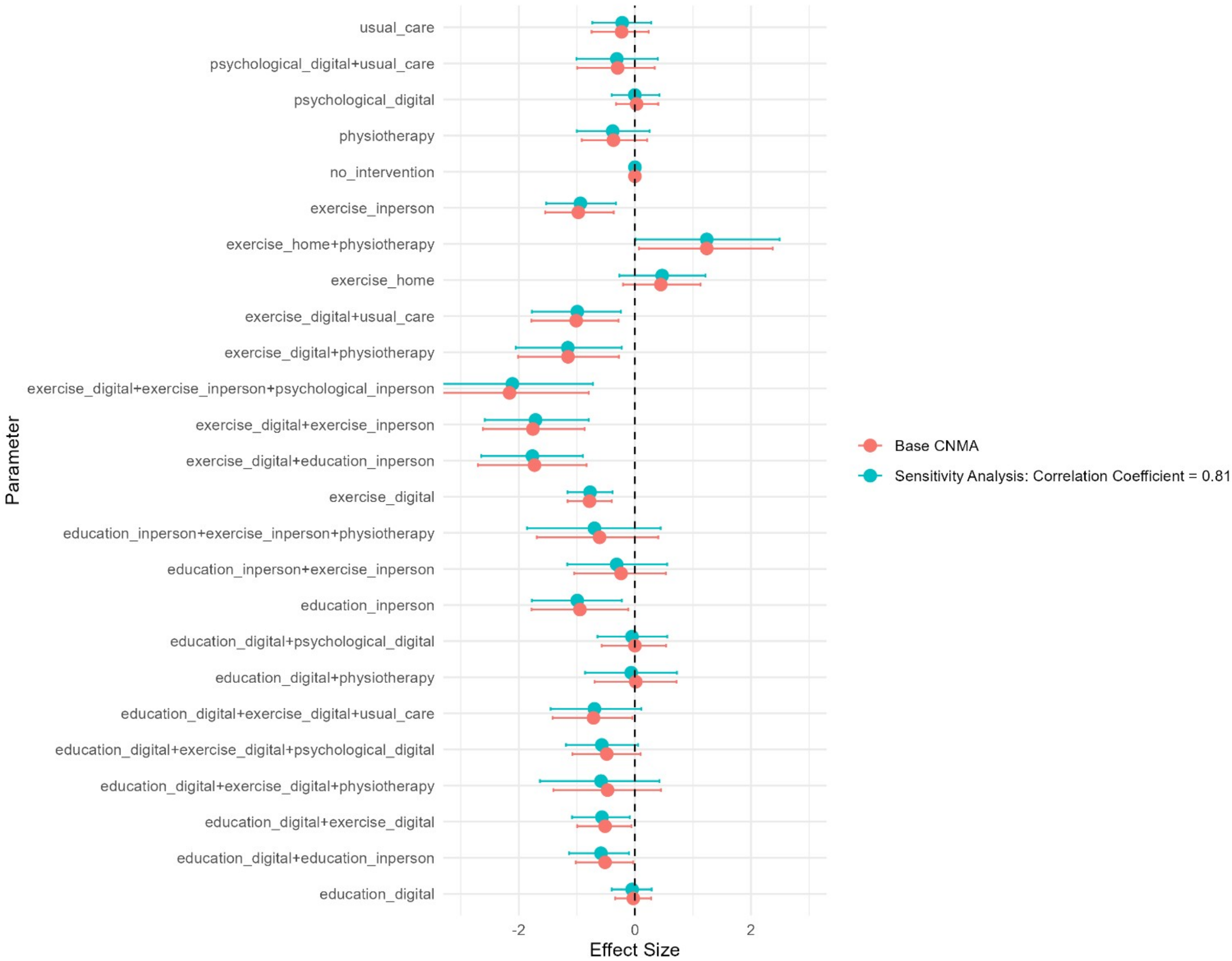

Supplemental Figure 70 - Forest plot of relative effects for the interaction pain intensity immediate sensitivity analysis "correlation coefficient for imputing missing SDs – 0.81" network on the intervention level with 95%-credible intervals compared to the base CNMA model

Sensitivity Analysis: Informative prior for between-trial standard deviation

Intervention level

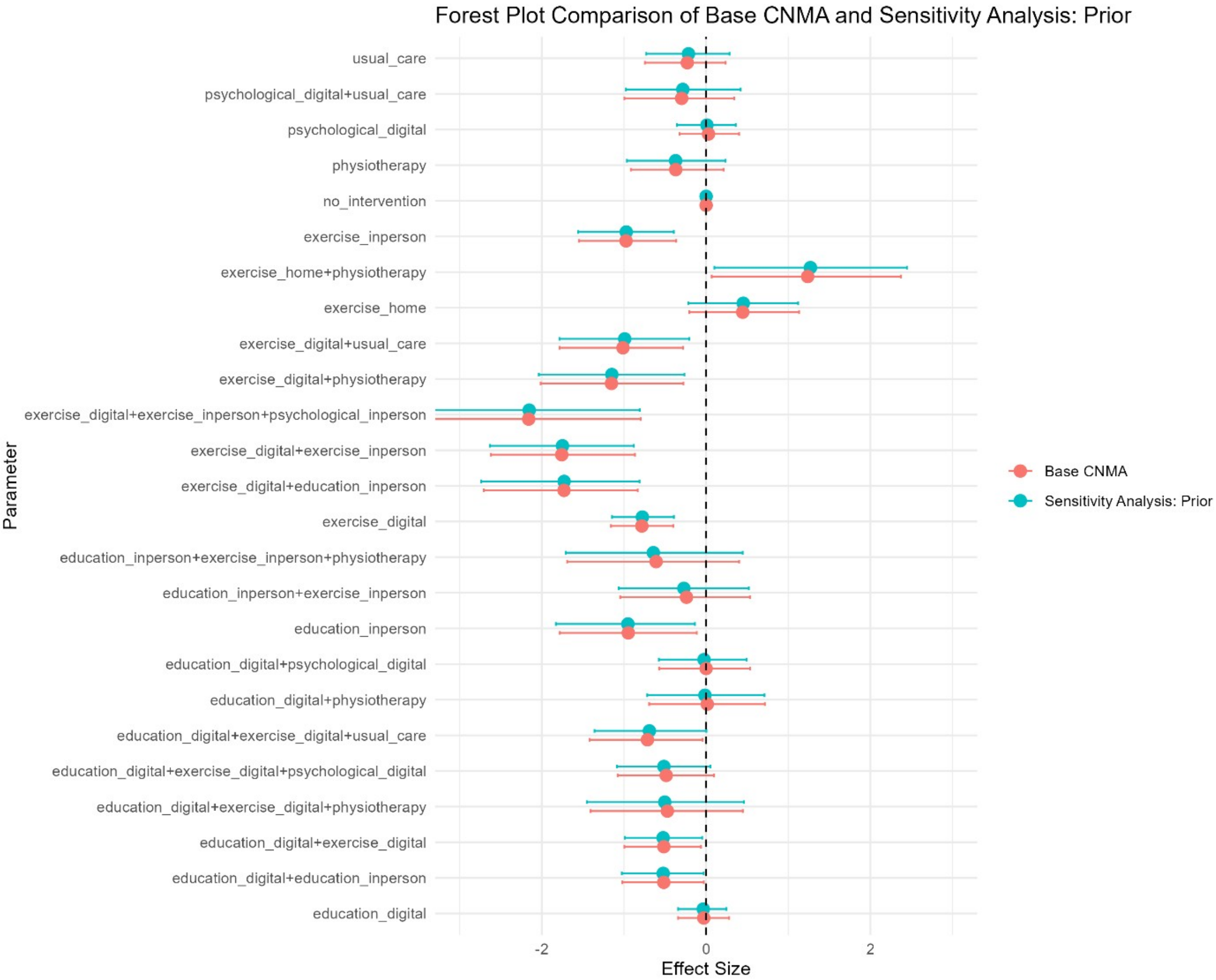

Supplemental Figure 71 - Forest plot of relative effects for the interaction pain intensity immediate sensitivity analysis "informative prior for tau" network on the intervention level with 95%-credible intervals compared to the base CNMA model

Supplemental Data 25 – Results of threshold analyses

Disability immediate

Study (Contrast)	SMD	95% Confidence Interval		Invariant Interval	
Akodu 2024 (3 vs 7)	0.02	(−0.44, 0.49)	18	(−13.71, 26.88)	13
Amorim 2019 (5 vs 11)	−0.04	(−0.64, 0.55)	10	(−11.77, 28.59)	14
Bernardelli 2020 (3 vs 7)	−0.29	(−0.78, 0.19)	2	(−143.03, 59.83)	19
Carpenter 2012 (1 vs 8)	−0.43	(−0.79, −0.08)	18	(−3.96, NT)	–
Chhabra 2018 (16 vs 21)	−1.21	(−1.61, −0.82)	21	(−2.94, 5.91)	16
Chiauzzi 2010 (4 vs 2)	0.08	(−0.37, 0.53)	21	(−12.20, 8.16)	16
Cui 2023 (28 vs 12)	−0.05	(−0.99, 0.89)	–	(NT, 35.98)	16
Day 2024 (2 vs 4)	−0.14	(−0.32, 0.05)	16	(−0.69, 6.66)	26
DelPozo−Cruz 2012 (25 vs 16)	1.84	(1.55, 2.13)	16	(0.34, 6.31)	7
Fanuscu 2024 (3 vs 7)	−0.41	(−1.21, 0.40)	16	(−5.65, 53.46)	26
Garreta−Catala 2023 (16 vs 12)	−0.31	(−1.39, 0.77)	7	(−315.70, 45.49)	21
Geraghty 2024 (16 vs 19)	−0.12	(−0.29, 0.04)	14	(−6.23, 0.18)	21
Hodges 2021 (2 vs 1)	−0.17	(−0.43, 0.08)	3	(−11.02, 7.02)	7
Itoh 2022 (16 vs 25)	−0.39	(−0.90, 0.12)	19	(−3.01, NT)	–
Koppenaal 2022 (14 vs 23)	−0.03	(−0.58, 0.51)	27	(−1.91, 1.90)	7
Krein 2013 (16 vs 6)	−0.22	(−0.52, 0.09)	21	(−0.96, 3.51)	27
Lara−Palomo 2022 (26 vs 20)	−0.43	(−0.74, −0.13)	18	(−2.29, 2.98)	16
Mbada 2019 (17 vs 6)	0.28	(−0.23, 0.80)	16	(−2.43, 3.89)	18
Mesa−Castrillon 2023 (16 vs 3)	−0.43	(−0.84, −0.02)	18	(−26.15, 35.96)	6
Özden 2022 (9 vs 3)	−0.29	(−0.58, −0.00)	21	(−3.67, 29.54)	7
Park 2023 (14 vs 3)	0.28	(0.05, 0.52)	16	(−18.22, 32.19)	24
Petrozzi 2019 (24 vs 16)	0.09	(−0.25, 0.42)	7	(−53.52, 158.15)	2
Sandal 2021 (25 vs 16)	0.09	(−0.08, 0.26)	15	(−13.99, 11.40)	21
Toelle 2019 (18 vs 12)	0.00	(−0.38, 0.38)	12	(−3.42, 14.62)	13
Villatoro−Luque 2023 (17 vs 6)	0.09	(−0.22, 0.40)	21	(−5.53, 8.12)	12
Williams 2018 (16 vs 10)	0.06	(−0.41, 0.54)	23	(−3.67, 2.03)	19
Yang 2019 (14 vs 20)	−0.28	(−1.56, 0.99)	10	(−5.51, NT)	–
Yang 2024 (1 vs 3)	−0.76	(−0.99, −0.54)	1	(−3.02, −0.01)	21
Zheng 2022 a (3 vs 6)	0.00	(−0.33, 0.33)	23	(−11.89, 11.00)	18
**Zheng 2022 b (15 vs 27)**	−0.65	(−1.21, −0.09)	7	(−0.84, NT)	–
Goode 2018 (22 vs 13)	−0.46	(−1.00, 0.08)	16	(−34.10, 37.99)	7
Goode 2018 (1 vs 13)	−0.89	(−1.44, −0.34)	13	(−139.20, 94.50)	18
Karaduman 2023 (3 vs 7)	−0.68	(−1.27, −0.09)	18	(−1.34, 8.21)	7
Karaduman 2023 (9 vs 7)	−1.28	(−1.86, −0.70)	21	(−3.75, NT)	–
Okudan 2024 (6 vs 3)	0.35	(−0.17, 0.86)	27	(−66.06, 32.76)	21
Okudan 2024 (1 vs 3)	−1.32	(−1.92, −0.72)	19	(−8.38, 12.58)	14

○ SMD — 95% Confidence Interval ▬ Invariant Interval

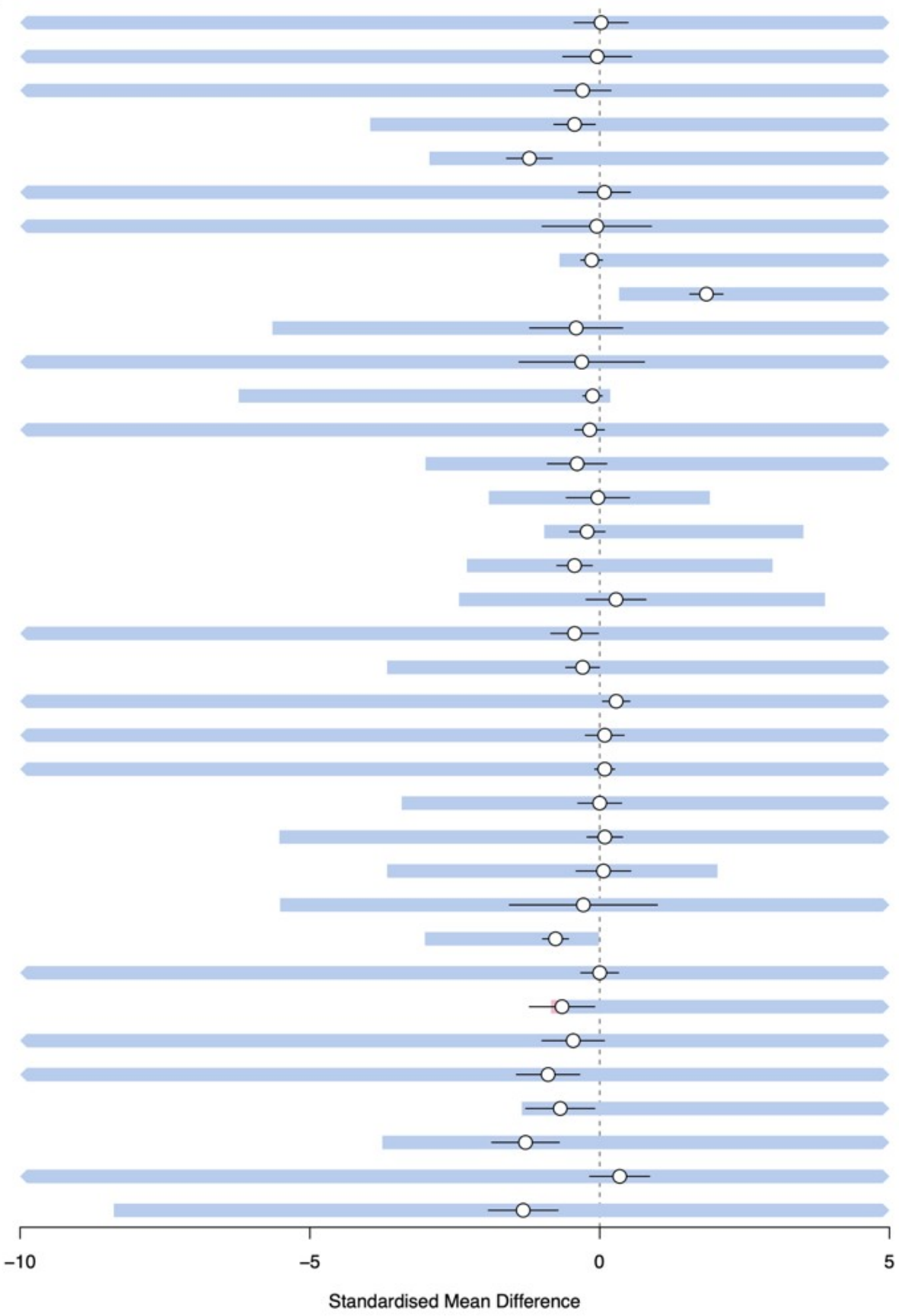

Supplemental Figure 72 - Threshold analysis for the outcome disability. Each row is showing treatment effects per study alongside 95% confidence intervals and invariant intervals (blue shaded bars). If the 95% confidence interval exceeds the invariant interval, this study is considered sensitive to change in the evidence which might alter the treatment ranking. The treatment that would be first-ranked in case of changing evidence is shown left and right from the invariant interval. Bold study labels and red shaded bars indicate where the 95% confindence interval exceeds the threshold. NT = no threshold in this direction could change the evidence. no_intervention = 1, education_digital = 2, exercise_digital = 3, psychological_digital = 4, education_inperson = 5, education_digital+exercise_digital = 6, exercise_inperson = 7, education_digital+psychological_digital = 8, exercise_home = 9, education_digital+education_inperson = 10, exercise_digital+education_inperson = 11, education_digital+exercise_digital+psychological_digital = 12, education_digital+exercise_home = 13, physiotherapy = 14, exercise_digital+exercise_inperson = 15, usual_care = 16, education_inperson+exercise_inperson = 17, education_digital+physiotherapy = 18, education_digital+usual_care = 19, exercise_digital+physiotherapy = 20, exercise_digital+usual_care = 21, education_digital+psychological_digital+exercise_home = 22, education_digital+exercise_digital+physiotherapy = 23, psychological_digital+usual_care = 24, education_digital+exercise_digital+usual_care = 25, exercise_home+physiotherapy = 26, exercise_digital+exercise_inperson+psychological_inperson = 27, education_inperson+exercise_inperson+physiotherapy = 28

Pain intensity immediate

Study (Contrast)	SMD	95% Confidence Interval		Invariant Interval	
Bayattork 2022 (7 vs 3)	−0.00	(−0.53, 0.53)	14	(−8.14, 0.95)	11
Bayattork 2022 (12 vs 3)	0.70	(0.07, 1.33)	11	(−0.81, 20.84)	14
**Okudan 2024 (6 vs 3)**	0.10	(−0.80, 1.00)	14	(−18.23, 0.95)	11
Okudan 2024 (1 vs 3)	0.80	(−0.35, 1.95)	11	(−1.24, 29.41)	14
Shaygan 2022 (10 vs 5)	0.10	(−0.76, 0.96)	14	(−34.20, 97.26)	20
Shaygan 2022 (1 vs 5)	1.30	(0.43, 2.17)	4	(−41.51, 41.68)	14
Karaduman 2023 (3 vs 7)	0.60	(−0.02, 1.22)	19	(−3.62, 6.32)	18
Karaduman 2023 (9 vs 7)	1.70	(1.12, 2.28)	18	(−1.26, 8.93)	19
Hodges 2021 (2 vs 1)	2.00	(−2.80, 6.80)	19	(−360.41, 3681.04)	14
Chiauzzi 2010 (4 vs 2)	−0.30	(−0.80, 0.20)	11	(−2.90, 140.39)	23
Mesa−Castrillon 2023 (15 vs 3)	0.90	(0.06, 1.74)	9	(−11.30, 8.39)	11
Park 2023 (12 vs 3)	0.30	(−0.28, 0.88)	11	(−11.22, 30.38)	4
Pongsatornsaiklang 2025 (15 vs 3)	0.53	(−0.14, 1.19)	19	(−17.07, 10.72)	20
Yang 2024 (1 vs 3)	0.90	(0.51, 1.29)	25	(0.02, 27.83)	18
Özden 2022 (9 vs 3)	0.80	(−0.41, 2.01)	25	(−8.46, 137.32)	18
Day 2024 (2 vs 4)	−0.23	(−0.68, 0.21)	14	(−13.03, 1.89)	11
Krein 2013 (15 vs 6)	0.10	(−0.38, 0.58)	11	(−0.78, 5.47)	14
Mbada 2019 (16 vs 6)	0.40	(−0.64, 1.44)	1	(−256.07, 21.87)	11
Villatoro−Luque 2023 (16 vs 6)	0.20	(−0.69, 1.09)	14	(−15.94, 21.44)	11
Zheng 2022 a (3 vs 6)	0.50	(−0.41, 1.41)	19	(−24.00, 5.19)	11
Fanuscu 2024 (3 vs 7)	−0.97	(−2.18, 0.24)	11	(−9.76, NT)	–
Carpenter 2012 (1 vs 8)	0.00	(−0.50, 0.50)	25	(−1.14, 1.95)	11
Schaller 2016 (2 vs 10)	0.10	(−0.19, 0.39)	17	(−1.25, 7.75)	13
Williams 2018 (15 vs 10)	0.40	(−0.24, 1.04)	21	(−6.69, 191.75)	5
Amorim 2019 (5 vs 11)	0.40	(−0.93, 1.73)	22	(−37.34, 972.88)	8
Cui 2023 (25 vs 13)	−0.30	(−2.00, 1.40)	23	(−34.23, 30.64)	11
Toelle 2019 (17 vs 13)	0.40	(−0.14, 0.94)	11	(−1.34, 102.71)	23
Petrozzi 2019 (21 vs 15)	−0.30	(−0.97, 0.37)	11	(−3.35, 46.96)	14
Sandal 2021 (22 vs 15)	−0.50	(−0.86, −0.14)	18	(−4.97, 0.20)	20
Lara−Palomo 2022 (23 vs 18)	2.30	(1.64, 2.96)	11	(−8.17, 14.09)	21
Yang 2019 (12 vs 18)	1.60	(−0.98, 4.18)	11	(−308.92, 97.39)	22
**Chhabra 2018 (15 vs 19)**	0.60	(−0.19, 1.39)	14	(0.12, NT)	–
Koppenaal 2022 (12 vs 20)	−0.10	(−1.02, 0.82)	18	(−10.27, 22.59)	11
Sanabria−Mazo 2023 (15 vs 21)	−0.07	(−0.52, 0.39)	11	(−1.41, 39.27)	14
Itoh 2022 (15 vs 22)	0.40	(−0.58, 1.38)	14	(−14.05, 1.49)	11
Zheng 2022 b (14 vs 24)	0.40	(−0.52, 1.32)	11	(−8.67, 718.59)	14

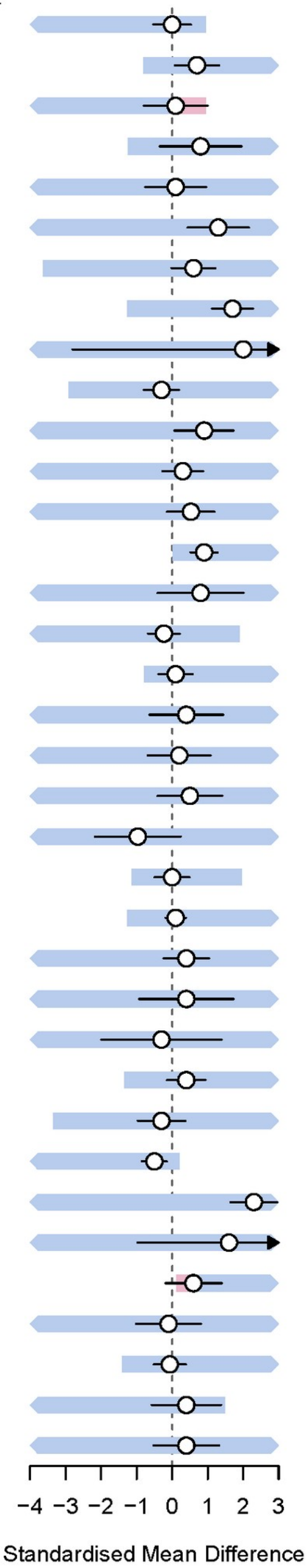

O SMD —— 95% Confidence Interval ▬ Invariant Interval

Supplemental Figure 73 - Threshold analysis for the outcome pain intensity. Each row is showing treatment effects per study alongside 95% confidence intervals and invariant intervals (blue shaded bars). If the 95% confidence interval exceeds the invariant interval, this study is considered sensitive to change in the evidence which might alter the treatment ranking. The treatment that would be first-ranked in case of changing evidence is shown left and right from the invariant interval. Bold study labels and red shaded bars indicate where the 95% confindence interval exceeds the threshold. NT = no threshold in this direction could change the evidence. no_intervention = 1, education_digital = 2, exercise_digital = 3, psychological_digital = 4, education_inperson = 5, education_digital+exercise_digital = 6, exercise_inperson = 7, education_digital+psychological_digital = 8, exercise_home = 9, education_digital+education_inperson = 10, exercise_digital+education_inperson = 11, physiotherapy = 12, education_digital+exercise_digital+psychological_digital = 13, exercise_digital+exercise_inperson = 14, usual_care = 15, education_inperson+exercise_inperson = 16, education_digital+physiotherapy = 17, exercise_digital+physiotherapy = 18, exercise_digital+usual_care = 19, education_digital+exercise_digital+physiotherapy = 20, psychological_digital+usual_care = 21, education_digital+exercise_digital+usual_care = 22, exercise_home+physiotherapy = 23, exercise_digital+exercise_inperson+psychological_inperson = 24, education_inperson+exercise_inperson+physiotherapy = 25

1. Engers, A. J. *et al.* Individual patient education for low back pain. *Cochrane Database of Systematic Reviews* https://doi.org/10.1002/14651858.CD004057.pub3 (2008) doi:10.1002/14651858.CD004057.pub3.
2. Hayden, J., van Tulder, M. W., Malmivaara, A. & Koes, B. W. Exercise therapy for treatment of non-specific low back pain. *Cochrane Database of Systematic Reviews* https://doi.org/10.1002/14651858.CD000335.pub2 (2005) doi:10.1002/14651858.CD000335.pub2.
3. Caspersen, C. J., Powell, K. E. & Christenson, G. M. Physical activity, exercise, and physical fitness: definitions and distinctions for health-related research. *Public Health Rep* **100**, 126–131 (1985).
4. Knoerl, R., Lavoie Smith, E. M. & Weisberg, J. Chronic Pain and Cognitive Behavioral Therapy: An Integrative Review. *West J Nurs Res* **38**, 596–628 (2016).
5. Higgins, J. & Deeks, J. Chapter 6: Choosing effect measures and computing estimates of effect. in *Cochrane Handbook for Systematic Reviews of Interventions version 6.5* (eds Higgins, J. et al.) (Cochrane, 2024).
6. Higgins, J., Eldridge, S. & Li, T. Chapter 23: Including variants on randomized trials [last updated October 2019]. in *Cochrane Handbook for Systematic Reviews of Interventions version 6.5* (eds Higgins, J. et al.) (Cochrane, 2024).
7. Adams, G. *et al.* Patterns of intra-cluster correlation from primary care research to inform study design and analysis. *Journal of Clinical Epidemiology* **57**, 785–794 (2004).
8. Furukawa, T. A., Barbui, C., Cipriani, A., Brambilla, P. & Watanabe, N. Imputing missing standard deviations in meta-analyses can provide accurate results. *Journal of Clinical Epidemiology* **59**, 7–10 (2006).
